# Leukemia incidence trends at the global, regional, and national level between 1990 and 2017

**DOI:** 10.1186/s40164-020-00170-6

**Published:** 2020-06-19

**Authors:** Ying Dong, Oumin Shi, Quanxiang Zeng, Xiaoqin Lu, Wei Wang, Yong Li, Qi Wang

**Affiliations:** 1 Department of Hematology, Maoming People’s Hospital, Maoming, Guangdong 525000 China; 2 Health Science Center, Shenzhen Second People’s Hospital, The First Affiliated Hospital of Shenzhen University, Shenzhen, 518020 China; 3 Digestion Department of Digestion, Maoming People’s Hospital, Maoming, Guangdong 525000 China; 4 Department of Obstetrics and Gynecology, The Second Affiliated Hospital of Zhengzhou University, Zhengzhou, Henan 450014 China; 5 School of Public Health, Zhengzhou University, Zhengzhou, Henan 450014 China; 6 Department of Oncology, Guizhou Provincial People’s Hospital, Guiyang, Guizhou 550002 China; 7 China-Canada Medical and Healthcare Science Association, Toronto, ON L3R 1A3 Canada

**Keywords:** Leukemia, ALL, CLL, AML, CML, Incidence, Global

## Abstract

**Background:**

Leukemias are a group of life-threatening malignant disorders of the blood and bone marrow. The incidence of leukemia varies by pathological types and among different populations.

**Methods:**

We retrieved the incidence data for leukemia by sex, age, location, calendar year, and type from the Global Burden of Disease online database. The estimated average percentage change (EAPC) was used to quantify the trends of the age-standardized incidence rate (ASIR) of leukemia from 1990 to 2017.

**Results:**

Globally, while the number of newly diagnosed leukemia cases increased from 354.5 thousand in 1990 to 518.5 thousand in 2017, the ASIR decreased by 0.43% per year. The number of acute lymphoblastic leukemia (ALL) cases worldwide increased from 49.1 thousand in 1990 to 64.2 thousand in 2017, whereas the ASIR experienced a decrease (EAPC = − 0.08, 95% CI − 0.15, − 0.02). Between 1990 and 2017, there were 55, 29, and 111 countries or territories that experienced a significant increase, remained stable, and experienced a significant decrease in ASIR of ALL, respectively. The case of chronic lymphocytic leukemia (CLL) has increased more than twice between 1990 and 2017. The ASIR of CLL increased by 0.46% per year from 1990 to 2017. More than 85% of all countries saw an increase in ASIR of CLL. In 1990, acute myeloid leukemia (AML) accounted for 18.0% of the total leukemia cases worldwide. This proportion increased to 23.1% in 2017. The ASIR of AML increased from 1.35/100,000 to 1.54/100,000, with an EAPC of 0.56 (95% CI 0.49, 0.62). A total of 127 countries or territories experienced a significant increase in the ASIR of AML. The number of chronic myeloid leukemia (CML) cases increased from 31.8 thousand in 1990 to 34.2 thousand in 2017. The ASIR of CML decreased from 0.75/100,000 to 0.43/100,000. A total of 141 countries or territories saw a decrease in ASIR of CML.

**Conclusions:**

A significant decrease in leukemia incidence was observed between 1990 and 2017. However, in the same period, the incidence rates of AML and CLL significantly increased in most countries, suggesting that both types of leukemia might become a major global public health concern.

## Background

Leukemias are a group of malignant disorders that present with increased numbers of leucocytes in the blood and/or bone marrow. In 2018, it is estimated there were a total of 437.0 thousand new cases of and 309.0 thousand cancer deaths from leukemia worldwide [1]. Leukemia types vary in pathogenesis, origin, incidence, and prognosis. The dominantly presenting leukemia cells can be mature cells, such as in chronic lymphocytic leukemia (CLL); precursor cells of various lineage, such as in the acute leukemias; or both precursor and mature cells, such as in chronic myeloid leukemia (CML) [2, 3]. Among acute leukemia types, acute lymphoblastic leukemia (ALL) is frequently diagnosed in children and young adults, with incidence peaks between 2 and 5 years of age [4], whereas acute myeloid leukemia (AML) is the most common acute type in adults, accounting for 1.3% of new cancer cases in the USA [5]. In addition, a set of rarely diagnosed leukemia types (e.g., atypical chronic myeloid leukemia) differs from the four main types in many aspects [3, 6, 7].

The incidence of leukemia varies among people of different ages, sexes, and races [8]. Such disparities are mainly associated with the levels of exposure to environmental and genetic risk factors. For example, about 10% of individuals who develop CLL have a family history of the disease [9], while ionizing radiation is an established causal exposure for childhood ALL, as evidenced by the modest but significantly elevated risk caused by X-ray pelvimetry during pregnancy [10]. Owing to the previous efforts to combat leukemia, the epidemiology of leukemia might change over time and vary from country to country. Knowing the updated epidemiological data for leukemia and analyzing the temporal trends of leukemia incidence are vital for learning the disease burden of leukemia and assessing the effectiveness of previous prevention strategies. To fill this gap, we used data from the Global Burden of Disease (GBD) 2017 study to analyze the incidence trends of leukemia by sex, location, and type at the global, regional, and national levels.

## Methods

### Data sources

The GBD study provides a tool to quantify health loss from hundreds of diseases, injuries, and risk factors so that health systems can be improved and disparities eliminated [11]. Data from the GBD study have also been widely used to learn the disease burden of cancer [12–16]. In the GBD study framework, the cancer incidence data were retrieved from individual cancer registries or aggregated databases of cancer registry data, such as “Cancer Incidence in Five Continents” (CI5), EUREG, or NORDCAN. Data were excluded if they were not representative of the coverage population (e.g., hospital-based registries), if they did not cover all malignant neoplasms as defined in ICD-9 (140–208) or ICD-10 (C00-C96) (e.g., specialty cancer registry), if they did not include data for both sexes and all age groups, if the data were limited to years prior to 1980, or if the source did not provide details on the population covered. Preference was given to registries with national coverage over those with only local coverage, except for countries where the GBD study provides subnational estimates [17]. The leukemia cases were identified using ICD-10 codes (C91–C93.7, C93.9–C95.2, C95.7–C95.92, Z80.6, Z85.6) and ICD-9 codes (204–208.92, V10.59–V10.69, V16.6). The ALL, CLL, AML, and CML cases were identified using the ICD-10 codes of “C91.0–C91.02,” “C91.1–C91.12,” “C92.0–C92.02, C92.3–C92.62, C93.0–C93.02, C94.0–C94.02, C94.2–C94.22, C94.4–C94.5,” and “C92.1–C92.12″ and the ICD-9 codes of “204.0–204.02,” “204.1–204.12,” “205.0–205.02, 205.3–205.32, 206.0–206.02, 207.0,” and “205.1–205.12, 206.1–206.12, 207.1,” respectively. Leukemias outside the four main types were defined as “other leukemias” and identified using the ICD-10 codes of “C91.2-C91.9, C92.2, C92.7–C92.9, C93.1–C93.9, C94.1, C94.3, C94.6–C95.9″ and the ICD-9 codes of “204.2–204.9, 205.2, 205.8–205.9, 206.2–207, 207.2–208.9.” In the current study, we retrieved the incidence data of leukemia by sex, calendar year, location, and age from the GBD online database [18]. A total of 5 Socio-demographic Index (SDI) regions (e.g., high SDI region), 21 GBD geographic regions (e.g., East Asia), and 195 countries or territories were included. The national SDI values were also retrieved from the GBD online database. SDI provides a composite average of rankings for the incomes per capita, average educational attainment, and fertility rates of all areas in the GBD study.

### Statistical analysis

We used the estimated average percentage change (EAPC) to quantify the trends of the age-standardized incidence rate (ASIR) of leukemia from 1990 to 2017. As described previously, EAPC can be calculated by a regression line, which was fitted to the natural logarithm of the rates, i.e., *y *=* α *+* βx *+* ɛ*, where y = ln(rate) and x = calendar year [19]. If the EAPC is statistically significant but the uncertainty intervals of the GBD estimates overlap, the ASIR is still considered to be stable [20]. The Pearson correlation tests were applied to assess the correlations between SDI values and changing leukemia incidence trends. All statistical analyses were performed using the R program (Version 3.5.3, R core team, Vienna, Austria). A P-value less than 0.05 was considered statistically significant.

## Results

### Leukemia incidence trends at the global level

**Fig. 1:**
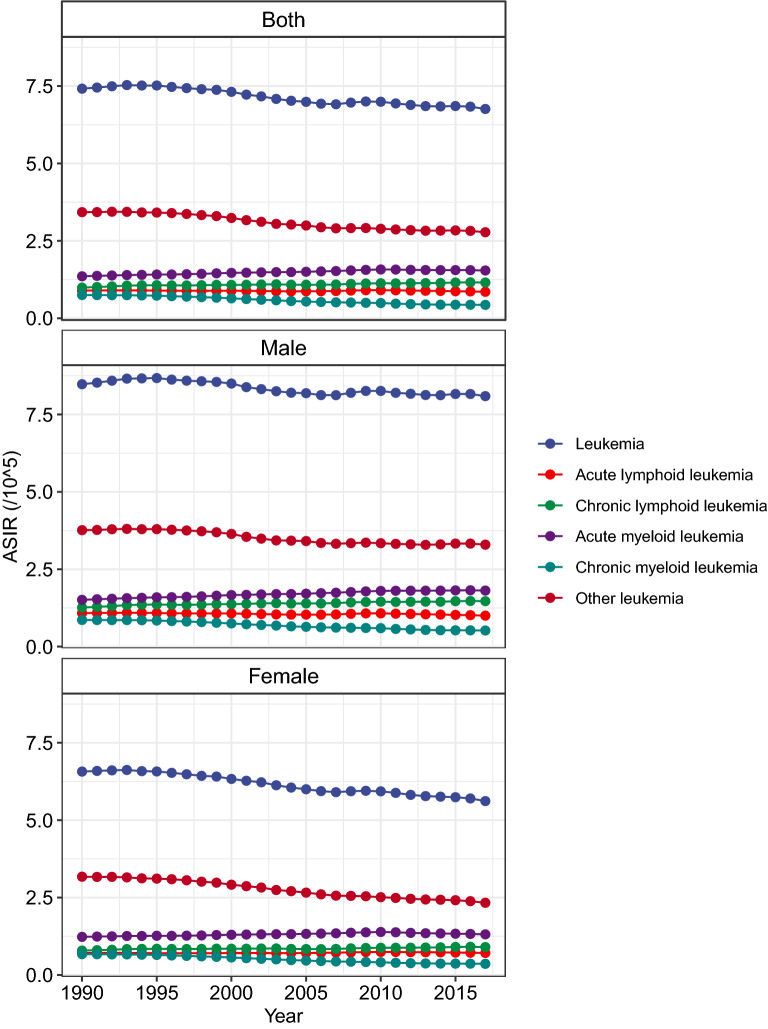
The temporal trends for the age-standardized incidence rate (ASIR) of leukemia by type and sex between 1990 and 2017

**Fig. 2:**
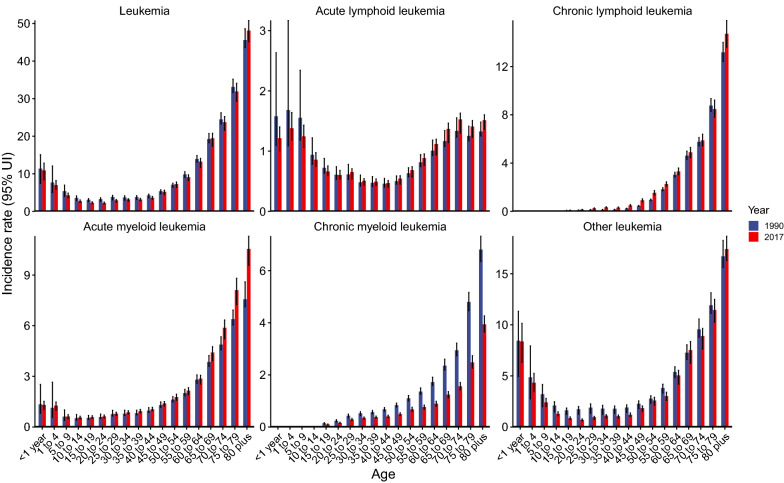
The incidence rate of leukemia by type and year among people of different ages

**Fig. 3:**
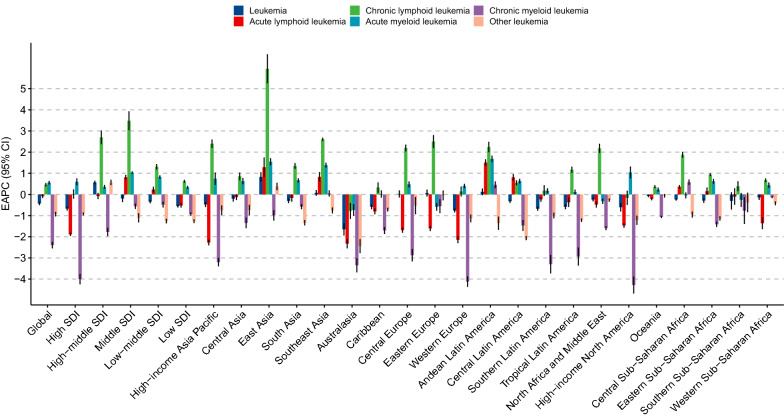
The estimated average percentage change (EAPC) and its 95% confidence interval (CI) of leukemia by type and location between 1990 and 2017

**Fig. 4:**
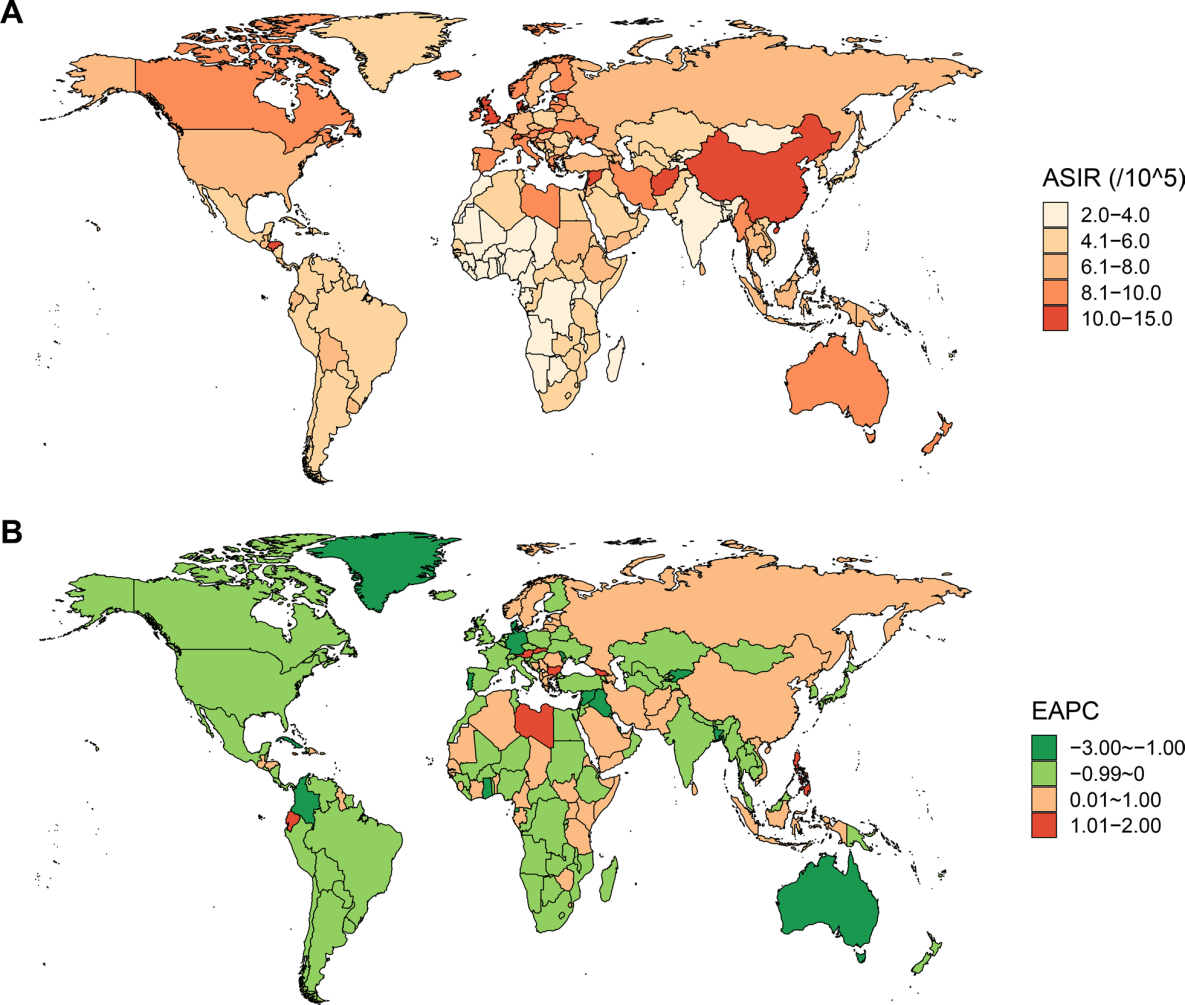
The age-standardized incidence rate (ASIR) of leukemia at the national level in 2017 (**a**) and the estimated average percentage change (EAPC) of leukemia ASIR between 1990 and 2017 (**b**)

Globally, while the number of newly diagnosed leukemia cases increased from 354.5 thousand in 1990 to 518.5 thousand in 2017, the ASIR deceased by 0.43% per year during the same period (Table 1; Fig. 1). The leukemia ASIR was higher in males than in females, whereas females experienced a more pronounced decrease in ASIR during the study period when compared to males (Table 1). Figure 2 displays a J-shaped age distribution of leukemia incidence in both 1990 and 2017. We integrated the 18 age groups into five age groups and found that the leukemia incidence only increased among people aged ≥ 70 years (Table 1). The greatest decrease was observed in people aged 5–14 years. More than half of the total cancer cases occurred in high and high-middle SDI regions. In 1990, the highest incidence occurred in high SDI regions (9.10/100,000), whereas in 2017, the highest incidence occurred in high-middle SDI regions (8.68/100,000). Only high-middle regions experienced a significant increase in leukemia ASIR during the study period (Table 1; Fig. 3). In 1990, the high leukemia ASIR was found in regions with advanced economies, such as Western Europe and Australasia. The ASIR experienced a remarkable decrease in these regions between 1990 and 2017. In 2017, the highest ASIR was seen in East Asia, where the ASIR increased by 0.83% per year (Table 1; Fig. 3). At the national level, the leukemia ASIR was highest in Syria (14.83/100,000), followed by the UK, Denmark, and Lebanon in 2017 (Fig. 4a). Between 1990 and 2017, a total of 58, 31, and 106 countries or territories experienced a significant increase, remained stable, and experienced a significant decrease in leukemia ASIR, respectively. The greatest increase was found in Slovakia (EAPC = 1.80, 95% CI 1.59, 2.00), followed by Jamaica and Ecuador (Fig. 4b; Additional file 1: Table S1). The most pronounced decrease was found in Bahrain (EAPC = − 2.60, 95% CI − 3.04, − 2.16), followed by Iraq and Australia (Fig. 4b; Additional file 1: Table S1).

**Table 1:** The incidence and its trends of leukemia, by sex, age, type, and region, between 1990 and 2017

	1990		2017		1990-2017
	Case number ($\times 1000$)	ASIR (/10^5^)	Case number ($\times 1000$)	ASIR (/10^5^)	EAPC (95% CI)
Overall	354.5	7.42	518.5	6.76	− 0.43 (− 0.48, − 0.38)
Sex					
Male	191.3	8.48	295.4	8.09	− 0.26 (− 0.32, − 0.21)
Female	163.1	6.57	223.1	5.62	− 0.65 (− 0.70, − 0.60)
Age (years)^a^					
Under 5	54.4	8.44	52.9	7.78	− 0.12 (− 0.20, − 0.05)
5–14	50.6	4.51	45.5	3.51	− 1.18 (− 1.38, − 0.98)
15–49	102.4	3.74	123.1	3.15	− 0.89 (− 1.00, − 0.77)
50–69	80.0	11.64	149.7	11.36	− 0.24 (− 0.33, − 0.15)
70+	67.0	32.96	147.3	34.03	0.11 (0.08, 0.14)
Types					
ALL	49.1	0.89	64.2	0.85	− 0.08 (− 0.15, − 0.02)
CLL	38.0	0.99	90.6	1.15	0.46 (0.40, 0.52)
AML	63.8	1.35	119.6	1.54	0.56 (0.49, 0.62)
CML	31.8	0.75	34.2	0.43	− 2.40 (− 2.53, − 2.26)
Others	171.8	3.43	209.9	2.78	− 0.93 (− 1.02, − 0.84)
SDI region					
High	106.6	9.10	150.8	7.73	− 0.67 (− 0.74, − 0.60)
High-middle	80.4	7.59	123.7	8.68	0.56 (0.50, 0.63)
Middle	93.2	6.57	131.9	6.47	− 0.19 (− 0.34, − 0.04)
Low-middle	44.4	4.87	66.8	4.54	− 0.34 (− 0.40, − 0.28)
Low	27.9	4.68	42.6	4.14	− 0.55 (− 0.59, − 0.50)
GBD region					
Andean Latin America	2.1	6.00	3.5	5.97	0.12 (− 0.01, 0.26)
Australasia	3.0	13.38	4.2	9.62	− 1.65 (− 1.92, − 1.38)
Caribbean	2.1	6.72	2.8	5.82	− 0.59 (− 0.68, − 0.50)
Central Asia	3.5	5.17	4.0	4.74	− 0.20 (− 0.31, − 0.08)
Central Europe	9.1	6.71	11.1	6.37	0.02 (− 0.12, 0.17)
Central Latin America	9.1	6.39	14.5	5.89	− 0.32 (− 0.38, − 0.27)
Central sub-Saharan Africa	1.8	4.07	3.4	3.87	− 0.24 (− 0.27, − 0.20)
East Asia	102.3	8.49	147.7	10.54	0.83 (0.63, 1.02)
Eastern Europe	17.1	7.00	19.7	6.91	0.06 (− 0.08, 0.21)
Eastern sub-Saharan Africa	7.2	4.66	13.3	4.48	− 0.29 (− 0.38, − 0.20)
High-income Asia Pacific	11.3	6.22	17.8	5.27	− 0.47 (− 0.57, − 0.38)
High-income North America	27.4	8.17	39.6	7.17	− 0.61 (− 0.78, − 0.43)
North Africa and Middle East	19.3	7.08	32.3	6.37	− 0.24 (− 0.31, − 0.17)
Oceania	0.3	6.61	0.6	6.34	− 0.07 (− 0.11, − 0.03)
South Asia	40.7	4.24	62.0	3.99	− 0.30 (− 0.39, − 0.22)
Southeast Asia	24.2	6.26	38.9	6.37	0.07 (− 0.04, 0.19)
Southern Latin America	3.0	6.25	3.9	5.29	− 0.68 (− 0.75, − 0.61)
Southern sub-Saharan Africa	1.6	4.36	2.5	4.10	− 0.30 (− 0.69, 0.08)
Tropical Latin America	7.2	5.50	10.2	4.65	− 0.58 (− 0.67, − 0.49)
Western Europe	56.8	10.62	75.7	8.86	− 0.76 (− 0.83, − 0.69)
Western sub-Saharan Africa	5.3	3.44	10.8	3.40	− 0.14 (− 0.23, − 0.05)

*ASIR* age-standardized incidence rate, *EAPC* estimated average percentage change, *SDI* socio-demographic index, *ALL* acute lymphoblastic leukemia, *CLL* chronic lymphoblastic leukemia, *AML* acute myeloid leukemia, *CML* chronic myeloid leukemia

^a^The incidence rates for each age group were crude incidence rather than the age-standardized incidence

### ALL incidence trends at the global, regional, and national level

**Fig. 5:**
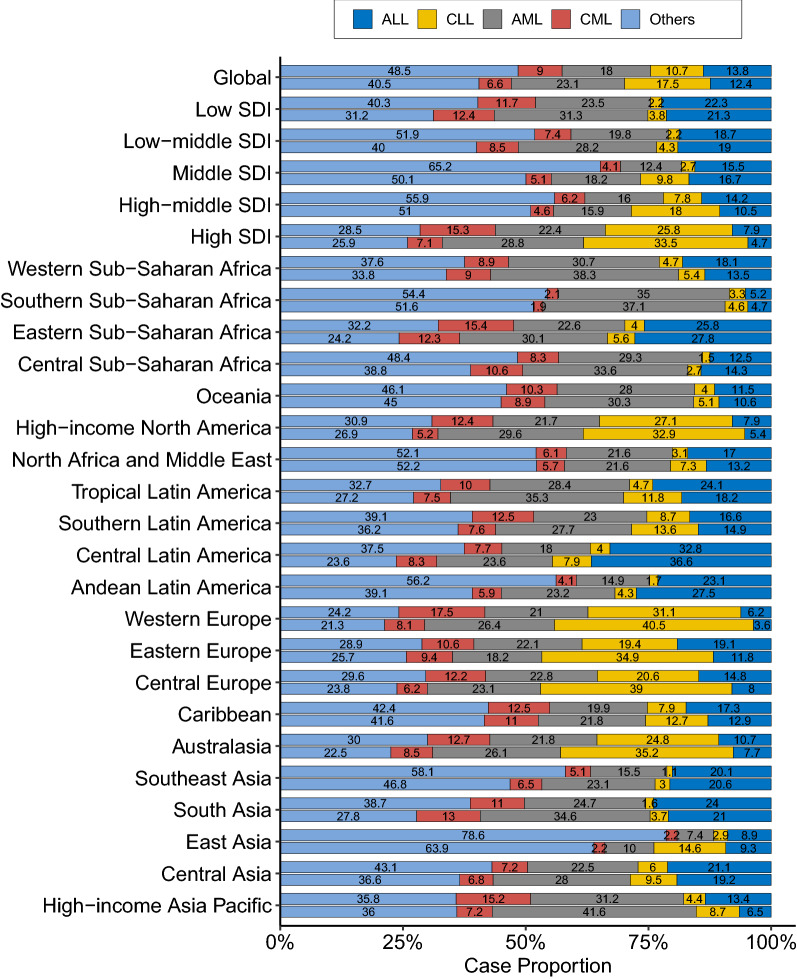
Proportions of leukemia cases by type and location in 1990 (top bar) and 2017 (bottom bar)

The global number of ALL cases increased from 49.1 thousand in 1990 to 64.2 thousand in 2017, whereas the ASIR experienced a mild decrease during this period (EAPC = − 0.08, 95% CI − 0.15, − 0.02; Fig. 1; Table 1). The proportion of ALL cases decreased from 13.8% to 12.4% between 1990 and 2017 (Fig. 5). ALL was more frequently diagnosed among children and young adults and in developing regions (Figs. 2 and 5). For example, in low-SDI regions, ALL cases accounted for > 20% of total leukemia cases in both 1990 and 2017. In Central Latin America, these proportions were > 30% (Fig. 5). Only five GBD regions experienced a significant increase in ASIR of ALL, with the greatest increase being observed in Andean Latin America, followed by East Asia and Southeast Asia (Fig. 3; Additional file 1: Table S1). As shown in Additional file 2: Figure S1, the ALL incidence was high in most developing countries. The highest incidence was found in Honduras (3.83/100,000), followed by Mexico and Dominica. Between 1990 and 2017, there were 55, 29, and 111 countries or territories that experienced a significant increase, remained stable, and experienced a significant decrease in ASIR of ALL, respectively (Additional file 1: Table S1, Additional file 2: Figure S1). The most pronounced increase was seen in El Salvador (EAPC = 5.20, 95% CI 4.34, 6.07; Additional file 1: Table S1, Additional file 2: Figure S1), followed by Guatemala and Ecuador. The greatest decrease was detected in Ghana (EAPC = − 5.18, 95% CI − 6.23, − 4.1), followed by Czech Republic and Guam (Additional file 1: Table S1, Additional file 2: Figure S1).

### CLL incidence trends at the global, regional, and national level

The proportion of CLL cases more than doubled between 1990 and 2017, increasing from 10.7 to 17.5% during this period (Table 1; Fig. 5). The ASIR of CLL increased by 0.46% per year from 1990 to 2017 (Table 1; Fig. 1). CLL was rarely diagnosed among young people, and the highest incidence was found in people aged ≥ 80 years (Fig. 2). Between 1990 and 2017, most regions experienced a significant increase in the ASIR of CLL. The greatest increase was detected in East Asia (EAPC = 6.26, 95% CI 5.64 6.88), followed by Southeast Asia and Eastern Europe (Fig. 3; Additional file 1: Table S1). As shown in Additional file 2: Figure S2, the CLL incidence was high in developed countries, with the highest incidence being observed in the UK (5.27/100,000), followed by Denmark and Slovakia in 2017. At the national level, more than 85% of all countries experienced an increase in the ASIR of CLL between 1990 and 2017 (Additional file 1: Table S1, Additional file 2: Figure S1). The greatest increase was found in Jamaica (EAPC = 7.02, 95% CI 6.18, 7.86), followed by China and South Korea. Only 12 countries or territories experienced a significant decrease in the ASIR of CLL. The most pronounced decrease was observed in the Netherlands (EAPC = − 2.98, 95% CI − 4.12, − 1.82), followed by Bahrain and Kyrgyzstan (Additional file 1: Table S1, Additional file 2: Figure S1).

### AML incidence trends at the global, regional, and national level

In 1990, AML accounted for 18.0% of total leukemia cases worldwide. This proportion increased to 23.1% in 2017 (Table 1; Fig. 5). The ASIR of AML increased from 1.35/100,000 to 1.54/100,000, with an EAPC of 0.56 (95% CI 0.49, 0.62) during this period (Table 1; Fig. 1). The incidence of AML showed a J-shaped age-pattern. In both 1990 and 2017, the lowest incidence was found among people aged 10–14 years (Fig. 2). The ASIR of AML increased in all five SDI regions and in a total of 13 GBD regions (Fig. 3; Additional file 1: Table S1). The greatest increase was found in Andean Latin America (EAPC = 1.68, 95% CI 1.55, 1.82), followed by East Asia and Southeast Asia. At the national level, the high incidence of AML was mostly observed in Europe. The highest incidence was found in the UK (4.05/100,000), followed by Brunei and Slovakia in 2017 (Additional file 2: Figure S3). A total of 127 countries or territories experienced a significant increase in the ASIR of AML between 1990 and 2017 (Additional file 1: Table S1; Additional file 2: Figure S3), with the highest increase being found in Ecuador (EAPC = 3.31, 95% CI 2.94, 3.69). Forty countries or territories experienced a significant decrease in the ASIR of AML during the same period. Most of these countries were located in the Middle East and Europe. The greatest decrease was found in Bahrain (EAPC = − 3.09, 95% CI − 3.57, − 2.60), followed by Qatar and Guyana (Additional file 1: Table S1; Additional file 2: Figure S3).

### CML incidence trends at the global, regional, and national level

CML is diagnosed relatively less often compared to other types of leukemia (Table 1; Fig. 5). Globally, the number of CML cases increased from 31.8 thousand in 1990 to 34.2 thousand in 2017. The ASIR of CML decreased from 0.75/100,000 to 0.43/100,000 during the same period (Table 1; Fig. 1). CML is rarely diagnosed among children and young adults. The incidence of CML decreased among all age groups between 1990 and 2017 (Fig. 2). The ASIR of CML decreased in all SDI and GBD regions, with the exception of Andean Latin America (EAPC = 0.46, 95% CI 0.32, 0.60) and Central sub-Saharan Africa (EAPC = 0.58, 95% CI 0.47, 0.69) (Fig. 3). At the national level, the highest incidence of CML was found in Ethiopia (1.98/100,000), followed by Brunei and Honduras in 2017 (Additional file 2: Figure S4). Only 34 countries or territories experienced a significant increase in the ASIR of CML between 1990 and 2017. The greatest increase was found in Jamaica (EAPC = 2.87, 95% CI 2.55, 3.20), followed by El Salvador and Ecuador (Additional file 1: Table S1; Additional file 2: Figure S4). Conversely, 141 countries or territories saw a decrease in the ASIR of CML. The most pronounced decrease was found in Germany (EAPC = − 5.23, 95% CI − 5.52, − 4.95), followed by the UK and Hungary (Additional file 1: Table S1; Additional file 2: Figure S4).

### Incidence trends of other leukemias at the global, regional, and national level

Worldwide, the number of cases for other leukemias increased from 171.8 thousand in 1990 to 209.9 thousand in 2017 (Table 1). The ASIR decreased by 0.93% per year during the study period (Table 1; Fig. 1). As shown in Fig. 2, the incidence of other leukemias showed a J-shaped age pattern. Approximately all SDI and GBD regions experienced a decreasing trend in the ASIR of other leukemias (Fig. 3). Exceptions were found in high-middle SDI regions and East Asia. In 2017, the highest ASIR was found in Syria (9.42/100,000), followed by Lebanon and China (Additional file 2: Figure S5). Between 1990 and 2017, 31 countries or territories experienced a significant increase in the ASIR of other leukemias. The greatest increase was observed in Slovakia (EAPC = 5.04, 95% CI 4.34, 5.75), followed by the Netherlands and Estonia (Additional file 1: Table S1; Additional file 2: Figure S5). In contrast, 139 countries or territories experienced a significant decrease in the ASIR of other leukemias. The most remarkable decrease was found in Equatorial Guinea (EAPC = − 3.5, 95% CI − 3.79, − 3.21), followed by Kyrgyzstan and Portugal (Additional file 1: Table S1; Additional file 2: Figure S5).

## Discussion

In this study, we comprehensively analyzed the secular leukemia incidence trends at the global, regional, and national level. We found that the overall ASIRs of leukemia, ALL, CML, and other leukemias significantly decreased over the last three decades. However, the ASIRs of AML and CLL increased at different magnitudes. The incidence of leukemia was heterogeneous among people in different age groups and from country to country. ALL was commonly diagnosed among children and young adults, whereas CLL, AML, and CML more frequently occurred among older people. From a geographical perspective, ALL was more commonly diagnosed in developing countries located in Latin America, the Middle East, and Southeast Asia. In contrast, CLL and AML were more commonly diagnosed in developed countries located in Europe and North America. Globally, CML was rarely diagnosed compared to the other three main types of leukemia. The changing incidence trends for the four major types varied worldwide. For example, the incidence of ALL decreased in most countries, whereas the incidence increased in several Asian and African countries. Conversely, the incidence of CLL increased in most countries. The decreasing trends were only seen in a few developed countries (e.g., the USA and Australia). The international variations in leukemia incidence and its changing trends not only mirror the effectiveness of previous prevention strategies but also indicate that updated and tailored prevention strategies have been established.

Although the risk factors contributing to leukemia have been extensively investigated, the current understanding of leukemia tumorigenesis remains limited. Previous studies have reported that exposure to ionizing radiation, herbicides and pesticides, and radon is associated with an increased risk of leukemia [21–23]. Moreover, the development of leukemia has been partly ascribed to genetic risk factors. For example, the initiating chromosomal aberrations comprise the deletion of chromosome 13q (del[13q]) in about 55% of CLL cases and the acquisition of chromosome 12 (trisomy 12) in 10–20% of CLL cases [24]. Epigenetic mutations of *DNMT3A*, *TET2*, and *ASXL1* have been identified in preleukaemic haemopoietic stem cells decades before the development of AML, suggesting that these are early founder events that precede leukemogenic transformation [25]. ALL in infants (< 12 months) is usually associated with *MLL* gene rearrangement. In contrast, non-*MLL*-rearranged B-ALL has a peak incidence between 2 and 5 years and a concordance rate of 10–15%, suggesting that, although initiation in utero is common, other “promotional” exposures are probably required for the later emergence of disease [4, 26]. CML is a clonal haemopoietic stem cell disorder characterized by a reciprocal translocation between the long arms of chromosomes 9 and 22 [27]. Despite the prior efforts, the 5-year survival rates of leukemia are not high, especially among high-risk patients and in developing countries [28–30]. Indeed, the discovery of the activity of interferon α in 1983, and the later addition of homoharringtonine (now known as omacetaxine and approved by the US Food and Drug Administration for the treatment of CML in 2012) and low-dose cytarabine resulted in a complete cytogenetic response rate (0% Ph-positive metaphases) of 20–30% and an improvement of the median survival to 6–7 years [31]. In standard medical and oncology textbooks, CLL is considered incurable [32]. In childhood ALL, the optimization of chemotherapy regimens over four decades, using mostly the same agents in improved combinations, has resulted in CR rates of 90–100% and potential cure rates of 80% or more. However, in adult ALL, the pediatric regimens have been modified over time to resemble the adult AML treatments, with a shorter duration of maintenance chemotherapy (long maintenance likely is responsible for an increased cure rate of 10–15%) and truncated classical postinduction consolidation in favor of early autologous and allogeneic stem cell transplantation [31]. These strategies have proven to be less successful.

In our study, we observed a significant decrease in leukemia incidence over the past three decades, mainly in developed countries. For example, the incidence of all types of leukemia decreased in Australia. Likewise, in Western Europe, only the incidence of AML showed an increasing trend. The decreases may have been at least partly driven by the following: (1) reducing the exposure to environmental risk factors, such as chemicals, particularly among children and pregnant women; (2) abstaining from high-risk parental behaviors, such as cigarette smoking; (3) increasing the intake of folate and vitamin supplementation during the preconception period or pregnancy; and (4) expanding the genetic screening for high-risk germline mutations. However, we also observed a significant increase in leukemia incidence in some developing countries. Such increases might be mainly explained by the continuous improvement of healthcare facilities and the quality of cancer surveillance systems in these countries. More cancer cases were diagnosed and recorded. On the other hand, the increases also suggest that leukemia is a hard-to-ignore public health concern in the relevant countries. More interventions are therefore needed. In addition, we also observed a significant decrease in the incidence of other leukemias, especially in developing regions. We assessed that this decrease could be ascribed to the development of diagnostic approaches that subsequently allowed for more leukemia cases to be precisely classified.

Surprisingly, we found that the incidence of both AML and CLL increased in most countries. The rise of AML incidence was partly due to an increasing prevalence of therapy for AML as more patients treated with cytotoxic chemotherapy are cured of their primary malignancy [33]. For CLL, previous studies reported a strong birth-cohort effect underlying this increasing trend and suggested that lifestyles and environmental factors may play a role in the development of CLL [34, 35]. Since AML and CLL accounted for nearly 40% of the total leukemia cases worldwide, the rising incidence indicates a further expansion of leukemia cases and the increase of the leukemia disease burden. The currently available toxicologic and observational epidemiological studies have provided strong evidentiary basis for the presence of casual associations (of small to moderate sizes) between several environmental exposures and leukemia. Awaiting a more complete evidentiary basis for decision-making, though ideal, will result in significant delays. However, educating clinicians and the public on primary prevention—actions that individuals can take to reduce their own family’s exposures to chemical risk factors for childhood leukemia and other disorders—is something that can occur now. Ultimately, regulatory actions based on the evolving science are needed to shift the burden from the individual to producers [36].

The limitations of our study should also be noted. First, all data were derived from mathematical models based on surveillance data rather than the surveillance data itself. Second, owing to the conventional limitations of cancer surveillance systems (e.g., changes between coding systems can lead to artificial differences in disease estimates), the disease burden of leukemia might be underestimated in some developing countries. Third, the diagnostic and classification criteria for leukemia types varied from country to country, which might introduce biases to learn the changing trends of certain types of leukemia.

## Conclusion

In sum, in the current study, we analyzed the incidence trends of leukemia types at the global, regional, and national level. We reported a significant decrease in leukemia incidence between 1990 and 2017. However, the incidence of both AML and CLL significantly increased in most countries during the study period, suggesting that these types of leukemia might become a major global public health concern. More importantly, the incidence of leukemia and its changing trends were highly heterogeneous across the world. Therefore, more prevention strategies tailored to each country are needed.

## Supplementary information

**Additional file 1.** Supplementary Table S1.

**Additional file 2.** Supplementary Figures S1–S5.

## Data Availability

The datasets supporting the conclusions of this article are included within the article.
