# Supplementary material for: Leukemia incidence trends at the global, regional, and national level between 1990 and 2017
Source: Exp Hematol Oncol. 2020 Jun 19;9:14. doi: 10.1186/s40164-020-00170-6 (PMC7304189; doi:10.1186/s40164-020-00170-6)
Supplement: Supplementary file 1 — Additional file 1. Supplementary Table S1. [file 40164_2020_170_MOESM1_ESM.pdf]

**Table S1. The changing trends of leukemia incidence, by sex, location, and type, between 1990 and 2017.**

| Sex | Location | Type | EAPC | 95% CI | |
|---|---|---|---|---|---|
| Male | Afghanistan | Acute lymphoid leukemia | 0.70 | 0.47 | 0.94 |
| Male | Afghanistan | Chronic lymphoid leukemia | 0.15 | 0.09 | 0.21 |
| Male | Afghanistan | Acute myeloid leukemia | 0.40 | 0.32 | 0.49 |
| Male | Afghanistan | Chronic myeloid leukemia | 0.26 | 0.04 | 0.47 |
| Male | Afghanistan | Leukemia | 0.50 | 0.30 | 0.69 |
| Male | Afghanistan | Other leukemia | 0.53 | 0.28 | 0.77 |
| Female | Afghanistan | Acute lymphoid leukemia | 0.93 | 0.71 | 1.16 |
| Female | Afghanistan | Chronic lymphoid leukemia | 0.33 | 0.24 | 0.42 |
| Female | Afghanistan | Acute myeloid leukemia | 0.33 | 0.20 | 0.47 |
| Female | Afghanistan | Chronic myeloid leukemia | -0.75 | -0.99 | -0.52 |
| Female | Afghanistan | Leukemia | 0.14 | -0.03 | 0.31 |
| Female | Afghanistan | Other leukemia | 0.11 | -0.09 | 0.30 |
| Both | Afghanistan | Acute lymphoid leukemia | 0.77 | 0.54 | 1.00 |
| Both | Afghanistan | Chronic lymphoid leukemia | 0.15 | 0.09 | 0.21 |
| Both | Afghanistan | Acute myeloid leukemia | 0.35 | 0.24 | 0.46 |
| Both | Afghanistan | Chronic myeloid leukemia | -0.41 | -0.65 | -0.17 |
| Both | Afghanistan | Leukemia | 0.28 | 0.09 | 0.46 |
| Both | Afghanistan | Other leukemia | 0.27 | 0.05 | 0.49 |
| Male | Albania | Acute lymphoid leukemia | 0.10 | -0.16 | 0.36 |
| Male | Albania | Chronic lymphoid leukemia | 1.32 | 1.18 | 1.46 |
| Male | Albania | Acute myeloid leukemia | 1.29 | 1.07 | 1.52 |
| Male | Albania | Chronic myeloid leukemia | -0.29 | -0.49 | -0.08 |
| Male | Albania | Leukemia | 0.60 | 0.47 | 0.73 |
| Male | Albania | Other leukemia | 0.11 | 0.00 | 0.22 |
| Female | Albania | Acute lymphoid leukemia | -0.75 | -0.92 | -0.59 |
| Female | Albania | Chronic lymphoid leukemia | 2.16 | 1.80 | 2.52 |
| Female | Albania | Acute myeloid leukemia | 0.43 | 0.26 | 0.60 |
| Female | Albania | Chronic myeloid leukemia | -1.77 | -2.07 | -1.48 |
| Female | Albania | Leukemia | 0.35 | 0.20 | 0.49 |
| Female | Albania | Other leukemia | 0.56 | 0.37 | 0.76 |
| Both | Albania | Acute lymphoid leukemia | -0.16 | -0.34 | 0.03 |
| Both | Albania | Chronic lymphoid leukemia | 1.63 | 1.44 | 1.82 |
| Both | Albania | Acute myeloid leukemia | 0.98 | 0.79 | 1.17 |
| Both | Albania | Chronic myeloid leukemia | -0.73 | -0.95 | -0.51 |
| Both | Albania | Leukemia | 0.54 | 0.41 | 0.67 |
| Both | Albania | Other leukemia | 0.35 | 0.22 | 0.49 |
| Male | Algeria | Acute lymphoid leukemia | -0.16 | -0.31 | 0.00 |
| Male | Algeria | Chronic lymphoid leukemia | 1.93 | 1.79 | 2.08 |
| Male | Algeria | Acute myeloid leukemia | 0.60 | 0.46 | 0.74 |
| Male | Algeria | Chronic myeloid leukemia | -0.25 | -0.39 | -0.11 |
| Male | Algeria | Leukemia | 0.11 | -0.01 | 0.23 |
| Male | Algeria | Other leukemia | -0.30 | -0.42 | -0.18 |
| Female | Algeria | Acute lymphoid leukemia | 0.56 | 0.32 | 0.80 |
| Female | Algeria | Chronic lymphoid leukemia | 2.49 | 2.33 | 2.65 |
| Female | Algeria | Acute myeloid leukemia | 0.39 | 0.24 | 0.54 |

| | | | | | |
|---|---|---|---|---|---|
| Female | Algeria | Chronic myeloid leukemia | -0.69 | -0.84 | -0.54 |
| Female | Algeria | Leukemia | 0.07 | -0.06 | 0.20 |
| Female | Algeria | Other leukemia | -0.28 | -0.41 | -0.15 |
| Both | Algeria | Acute lymphoid leukemia | 0.04 | -0.13 | 0.21 |
| Both | Algeria | Chronic lymphoid leukemia | 2.18 | 2.03 | 2.32 |
| Both | Algeria | Acute myeloid leukemia | 0.51 | 0.37 | 0.65 |
| Both | Algeria | Chronic myeloid leukemia | -0.45 | -0.58 | -0.31 |
| Both | Algeria | Leukemia | 0.10 | -0.02 | 0.22 |
| Both | Algeria | Other leukemia | -0.28 | -0.39 | -0.16 |
| Male | American Samoa | Acute lymphoid leukemia | -1.19 | -1.37 | -1.01 |
| Male | American Samoa | Chronic lymphoid leukemia | 0.24 | 0.16 | 0.33 |
| Male | American Samoa | Acute myeloid leukemia | 0.38 | 0.03 | 0.73 |
| Male | American Samoa | Chronic myeloid leukemia | -1.35 | -1.66 | -1.03 |
| Male | American Samoa | Leukemia | -0.08 | -0.20 | 0.04 |
| Male | American Samoa | Other leukemia | 0.14 | -0.06 | 0.34 |
| Female | American Samoa | Acute lymphoid leukemia | -2.28 | -2.77 | -1.80 |
| Female | American Samoa | Chronic lymphoid leukemia | 1.69 | 1.57 | 1.82 |
| Female | American Samoa | Acute myeloid leukemia | -1.70 | -1.92 | -1.48 |
| Female | American Samoa | Chronic myeloid leukemia | -2.15 | -2.51 | -1.80 |
| Female | American Samoa | Leukemia | -0.37 | -0.44 | -0.29 |
| Female | American Samoa | Other leukemia | 0.29 | -0.02 | 0.61 |
| Both | American Samoa | Acute lymphoid leukemia | -1.93 | -2.29 | -1.57 |
| Both | American Samoa | Chronic lymphoid leukemia | 1.54 | 1.41 | 1.66 |
| Both | American Samoa | Acute myeloid leukemia | -0.41 | -0.66 | -0.15 |
| Both | American Samoa | Chronic myeloid leukemia | -1.72 | -2.02 | -1.41 |
| Both | American Samoa | Leukemia | -0.26 | -0.31 | -0.20 |
| Both | American Samoa | Other leukemia | 0.15 | -0.03 | 0.33 |
| Male | Andean Latin America | Acute lymphoid leukemia | 1.24 | 1.08 | 1.40 |
| Male | Andean Latin America | Chronic lymphoid leukemia | 1.95 | 1.73 | 2.17 |
| Male | Andean Latin America | Acute myeloid leukemia | 1.70 | 1.56 | 1.84 |
| Male | Andean Latin America | Chronic myeloid leukemia | 0.62 | 0.47 | 0.77 |
| Male | Andean Latin America | Leukemia | 0.15 | 0.01 | 0.29 |
| Male | Andean Latin America | Other leukemia | -1.24 | -1.52 | -0.95 |
| Female | Andean Latin America | Acute lymphoid leukemia | 1.84 | 1.73 | 1.96 |
| Female | Andean Latin America | Chronic lymphoid leukemia | 2.66 | 2.38 | 2.95 |
| Female | Andean Latin America | Acute myeloid leukemia | 1.66 | 1.50 | 1.83 |
| Female | Andean Latin America | Chronic myeloid leukemia | 0.24 | 0.10 | 0.38 |
| Female | Andean Latin America | Leukemia | 0.09 | -0.05 | 0.23 |
| Female | Andean Latin America | Other leukemia | -1.50 | -1.81 | -1.20 |
| Both | Andean Latin America | Leukemia | 0.12 | -0.01 | 0.26 |
| Both | Andean Latin America | Acute lymphoid leukemia | 1.51 | 1.37 | 1.64 |
| Both | Andean Latin America | Chronic lymphoid leukemia | 2.24 | 2.02 | 2.47 |
| Both | Andean Latin America | Acute myeloid leukemia | 1.68 | 1.55 | 1.82 |
| Both | Andean Latin America | Chronic myeloid leukemia | 0.46 | 0.32 | 0.60 |
| Both | Andean Latin America | Other leukemia | -1.36 | -1.65 | -1.07 |
| Male | Andorra | Acute lymphoid leukemia | -1.50 | -1.58 | -1.43 |
| Male | Andorra | Chronic lymphoid leukemia | 0.44 | 0.16 | 0.73 |
| Male | Andorra | Acute myeloid leukemia | -0.55 | -0.62 | -0.47 |

| | | | | | |
|---|---|---|---|---|---|
| Male | Andorra | Chronic myeloid leukemia | -2.40 | -2.51 | -2.29 |
| Male | Andorra | Leukemia | -0.55 | -0.66 | -0.43 |
| Male | Andorra | Other leukemia | -1.41 | -1.47 | -1.36 |
| Female | Andorra | Acute lymphoid leukemia | -0.69 | -0.74 | -0.64 |
| Female | Andorra | Chronic lymphoid leukemia | 0.56 | 0.37 | 0.75 |
| Female | Andorra | Acute myeloid leukemia | -0.23 | -0.25 | -0.21 |
| Female | Andorra | Chronic myeloid leukemia | -3.09 | -3.39 | -2.79 |
| Female | Andorra | Leukemia | -0.43 | -0.52 | -0.34 |
| Female | Andorra | Other leukemia | -1.27 | -1.42 | -1.11 |
| Both | Andorra | Acute lymphoid leukemia | -1.24 | -1.31 | -1.17 |
| Both | Andorra | Chronic lymphoid leukemia | 0.41 | 0.17 | 0.65 |
| Both | Andorra | Acute myeloid leukemia | -0.48 | -0.53 | -0.43 |
| Both | Andorra | Chronic myeloid leukemia | -2.72 | -2.87 | -2.57 |
| Both | Andorra | Leukemia | -0.57 | -0.65 | -0.49 |
| Both | Andorra | Other leukemia | -1.45 | -1.52 | -1.38 |
| Male | Angola | Acute lymphoid leukemia | -0.27 | -0.56 | 0.02 |
| Male | Angola | Chronic lymphoid leukemia | 3.17 | 3.00 | 3.34 |
| Male | Angola | Acute myeloid leukemia | 0.01 | -0.07 | 0.09 |
| Male | Angola | Chronic myeloid leukemia | 0.95 | 0.82 | 1.09 |
| Male | Angola | Leukemia | -0.30 | -0.42 | -0.19 |
| Male | Angola | Other leukemia | -1.48 | -1.71 | -1.25 |
| Female | Angola | Acute lymphoid leukemia | 0.72 | 0.60 | 0.83 |
| Female | Angola | Chronic lymphoid leukemia | 0.99 | 0.92 | 1.07 |
| Female | Angola | Acute myeloid leukemia | 1.06 | 0.91 | 1.22 |
| Female | Angola | Chronic myeloid leukemia | -0.56 | -0.79 | -0.32 |
| Female | Angola | Leukemia | -0.94 | -1.12 | -0.76 |
| Female | Angola | Other leukemia | -2.10 | -2.37 | -1.83 |
| Both | Angola | Acute lymphoid leukemia | 0.11 | -0.09 | 0.30 |
| Both | Angola | Chronic lymphoid leukemia | 1.99 | 1.88 | 2.10 |
| Both | Angola | Acute myeloid leukemia | 0.05 | -0.01 | 0.12 |
| Both | Angola | Chronic myeloid leukemia | 0.09 | -0.09 | 0.28 |
| Both | Angola | Leukemia | -0.64 | -0.79 | -0.49 |
| Both | Angola | Other leukemia | -1.81 | -2.07 | -1.55 |
| Male | Antigua and Barbuda | Acute lymphoid leukemia | 0.61 | 0.50 | 0.72 |
| Male | Antigua and Barbuda | Chronic lymphoid leukemia | 0.70 | 0.63 | 0.77 |
| Male | Antigua and Barbuda | Acute myeloid leukemia | 1.01 | 0.89 | 1.12 |
| Male | Antigua and Barbuda | Chronic myeloid leukemia | -2.39 | -2.54 | -2.25 |
| Male | Antigua and Barbuda | Leukemia | -0.46 | -0.51 | -0.40 |
| Male | Antigua and Barbuda | Other leukemia | -0.91 | -1.02 | -0.79 |
| Female | Antigua and Barbuda | Acute lymphoid leukemia | 0.42 | 0.33 | 0.50 |
| Female | Antigua and Barbuda | Chronic lymphoid leukemia | 1.80 | 1.72 | 1.89 |
| Female | Antigua and Barbuda | Acute myeloid leukemia | 1.28 | 1.18 | 1.38 |
| Female | Antigua and Barbuda | Chronic myeloid leukemia | -2.51 | -2.71 | -2.30 |
| Female | Antigua and Barbuda | Leukemia | 0.19 | 0.08 | 0.29 |
| Female | Antigua and Barbuda | Other leukemia | 0.14 | 0.00 | 0.28 |
| Both | Antigua and Barbuda | Acute lymphoid leukemia | 0.59 | 0.50 | 0.68 |
| Both | Antigua and Barbuda | Chronic lymphoid leukemia | 1.13 | 1.09 | 1.17 |
| Both | Antigua and Barbuda | Acute myeloid leukemia | 1.16 | 1.07 | 1.26 |

| | | | | | |
|---|---|---|---|---|---|
| Both | Antigua and Barbuda | Chronic myeloid leukemia | -2.35 | -2.51 | -2.19 |
| Both | Antigua and Barbuda | Leukemia | -0.11 | -0.16 | -0.07 |
| Both | Antigua and Barbuda | Other leukemia | -0.33 | -0.40 | -0.26 |
| Male | Argentina | Acute lymphoid leukemia | -0.22 | -0.28 | -0.16 |
| Male | Argentina | Chronic lymphoid leukemia | -0.38 | -0.69 | -0.08 |
| Male | Argentina | Acute myeloid leukemia | 0.01 | -0.09 | 0.11 |
| Male | Argentina | Chronic myeloid leukemia | -3.56 | -3.96 | -3.15 |
| Male | Argentina | Leukemia | -0.86 | -0.93 | -0.78 |
| Male | Argentina | Other leukemia | -1.06 | -1.17 | -0.95 |
| Female | Argentina | Acute lymphoid leukemia | 0.08 | -0.08 | 0.25 |
| Female | Argentina | Chronic lymphoid leukemia | -0.01 | -0.27 | 0.26 |
| Female | Argentina | Acute myeloid leukemia | 0.18 | 0.01 | 0.34 |
| Female | Argentina | Chronic myeloid leukemia | -3.93 | -4.46 | -3.39 |
| Female | Argentina | Leukemia | -0.67 | -0.81 | -0.54 |
| Female | Argentina | Other leukemia | -1.05 | -1.14 | -0.96 |
| Both | Argentina | Acute lymphoid leukemia | -0.09 | -0.17 | -0.01 |
| Both | Argentina | Chronic lymphoid leukemia | -0.23 | -0.50 | 0.05 |
| Both | Argentina | Acute myeloid leukemia | 0.06 | -0.05 | 0.18 |
| Both | Argentina | Chronic myeloid leukemia | -3.72 | -4.18 | -3.27 |
| Both | Argentina | Leukemia | -0.80 | -0.89 | -0.71 |
| Both | Argentina | Other leukemia | -1.09 | -1.19 | -0.99 |
| Male | Armenia | Acute lymphoid leukemia | -1.42 | -1.61 | -1.23 |
| Male | Armenia | Chronic lymphoid leukemia | 1.86 | 1.64 | 2.07 |
| Male | Armenia | Acute myeloid leukemia | -0.81 | -0.95 | -0.66 |
| Male | Armenia | Chronic myeloid leukemia | -1.51 | -1.73 | -1.29 |
| Male | Armenia | Leukemia | -0.65 | -0.82 | -0.49 |
| Male | Armenia | Other leukemia | -0.79 | -1.01 | -0.57 |
| Female | Armenia | Acute lymphoid leukemia | -0.77 | -0.93 | -0.61 |
| Female | Armenia | Chronic lymphoid leukemia | 1.00 | 0.79 | 1.22 |
| Female | Armenia | Acute myeloid leukemia | -0.54 | -0.77 | -0.31 |
| Female | Armenia | Chronic myeloid leukemia | -1.78 | -1.92 | -1.64 |
| Female | Armenia | Leukemia | -0.71 | -0.87 | -0.54 |
| Female | Armenia | Other leukemia | -1.12 | -1.30 | -0.94 |
| Both | Armenia | Acute lymphoid leukemia | -1.22 | -1.33 | -1.11 |
| Both | Armenia | Chronic lymphoid leukemia | 1.46 | 1.28 | 1.64 |
| Both | Armenia | Acute myeloid leukemia | -0.68 | -0.78 | -0.58 |
| Both | Armenia | Chronic myeloid leukemia | -1.66 | -1.79 | -1.53 |
| Both | Armenia | Leukemia | -0.70 | -0.81 | -0.59 |
| Both | Armenia | Other leukemia | -0.97 | -1.12 | -0.81 |
| Male | Australasia | Acute lymphoid leukemia | -2.25 | -2.49 | -2.00 |
| Male | Australasia | Chronic lymphoid leukemia | -1.03 | -1.44 | -0.61 |
| Male | Australasia | Acute myeloid leukemia | -0.71 | -0.91 | -0.51 |
| Male | Australasia | Chronic myeloid leukemia | -3.23 | -3.53 | -2.93 |
| Male | Australasia | Leukemia | -1.78 | -2.07 | -1.49 |
| Male | Australasia | Other leukemia | -2.58 | -2.95 | -2.22 |
| Female | Australasia | Acute lymphoid leukemia | -2.69 | -2.88 | -2.49 |
| Female | Australasia | Chronic lymphoid leukemia | -0.61 | -0.87 | -0.34 |
| Female | Australasia | Acute myeloid leukemia | -0.90 | -1.23 | -0.57 |

| | | | | | |
|---|---|---|---|---|---|
| Female | Australasia | Chronic myeloid leukemia | -3.90 | -4.23 | -3.58 |
| Female | Australasia | Leukemia | -1.63 | -1.86 | -1.39 |
| Female | Australasia | Other leukemia | -2.38 | -2.63 | -2.13 |
| Both | Australasia | Leukemia | -1.65 | -1.92 | -1.38 |
| Both | Australasia | Acute lymphoid leukemia | -2.33 | -2.53 | -2.13 |
| Both | Australasia | Chronic lymphoid leukemia | -0.78 | -1.13 | -0.43 |
| Both | Australasia | Acute myeloid leukemia | -0.73 | -0.99 | -0.48 |
| Both | Australasia | Chronic myeloid leukemia | -3.35 | -3.66 | -3.03 |
| Both | Australasia | Other leukemia | -2.44 | -2.75 | -2.13 |
| Male | Australia | Acute lymphoid leukemia | -2.22 | -2.49 | -1.95 |
| Male | Australia | Chronic lymphoid leukemia | -1.23 | -1.69 | -0.77 |
| Male | Australia | Acute myeloid leukemia | -0.73 | -0.97 | -0.49 |
| Male | Australia | Chronic myeloid leukemia | -3.13 | -3.45 | -2.82 |
| Male | Australia | Leukemia | -1.92 | -2.24 | -1.59 |
| Male | Australia | Other leukemia | -2.81 | -3.20 | -2.43 |
| Female | Australia | Acute lymphoid leukemia | -2.81 | -3.05 | -2.57 |
| Female | Australia | Chronic lymphoid leukemia | -0.66 | -0.94 | -0.38 |
| Female | Australia | Acute myeloid leukemia | -1.06 | -1.40 | -0.72 |
| Female | Australia | Chronic myeloid leukemia | -3.96 | -4.32 | -3.60 |
| Female | Australia | Leukemia | -1.78 | -2.02 | -1.53 |
| Female | Australia | Other leukemia | -2.56 | -2.81 | -2.30 |
| Both | Australia | Acute lymphoid leukemia | -2.34 | -2.56 | -2.12 |
| Both | Australia | Chronic lymphoid leukemia | -0.93 | -1.32 | -0.53 |
| Both | Australia | Acute myeloid leukemia | -0.82 | -1.09 | -0.54 |
| Both | Australia | Chronic myeloid leukemia | -3.30 | -3.63 | -2.96 |
| Both | Australia | Leukemia | -1.79 | -2.08 | -1.50 |
| Both | Australia | Other leukemia | -2.65 | -2.97 | -2.33 |
| Male | Austria | Acute lymphoid leukemia | -1.44 | -1.61 | -1.26 |
| Male | Austria | Chronic lymphoid leukemia | 1.75 | 1.39 | 2.10 |
| Male | Austria | Acute myeloid leukemia | 2.59 | 2.43 | 2.76 |
| Male | Austria | Chronic myeloid leukemia | -0.82 | -1.03 | -0.61 |
| Male | Austria | Leukemia | 1.23 | 1.04 | 1.42 |
| Male | Austria | Other leukemia | 0.56 | -0.08 | 1.21 |
| Female | Austria | Acute lymphoid leukemia | -0.48 | -0.66 | -0.30 |
| Female | Austria | Chronic lymphoid leukemia | 2.00 | 1.77 | 2.23 |
| Female | Austria | Acute myeloid leukemia | 1.84 | 1.72 | 1.96 |
| Female | Austria | Chronic myeloid leukemia | -3.22 | -3.55 | -2.89 |
| Female | Austria | Leukemia | 0.62 | 0.51 | 0.73 |
| Female | Austria | Other leukemia | -1.04 | -1.49 | -0.60 |
| Both | Austria | Acute lymphoid leukemia | -1.01 | -1.14 | -0.88 |
| Both | Austria | Chronic lymphoid leukemia | 2.01 | 1.71 | 2.30 |
| Both | Austria | Acute myeloid leukemia | 2.26 | 2.13 | 2.38 |
| Both | Austria | Chronic myeloid leukemia | -1.82 | -2.03 | -1.61 |
| Both | Austria | Leukemia | 1.05 | 0.92 | 1.17 |
| Both | Austria | Other leukemia | -0.05 | -0.61 | 0.52 |
| Male | Azerbaijan | Acute lymphoid leukemia | 1.31 | 0.84 | 1.78 |
| Male | Azerbaijan | Chronic lymphoid leukemia | 1.80 | 1.52 | 2.07 |
| Male | Azerbaijan | Acute myeloid leukemia | 2.02 | 1.77 | 2.26 |

| | | | | | |
|---|---|---|---|---|---|
| Male | Azerbaijan | Chronic myeloid leukemia | -0.17 | -0.44 | 0.11 |
| Male | Azerbaijan | Leukemia | 1.05 | 0.82 | 1.29 |
| Male | Azerbaijan | Other leukemia | 0.86 | 0.61 | 1.11 |
| Female | Azerbaijan | Acute lymphoid leukemia | 0.66 | 0.27 | 1.04 |
| Female | Azerbaijan | Chronic lymphoid leukemia | 0.61 | 0.38 | 0.85 |
| Female | Azerbaijan | Acute myeloid leukemia | 1.46 | 1.21 | 1.70 |
| Female | Azerbaijan | Chronic myeloid leukemia | -0.52 | -0.95 | -0.10 |
| Female | Azerbaijan | Leukemia | 0.80 | 0.48 | 1.11 |
| Female | Azerbaijan | Other leukemia | 0.73 | 0.36 | 1.10 |
| Both | Azerbaijan | Acute lymphoid leukemia | 1.00 | 0.57 | 1.44 |
| Both | Azerbaijan | Chronic lymphoid leukemia | 1.13 | 0.90 | 1.36 |
| Both | Azerbaijan | Acute myeloid leukemia | 1.68 | 1.43 | 1.92 |
| Both | Azerbaijan | Chronic myeloid leukemia | -0.33 | -0.59 | -0.08 |
| Both | Azerbaijan | Leukemia | 0.95 | 0.68 | 1.22 |
| Both | Azerbaijan | Other leukemia | 0.84 | 0.54 | 1.13 |
| Male | Bahamas | Acute lymphoid leukemia | -0.38 | -0.59 | -0.18 |
| Male | Bahamas | Chronic lymphoid leukemia | 0.33 | 0.21 | 0.45 |
| Male | Bahamas | Acute myeloid leukemia | 0.10 | -0.07 | 0.27 |
| Male | Bahamas | Chronic myeloid leukemia | -0.44 | -0.59 | -0.29 |
| Male | Bahamas | Leukemia | -0.36 | -0.46 | -0.25 |
| Male | Bahamas | Other leukemia | -1.03 | -1.13 | -0.93 |
| Female | Bahamas | Acute lymphoid leukemia | -0.29 | -0.45 | -0.13 |
| Female | Bahamas | Chronic lymphoid leukemia | 1.18 | 1.02 | 1.34 |
| Female | Bahamas | Acute myeloid leukemia | -0.08 | -0.20 | 0.05 |
| Female | Bahamas | Chronic myeloid leukemia | -1.21 | -1.33 | -1.09 |
| Female | Bahamas | Leukemia | -0.27 | -0.40 | -0.15 |
| Female | Bahamas | Other leukemia | -1.52 | -1.77 | -1.27 |
| Both | Bahamas | Acute lymphoid leukemia | -0.35 | -0.49 | -0.20 |
| Both | Bahamas | Chronic lymphoid leukemia | 0.85 | 0.72 | 0.99 |
| Both | Bahamas | Acute myeloid leukemia | 0.03 | -0.11 | 0.17 |
| Both | Bahamas | Chronic myeloid leukemia | -0.61 | -0.73 | -0.50 |
| Both | Bahamas | Leukemia | -0.28 | -0.40 | -0.16 |
| Both | Bahamas | Other leukemia | -1.17 | -1.33 | -1.00 |
| Male | Bahrain | Acute lymphoid leukemia | -3.45 | -3.94 | -2.96 |
| Male | Bahrain | Chronic lymphoid leukemia | -2.17 | -2.69 | -1.64 |
| Male | Bahrain | Acute myeloid leukemia | -3.00 | -3.47 | -2.52 |
| Male | Bahrain | Chronic myeloid leukemia | -4.01 | -4.57 | -3.45 |
| Male | Bahrain | Leukemia | -2.48 | -2.92 | -2.03 |
| Male | Bahrain | Other leukemia | -1.76 | -2.34 | -1.18 |
| Female | Bahrain | Acute lymphoid leukemia | -2.76 | -3.34 | -2.18 |
| Female | Bahrain | Chronic lymphoid leukemia | -2.23 | -2.91 | -1.55 |
| Female | Bahrain | Acute myeloid leukemia | -3.34 | -3.89 | -2.79 |
| Female | Bahrain | Chronic myeloid leukemia | -5.18 | -5.85 | -4.52 |
| Female | Bahrain | Leukemia | -2.81 | -3.28 | -2.34 |
| Female | Bahrain | Other leukemia | -2.08 | -2.47 | -1.69 |
| Both | Bahrain | Acute lymphoid leukemia | -3.16 | -3.64 | -2.68 |
| Both | Bahrain | Chronic lymphoid leukemia | -2.27 | -2.83 | -1.70 |
| Both | Bahrain | Acute myeloid leukemia | -3.09 | -3.57 | -2.60 |

| | | | | | |
|---|---|---|---|---|---|
| Both | Bahrain | Chronic myeloid leukemia | -4.45 | -5.05 | -3.85 |
| Both | Bahrain | Leukemia | -2.60 | -3.04 | -2.16 |
| Both | Bahrain | Other leukemia | -1.90 | -2.39 | -1.41 |
| Male | Bangladesh | Acute lymphoid leukemia | -1.03 | -1.20 | -0.85 |
| Male | Bangladesh | Chronic lymphoid leukemia | -0.70 | -0.91 | -0.50 |
| Male | Bangladesh | Acute myeloid leukemia | -0.49 | -0.69 | -0.29 |
| Male | Bangladesh | Chronic myeloid leukemia | -2.15 | -2.28 | -2.02 |
| Male | Bangladesh | Leukemia | -1.38 | -1.53 | -1.24 |
| Male | Bangladesh | Other leukemia | -2.35 | -2.49 | -2.21 |
| Female | Bangladesh | Acute lymphoid leukemia | 0.00 | -0.28 | 0.29 |
| Female | Bangladesh | Chronic lymphoid leukemia | 1.07 | 0.92 | 1.23 |
| Female | Bangladesh | Acute myeloid leukemia | -0.84 | -0.95 | -0.73 |
| Female | Bangladesh | Chronic myeloid leukemia | -2.34 | -2.44 | -2.24 |
| Female | Bangladesh | Leukemia | -1.64 | -1.74 | -1.54 |
| Female | Bangladesh | Other leukemia | -2.92 | -2.99 | -2.85 |
| Both | Bangladesh | Acute lymphoid leukemia | -0.68 | -0.89 | -0.48 |
| Both | Bangladesh | Chronic lymphoid leukemia | -0.32 | -0.46 | -0.18 |
| Both | Bangladesh | Acute myeloid leukemia | -0.67 | -0.83 | -0.51 |
| Both | Bangladesh | Chronic myeloid leukemia | -2.23 | -2.33 | -2.13 |
| Both | Bangladesh | Leukemia | -1.51 | -1.63 | -1.39 |
| Both | Bangladesh | Other leukemia | -2.63 | -2.72 | -2.53 |
| Male | Barbados | Acute lymphoid leukemia | -0.42 | -0.56 | -0.28 |
| Male | Barbados | Chronic lymphoid leukemia | 0.64 | 0.44 | 0.84 |
| Male | Barbados | Acute myeloid leukemia | 0.27 | 0.17 | 0.36 |
| Male | Barbados | Chronic myeloid leukemia | -0.95 | -1.12 | -0.78 |
| Male | Barbados | Leukemia | -0.61 | -0.72 | -0.50 |
| Male | Barbados | Other leukemia | -1.30 | -1.44 | -1.16 |
| Female | Barbados | Acute lymphoid leukemia | 0.46 | 0.14 | 0.78 |
| Female | Barbados | Chronic lymphoid leukemia | 1.79 | 1.61 | 1.97 |
| Female | Barbados | Acute myeloid leukemia | 0.85 | 0.69 | 1.01 |
| Female | Barbados | Chronic myeloid leukemia | -0.93 | -1.02 | -0.83 |
| Female | Barbados | Leukemia | 0.11 | 0.01 | 0.22 |
| Female | Barbados | Other leukemia | -0.91 | -1.06 | -0.75 |
| Both | Barbados | Acute lymphoid leukemia | 0.02 | -0.13 | 0.17 |
| Both | Barbados | Chronic lymphoid leukemia | 1.15 | 1.02 | 1.29 |
| Both | Barbados | Acute myeloid leukemia | 0.61 | 0.50 | 0.71 |
| Both | Barbados | Chronic myeloid leukemia | -0.89 | -1.02 | -0.76 |
| Both | Barbados | Leukemia | -0.24 | -0.30 | -0.17 |
| Both | Barbados | Other leukemia | -1.09 | -1.21 | -0.97 |
| Male | Belarus | Acute lymphoid leukemia | -1.06 | -1.33 | -0.78 |
| Male | Belarus | Chronic lymphoid leukemia | 3.37 | 3.02 | 3.73 |
| Male | Belarus | Acute myeloid leukemia | 0.35 | 0.09 | 0.60 |
| Male | Belarus | Chronic myeloid leukemia | -0.02 | -0.43 | 0.39 |
| Male | Belarus | Leukemia | 0.09 | -0.14 | 0.31 |
| Male | Belarus | Other leukemia | -1.26 | -1.50 | -1.03 |
| Female | Belarus | Acute lymphoid leukemia | -1.23 | -1.60 | -0.86 |
| Female | Belarus | Chronic lymphoid leukemia | 2.94 | 2.65 | 3.24 |
| Female | Belarus | Acute myeloid leukemia | 0.05 | -0.23 | 0.34 |

| | | | | | |
|---|---|---|---|---|---|
| Female | Belarus | Chronic myeloid leukemia | -0.45 | -0.81 | -0.08 |
| Female | Belarus | Leukemia | -0.44 | -0.65 | -0.23 |
| Female | Belarus | Other leukemia | -2.11 | -2.40 | -1.82 |
| Both | Belarus | Acute lymphoid leukemia | -1.20 | -1.48 | -0.92 |
| Both | Belarus | Chronic lymphoid leukemia | 3.23 | 2.90 | 3.55 |
| Both | Belarus | Acute myeloid leukemia | 0.22 | -0.03 | 0.48 |
| Both | Belarus | Chronic myeloid leukemia | -0.22 | -0.61 | 0.17 |
| Both | Belarus | Leukemia | -0.19 | -0.39 | 0.02 |
| Both | Belarus | Other leukemia | -1.74 | -1.99 | -1.48 |
| Male | Belgium | Acute lymphoid leukemia | -2.16 | -2.50 | -1.82 |
| Male | Belgium | Chronic lymphoid leukemia | 0.27 | 0.01 | 0.52 |
| Male | Belgium | Acute myeloid leukemia | 0.54 | 0.35 | 0.73 |
| Male | Belgium | Chronic myeloid leukemia | -2.59 | -2.94 | -2.24 |
| Male | Belgium | Leukemia | -0.55 | -0.78 | -0.32 |
| Male | Belgium | Other leukemia | -0.79 | -1.47 | -0.10 |
| Female | Belgium | Acute lymphoid leukemia | -1.93 | -2.10 | -1.75 |
| Female | Belgium | Chronic lymphoid leukemia | 0.28 | 0.04 | 0.53 |
| Female | Belgium | Acute myeloid leukemia | 0.88 | 0.65 | 1.11 |
| Female | Belgium | Chronic myeloid leukemia | -3.60 | -3.87 | -3.33 |
| Female | Belgium | Leukemia | -0.52 | -0.70 | -0.34 |
| Female | Belgium | Other leukemia | -0.95 | -1.54 | -0.36 |
| Both | Belgium | Acute lymphoid leukemia | -2.04 | -2.28 | -1.80 |
| Both | Belgium | Chronic lymphoid leukemia | 0.39 | 0.16 | 0.61 |
| Both | Belgium | Acute myeloid leukemia | 0.71 | 0.52 | 0.90 |
| Both | Belgium | Chronic myeloid leukemia | -2.90 | -3.18 | -2.61 |
| Both | Belgium | Leukemia | -0.47 | -0.68 | -0.26 |
| Both | Belgium | Other leukemia | -0.76 | -1.42 | -0.10 |
| Male | Belize | Acute lymphoid leukemia | -0.01 | -0.21 | 0.19 |
| Male | Belize | Chronic lymphoid leukemia | 2.35 | 2.07 | 2.64 |
| Male | Belize | Acute myeloid leukemia | 1.29 | 1.02 | 1.56 |
| Male | Belize | Chronic myeloid leukemia | -0.01 | -0.45 | 0.43 |
| Male | Belize | Leukemia | -0.09 | -0.37 | 0.19 |
| Male | Belize | Other leukemia | -0.52 | -0.82 | -0.22 |
| Female | Belize | Acute lymphoid leukemia | -0.07 | -0.35 | 0.22 |
| Female | Belize | Chronic lymphoid leukemia | 2.29 | 1.96 | 2.63 |
| Female | Belize | Acute myeloid leukemia | 0.83 | 0.64 | 1.03 |
| Female | Belize | Chronic myeloid leukemia | -0.28 | -0.66 | 0.11 |
| Female | Belize | Leukemia | -0.47 | -0.69 | -0.25 |
| Female | Belize | Other leukemia | -0.81 | -1.03 | -0.59 |
| Both | Belize | Acute lymphoid leukemia | -0.04 | -0.23 | 0.15 |
| Both | Belize | Chronic lymphoid leukemia | 2.42 | 2.15 | 2.69 |
| Both | Belize | Acute myeloid leukemia | 1.05 | 0.83 | 1.27 |
| Both | Belize | Chronic myeloid leukemia | -0.11 | -0.51 | 0.30 |
| Both | Belize | Leukemia | -0.25 | -0.49 | -0.01 |
| Both | Belize | Other leukemia | -0.65 | -0.90 | -0.39 |
| Male | Benin | Acute lymphoid leukemia | -1.54 | -1.75 | -1.32 |
| Male | Benin | Chronic lymphoid leukemia | 1.27 | 1.22 | 1.31 |
| Male | Benin | Acute myeloid leukemia | 0.45 | 0.31 | 0.60 |

| | | | | | |
|---|---|---|---|---|---|
| Male | Benin | Chronic myeloid leukemia | 0.13 | 0.06 | 0.21 |
| Male | Benin | Leukemia | 0.15 | 0.08 | 0.21 |
| Male | Benin | Other leukemia | 0.33 | 0.24 | 0.42 |
| Female | Benin | Acute lymphoid leukemia | 0.53 | 0.44 | 0.62 |
| Female | Benin | Chronic lymphoid leukemia | 0.39 | 0.33 | 0.44 |
| Female | Benin | Acute myeloid leukemia | 0.58 | 0.51 | 0.65 |
| Female | Benin | Chronic myeloid leukemia | -0.53 | -0.63 | -0.43 |
| Female | Benin | Leukemia | -0.08 | -0.14 | -0.02 |
| Female | Benin | Other leukemia | -0.68 | -0.75 | -0.62 |
| Both | Benin | Acute lymphoid leukemia | -0.86 | -1.04 | -0.68 |
| Both | Benin | Chronic lymphoid leukemia | 0.73 | 0.67 | 0.78 |
| Both | Benin | Acute myeloid leukemia | 0.54 | 0.44 | 0.64 |
| Both | Benin | Chronic myeloid leukemia | -0.31 | -0.38 | -0.24 |
| Both | Benin | Leukemia | 0.04 | -0.02 | 0.10 |
| Both | Benin | Other leukemia | -0.11 | -0.17 | -0.05 |
| Male | Bermuda | Acute lymphoid leukemia | -1.95 | -2.42 | -1.47 |
| Male | Bermuda | Chronic lymphoid leukemia | 0.69 | 0.47 | 0.91 |
| Male | Bermuda | Acute myeloid leukemia | -0.53 | -0.77 | -0.29 |
| Male | Bermuda | Chronic myeloid leukemia | -1.49 | -1.79 | -1.18 |
| Male | Bermuda | Leukemia | -0.81 | -1.04 | -0.59 |
| Male | Bermuda | Other leukemia | -1.48 | -1.69 | -1.26 |
| Female | Bermuda | Acute lymphoid leukemia | -1.33 | -1.46 | -1.19 |
| Female | Bermuda | Chronic lymphoid leukemia | -0.40 | -0.71 | -0.09 |
| Female | Bermuda | Acute myeloid leukemia | -1.95 | -2.01 | -1.88 |
| Female | Bermuda | Chronic myeloid leukemia | -4.32 | -4.66 | -3.98 |
| Female | Bermuda | Leukemia | -2.30 | -2.40 | -2.20 |
| Female | Bermuda | Other leukemia | -2.78 | -2.97 | -2.60 |
| Both | Bermuda | Acute lymphoid leukemia | -1.69 | -2.00 | -1.37 |
| Both | Bermuda | Chronic lymphoid leukemia | 0.77 | 0.57 | 0.96 |
| Both | Bermuda | Acute myeloid leukemia | -1.18 | -1.31 | -1.05 |
| Both | Bermuda | Chronic myeloid leukemia | -1.61 | -1.88 | -1.34 |
| Both | Bermuda | Leukemia | -1.28 | -1.42 | -1.14 |
| Both | Bermuda | Other leukemia | -1.94 | -2.08 | -1.80 |
| Male | Bhutan | Acute lymphoid leukemia | -1.09 | -1.28 | -0.90 |
| Male | Bhutan | Chronic lymphoid leukemia | 1.48 | 1.35 | 1.60 |
| Male | Bhutan | Acute myeloid leukemia | 0.71 | 0.49 | 0.93 |
| Male | Bhutan | Chronic myeloid leukemia | -0.77 | -0.83 | -0.70 |
| Male | Bhutan | Leukemia | -0.46 | -0.59 | -0.33 |
| Male | Bhutan | Other leukemia | -1.69 | -1.80 | -1.58 |
| Female | Bhutan | Acute lymphoid leukemia | 0.24 | 0.04 | 0.44 |
| Female | Bhutan | Chronic lymphoid leukemia | 0.46 | 0.29 | 0.63 |
| Female | Bhutan | Acute myeloid leukemia | -0.31 | -0.42 | -0.20 |
| Female | Bhutan | Chronic myeloid leukemia | -2.75 | -2.87 | -2.64 |
| Female | Bhutan | Leukemia | -1.51 | -1.61 | -1.41 |
| Female | Bhutan | Other leukemia | -3.12 | -3.28 | -2.95 |
| Both | Bhutan | Acute lymphoid leukemia | -0.62 | -0.81 | -0.44 |
| Both | Bhutan | Chronic lymphoid leukemia | 1.24 | 1.15 | 1.32 |
| Both | Bhutan | Acute myeloid leukemia | 0.31 | 0.13 | 0.48 |

| | | | | | |
|---|---|---|---|---|---|
| Both | Bhutan | Chronic myeloid leukemia | -1.85 | -1.90 | -1.80 |
| Both | Bhutan | Leukemia | -0.90 | -1.01 | -0.79 |
| Both | Bhutan | Other leukemia | -2.34 | -2.47 | -2.22 |
| Male | Bolivia | Acute lymphoid leukemia | 0.27 | 0.01 | 0.52 |
| Male | Bolivia | Chronic lymphoid leukemia | 1.85 | 1.62 | 2.08 |
| Male | Bolivia | Acute myeloid leukemia | 1.62 | 1.25 | 2.00 |
| Male | Bolivia | Chronic myeloid leukemia | -0.25 | -0.35 | -0.15 |
| Male | Bolivia | Leukemia | -0.33 | -0.49 | -0.17 |
| Male | Bolivia | Other leukemia | -1.50 | -1.60 | -1.41 |
| Female | Bolivia | Acute lymphoid leukemia | 1.08 | 0.60 | 1.57 |
| Female | Bolivia | Chronic lymphoid leukemia | 1.22 | 1.13 | 1.31 |
| Female | Bolivia | Acute myeloid leukemia | 0.91 | 0.55 | 1.28 |
| Female | Bolivia | Chronic myeloid leukemia | -1.14 | -1.32 | -0.95 |
| Female | Bolivia | Leukemia | -0.74 | -0.98 | -0.50 |
| Female | Bolivia | Other leukemia | -2.11 | -2.30 | -1.92 |
| Both | Bolivia | Acute lymphoid leukemia | 0.60 | 0.25 | 0.95 |
| Both | Bolivia | Chronic lymphoid leukemia | 1.60 | 1.44 | 1.77 |
| Both | Bolivia | Acute myeloid leukemia | 1.27 | 0.91 | 1.64 |
| Both | Bolivia | Chronic myeloid leukemia | -0.72 | -0.85 | -0.58 |
| Both | Bolivia | Leukemia | -0.54 | -0.74 | -0.34 |
| Both | Bolivia | Other leukemia | -1.81 | -1.95 | -1.67 |
| Male | Bosnia and Herzegovina | Acute lymphoid leukemia | -0.58 | -0.75 | -0.40 |
| Male | Bosnia and Herzegovina | Chronic lymphoid leukemia | 1.99 | 1.75 | 2.24 |
| Male | Bosnia and Herzegovina | Acute myeloid leukemia | 0.45 | 0.31 | 0.58 |
| Male | Bosnia and Herzegovina | Chronic myeloid leukemia | -0.82 | -1.06 | -0.57 |
| Male | Bosnia and Herzegovina | Leukemia | 0.51 | 0.40 | 0.63 |
| Male | Bosnia and Herzegovina | Other leukemia | 0.11 | -0.07 | 0.28 |
| Female | Bosnia and Herzegovina | Acute lymphoid leukemia | -0.29 | -0.42 | -0.16 |
| Female | Bosnia and Herzegovina | Chronic lymphoid leukemia | 2.73 | 2.55 | 2.90 |
| Female | Bosnia and Herzegovina | Acute myeloid leukemia | 0.56 | 0.46 | 0.65 |
| Female | Bosnia and Herzegovina | Chronic myeloid leukemia | -1.44 | -1.74 | -1.15 |
| Female | Bosnia and Herzegovina | Leukemia | 0.62 | 0.50 | 0.75 |
| Female | Bosnia and Herzegovina | Other leukemia | 0.63 | 0.48 | 0.79 |
| Both | Bosnia and Herzegovina | Acute lymphoid leukemia | -0.46 | -0.62 | -0.31 |
| Both | Bosnia and Herzegovina | Chronic lymphoid leukemia | 2.33 | 2.12 | 2.54 |
| Both | Bosnia and Herzegovina | Acute myeloid leukemia | 0.49 | 0.39 | 0.59 |
| Both | Bosnia and Herzegovina | Chronic myeloid leukemia | -1.14 | -1.41 | -0.86 |
| Both | Bosnia and Herzegovina | Leukemia | 0.57 | 0.46 | 0.67 |
| Both | Bosnia and Herzegovina | Other leukemia | 0.35 | 0.19 | 0.50 |
| Male | Botswana | Acute lymphoid leukemia | -0.58 | -0.70 | -0.47 |
| Male | Botswana | Chronic lymphoid leukemia | 1.14 | 0.96 | 1.33 |
| Male | Botswana | Acute myeloid leukemia | -0.45 | -0.65 | -0.25 |
| Male | Botswana | Chronic myeloid leukemia | -0.91 | -1.06 | -0.76 |
| Male | Botswana | Leukemia | -0.62 | -0.73 | -0.51 |
| Male | Botswana | Other leukemia | -1.23 | -1.34 | -1.13 |
| Female | Botswana | Acute lymphoid leukemia | 0.73 | 0.37 | 1.09 |
| Female | Botswana | Chronic lymphoid leukemia | 2.74 | 2.15 | 3.34 |
| Female | Botswana | Acute myeloid leukemia | 1.10 | 0.57 | 1.63 |

| | | | | | |
|---|---|---|---|---|---|
| Female | Botswana | Chronic myeloid leukemia | 0.84 | 0.26 | 1.41 |
| Female | Botswana | Leukemia | 0.16 | -0.22 | 0.54 |
| Female | Botswana | Other leukemia | -0.16 | -0.53 | 0.22 |
| Both | Botswana | Acute lymphoid leukemia | 0.19 | -0.04 | 0.42 |
| Both | Botswana | Chronic lymphoid leukemia | 1.46 | 1.12 | 1.79 |
| Both | Botswana | Acute myeloid leukemia | -0.22 | -0.38 | -0.05 |
| Both | Botswana | Chronic myeloid leukemia | 0.08 | -0.30 | 0.45 |
| Both | Botswana | Leukemia | -0.27 | -0.48 | -0.06 |
| Both | Botswana | Other leukemia | -0.50 | -0.78 | -0.22 |
| Male | Brazil | Acute lymphoid leukemia | -0.28 | -0.48 | -0.09 |
| Male | Brazil | Chronic lymphoid leukemia | 1.35 | 1.22 | 1.47 |
| Male | Brazil | Acute myeloid leukemia | 0.04 | -0.05 | 0.12 |
| Male | Brazil | Chronic myeloid leukemia | -2.61 | -3.02 | -2.19 |
| Male | Brazil | Leukemia | -0.46 | -0.54 | -0.38 |
| Male | Brazil | Other leukemia | -0.96 | -1.04 | -0.87 |
| Female | Brazil | Acute lymphoid leukemia | -0.61 | -0.82 | -0.40 |
| Female | Brazil | Chronic lymphoid leukemia | 0.90 | 0.74 | 1.06 |
| Female | Brazil | Acute myeloid leukemia | 0.15 | 0.03 | 0.27 |
| Female | Brazil | Chronic myeloid leukemia | -3.52 | -3.95 | -3.08 |
| Female | Brazil | Leukemia | -0.71 | -0.81 | -0.62 |
| Female | Brazil | Other leukemia | -1.32 | -1.39 | -1.26 |
| Both | Brazil | Acute lymphoid leukemia | -0.42 | -0.61 | -0.23 |
| Both | Brazil | Chronic lymphoid leukemia | 1.13 | 1.01 | 1.25 |
| Both | Brazil | Acute myeloid leukemia | 0.07 | -0.02 | 0.17 |
| Both | Brazil | Chronic myeloid leukemia | -3.02 | -3.43 | -2.60 |
| Both | Brazil | Leukemia | -0.59 | -0.68 | -0.51 |
| Both | Brazil | Other leukemia | -1.14 | -1.21 | -1.07 |
| Male | Brunei | Acute lymphoid leukemia | 1.67 | 1.38 | 1.96 |
| Male | Brunei | Chronic lymphoid leukemia | 4.68 | 4.42 | 4.95 |
| Male | Brunei | Acute myeloid leukemia | 2.64 | 2.42 | 2.86 |
| Male | Brunei | Chronic myeloid leukemia | 0.79 | 0.57 | 1.02 |
| Male | Brunei | Leukemia | 1.43 | 1.20 | 1.66 |
| Male | Brunei | Other leukemia | 0.15 | -0.12 | 0.41 |
| Female | Brunei | Acute lymphoid leukemia | 1.01 | 0.76 | 1.25 |
| Female | Brunei | Chronic lymphoid leukemia | 3.02 | 2.80 | 3.25 |
| Female | Brunei | Acute myeloid leukemia | 1.39 | 1.09 | 1.69 |
| Female | Brunei | Chronic myeloid leukemia | -0.04 | -0.35 | 0.26 |
| Female | Brunei | Leukemia | 0.70 | 0.42 | 0.97 |
| Female | Brunei | Other leukemia | -1.81 | -2.08 | -1.53 |
| Both | Brunei | Acute lymphoid leukemia | 1.26 | 1.01 | 1.51 |
| Both | Brunei | Chronic lymphoid leukemia | 3.78 | 3.54 | 4.02 |
| Both | Brunei | Acute myeloid leukemia | 1.92 | 1.66 | 2.18 |
| Both | Brunei | Chronic myeloid leukemia | 0.43 | 0.16 | 0.69 |
| Both | Brunei | Leukemia | 1.06 | 0.81 | 1.31 |
| Both | Brunei | Other leukemia | -0.67 | -0.94 | -0.39 |
| Male | Bulgaria | Acute lymphoid leukemia | -1.29 | -1.73 | -0.86 |
| Male | Bulgaria | Chronic lymphoid leukemia | 3.64 | 3.38 | 3.90 |
| Male | Bulgaria | Acute myeloid leukemia | 0.53 | 0.24 | 0.82 |

| | | | | | |
|---|---|---|---|---|---|
| Male | Bulgaria | Chronic myeloid leukemia | -1.90 | -2.68 | -1.12 |
| Male | Bulgaria | Leukemia | 0.84 | 0.65 | 1.04 |
| Male | Bulgaria | Other leukemia | 2.23 | 1.96 | 2.51 |
| Female | Bulgaria | Acute lymphoid leukemia | -2.01 | -2.54 | -1.48 |
| Female | Bulgaria | Chronic lymphoid leukemia | 2.51 | 2.12 | 2.89 |
| Female | Bulgaria | Acute myeloid leukemia | 1.46 | 0.98 | 1.94 |
| Female | Bulgaria | Chronic myeloid leukemia | -2.35 | -3.18 | -1.52 |
| Female | Bulgaria | Leukemia | 1.52 | 1.12 | 1.93 |
| Female | Bulgaria | Other leukemia | 2.60 | 2.13 | 3.07 |
| Both | Bulgaria | Acute lymphoid leukemia | -1.48 | -1.92 | -1.04 |
| Both | Bulgaria | Chronic lymphoid leukemia | 3.11 | 2.81 | 3.42 |
| Both | Bulgaria | Acute myeloid leukemia | 0.95 | 0.58 | 1.32 |
| Both | Bulgaria | Chronic myeloid leukemia | -2.07 | -2.84 | -1.29 |
| Both | Bulgaria | Leukemia | 1.08 | 0.81 | 1.36 |
| Both | Bulgaria | Other leukemia | 2.36 | 2.04 | 2.68 |
| Male | Burkina Faso | Acute lymphoid leukemia | -1.73 | -2.08 | -1.38 |
| Male | Burkina Faso | Chronic lymphoid leukemia | 0.53 | 0.35 | 0.71 |
| Male | Burkina Faso | Acute myeloid leukemia | 0.02 | -0.25 | 0.30 |
| Male | Burkina Faso | Chronic myeloid leukemia | 0.45 | 0.34 | 0.55 |
| Male | Burkina Faso | Leukemia | 0.07 | -0.12 | 0.27 |
| Male | Burkina Faso | Other leukemia | 0.56 | 0.43 | 0.69 |
| Female | Burkina Faso | Acute lymphoid leukemia | -0.24 | -0.44 | -0.05 |
| Female | Burkina Faso | Chronic lymphoid leukemia | -0.10 | -0.21 | 0.00 |
| Female | Burkina Faso | Acute myeloid leukemia | -0.03 | -0.18 | 0.12 |
| Female | Burkina Faso | Chronic myeloid leukemia | -1.22 | -1.36 | -1.09 |
| Female | Burkina Faso | Leukemia | -0.64 | -0.80 | -0.49 |
| Female | Burkina Faso | Other leukemia | -1.07 | -1.24 | -0.90 |
| Both | Burkina Faso | Acute lymphoid leukemia | -1.25 | -1.54 | -0.96 |
| Both | Burkina Faso | Chronic lymphoid leukemia | 0.14 | 0.00 | 0.27 |
| Both | Burkina Faso | Acute myeloid leukemia | 0.03 | -0.19 | 0.26 |
| Both | Burkina Faso | Chronic myeloid leukemia | -0.49 | -0.60 | -0.39 |
| Both | Burkina Faso | Leukemia | -0.27 | -0.45 | -0.10 |
| Both | Burkina Faso | Other leukemia | -0.21 | -0.36 | -0.07 |
| Male | Burundi | Acute lymphoid leukemia | -0.94 | -1.12 | -0.76 |
| Male | Burundi | Chronic lymphoid leukemia | 1.79 | 1.63 | 1.96 |
| Male | Burundi | Acute myeloid leukemia | 0.08 | 0.03 | 0.14 |
| Male | Burundi | Chronic myeloid leukemia | -1.00 | -1.15 | -0.85 |
| Male | Burundi | Leukemia | -0.59 | -0.67 | -0.50 |
| Male | Burundi | Other leukemia | -1.52 | -1.61 | -1.43 |
| Female | Burundi | Acute lymphoid leukemia | 0.53 | 0.37 | 0.69 |
| Female | Burundi | Chronic lymphoid leukemia | 0.40 | 0.34 | 0.47 |
| Female | Burundi | Acute myeloid leukemia | 0.28 | 0.21 | 0.35 |
| Female | Burundi | Chronic myeloid leukemia | -1.18 | -1.30 | -1.06 |
| Female | Burundi | Leukemia | -0.67 | -0.74 | -0.59 |
| Female | Burundi | Other leukemia | -1.41 | -1.55 | -1.28 |
| Both | Burundi | Acute lymphoid leukemia | -0.33 | -0.48 | -0.18 |
| Both | Burundi | Chronic lymphoid leukemia | 0.80 | 0.73 | 0.86 |
| Both | Burundi | Acute myeloid leukemia | 0.35 | 0.29 | 0.41 |

| | | | | | |
|---|---|---|---|---|---|
| Both | Burundi | Chronic myeloid leukemia | -1.18 | -1.29 | -1.08 |
| Both | Burundi | Leukemia | -0.57 | -0.64 | -0.51 |
| Both | Burundi | Other leukemia | -1.46 | -1.56 | -1.36 |
| Male | Cambodia | Acute lymphoid leukemia | 1.45 | 1.29 | 1.61 |
| Male | Cambodia | Chronic lymphoid leukemia | 2.41 | 2.25 | 2.58 |
| Male | Cambodia | Acute myeloid leukemia | 1.53 | 1.45 | 1.61 |
| Male | Cambodia | Chronic myeloid leukemia | -0.16 | -0.23 | -0.09 |
| Male | Cambodia | Leukemia | 0.07 | 0.05 | 0.10 |
| Male | Cambodia | Other leukemia | -0.65 | -0.67 | -0.63 |
| Female | Cambodia | Acute lymphoid leukemia | 1.06 | 0.92 | 1.21 |
| Female | Cambodia | Chronic lymphoid leukemia | 1.55 | 1.45 | 1.64 |
| Female | Cambodia | Acute myeloid leukemia | 0.69 | 0.63 | 0.75 |
| Female | Cambodia | Chronic myeloid leukemia | -1.12 | -1.25 | -0.99 |
| Female | Cambodia | Leukemia | -0.92 | -0.98 | -0.86 |
| Female | Cambodia | Other leukemia | -1.59 | -1.68 | -1.50 |
| Both | Cambodia | Acute lymphoid leukemia | 1.30 | 1.15 | 1.44 |
| Both | Cambodia | Chronic lymphoid leukemia | 1.94 | 1.83 | 2.06 |
| Both | Cambodia | Acute myeloid leukemia | 1.01 | 0.94 | 1.07 |
| Both | Cambodia | Chronic myeloid leukemia | -0.73 | -0.79 | -0.67 |
| Both | Cambodia | Leukemia | -0.49 | -0.52 | -0.45 |
| Both | Cambodia | Other leukemia | -1.20 | -1.26 | -1.15 |
| Male | Cameroon | Acute lymphoid leukemia | -1.42 | -1.62 | -1.21 |
| Male | Cameroon | Chronic lymphoid leukemia | 1.33 | 1.25 | 1.41 |
| Male | Cameroon | Acute myeloid leukemia | 0.24 | 0.11 | 0.37 |
| Male | Cameroon | Chronic myeloid leukemia | 0.40 | 0.22 | 0.57 |
| Male | Cameroon | Leukemia | 0.29 | 0.18 | 0.39 |
| Male | Cameroon | Other leukemia | 0.76 | 0.57 | 0.94 |
| Female | Cameroon | Acute lymphoid leukemia | 0.20 | 0.09 | 0.31 |
| Female | Cameroon | Chronic lymphoid leukemia | 0.10 | -0.06 | 0.26 |
| Female | Cameroon | Acute myeloid leukemia | 0.41 | 0.32 | 0.51 |
| Female | Cameroon | Chronic myeloid leukemia | -0.85 | -1.04 | -0.66 |
| Female | Cameroon | Leukemia | -0.27 | -0.37 | -0.16 |
| Female | Cameroon | Other leukemia | -0.83 | -0.96 | -0.70 |
| Both | Cameroon | Acute lymphoid leukemia | -0.89 | -1.06 | -0.71 |
| Both | Cameroon | Chronic lymphoid leukemia | 0.54 | 0.42 | 0.66 |
| Both | Cameroon | Acute myeloid leukemia | 0.32 | 0.23 | 0.42 |
| Both | Cameroon | Chronic myeloid leukemia | -0.23 | -0.41 | -0.06 |
| Both | Cameroon | Leukemia | 0.06 | -0.04 | 0.16 |
| Both | Cameroon | Other leukemia | 0.15 | -0.01 | 0.30 |
| Male | Canada | Acute lymphoid leukemia | -2.55 | -2.75 | -2.35 |
| Male | Canada | Chronic lymphoid leukemia | -0.03 | -0.27 | 0.20 |
| Male | Canada | Acute myeloid leukemia | 0.26 | 0.10 | 0.41 |
| Male | Canada | Chronic myeloid leukemia | -4.26 | -4.72 | -3.79 |
| Male | Canada | Leukemia | -0.91 | -1.12 | -0.70 |
| Male | Canada | Other leukemia | -1.57 | -2.00 | -1.14 |
| Female | Canada | Acute lymphoid leukemia | -2.70 | -3.03 | -2.37 |
| Female | Canada | Chronic lymphoid leukemia | -0.02 | -0.28 | 0.24 |
| Female | Canada | Acute myeloid leukemia | 0.62 | 0.50 | 0.73 |

| | | | | | |
|---|---|---|---|---|---|
| Female | Canada | Chronic myeloid leukemia | -4.45 | -4.89 | -4.01 |
| Female | Canada | Leukemia | -0.86 | -0.99 | -0.74 |
| Female | Canada | Other leukemia | -1.57 | -1.87 | -1.27 |
| Both | Canada | Acute lymphoid leukemia | -2.61 | -2.81 | -2.40 |
| Both | Canada | Chronic lymphoid leukemia | 0.01 | -0.23 | 0.26 |
| Both | Canada | Acute myeloid leukemia | 0.43 | 0.32 | 0.54 |
| Both | Canada | Chronic myeloid leukemia | -4.33 | -4.78 | -3.87 |
| Both | Canada | Leukemia | -0.87 | -1.04 | -0.69 |
| Both | Canada | Other leukemia | -1.54 | -1.91 | -1.17 |
| Male | Cape Verde | Acute lymphoid leukemia | -0.57 | -0.89 | -0.25 |
| Male | Cape Verde | Chronic lymphoid leukemia | 2.02 | 1.72 | 2.32 |
| Male | Cape Verde | Acute myeloid leukemia | 1.53 | 1.23 | 1.82 |
| Male | Cape Verde | Chronic myeloid leukemia | 1.61 | 1.09 | 2.13 |
| Male | Cape Verde | Leukemia | 0.84 | 0.49 | 1.20 |
| Male | Cape Verde | Other leukemia | 0.30 | -0.11 | 0.71 |
| Female | Cape Verde | Acute lymphoid leukemia | 0.72 | 0.55 | 0.88 |
| Female | Cape Verde | Chronic lymphoid leukemia | 2.23 | 1.95 | 2.51 |
| Female | Cape Verde | Acute myeloid leukemia | 1.39 | 1.21 | 1.58 |
| Female | Cape Verde | Chronic myeloid leukemia | 0.14 | 0.02 | 0.26 |
| Female | Cape Verde | Leukemia | 0.76 | 0.54 | 0.97 |
| Female | Cape Verde | Other leukemia | -0.21 | -0.56 | 0.15 |
| Both | Cape Verde | Acute lymphoid leukemia | 0.00 | -0.23 | 0.24 |
| Both | Cape Verde | Chronic lymphoid leukemia | 2.15 | 1.89 | 2.42 |
| Both | Cape Verde | Acute myeloid leukemia | 1.66 | 1.41 | 1.91 |
| Both | Cape Verde | Chronic myeloid leukemia | 0.90 | 0.58 | 1.22 |
| Both | Cape Verde | Leukemia | 0.91 | 0.62 | 1.21 |
| Both | Cape Verde | Other leukemia | 0.30 | -0.09 | 0.68 |
| Male | Caribbean | Acute lymphoid leukemia | -0.88 | -1.01 | -0.74 |
| Male | Caribbean | Chronic lymphoid leukemia | 0.21 | -0.02 | 0.45 |
| Male | Caribbean | Acute myeloid leukemia | 0.07 | -0.07 | 0.22 |
| Male | Caribbean | Chronic myeloid leukemia | -1.43 | -1.61 | -1.25 |
| Male | Caribbean | Leukemia | -0.46 | -0.55 | -0.37 |
| Male | Caribbean | Other leukemia | -0.42 | -0.48 | -0.37 |
| Female | Caribbean | Acute lymphoid leukemia | -0.69 | -0.78 | -0.60 |
| Female | Caribbean | Chronic lymphoid leukemia | 0.53 | 0.31 | 0.75 |
| Female | Caribbean | Acute myeloid leukemia | -0.02 | -0.18 | 0.14 |
| Female | Caribbean | Chronic myeloid leukemia | -2.05 | -2.15 | -1.96 |
| Female | Caribbean | Leukemia | -0.73 | -0.82 | -0.64 |
| Female | Caribbean | Other leukemia | -1.03 | -1.13 | -0.94 |
| Both | Caribbean | Acute lymphoid leukemia | -0.81 | -0.92 | -0.69 |
| Both | Caribbean | Chronic lymphoid leukemia | 0.33 | 0.10 | 0.55 |
| Both | Caribbean | Acute myeloid leukemia | 0.02 | -0.13 | 0.17 |
| Both | Caribbean | Chronic myeloid leukemia | -1.70 | -1.84 | -1.57 |
| Both | Caribbean | Other leukemia | -0.72 | -0.79 | -0.65 |
| Both | Caribbean | Leukemia | -0.59 | -0.68 | -0.50 |
| Male | Central African Republic | Acute lymphoid leukemia | 1.19 | 1.12 | 1.26 |
| Male | Central African Republic | Chronic lymphoid leukemia | 2.70 | 2.51 | 2.88 |
| Male | Central African Republic | Acute myeloid leukemia | -0.46 | -0.60 | -0.31 |

| | | | | | |
|---|---|---|---|---|---|
| Male | Central African Republic | Chronic myeloid leukemia | 1.60 | 1.45 | 1.75 |
| Male | Central African Republic | Leukemia | 0.15 | 0.07 | 0.23 |
| Male | Central African Republic | Other leukemia | -0.09 | -0.17 | 0.00 |
| Female | Central African Republic | Acute lymphoid leukemia | 0.54 | 0.46 | 0.62 |
| Female | Central African Republic | Chronic lymphoid leukemia | 0.56 | 0.44 | 0.68 |
| Female | Central African Republic | Acute myeloid leukemia | 1.16 | 1.04 | 1.27 |
| Female | Central African Republic | Chronic myeloid leukemia | 0.11 | -0.02 | 0.24 |
| Female | Central African Republic | Leukemia | -0.22 | -0.32 | -0.11 |
| Female | Central African Republic | Other leukemia | -0.84 | -0.99 | -0.69 |
| Both | Central African Republic | Acute lymphoid leukemia | 0.94 | 0.87 | 1.00 |
| Both | Central African Republic | Chronic lymphoid leukemia | 1.44 | 1.31 | 1.57 |
| Both | Central African Republic | Acute myeloid leukemia | -0.08 | -0.18 | 0.03 |
| Both | Central African Republic | Chronic myeloid leukemia | 0.63 | 0.50 | 0.76 |
| Both | Central African Republic | Other leukemia | -0.54 | -0.66 | -0.42 |
| Both | Central African Republic | Leukemia | -0.04 | -0.12 | 0.05 |
| Male | Central Asia | Acute lymphoid leukemia | 0.04 | -0.09 | 0.16 |
| Male | Central Asia | Chronic lymphoid leukemia | 1.68 | 1.44 | 1.93 |
| Male | Central Asia | Acute myeloid leukemia | 0.61 | 0.45 | 0.78 |
| Male | Central Asia | Chronic myeloid leukemia | -1.58 | -1.72 | -1.44 |
| Male | Central Asia | Leukemia | -0.07 | -0.17 | 0.03 |
| Male | Central Asia | Other leukemia | -0.55 | -0.75 | -0.36 |
| Female | Central Asia | Acute lymphoid leukemia | -0.39 | -0.51 | -0.28 |
| Female | Central Asia | Chronic lymphoid leukemia | 0.11 | 0.01 | 0.20 |
| Female | Central Asia | Acute myeloid leukemia | 0.67 | 0.53 | 0.82 |
| Female | Central Asia | Chronic myeloid leukemia | -1.07 | -1.42 | -0.72 |
| Female | Central Asia | Leukemia | -0.34 | -0.50 | -0.19 |
| Female | Central Asia | Other leukemia | -0.95 | -1.21 | -0.69 |
| Both | Central Asia | Leukemia | -0.20 | -0.31 | -0.08 |
| Both | Central Asia | Acute lymphoid leukemia | -0.13 | -0.24 | -0.01 |
| Both | Central Asia | Chronic lymphoid leukemia | 0.85 | 0.70 | 1.01 |
| Both | Central Asia | Acute myeloid leukemia | 0.63 | 0.50 | 0.75 |
| Both | Central Asia | Chronic myeloid leukemia | -1.34 | -1.57 | -1.11 |
| Both | Central Asia | Other leukemia | -0.74 | -0.96 | -0.51 |
| Male | Central Europe | Acute lymphoid leukemia | -1.60 | -1.72 | -1.48 |
| Male | Central Europe | Chronic lymphoid leukemia | 2.39 | 2.24 | 2.54 |
| Male | Central Europe | Acute myeloid leukemia | 0.56 | 0.44 | 0.67 |
| Male | Central Europe | Chronic myeloid leukemia | -2.34 | -2.60 | -2.09 |
| Male | Central Europe | Leukemia | 0.21 | 0.07 | 0.35 |
| Male | Central Europe | Other leukemia | -0.37 | -0.74 | 0.00 |
| Female | Central Europe | Acute lymphoid leukemia | -1.85 | -1.92 | -1.77 |
| Female | Central Europe | Chronic lymphoid leukemia | 1.85 | 1.69 | 2.01 |
| Female | Central Europe | Acute myeloid leukemia | 0.42 | 0.28 | 0.56 |
| Female | Central Europe | Chronic myeloid leukemia | -3.73 | -4.08 | -3.39 |
| Female | Central Europe | Leukemia | -0.19 | -0.36 | -0.01 |
| Female | Central Europe | Other leukemia | -0.69 | -1.10 | -0.28 |
| Both | Central Europe | Leukemia | 0.02 | -0.12 | 0.17 |
| Both | Central Europe | Acute lymphoid leukemia | -1.69 | -1.79 | -1.59 |
| Both | Central Europe | Chronic lymphoid leukemia | 2.20 | 2.06 | 2.34 |

| | | | | | |
|---|---|---|---|---|---|
| Both | Central Europe | Acute myeloid leukemia | 0.48 | 0.36 | 0.60 |
| Both | Central Europe | Chronic myeloid leukemia | -2.87 | -3.14 | -2.60 |
| Both | Central Europe | Other leukemia | -0.53 | -0.91 | -0.15 |
| Male | Central Latin America | Acute lymphoid leukemia | 0.83 | 0.68 | 0.97 |
| Male | Central Latin America | Chronic lymphoid leukemia | 0.96 | 0.85 | 1.07 |
| Male | Central Latin America | Acute myeloid leukemia | 0.56 | 0.46 | 0.66 |
| Male | Central Latin America | Chronic myeloid leukemia | -1.07 | -1.28 | -0.87 |
| Male | Central Latin America | Leukemia | -0.23 | -0.29 | -0.16 |
| Male | Central Latin America | Other leukemia | -1.95 | -2.03 | -1.87 |
| Female | Central Latin America | Acute lymphoid leukemia | 0.77 | 0.64 | 0.91 |
| Female | Central Latin America | Chronic lymphoid leukemia | 0.07 | -0.06 | 0.20 |
| Female | Central Latin America | Acute myeloid leukemia | 0.73 | 0.64 | 0.81 |
| Female | Central Latin America | Chronic myeloid leukemia | -1.99 | -2.27 | -1.70 |
| Female | Central Latin America | Leukemia | -0.42 | -0.47 | -0.37 |
| Female | Central Latin America | Other leukemia | -2.18 | -2.25 | -2.11 |
| Both | Central Latin America | Leukemia | -0.32 | -0.38 | -0.27 |
| Both | Central Latin America | Acute lymphoid leukemia | 0.80 | 0.67 | 0.94 |
| Both | Central Latin America | Chronic lymphoid leukemia | 0.55 | 0.45 | 0.65 |
| Both | Central Latin America | Acute myeloid leukemia | 0.63 | 0.54 | 0.72 |
| Both | Central Latin America | Chronic myeloid leukemia | -1.47 | -1.70 | -1.23 |
| Both | Central Latin America | Other leukemia | -2.06 | -2.13 | -2.00 |
| Male | Central Sub-Saharan Africa | Acute lymphoid leukemia | 0.24 | 0.20 | 0.29 |
| Male | Central Sub-Saharan Africa | Chronic lymphoid leukemia | 3.03 | 2.85 | 3.22 |
| Male | Central Sub-Saharan Africa | Acute myeloid leukemia | -0.23 | -0.37 | -0.10 |
| Male | Central Sub-Saharan Africa | Chronic myeloid leukemia | 1.44 | 1.31 | 1.57 |
| Male | Central Sub-Saharan Africa | Leukemia | -0.05 | -0.11 | 0.00 |
| Male | Central Sub-Saharan Africa | Other leukemia | -0.62 | -0.70 | -0.54 |
| Female | Central Sub-Saharan Africa | Acute lymphoid leukemia | 0.56 | 0.45 | 0.67 |
| Female | Central Sub-Saharan Africa | Chronic lymphoid leukemia | 1.08 | 1.00 | 1.17 |
| Female | Central Sub-Saharan Africa | Acute myeloid leukemia | 1.05 | 0.96 | 1.14 |
| Female | Central Sub-Saharan Africa | Chronic myeloid leukemia | 0.10 | -0.02 | 0.22 |
| Female | Central Sub-Saharan Africa | Leukemia | -0.37 | -0.44 | -0.30 |
| Female | Central Sub-Saharan Africa | Other leukemia | -1.19 | -1.33 | -1.05 |
| Both | Central Sub-Saharan Africa | Acute lymphoid leukemia | 0.36 | 0.31 | 0.42 |
| Both | Central Sub-Saharan Africa | Chronic lymphoid leukemia | 1.88 | 1.76 | 1.99 |
| Both | Central Sub-Saharan Africa | Acute myeloid leukemia | -0.04 | -0.16 | 0.07 |
| Both | Central Sub-Saharan Africa | Chronic myeloid leukemia | 0.58 | 0.47 | 0.69 |
| Both | Central Sub-Saharan Africa | Other leukemia | -0.95 | -1.07 | -0.83 |
| Both | Central Sub-Saharan Africa | Leukemia | -0.24 | -0.27 | -0.20 |
| Male | Chad | Acute lymphoid leukemia | -1.04 | -1.20 | -0.87 |
| Male | Chad | Chronic lymphoid leukemia | 1.17 | 1.11 | 1.23 |
| Male | Chad | Acute myeloid leukemia | 0.72 | 0.60 | 0.83 |
| Male | Chad | Chronic myeloid leukemia | 1.05 | 0.87 | 1.22 |
| Male | Chad | Leukemia | 0.74 | 0.62 | 0.87 |
| Male | Chad | Other leukemia | 1.19 | 0.98 | 1.41 |
| Female | Chad | Acute lymphoid leukemia | 0.94 | 0.85 | 1.03 |
| Female | Chad | Chronic lymphoid leukemia | 0.78 | 0.68 | 0.88 |
| Female | Chad | Acute myeloid leukemia | 1.16 | 1.05 | 1.27 |

| | | | | | |
|---|---|---|---|---|---|
| Female | Chad | Chronic myeloid leukemia | 0.14 | -0.01 | 0.28 |
| Female | Chad | Leukemia | 0.52 | 0.41 | 0.63 |
| Female | Chad | Other leukemia | 0.11 | -0.01 | 0.24 |
| Both | Chad | Acute lymphoid leukemia | -0.24 | -0.35 | -0.14 |
| Both | Chad | Chronic lymphoid leukemia | 0.71 | 0.62 | 0.80 |
| Both | Chad | Acute myeloid leukemia | 0.93 | 0.85 | 1.01 |
| Both | Chad | Chronic myeloid leukemia | 0.53 | 0.39 | 0.67 |
| Both | Chad | Leukemia | 0.65 | 0.54 | 0.76 |
| Both | Chad | Other leukemia | 0.73 | 0.57 | 0.90 |
| Male | Chile | Acute lymphoid leukemia | -0.47 | -0.62 | -0.31 |
| Male | Chile | Chronic lymphoid leukemia | 1.57 | 1.25 | 1.89 |
| Male | Chile | Acute myeloid leukemia | 0.98 | 0.80 | 1.16 |
| Male | Chile | Chronic myeloid leukemia | -2.39 | -2.92 | -1.86 |
| Male | Chile | Leukemia | -0.03 | -0.12 | 0.05 |
| Male | Chile | Other leukemia | -0.15 | -0.56 | 0.26 |
| Female | Chile | Acute lymphoid leukemia | -0.13 | -0.38 | 0.12 |
| Female | Chile | Chronic lymphoid leukemia | 1.62 | 1.32 | 1.91 |
| Female | Chile | Acute myeloid leukemia | 0.79 | 0.69 | 0.88 |
| Female | Chile | Chronic myeloid leukemia | -2.09 | -2.51 | -1.68 |
| Female | Chile | Leukemia | -0.11 | -0.20 | -0.01 |
| Female | Chile | Other leukemia | -0.79 | -1.17 | -0.41 |
| Both | Chile | Acute lymphoid leukemia | -0.32 | -0.50 | -0.14 |
| Both | Chile | Chronic lymphoid leukemia | 1.57 | 1.30 | 1.85 |
| Both | Chile | Acute myeloid leukemia | 0.88 | 0.76 | 0.99 |
| Both | Chile | Chronic myeloid leukemia | -2.27 | -2.74 | -1.80 |
| Both | Chile | Leukemia | -0.08 | -0.16 | 0.00 |
| Both | Chile | Other leukemia | -0.43 | -0.82 | -0.04 |
| Male | China | Acute lymphoid leukemia | 1.23 | 0.72 | 1.74 |
| Male | China | Chronic lymphoid leukemia | 6.30 | 5.68 | 6.94 |
| Male | China | Acute myeloid leukemia | 1.64 | 1.48 | 1.80 |
| Male | China | Chronic myeloid leukemia | -0.10 | -0.31 | 0.11 |
| Male | China | Leukemia | 1.01 | 0.78 | 1.24 |
| Male | China | Other leukemia | 0.57 | 0.38 | 0.75 |
| Female | China | Acute lymphoid leukemia | 1.46 | 1.07 | 1.84 |
| Female | China | Chronic lymphoid leukemia | 5.53 | 4.79 | 6.27 |
| Female | China | Acute myeloid leukemia | 1.47 | 1.28 | 1.65 |
| Female | China | Chronic myeloid leukemia | -2.52 | -2.80 | -2.24 |
| Female | China | Leukemia | 0.59 | 0.42 | 0.76 |
| Female | China | Other leukemia | 0.14 | 0.00 | 0.27 |
| Both | China | Acute lymphoid leukemia | 1.31 | 0.86 | 1.77 |
| Both | China | Chronic lymphoid leukemia | 5.99 | 5.32 | 6.66 |
| Both | China | Acute myeloid leukemia | 1.54 | 1.39 | 1.70 |
| Both | China | Chronic myeloid leukemia | -1.06 | -1.30 | -0.82 |
| Both | China | Leukemia | 0.82 | 0.62 | 1.02 |
| Both | China | Other leukemia | 0.37 | 0.22 | 0.53 |
| Male | Colombia | Acute lymphoid leukemia | -0.78 | -0.95 | -0.60 |
| Male | Colombia | Chronic lymphoid leukemia | -0.80 | -1.03 | -0.57 |
| Male | Colombia | Acute myeloid leukemia | 1.00 | 0.80 | 1.20 |

| | | | | | |
|---|---|---|---|---|---|
| Male | Colombia | Chronic myeloid leukemia | -1.41 | -1.66 | -1.16 |
| Male | Colombia | Leukemia | -0.87 | -1.02 | -0.71 |
| Male | Colombia | Other leukemia | -1.91 | -2.29 | -1.53 |
| Female | Colombia | Acute lymphoid leukemia | -0.92 | -1.15 | -0.70 |
| Female | Colombia | Chronic lymphoid leukemia | -1.65 | -2.12 | -1.17 |
| Female | Colombia | Acute myeloid leukemia | 0.80 | 0.61 | 0.99 |
| Female | Colombia | Chronic myeloid leukemia | -2.78 | -3.00 | -2.56 |
| Female | Colombia | Leukemia | -1.36 | -1.54 | -1.17 |
| Female | Colombia | Other leukemia | -2.62 | -2.89 | -2.36 |
| Both | Colombia | Acute lymphoid leukemia | -0.85 | -1.02 | -0.67 |
| Both | Colombia | Chronic lymphoid leukemia | -1.24 | -1.55 | -0.93 |
| Both | Colombia | Acute myeloid leukemia | 0.89 | 0.70 | 1.07 |
| Both | Colombia | Chronic myeloid leukemia | -2.02 | -2.23 | -1.80 |
| Both | Colombia | Leukemia | -1.11 | -1.27 | -0.95 |
| Both | Colombia | Other leukemia | -2.26 | -2.58 | -1.93 |
| Male | Comoros | Acute lymphoid leukemia | -0.50 | -0.77 | -0.23 |
| Male | Comoros | Chronic lymphoid leukemia | 2.90 | 2.67 | 3.13 |
| Male | Comoros | Acute myeloid leukemia | 0.71 | 0.60 | 0.83 |
| Male | Comoros | Chronic myeloid leukemia | -0.41 | -0.63 | -0.18 |
| Male | Comoros | Leukemia | 0.10 | -0.07 | 0.28 |
| Male | Comoros | Other leukemia | -1.12 | -1.31 | -0.93 |
| Female | Comoros | Acute lymphoid leukemia | 0.84 | 0.68 | 1.01 |
| Female | Comoros | Chronic lymphoid leukemia | 0.87 | 0.82 | 0.93 |
| Female | Comoros | Acute myeloid leukemia | 0.89 | 0.77 | 1.01 |
| Female | Comoros | Chronic myeloid leukemia | -0.64 | -0.67 | -0.61 |
| Female | Comoros | Leukemia | -0.26 | -0.32 | -0.20 |
| Female | Comoros | Other leukemia | -1.38 | -1.45 | -1.31 |
| Both | Comoros | Acute lymphoid leukemia | 0.11 | -0.10 | 0.33 |
| Both | Comoros | Chronic lymphoid leukemia | 1.71 | 1.59 | 1.83 |
| Both | Comoros | Acute myeloid leukemia | 0.66 | 0.55 | 0.76 |
| Both | Comoros | Chronic myeloid leukemia | -0.54 | -0.64 | -0.44 |
| Both | Comoros | Leukemia | -0.10 | -0.21 | 0.01 |
| Both | Comoros | Other leukemia | -1.26 | -1.34 | -1.18 |
| Male | Costa Rica | Acute lymphoid leukemia | 0.77 | 0.44 | 1.10 |
| Male | Costa Rica | Chronic lymphoid leukemia | 2.63 | 2.04 | 3.23 |
| Male | Costa Rica | Acute myeloid leukemia | 0.23 | 0.00 | 0.47 |
| Male | Costa Rica | Chronic myeloid leukemia | -0.66 | -0.89 | -0.43 |
| Male | Costa Rica | Leukemia | 0.30 | 0.11 | 0.48 |
| Male | Costa Rica | Other leukemia | -0.95 | -1.12 | -0.79 |
| Female | Costa Rica | Acute lymphoid leukemia | -0.49 | -0.81 | -0.18 |
| Female | Costa Rica | Chronic lymphoid leukemia | 1.99 | 1.56 | 2.43 |
| Female | Costa Rica | Acute myeloid leukemia | -0.25 | -0.47 | -0.03 |
| Female | Costa Rica | Chronic myeloid leukemia | -0.88 | -1.24 | -0.52 |
| Female | Costa Rica | Leukemia | -0.43 | -0.63 | -0.24 |
| Female | Costa Rica | Other leukemia | -1.50 | -1.70 | -1.29 |
| Both | Costa Rica | Acute lymphoid leukemia | 0.18 | -0.12 | 0.47 |
| Both | Costa Rica | Chronic lymphoid leukemia | 2.33 | 1.89 | 2.78 |
| Both | Costa Rica | Acute myeloid leukemia | -0.02 | -0.23 | 0.19 |

| | | | | | |
|---|---|---|---|---|---|
| Both | Costa Rica | Chronic myeloid leukemia | -0.79 | -1.04 | -0.54 |
| Both | Costa Rica | Leukemia | -0.05 | -0.22 | 0.12 |
| Both | Costa Rica | Other leukemia | -1.23 | -1.37 | -1.08 |
| Male | Croatia | Acute lymphoid leukemia | -2.33 | -2.58 | -2.07 |
| Male | Croatia | Chronic lymphoid leukemia | 1.72 | 1.33 | 2.12 |
| Male | Croatia | Acute myeloid leukemia | 1.48 | 1.15 | 1.82 |
| Male | Croatia | Chronic myeloid leukemia | -2.79 | -3.00 | -2.58 |
| Male | Croatia | Leukemia | 0.13 | -0.07 | 0.33 |
| Male | Croatia | Other leukemia | -1.28 | -1.70 | -0.85 |
| Female | Croatia | Acute lymphoid leukemia | -2.27 | -2.59 | -1.96 |
| Female | Croatia | Chronic lymphoid leukemia | 0.74 | 0.42 | 1.07 |
| Female | Croatia | Acute myeloid leukemia | 0.30 | 0.07 | 0.52 |
| Female | Croatia | Chronic myeloid leukemia | -3.67 | -4.01 | -3.32 |
| Female | Croatia | Leukemia | -1.09 | -1.26 | -0.92 |
| Female | Croatia | Other leukemia | -3.11 | -3.63 | -2.58 |
| Both | Croatia | Acute lymphoid leukemia | -2.29 | -2.50 | -2.08 |
| Both | Croatia | Chronic lymphoid leukemia | 1.41 | 1.07 | 1.74 |
| Both | Croatia | Acute myeloid leukemia | 0.97 | 0.70 | 1.24 |
| Both | Croatia | Chronic myeloid leukemia | -3.08 | -3.27 | -2.89 |
| Both | Croatia | Leukemia | -0.33 | -0.48 | -0.19 |
| Both | Croatia | Other leukemia | -2.01 | -2.44 | -1.58 |
| Male | Cuba | Acute lymphoid leukemia | -1.75 | -1.99 | -1.51 |
| Male | Cuba | Chronic lymphoid leukemia | -0.40 | -0.72 | -0.09 |
| Male | Cuba | Acute myeloid leukemia | -0.64 | -0.83 | -0.46 |
| Male | Cuba | Chronic myeloid leukemia | -1.58 | -1.76 | -1.41 |
| Male | Cuba | Leukemia | -1.15 | -1.36 | -0.94 |
| Male | Cuba | Other leukemia | -1.37 | -1.60 | -1.14 |
| Female | Cuba | Acute lymphoid leukemia | -2.05 | -2.20 | -1.90 |
| Female | Cuba | Chronic lymphoid leukemia | 0.44 | 0.16 | 0.73 |
| Female | Cuba | Acute myeloid leukemia | -0.72 | -0.88 | -0.56 |
| Female | Cuba | Chronic myeloid leukemia | -2.37 | -2.50 | -2.25 |
| Female | Cuba | Leukemia | -1.32 | -1.46 | -1.17 |
| Female | Cuba | Other leukemia | -1.93 | -2.15 | -1.71 |
| Both | Cuba | Acute lymphoid leukemia | -1.87 | -2.05 | -1.69 |
| Both | Cuba | Chronic lymphoid leukemia | -0.06 | -0.35 | 0.23 |
| Both | Cuba | Acute myeloid leukemia | -0.69 | -0.85 | -0.52 |
| Both | Cuba | Chronic myeloid leukemia | -1.92 | -2.05 | -1.79 |
| Both | Cuba | Leukemia | -1.23 | -1.41 | -1.06 |
| Both | Cuba | Other leukemia | -1.64 | -1.85 | -1.43 |
| Male | Cyprus | Acute lymphoid leukemia | -0.30 | -0.70 | 0.10 |
| Male | Cyprus | Chronic lymphoid leukemia | 4.10 | 3.60 | 4.60 |
| Male | Cyprus | Acute myeloid leukemia | 2.30 | 2.10 | 2.50 |
| Male | Cyprus | Chronic myeloid leukemia | 0.45 | -0.18 | 1.09 |
| Male | Cyprus | Leukemia | 1.80 | 1.43 | 2.16 |
| Male | Cyprus | Other leukemia | -0.05 | -0.58 | 0.49 |
| Female | Cyprus | Acute lymphoid leukemia | -2.59 | -2.77 | -2.42 |
| Female | Cyprus | Chronic lymphoid leukemia | 2.29 | 2.02 | 2.56 |
| Female | Cyprus | Acute myeloid leukemia | -0.56 | -0.68 | -0.44 |

| | | | | | |
|---|---|---|---|---|---|
| Female | Cyprus | Chronic myeloid leukemia | -4.57 | -5.02 | -4.12 |
| Female | Cyprus | Leukemia | -0.42 | -0.52 | -0.32 |
| Female | Cyprus | Other leukemia | -1.71 | -1.98 | -1.44 |
| Both | Cyprus | Acute lymphoid leukemia | -1.33 | -1.60 | -1.05 |
| Both | Cyprus | Chronic lymphoid leukemia | 3.29 | 2.94 | 3.65 |
| Both | Cyprus | Acute myeloid leukemia | 1.03 | 0.90 | 1.16 |
| Both | Cyprus | Chronic myeloid leukemia | -1.65 | -2.08 | -1.23 |
| Both | Cyprus | Leukemia | 0.82 | 0.60 | 1.03 |
| Both | Cyprus | Other leukemia | -0.77 | -1.16 | -0.37 |
| Male | Czech Republic | Acute lymphoid leukemia | -4.23 | -4.88 | -3.58 |
| Male | Czech Republic | Chronic lymphoid leukemia | 2.09 | 1.52 | 2.67 |
| Male | Czech Republic | Acute myeloid leukemia | -0.67 | -0.95 | -0.40 |
| Male | Czech Republic | Chronic myeloid leukemia | -1.88 | -2.33 | -1.42 |
| Male | Czech Republic | Leukemia | -0.68 | -0.92 | -0.45 |
| Male | Czech Republic | Other leukemia | -1.45 | -1.81 | -1.09 |
| Female | Czech Republic | Acute lymphoid leukemia | -4.93 | -5.34 | -4.53 |
| Female | Czech Republic | Chronic lymphoid leukemia | 1.62 | 1.02 | 2.21 |
| Female | Czech Republic | Acute myeloid leukemia | -0.80 | -1.03 | -0.57 |
| Female | Czech Republic | Chronic myeloid leukemia | -4.43 | -5.17 | -3.69 |
| Female | Czech Republic | Leukemia | -1.35 | -1.54 | -1.16 |
| Female | Czech Republic | Other leukemia | -1.83 | -2.17 | -1.48 |
| Both | Czech Republic | Acute lymphoid leukemia | -4.41 | -4.95 | -3.87 |
| Both | Czech Republic | Chronic lymphoid leukemia | 2.01 | 1.45 | 2.58 |
| Both | Czech Republic | Acute myeloid leukemia | -0.69 | -0.94 | -0.45 |
| Both | Czech Republic | Chronic myeloid leukemia | -2.77 | -3.25 | -2.28 |
| Both | Czech Republic | Leukemia | -0.93 | -1.13 | -0.73 |
| Both | Czech Republic | Other leukemia | -1.60 | -1.93 | -1.26 |
| Male | Democratic Republic of the Congo | Acute lymphoid leukemia | 0.36 | 0.22 | 0.51 |
| Male | Democratic Republic of the Congo | Chronic lymphoid leukemia | 2.94 | 2.75 | 3.13 |
| Male | Democratic Republic of the Congo | Acute myeloid leukemia | -0.38 | -0.58 | -0.18 |
| Male | Democratic Republic of the Congo | Chronic myeloid leukemia | 1.69 | 1.54 | 1.84 |
| Male | Democratic Republic of the Congo | Leukemia | 0.01 | -0.10 | 0.12 |
| Male | Democratic Republic of the Congo | Other leukemia | -0.31 | -0.37 | -0.25 |
| Female | Democratic Republic of the Congo | Acute lymphoid leukemia | 0.45 | 0.29 | 0.61 |
| Female | Democratic Republic of the Congo | Chronic lymphoid leukemia | 1.12 | 1.01 | 1.23 |
| Female | Democratic Republic of the Congo | Acute myeloid leukemia | 0.98 | 0.86 | 1.10 |
| Female | Democratic Republic of the Congo | Chronic myeloid leukemia | 0.35 | 0.25 | 0.45 |
| Female | Democratic Republic of the Congo | Leukemia | -0.19 | -0.23 | -0.15 |
| Female | Democratic Republic of the Congo | Other leukemia | -0.90 | -1.00 | -0.80 |
| Both | Democratic Republic of the Congo | Acute lymphoid leukemia | 0.39 | 0.24 | 0.54 |
| Both | Democratic Republic of the Congo | Chronic lymphoid leukemia | 1.84 | 1.71 | 1.97 |
| Both | Democratic Republic of the Congo | Acute myeloid leukemia | -0.18 | -0.38 | 0.01 |
| Both | Democratic Republic of the Congo | Chronic myeloid leukemia | 0.81 | 0.71 | 0.91 |
| Both | Democratic Republic of the Congo | Leukemia | -0.12 | -0.19 | -0.06 |
| Both | Democratic Republic of the Congo | Other leukemia | -0.65 | -0.73 | -0.57 |
| Male | Denmark | Acute lymphoid leukemia | -2.48 | -2.85 | -2.12 |
| Male | Denmark | Chronic lymphoid leukemia | -0.48 | -0.98 | 0.02 |
| Male | Denmark | Acute myeloid leukemia | -0.93 | -1.49 | -0.38 |

| | | | | | |
|---|---|---|---|---|---|
| Male | Denmark | Chronic myeloid leukemia | -2.83 | -3.28 | -2.38 |
| Male | Denmark | Leukemia | -1.23 | -1.60 | -0.85 |
| Male | Denmark | Other leukemia | -1.68 | -2.03 | -1.33 |
| Female | Denmark | Acute lymphoid leukemia | -2.25 | -2.65 | -1.85 |
| Female | Denmark | Chronic lymphoid leukemia | -0.78 | -1.44 | -0.11 |
| Female | Denmark | Acute myeloid leukemia | -1.57 | -2.14 | -0.99 |
| Female | Denmark | Chronic myeloid leukemia | -4.25 | -4.70 | -3.80 |
| Female | Denmark | Leukemia | -1.81 | -2.27 | -1.35 |
| Female | Denmark | Other leukemia | -2.42 | -2.77 | -2.08 |
| Both | Denmark | Acute lymphoid leukemia | -2.37 | -2.72 | -2.03 |
| Both | Denmark | Chronic lymphoid leukemia | -0.53 | -1.09 | 0.04 |
| Both | Denmark | Acute myeloid leukemia | -1.23 | -1.79 | -0.67 |
| Both | Denmark | Chronic myeloid leukemia | -3.35 | -3.78 | -2.92 |
| Both | Denmark | Leukemia | -1.44 | -1.84 | -1.03 |
| Both | Denmark | Other leukemia | -1.95 | -2.27 | -1.62 |
| Male | Djibouti | Acute lymphoid leukemia | -0.49 | -0.75 | -0.23 |
| Male | Djibouti | Chronic lymphoid leukemia | 3.04 | 2.88 | 3.21 |
| Male | Djibouti | Acute myeloid leukemia | 0.76 | 0.64 | 0.88 |
| Male | Djibouti | Chronic myeloid leukemia | -0.01 | -0.11 | 0.08 |
| Male | Djibouti | Leukemia | 0.12 | -0.04 | 0.28 |
| Male | Djibouti | Other leukemia | -1.30 | -1.58 | -1.02 |
| Female | Djibouti | Acute lymphoid leukemia | 1.20 | 1.02 | 1.38 |
| Female | Djibouti | Chronic lymphoid leukemia | 0.62 | 0.58 | 0.66 |
| Female | Djibouti | Acute myeloid leukemia | 0.85 | 0.75 | 0.95 |
| Female | Djibouti | Chronic myeloid leukemia | -0.70 | -0.81 | -0.59 |
| Female | Djibouti | Leukemia | -0.31 | -0.46 | -0.16 |
| Female | Djibouti | Other leukemia | -1.50 | -1.76 | -1.23 |
| Both | Djibouti | Acute lymphoid leukemia | 0.15 | -0.06 | 0.37 |
| Both | Djibouti | Chronic lymphoid leukemia | 1.58 | 1.50 | 1.66 |
| Both | Djibouti | Acute myeloid leukemia | 0.84 | 0.72 | 0.97 |
| Both | Djibouti | Chronic myeloid leukemia | -0.41 | -0.50 | -0.32 |
| Both | Djibouti | Leukemia | -0.04 | -0.19 | 0.11 |
| Both | Djibouti | Other leukemia | -1.42 | -1.69 | -1.15 |
| Male | Dominica | Acute lymphoid leukemia | 1.97 | 1.79 | 2.15 |
| Male | Dominica | Chronic lymphoid leukemia | 0.86 | 0.62 | 1.09 |
| Male | Dominica | Acute myeloid leukemia | 0.07 | -0.06 | 0.20 |
| Male | Dominica | Chronic myeloid leukemia | -0.06 | -0.16 | 0.04 |
| Male | Dominica | Leukemia | 0.45 | 0.33 | 0.57 |
| Male | Dominica | Other leukemia | -0.55 | -0.65 | -0.45 |
| Female | Dominica | Acute lymphoid leukemia | 1.96 | 1.81 | 2.12 |
| Female | Dominica | Chronic lymphoid leukemia | 1.71 | 1.59 | 1.83 |
| Female | Dominica | Acute myeloid leukemia | 1.54 | 1.39 | 1.69 |
| Female | Dominica | Chronic myeloid leukemia | -3.38 | -3.80 | -2.95 |
| Female | Dominica | Leukemia | 0.62 | 0.48 | 0.76 |
| Female | Dominica | Other leukemia | -0.23 | -0.42 | -0.04 |
| Both | Dominica | Acute lymphoid leukemia | 2.03 | 1.86 | 2.19 |
| Both | Dominica | Chronic lymphoid leukemia | 1.40 | 1.22 | 1.59 |
| Both | Dominica | Acute myeloid leukemia | 1.13 | 1.01 | 1.26 |

| | | | | | |
|---|---|---|---|---|---|
| Both | Dominica | Chronic myeloid leukemia | 0.17 | 0.09 | 0.24 |
| Both | Dominica | Leukemia | 0.60 | 0.48 | 0.72 |
| Both | Dominica | Other leukemia | -0.35 | -0.50 | -0.21 |
| Male | Dominican Republic | Acute lymphoid leukemia | 0.06 | -0.16 | 0.29 |
| Male | Dominican Republic | Chronic lymphoid leukemia | 3.90 | 3.73 | 4.08 |
| Male | Dominican Republic | Acute myeloid leukemia | 2.85 | 2.62 | 3.09 |
| Male | Dominican Republic | Chronic myeloid leukemia | 1.50 | 0.97 | 2.04 |
| Male | Dominican Republic | Leukemia | 0.25 | 0.09 | 0.41 |
| Male | Dominican Republic | Other leukemia | -0.69 | -0.87 | -0.51 |
| Female | Dominican Republic | Acute lymphoid leukemia | 2.93 | 2.55 | 3.30 |
| Female | Dominican Republic | Chronic lymphoid leukemia | 4.26 | 4.01 | 4.51 |
| Female | Dominican Republic | Acute myeloid leukemia | 3.35 | 2.99 | 3.70 |
| Female | Dominican Republic | Chronic myeloid leukemia | 0.60 | 0.35 | 0.85 |
| Female | Dominican Republic | Leukemia | 0.05 | -0.14 | 0.24 |
| Female | Dominican Republic | Other leukemia | -1.38 | -1.59 | -1.17 |
| Both | Dominican Republic | Acute lymphoid leukemia | 0.89 | 0.71 | 1.06 |
| Both | Dominican Republic | Chronic lymphoid leukemia | 4.01 | 3.91 | 4.12 |
| Both | Dominican Republic | Acute myeloid leukemia | 3.08 | 2.80 | 3.36 |
| Both | Dominican Republic | Chronic myeloid leukemia | 1.22 | 0.94 | 1.51 |
| Both | Dominican Republic | Leukemia | 0.15 | 0.00 | 0.31 |
| Both | Dominican Republic | Other leukemia | -1.02 | -1.20 | -0.84 |
| Male | East Asia | Acute lymphoid leukemia | 1.21 | 0.71 | 1.70 |
| Male | East Asia | Chronic lymphoid leukemia | 6.26 | 5.64 | 6.88 |
| Male | East Asia | Acute myeloid leukemia | 1.67 | 1.52 | 1.81 |
| Male | East Asia | Chronic myeloid leukemia | -0.10 | -0.30 | 0.10 |
| Male | East Asia | Leukemia | 1.02 | 0.79 | 1.25 |
| Male | East Asia | Other leukemia | 0.57 | 0.39 | 0.76 |
| Female | East Asia | Acute lymphoid leukemia | 1.42 | 1.05 | 1.80 |
| Female | East Asia | Chronic lymphoid leukemia | 5.48 | 4.75 | 6.20 |
| Female | East Asia | Acute myeloid leukemia | 1.45 | 1.28 | 1.62 |
| Female | East Asia | Chronic myeloid leukemia | -2.35 | -2.61 | -2.08 |
| Female | East Asia | Leukemia | 0.59 | 0.43 | 0.76 |
| Female | East Asia | Other leukemia | 0.14 | 0.01 | 0.27 |
| Both | East Asia | Acute lymphoid leukemia | 1.29 | 0.85 | 1.73 |
| Both | East Asia | Chronic lymphoid leukemia | 5.94 | 5.28 | 6.60 |
| Both | East Asia | Acute myeloid leukemia | 1.55 | 1.40 | 1.69 |
| Both | East Asia | Chronic myeloid leukemia | -0.99 | -1.21 | -0.76 |
| Both | East Asia | Other leukemia | 0.38 | 0.22 | 0.53 |
| Both | East Asia | Leukemia | 0.83 | 0.63 | 1.02 |
| Male | Eastern Europe | Acute lymphoid leukemia | -1.56 | -1.69 | -1.43 |
| Male | Eastern Europe | Chronic lymphoid leukemia | 2.16 | 1.93 | 2.40 |
| Male | Eastern Europe | Acute myeloid leukemia | 0.14 | -0.06 | 0.34 |
| Male | Eastern Europe | Chronic myeloid leukemia | -0.60 | -0.90 | -0.29 |
| Male | Eastern Europe | Leukemia | 0.14 | -0.01 | 0.29 |
| Male | Eastern Europe | Other leukemia | -0.23 | -0.45 | 0.00 |
| Female | Eastern Europe | Acute lymphoid leukemia | -1.60 | -1.68 | -1.52 |
| Female | Eastern Europe | Chronic lymphoid leukemia | 2.65 | 2.32 | 2.99 |
| Female | Eastern Europe | Acute myeloid leukemia | -1.43 | -1.63 | -1.22 |

| | | | | | |
|---|---|---|---|---|---|
| Female | Eastern Europe | Chronic myeloid leukemia | -0.63 | -0.93 | -0.32 |
| Female | Eastern Europe | Leukemia | -0.09 | -0.22 | 0.04 |
| Female | Eastern Europe | Other leukemia | 0.13 | -0.08 | 0.34 |
| Both | Eastern Europe | Acute lymphoid leukemia | -1.60 | -1.71 | -1.50 |
| Both | Eastern Europe | Chronic lymphoid leukemia | 2.50 | 2.22 | 2.78 |
| Both | Eastern Europe | Acute myeloid leukemia | -0.59 | -0.75 | -0.43 |
| Both | Eastern Europe | Chronic myeloid leukemia | -0.55 | -0.86 | -0.25 |
| Both | Eastern Europe | Leukemia | 0.06 | -0.08 | 0.21 |
| Both | Eastern Europe | Other leukemia | -0.04 | -0.25 | 0.17 |
| Male | Eastern Sub-Saharan Africa | Acute lymphoid leukemia | -0.31 | -0.49 | -0.14 |
| Male | Eastern Sub-Saharan Africa | Chronic lymphoid leukemia | 2.04 | 1.98 | 2.10 |
| Male | Eastern Sub-Saharan Africa | Acute myeloid leukemia | 0.89 | 0.81 | 0.97 |
| Male | Eastern Sub-Saharan Africa | Chronic myeloid leukemia | -0.49 | -0.58 | -0.40 |
| Male | Eastern Sub-Saharan Africa | Leukemia | 0.20 | 0.11 | 0.28 |
| Male | Eastern Sub-Saharan Africa | Other leukemia | -0.71 | -0.79 | -0.64 |
| Female | Eastern Sub-Saharan Africa | Acute lymphoid leukemia | 0.88 | 0.77 | 1.00 |
| Female | Eastern Sub-Saharan Africa | Chronic lymphoid leukemia | -0.09 | -0.16 | -0.02 |
| Female | Eastern Sub-Saharan Africa | Acute myeloid leukemia | 0.24 | 0.12 | 0.36 |
| Female | Eastern Sub-Saharan Africa | Chronic myeloid leukemia | -2.15 | -2.27 | -2.02 |
| Female | Eastern Sub-Saharan Africa | Leukemia | -0.82 | -0.92 | -0.73 |
| Female | Eastern Sub-Saharan Africa | Other leukemia | -1.51 | -1.60 | -1.42 |
| Both | Eastern Sub-Saharan Africa | Leukemia | -0.29 | -0.38 | -0.20 |
| Both | Eastern Sub-Saharan Africa | Acute lymphoid leukemia | 0.15 | 0.00 | 0.31 |
| Both | Eastern Sub-Saharan Africa | Chronic lymphoid leukemia | 0.94 | 0.89 | 0.98 |
| Both | Eastern Sub-Saharan Africa | Acute myeloid leukemia | 0.62 | 0.53 | 0.71 |
| Both | Eastern Sub-Saharan Africa | Chronic myeloid leukemia | -1.41 | -1.51 | -1.31 |
| Both | Eastern Sub-Saharan Africa | Other leukemia | -1.13 | -1.21 | -1.05 |
| Male | Ecuador | Acute lymphoid leukemia | 3.33 | 2.92 | 3.74 |
| Male | Ecuador | Chronic lymphoid leukemia | 3.12 | 2.74 | 3.50 |
| Male | Ecuador | Acute myeloid leukemia | 3.21 | 2.86 | 3.56 |
| Male | Ecuador | Chronic myeloid leukemia | 2.12 | 1.76 | 2.48 |
| Male | Ecuador | Leukemia | 1.36 | 1.10 | 1.62 |
| Male | Ecuador | Other leukemia | -0.94 | -1.30 | -0.58 |
| Female | Ecuador | Acute lymphoid leukemia | 3.13 | 2.87 | 3.39 |
| Female | Ecuador | Chronic lymphoid leukemia | 2.97 | 2.28 | 3.66 |
| Female | Ecuador | Acute myeloid leukemia | 3.44 | 2.98 | 3.90 |
| Female | Ecuador | Chronic myeloid leukemia | 2.78 | 2.36 | 3.20 |
| Female | Ecuador | Leukemia | 1.22 | 0.99 | 1.46 |
| Female | Ecuador | Other leukemia | -1.23 | -1.63 | -0.84 |
| Both | Ecuador | Acute lymphoid leukemia | 3.25 | 2.92 | 3.57 |
| Both | Ecuador | Chronic lymphoid leukemia | 3.04 | 2.67 | 3.42 |
| Both | Ecuador | Acute myeloid leukemia | 3.31 | 2.94 | 3.69 |
| Both | Ecuador | Chronic myeloid leukemia | 2.39 | 2.02 | 2.76 |
| Both | Ecuador | Leukemia | 1.30 | 1.06 | 1.53 |
| Both | Ecuador | Other leukemia | -1.08 | -1.45 | -0.72 |
| Male | Egypt | Acute lymphoid leukemia | 0.14 | -0.03 | 0.32 |
| Male | Egypt | Chronic lymphoid leukemia | 1.21 | 1.08 | 1.34 |
| Male | Egypt | Acute myeloid leukemia | -0.14 | -0.35 | 0.07 |

| | | | | | |
|---|---|---|---|---|---|
| Male | Egypt | Chronic myeloid leukemia | 0.45 | 0.25 | 0.66 |
| Male | Egypt | Leukemia | 0.10 | -0.04 | 0.24 |
| Male | Egypt | Other leukemia | 0.07 | -0.07 | 0.22 |
| Female | Egypt | Acute lymphoid leukemia | 0.17 | 0.07 | 0.27 |
| Female | Egypt | Chronic lymphoid leukemia | 2.10 | 2.01 | 2.19 |
| Female | Egypt | Acute myeloid leukemia | 0.09 | 0.04 | 0.15 |
| Female | Egypt | Chronic myeloid leukemia | -1.02 | -1.10 | -0.93 |
| Female | Egypt | Leukemia | -0.35 | -0.40 | -0.29 |
| Female | Egypt | Other leukemia | -0.47 | -0.53 | -0.40 |
| Both | Egypt | Acute lymphoid leukemia | 0.17 | 0.03 | 0.30 |
| Both | Egypt | Chronic lymphoid leukemia | 1.51 | 1.40 | 1.62 |
| Both | Egypt | Acute myeloid leukemia | -0.04 | -0.17 | 0.09 |
| Both | Egypt | Chronic myeloid leukemia | -0.08 | -0.22 | 0.06 |
| Both | Egypt | Leukemia | -0.07 | -0.16 | 0.02 |
| Both | Egypt | Other leukemia | -0.14 | -0.23 | -0.05 |
| Male | El Salvador | Acute lymphoid leukemia | 5.35 | 4.52 | 6.18 |
| Male | El Salvador | Chronic lymphoid leukemia | 5.52 | 4.98 | 6.07 |
| Male | El Salvador | Acute myeloid leukemia | 3.61 | 3.04 | 4.19 |
| Male | El Salvador | Chronic myeloid leukemia | 2.82 | 2.24 | 3.40 |
| Male | El Salvador | Leukemia | 0.30 | 0.17 | 0.43 |
| Male | El Salvador | Other leukemia | -1.38 | -1.73 | -1.04 |
| Female | El Salvador | Acute lymphoid leukemia | 5.08 | 4.18 | 5.99 |
| Female | El Salvador | Chronic lymphoid leukemia | 3.30 | 2.61 | 4.00 |
| Female | El Salvador | Acute myeloid leukemia | 2.81 | 2.14 | 3.49 |
| Female | El Salvador | Chronic myeloid leukemia | 2.75 | 2.25 | 3.25 |
| Female | El Salvador | Leukemia | -0.63 | -0.74 | -0.53 |
| Female | El Salvador | Other leukemia | -2.11 | -2.37 | -1.86 |
| Both | El Salvador | Acute lymphoid leukemia | 5.20 | 4.34 | 6.07 |
| Both | El Salvador | Chronic lymphoid leukemia | 4.34 | 3.81 | 4.87 |
| Both | El Salvador | Acute myeloid leukemia | 3.21 | 2.59 | 3.83 |
| Both | El Salvador | Chronic myeloid leukemia | 2.71 | 2.20 | 3.22 |
| Both | El Salvador | Leukemia | -0.17 | -0.27 | -0.06 |
| Both | El Salvador | Other leukemia | -1.76 | -2.06 | -1.46 |
| Male | Equatorial Guinea | Acute lymphoid leukemia | -1.75 | -2.07 | -1.42 |
| Male | Equatorial Guinea | Chronic lymphoid leukemia | 3.09 | 2.87 | 3.31 |
| Male | Equatorial Guinea | Acute myeloid leukemia | -0.22 | -0.34 | -0.09 |
| Male | Equatorial Guinea | Chronic myeloid leukemia | -1.46 | -1.87 | -1.04 |
| Male | Equatorial Guinea | Leukemia | -1.34 | -1.58 | -1.09 |
| Male | Equatorial Guinea | Other leukemia | -3.53 | -3.91 | -3.14 |
| Female | Equatorial Guinea | Acute lymphoid leukemia | 0.44 | 0.20 | 0.68 |
| Female | Equatorial Guinea | Chronic lymphoid leukemia | 2.34 | 2.17 | 2.51 |
| Female | Equatorial Guinea | Acute myeloid leukemia | 1.44 | 1.29 | 1.59 |
| Female | Equatorial Guinea | Chronic myeloid leukemia | -2.12 | -2.32 | -1.91 |
| Female | Equatorial Guinea | Leukemia | -1.72 | -1.93 | -1.51 |
| Female | Equatorial Guinea | Other leukemia | -3.47 | -3.71 | -3.22 |
| Both | Equatorial Guinea | Acute lymphoid leukemia | -0.82 | -1.10 | -0.54 |
| Both | Equatorial Guinea | Chronic lymphoid leukemia | 2.64 | 2.46 | 2.82 |
| Both | Equatorial Guinea | Acute myeloid leukemia | 0.19 | 0.09 | 0.29 |

| | | | | | |
|---|---|---|---|---|---|
| Both | Equatorial Guinea | Chronic myeloid leukemia | -1.91 | -2.16 | -1.65 |
| Both | Equatorial Guinea | Leukemia | -1.53 | -1.76 | -1.30 |
| Both | Equatorial Guinea | Other leukemia | -3.50 | -3.79 | -3.21 |
| Male | Eritrea | Acute lymphoid leukemia | 0.16 | -0.19 | 0.51 |
| Male | Eritrea | Chronic lymphoid leukemia | 2.57 | 2.13 | 3.02 |
| Male | Eritrea | Acute myeloid leukemia | 0.91 | 0.22 | 1.60 |
| Male | Eritrea | Chronic myeloid leukemia | -0.51 | -0.78 | -0.25 |
| Male | Eritrea | Leukemia | 0.23 | -0.22 | 0.68 |
| Male | Eritrea | Other leukemia | -0.94 | -1.23 | -0.65 |
| Female | Eritrea | Acute lymphoid leukemia | 1.63 | 1.42 | 1.84 |
| Female | Eritrea | Chronic lymphoid leukemia | 1.26 | 1.17 | 1.35 |
| Female | Eritrea | Acute myeloid leukemia | 1.41 | 1.24 | 1.57 |
| Female | Eritrea | Chronic myeloid leukemia | -0.27 | -0.38 | -0.16 |
| Female | Eritrea | Leukemia | 0.14 | 0.04 | 0.24 |
| Female | Eritrea | Other leukemia | -0.90 | -1.01 | -0.78 |
| Both | Eritrea | Acute lymphoid leukemia | 0.69 | 0.40 | 0.97 |
| Both | Eritrea | Chronic lymphoid leukemia | 1.74 | 1.52 | 1.95 |
| Both | Eritrea | Acute myeloid leukemia | 0.92 | 0.35 | 1.49 |
| Both | Eritrea | Chronic myeloid leukemia | -0.33 | -0.46 | -0.19 |
| Both | Eritrea | Leukemia | 0.17 | -0.12 | 0.46 |
| Both | Eritrea | Other leukemia | -0.88 | -1.06 | -0.69 |
| Male | Estonia | Acute lymphoid leukemia | -2.13 | -2.43 | -1.83 |
| Male | Estonia | Chronic lymphoid leukemia | 2.19 | 1.60 | 2.78 |
| Male | Estonia | Acute myeloid leukemia | 0.59 | 0.36 | 0.81 |
| Male | Estonia | Chronic myeloid leukemia | -1.15 | -1.48 | -0.83 |
| Male | Estonia | Leukemia | 1.13 | 0.81 | 1.45 |
| Male | Estonia | Other leukemia | 3.51 | 2.28 | 4.76 |
| Female | Estonia | Acute lymphoid leukemia | -1.28 | -1.54 | -1.03 |
| Female | Estonia | Chronic lymphoid leukemia | 2.05 | 1.70 | 2.40 |
| Female | Estonia | Acute myeloid leukemia | 0.50 | 0.34 | 0.66 |
| Female | Estonia | Chronic myeloid leukemia | -1.19 | -1.49 | -0.89 |
| Female | Estonia | Leukemia | 0.74 | 0.58 | 0.90 |
| Female | Estonia | Other leukemia | 1.30 | 0.43 | 2.18 |
| Both | Estonia | Acute lymphoid leukemia | -1.78 | -2.04 | -1.53 |
| Both | Estonia | Chronic lymphoid leukemia | 2.24 | 1.78 | 2.70 |
| Both | Estonia | Acute myeloid leukemia | 0.62 | 0.49 | 0.76 |
| Both | Estonia | Chronic myeloid leukemia | -1.04 | -1.30 | -0.78 |
| Both | Estonia | Leukemia | 0.99 | 0.76 | 1.22 |
| Both | Estonia | Other leukemia | 2.43 | 1.38 | 3.50 |
| Male | Ethiopia | Acute lymphoid leukemia | -0.87 | -1.03 | -0.71 |
| Male | Ethiopia | Chronic lymphoid leukemia | 1.11 | 1.00 | 1.23 |
| Male | Ethiopia | Acute myeloid leukemia | 1.37 | 1.29 | 1.45 |
| Male | Ethiopia | Chronic myeloid leukemia | -1.07 | -1.14 | -1.00 |
| Male | Ethiopia | Leukemia | -0.21 | -0.30 | -0.12 |
| Male | Ethiopia | Other leukemia | -1.11 | -1.19 | -1.02 |
| Female | Ethiopia | Acute lymphoid leukemia | 0.45 | 0.36 | 0.54 |
| Female | Ethiopia | Chronic lymphoid leukemia | -0.74 | -0.81 | -0.66 |
| Female | Ethiopia | Acute myeloid leukemia | -0.13 | -0.23 | -0.03 |

| | | | | | |
|---|---|---|---|---|---|
| Female | Ethiopia | Chronic myeloid leukemia | -2.50 | -2.65 | -2.35 |
| Female | Ethiopia | Leukemia | -1.34 | -1.42 | -1.26 |
| Female | Ethiopia | Other leukemia | -2.39 | -2.53 | -2.24 |
| Both | Ethiopia | Acute lymphoid leukemia | -0.33 | -0.45 | -0.20 |
| Both | Ethiopia | Chronic lymphoid leukemia | 0.45 | 0.37 | 0.52 |
| Both | Ethiopia | Acute myeloid leukemia | 0.65 | 0.57 | 0.74 |
| Both | Ethiopia | Chronic myeloid leukemia | -1.81 | -1.90 | -1.72 |
| Both | Ethiopia | Leukemia | -0.73 | -0.80 | -0.66 |
| Both | Ethiopia | Other leukemia | -1.71 | -1.80 | -1.61 |
| Male | Federated States of Micronesia | Acute lymphoid leukemia | -0.35 | -0.41 | -0.29 |
| Male | Federated States of Micronesia | Chronic lymphoid leukemia | -0.68 | -0.79 | -0.58 |
| Male | Federated States of Micronesia | Acute myeloid leukemia | 0.34 | 0.28 | 0.40 |
| Male | Federated States of Micronesia | Chronic myeloid leukemia | -1.35 | -1.45 | -1.24 |
| Male | Federated States of Micronesia | Leukemia | -0.24 | -0.27 | -0.21 |
| Male | Federated States of Micronesia | Other leukemia | -0.42 | -0.46 | -0.39 |
| Female | Federated States of Micronesia | Acute lymphoid leukemia | -0.08 | -0.15 | -0.01 |
| Female | Federated States of Micronesia | Chronic lymphoid leukemia | 0.38 | 0.32 | 0.44 |
| Female | Federated States of Micronesia | Acute myeloid leukemia | -0.78 | -0.87 | -0.69 |
| Female | Federated States of Micronesia | Chronic myeloid leukemia | -1.92 | -2.07 | -1.78 |
| Female | Federated States of Micronesia | Leukemia | -0.83 | -0.91 | -0.76 |
| Female | Federated States of Micronesia | Other leukemia | -1.15 | -1.23 | -1.07 |
| Both | Federated States of Micronesia | Acute lymphoid leukemia | -0.23 | -0.28 | -0.18 |
| Both | Federated States of Micronesia | Chronic lymphoid leukemia | 0.45 | 0.40 | 0.49 |
| Both | Federated States of Micronesia | Acute myeloid leukemia | -0.12 | -0.17 | -0.06 |
| Both | Federated States of Micronesia | Chronic myeloid leukemia | -1.65 | -1.77 | -1.53 |
| Both | Federated States of Micronesia | Leukemia | -0.53 | -0.58 | -0.48 |
| Both | Federated States of Micronesia | Other leukemia | -0.78 | -0.84 | -0.72 |
| Male | Fiji | Acute lymphoid leukemia | -1.66 | -1.94 | -1.38 |
| Male | Fiji | Chronic lymphoid leukemia | 0.51 | 0.36 | 0.66 |
| Male | Fiji | Acute myeloid leukemia | 0.95 | 0.77 | 1.13 |
| Male | Fiji | Chronic myeloid leukemia | -2.39 | -2.71 | -2.07 |
| Male | Fiji | Leukemia | 0.13 | 0.06 | 0.20 |
| Male | Fiji | Other leukemia | -0.51 | -0.67 | -0.35 |
| Female | Fiji | Acute lymphoid leukemia | -1.48 | -1.69 | -1.27 |
| Female | Fiji | Chronic lymphoid leukemia | -0.19 | -0.36 | -0.03 |
| Female | Fiji | Acute myeloid leukemia | 0.51 | 0.07 | 0.95 |
| Female | Fiji | Chronic myeloid leukemia | -2.40 | -2.80 | -2.00 |
| Female | Fiji | Leukemia | -0.75 | -0.89 | -0.62 |
| Female | Fiji | Other leukemia | -2.02 | -2.41 | -1.64 |
| Both | Fiji | Acute lymphoid leukemia | -1.57 | -1.81 | -1.33 |
| Both | Fiji | Chronic lymphoid leukemia | 0.03 | -0.12 | 0.19 |
| Both | Fiji | Acute myeloid leukemia | 0.70 | 0.44 | 0.96 |
| Both | Fiji | Chronic myeloid leukemia | -2.38 | -2.60 | -2.15 |
| Both | Fiji | Leukemia | -0.31 | -0.40 | -0.21 |
| Both | Fiji | Other leukemia | -1.26 | -1.51 | -1.01 |
| Male | Finland | Acute lymphoid leukemia | -2.37 | -2.71 | -2.04 |
| Male | Finland | Chronic lymphoid leukemia | -0.05 | -0.47 | 0.36 |
| Male | Finland | Acute myeloid leukemia | -1.07 | -1.32 | -0.83 |

| | | | | | |
|---|---|---|---|---|---|
| Male | Finland | Chronic myeloid leukemia | -3.51 | -3.91 | -3.10 |
| Male | Finland | Leukemia | -1.16 | -1.42 | -0.91 |
| Male | Finland | Other leukemia | -1.51 | -1.74 | -1.28 |
| Female | Finland | Acute lymphoid leukemia | -2.11 | -2.47 | -1.75 |
| Female | Finland | Chronic lymphoid leukemia | 0.28 | -0.07 | 0.63 |
| Female | Finland | Acute myeloid leukemia | -0.04 | -0.45 | 0.37 |
| Female | Finland | Chronic myeloid leukemia | -4.26 | -4.65 | -3.86 |
| Female | Finland | Leukemia | -0.85 | -1.10 | -0.60 |
| Female | Finland | Other leukemia | -1.88 | -2.24 | -1.51 |
| Both | Finland | Acute lymphoid leukemia | -2.23 | -2.57 | -1.90 |
| Both | Finland | Chronic lymphoid leukemia | 0.28 | -0.11 | 0.67 |
| Both | Finland | Acute myeloid leukemia | -0.48 | -0.80 | -0.16 |
| Both | Finland | Chronic myeloid leukemia | -3.68 | -4.07 | -3.28 |
| Both | Finland | Leukemia | -0.91 | -1.16 | -0.66 |
| Both | Finland | Other leukemia | -1.56 | -1.84 | -1.27 |
| Male | France | Acute lymphoid leukemia | -2.53 | -2.73 | -2.33 |
| Male | France | Chronic lymphoid leukemia | 0.28 | 0.03 | 0.53 |
| Male | France | Acute myeloid leukemia | 0.68 | 0.49 | 0.88 |
| Male | France | Chronic myeloid leukemia | -3.14 | -3.65 | -2.63 |
| Male | France | Leukemia | -0.68 | -0.84 | -0.52 |
| Male | France | Other leukemia | -1.04 | -1.19 | -0.90 |
| Female | France | Acute lymphoid leukemia | -2.17 | -2.29 | -2.05 |
| Female | France | Chronic lymphoid leukemia | 0.28 | 0.06 | 0.50 |
| Female | France | Acute myeloid leukemia | 0.93 | 0.71 | 1.14 |
| Female | France | Chronic myeloid leukemia | -4.40 | -4.90 | -3.90 |
| Female | France | Leukemia | -0.80 | -0.93 | -0.66 |
| Female | France | Other leukemia | -1.46 | -1.58 | -1.34 |
| Both | France | Acute lymphoid leukemia | -2.39 | -2.54 | -2.24 |
| Both | France | Chronic lymphoid leukemia | 0.33 | 0.08 | 0.58 |
| Both | France | Acute myeloid leukemia | 0.78 | 0.59 | 0.97 |
| Both | France | Chronic myeloid leukemia | -3.60 | -4.11 | -3.10 |
| Both | France | Leukemia | -0.70 | -0.86 | -0.55 |
| Both | France | Other leukemia | -1.15 | -1.29 | -1.01 |
| Male | Gabon | Acute lymphoid leukemia | 1.49 | 1.20 | 1.78 |
| Male | Gabon | Chronic lymphoid leukemia | 3.73 | 3.50 | 3.96 |
| Male | Gabon | Acute myeloid leukemia | 0.49 | 0.35 | 0.64 |
| Male | Gabon | Chronic myeloid leukemia | 1.71 | 1.52 | 1.89 |
| Male | Gabon | Leukemia | 0.60 | 0.46 | 0.74 |
| Male | Gabon | Other leukemia | -0.26 | -0.41 | -0.10 |
| Female | Gabon | Acute lymphoid leukemia | 0.75 | 0.58 | 0.92 |
| Female | Gabon | Chronic lymphoid leukemia | 0.82 | 0.72 | 0.93 |
| Female | Gabon | Acute myeloid leukemia | 1.01 | 0.83 | 1.20 |
| Female | Gabon | Chronic myeloid leukemia | -0.38 | -0.69 | -0.07 |
| Female | Gabon | Leukemia | -0.53 | -0.71 | -0.35 |
| Female | Gabon | Other leukemia | -1.69 | -1.92 | -1.46 |
| Both | Gabon | Acute lymphoid leukemia | 1.17 | 0.94 | 1.40 |
| Both | Gabon | Chronic lymphoid leukemia | 2.09 | 1.98 | 2.21 |
| Both | Gabon | Acute myeloid leukemia | 0.69 | 0.54 | 0.84 |

| | | | | | |
|---|---|---|---|---|---|
| Both | Gabon | Chronic myeloid leukemia | 0.47 | 0.26 | 0.68 |
| Both | Gabon | Leukemia | 0.15 | 0.01 | 0.30 |
| Both | Gabon | Other leukemia | -1.05 | -1.22 | -0.87 |
| Male | Gambia | Acute lymphoid leukemia | -0.62 | -0.70 | -0.55 |
| Male | Gambia | Chronic lymphoid leukemia | 2.16 | 1.97 | 2.35 |
| Male | Gambia | Acute myeloid leukemia | 1.06 | 0.95 | 1.17 |
| Male | Gambia | Chronic myeloid leukemia | 0.85 | 0.72 | 0.97 |
| Male | Gambia | Leukemia | 0.73 | 0.63 | 0.82 |
| Male | Gambia | Other leukemia | 0.55 | 0.44 | 0.66 |
| Female | Gambia | Acute lymphoid leukemia | 0.98 | 0.83 | 1.14 |
| Female | Gambia | Chronic lymphoid leukemia | 0.59 | 0.54 | 0.64 |
| Female | Gambia | Acute myeloid leukemia | 1.03 | 0.92 | 1.14 |
| Female | Gambia | Chronic myeloid leukemia | 0.05 | -0.02 | 0.12 |
| Female | Gambia | Leukemia | 0.32 | 0.25 | 0.39 |
| Female | Gambia | Other leukemia | -0.45 | -0.51 | -0.39 |
| Both | Gambia | Acute lymphoid leukemia | -0.24 | -0.34 | -0.14 |
| Both | Gambia | Chronic lymphoid leukemia | 1.33 | 1.24 | 1.42 |
| Both | Gambia | Acute myeloid leukemia | 0.96 | 0.87 | 1.05 |
| Both | Gambia | Chronic myeloid leukemia | 0.50 | 0.39 | 0.61 |
| Both | Gambia | Leukemia | 0.51 | 0.44 | 0.57 |
| Both | Gambia | Other leukemia | 0.06 | 0.00 | 0.11 |
| Male | Georgia | Acute lymphoid leukemia | -0.60 | -1.55 | 0.36 |
| Male | Georgia | Chronic lymphoid leukemia | 8.09 | 5.92 | 10.31 |
| Male | Georgia | Acute myeloid leukemia | 2.29 | 1.08 | 3.51 |
| Male | Georgia | Chronic myeloid leukemia | 1.15 | 0.28 | 2.03 |
| Male | Georgia | Leukemia | 1.57 | 1.06 | 2.07 |
| Male | Georgia | Other leukemia | 1.30 | 0.38 | 2.23 |
| Female | Georgia | Acute lymphoid leukemia | -0.33 | -0.89 | 0.24 |
| Female | Georgia | Chronic lymphoid leukemia | 1.28 | 0.64 | 1.92 |
| Female | Georgia | Acute myeloid leukemia | 1.79 | 0.63 | 2.98 |
| Female | Georgia | Chronic myeloid leukemia | 1.68 | 0.51 | 2.87 |
| Female | Georgia | Leukemia | 0.91 | 0.56 | 1.26 |
| Female | Georgia | Other leukemia | 0.71 | -0.11 | 1.53 |
| Both | Georgia | Acute lymphoid leukemia | -0.50 | -1.26 | 0.27 |
| Both | Georgia | Chronic lymphoid leukemia | 4.19 | 2.90 | 5.50 |
| Both | Georgia | Acute myeloid leukemia | 2.07 | 0.90 | 3.25 |
| Both | Georgia | Chronic myeloid leukemia | 1.42 | 0.44 | 2.41 |
| Both | Georgia | Leukemia | 1.26 | 0.86 | 1.66 |
| Both | Georgia | Other leukemia | 1.03 | 0.17 | 1.89 |
| Male | Germany | Acute lymphoid leukemia | -2.23 | -2.54 | -1.91 |
| Male | Germany | Chronic lymphoid leukemia | -1.14 | -1.31 | -0.97 |
| Male | Germany | Acute myeloid leukemia | -0.82 | -1.08 | -0.56 |
| Male | Germany | Chronic myeloid leukemia | -5.28 | -5.57 | -4.99 |
| Male | Germany | Leukemia | -2.03 | -2.18 | -1.87 |
| Male | Germany | Other leukemia | -2.47 | -2.61 | -2.33 |
| Female | Germany | Acute lymphoid leukemia | -2.04 | -2.34 | -1.73 |
| Female | Germany | Chronic lymphoid leukemia | -0.44 | -0.61 | -0.28 |
| Female | Germany | Acute myeloid leukemia | -0.31 | -0.54 | -0.08 |

| | | | | | |
|---|---|---|---|---|---|
| Female | Germany | Chronic myeloid leukemia | -5.39 | -5.67 | -5.11 |
| Female | Germany | Leukemia | -1.58 | -1.73 | -1.44 |
| Female | Germany | Other leukemia | -2.29 | -2.46 | -2.11 |
| Both | Germany | Acute lymphoid leukemia | -2.13 | -2.44 | -1.82 |
| Both | Germany | Chronic lymphoid leukemia | -0.70 | -0.87 | -0.53 |
| Both | Germany | Acute myeloid leukemia | -0.51 | -0.73 | -0.29 |
| Both | Germany | Chronic myeloid leukemia | -5.23 | -5.52 | -4.95 |
| Both | Germany | Leukemia | -1.72 | -1.88 | -1.56 |
| Both | Germany | Other leukemia | -2.24 | -2.40 | -2.07 |
| Male | Ghana | Acute lymphoid leukemia | -7.08 | -8.45 | -5.68 |
| Male | Ghana | Chronic lymphoid leukemia | 1.94 | 1.69 | 2.19 |
| Male | Ghana | Acute myeloid leukemia | -0.95 | -1.39 | -0.50 |
| Male | Ghana | Chronic myeloid leukemia | -0.22 | -0.34 | -0.11 |
| Male | Ghana | Leukemia | -1.70 | -2.13 | -1.27 |
| Male | Ghana | Other leukemia | -0.85 | -1.06 | -0.65 |
| Female | Ghana | Acute lymphoid leukemia | -1.51 | -2.00 | -1.02 |
| Female | Ghana | Chronic lymphoid leukemia | -0.34 | -0.45 | -0.23 |
| Female | Ghana | Acute myeloid leukemia | -0.79 | -1.04 | -0.53 |
| Female | Ghana | Chronic myeloid leukemia | -1.96 | -2.20 | -1.72 |
| Female | Ghana | Leukemia | -1.54 | -1.84 | -1.25 |
| Female | Ghana | Other leukemia | -3.02 | -3.43 | -2.61 |
| Both | Ghana | Acute lymphoid leukemia | -5.18 | -6.23 | -4.10 |
| Both | Ghana | Chronic lymphoid leukemia | 0.47 | 0.44 | 0.50 |
| Both | Ghana | Acute myeloid leukemia | -0.87 | -1.22 | -0.51 |
| Both | Ghana | Chronic myeloid leukemia | -1.11 | -1.26 | -0.95 |
| Both | Ghana | Leukemia | -1.70 | -2.07 | -1.33 |
| Both | Ghana | Other leukemia | -1.62 | -1.88 | -1.36 |
| Male | Global | Acute lymphoid leukemia | -0.23 | -0.30 | -0.16 |
| Male | Global | Chronic lymphoid leukemia | 0.48 | 0.42 | 0.53 |
| Male | Global | Acute myeloid leukemia | 0.72 | 0.67 | 0.77 |
| Male | Global | Chronic myeloid leukemia | -2.17 | -2.29 | -2.05 |
| Male | Global | Leukemia | -0.26 | -0.32 | -0.21 |
| Male | Global | Other leukemia | -0.66 | -0.75 | -0.57 |
| Female | Global | Acute lymphoid leukemia | 0.15 | 0.08 | 0.22 |
| Female | Global | Chronic lymphoid leukemia | 0.38 | 0.31 | 0.46 |
| Female | Global | Acute myeloid leukemia | 0.35 | 0.27 | 0.44 |
| Female | Global | Chronic myeloid leukemia | -2.70 | -2.85 | -2.55 |
| Female | Global | Leukemia | -0.65 | -0.70 | -0.60 |
| Female | Global | Other leukemia | -1.26 | -1.34 | -1.19 |
| Both | Global | Acute lymphoid leukemia | -0.08 | -0.15 | -0.02 |
| Both | Global | Chronic lymphoid leukemia | 0.46 | 0.40 | 0.52 |
| Both | Global | Acute myeloid leukemia | 0.56 | 0.49 | 0.62 |
| Both | Global | Chronic myeloid leukemia | -2.40 | -2.53 | -2.26 |
| Both | Global | Leukemia | -0.43 | -0.48 | -0.38 |
| Both | Global | Other leukemia | -0.93 | -1.02 | -0.84 |
| Male | Greece | Acute lymphoid leukemia | -0.88 | -1.00 | -0.77 |
| Male | Greece | Chronic lymphoid leukemia | 1.49 | 1.15 | 1.82 |
| Male | Greece | Acute myeloid leukemia | 1.11 | 0.94 | 1.28 |

| | | | | | |
|---|---|---|---|---|---|
| Male | Greece | Chronic myeloid leukemia | -1.72 | -2.12 | -1.33 |
| Male | Greece | Leukemia | -0.11 | -0.42 | 0.20 |
| Male | Greece | Other leukemia | -1.33 | -1.87 | -0.78 |
| Female | Greece | Acute lymphoid leukemia | -0.37 | -0.59 | -0.15 |
| Female | Greece | Chronic lymphoid leukemia | 1.96 | 1.57 | 2.34 |
| Female | Greece | Acute myeloid leukemia | 1.64 | 1.51 | 1.78 |
| Female | Greece | Chronic myeloid leukemia | -2.43 | -2.66 | -2.19 |
| Female | Greece | Leukemia | 0.29 | -0.03 | 0.60 |
| Female | Greece | Other leukemia | -0.96 | -1.65 | -0.27 |
| Both | Greece | Acute lymphoid leukemia | -0.67 | -0.80 | -0.54 |
| Both | Greece | Chronic lymphoid leukemia | 1.71 | 1.35 | 2.07 |
| Both | Greece | Acute myeloid leukemia | 1.34 | 1.23 | 1.46 |
| Both | Greece | Chronic myeloid leukemia | -1.98 | -2.30 | -1.65 |
| Both | Greece | Leukemia | 0.06 | -0.24 | 0.37 |
| Both | Greece | Other leukemia | -1.18 | -1.78 | -0.58 |
| Male | Greenland | Acute lymphoid leukemia | -3.80 | -4.12 | -3.48 |
| Male | Greenland | Chronic lymphoid leukemia | 0.74 | 0.60 | 0.88 |
| Male | Greenland | Acute myeloid leukemia | -0.85 | -1.14 | -0.57 |
| Male | Greenland | Chronic myeloid leukemia | -4.75 | -5.08 | -4.42 |
| Male | Greenland | Leukemia | -1.40 | -1.50 | -1.31 |
| Male | Greenland | Other leukemia | -1.40 | -1.85 | -0.93 |
| Female | Greenland | Acute lymphoid leukemia | -3.05 | -3.32 | -2.77 |
| Female | Greenland | Chronic lymphoid leukemia | -0.87 | -1.01 | -0.74 |
| Female | Greenland | Acute myeloid leukemia | -0.57 | -0.80 | -0.35 |
| Female | Greenland | Chronic myeloid leukemia | -5.38 | -5.63 | -5.14 |
| Female | Greenland | Leukemia | -1.68 | -1.78 | -1.58 |
| Female | Greenland | Other leukemia | -2.70 | -2.90 | -2.51 |
| Both | Greenland | Acute lymphoid leukemia | -3.45 | -3.66 | -3.25 |
| Both | Greenland | Chronic lymphoid leukemia | 0.31 | 0.20 | 0.41 |
| Both | Greenland | Acute myeloid leukemia | -0.57 | -0.72 | -0.43 |
| Both | Greenland | Chronic myeloid leukemia | -4.49 | -4.80 | -4.18 |
| Both | Greenland | Leukemia | -1.34 | -1.41 | -1.27 |
| Both | Greenland | Other leukemia | -1.97 | -2.19 | -1.75 |
| Male | Grenada | Acute lymphoid leukemia | -0.87 | -1.13 | -0.61 |
| Male | Grenada | Chronic lymphoid leukemia | 3.37 | 2.50 | 4.25 |
| Male | Grenada | Acute myeloid leukemia | -0.71 | -1.10 | -0.32 |
| Male | Grenada | Chronic myeloid leukemia | -0.19 | -0.69 | 0.32 |
| Male | Grenada | Leukemia | -0.87 | -1.09 | -0.65 |
| Male | Grenada | Other leukemia | -2.16 | -2.50 | -1.82 |
| Female | Grenada | Acute lymphoid leukemia | -3.13 | -3.84 | -2.41 |
| Female | Grenada | Chronic lymphoid leukemia | 2.93 | 2.18 | 3.68 |
| Female | Grenada | Acute myeloid leukemia | -0.39 | -0.77 | -0.02 |
| Female | Grenada | Chronic myeloid leukemia | -4.72 | -5.45 | -3.98 |
| Female | Grenada | Leukemia | -0.21 | -0.58 | 0.17 |
| Female | Grenada | Other leukemia | -0.23 | -0.79 | 0.33 |
| Both | Grenada | Acute lymphoid leukemia | -2.06 | -2.51 | -1.62 |
| Both | Grenada | Chronic lymphoid leukemia | 2.74 | 1.99 | 3.49 |
| Both | Grenada | Acute myeloid leukemia | -0.47 | -0.85 | -0.09 |

| | | | | | |
|---|---|---|---|---|---|
| Both | Grenada | Chronic myeloid leukemia | -1.34 | -1.66 | -1.03 |
| Both | Grenada | Leukemia | -0.47 | -0.75 | -0.19 |
| Both | Grenada | Other leukemia | -1.04 | -1.32 | -0.76 |
| Male | Guam | Acute lymphoid leukemia | -4.21 | -4.60 | -3.82 |
| Male | Guam | Chronic lymphoid leukemia | -3.32 | -3.84 | -2.80 |
| Male | Guam | Acute myeloid leukemia | 1.62 | 1.34 | 1.90 |
| Male | Guam | Chronic myeloid leukemia | -1.80 | -2.35 | -1.25 |
| Male | Guam | Leukemia | 0.23 | 0.08 | 0.37 |
| Male | Guam | Other leukemia | 0.22 | -0.13 | 0.57 |
| Female | Guam | Acute lymphoid leukemia | -1.15 | -1.35 | -0.95 |
| Female | Guam | Chronic lymphoid leukemia | 0.60 | 0.31 | 0.88 |
| Female | Guam | Acute myeloid leukemia | -2.32 | -2.92 | -1.72 |
| Female | Guam | Chronic myeloid leukemia | 1.71 | 0.66 | 2.77 |
| Female | Guam | Leukemia | 0.19 | -0.19 | 0.57 |
| Female | Guam | Other leukemia | 2.07 | 1.56 | 2.57 |
| Both | Guam | Acute lymphoid leukemia | -3.62 | -3.90 | -3.33 |
| Both | Guam | Chronic lymphoid leukemia | 0.23 | -0.07 | 0.53 |
| Both | Guam | Acute myeloid leukemia | 0.56 | 0.38 | 0.74 |
| Both | Guam | Chronic myeloid leukemia | -0.41 | -1.06 | 0.24 |
| Both | Guam | Leukemia | 0.26 | 0.09 | 0.43 |
| Both | Guam | Other leukemia | 0.97 | 0.67 | 1.28 |
| Male | Guatemala | Acute lymphoid leukemia | 4.44 | 4.13 | 4.75 |
| Male | Guatemala | Chronic lymphoid leukemia | 1.58 | 0.98 | 2.19 |
| Male | Guatemala | Acute myeloid leukemia | 3.31 | 3.01 | 3.62 |
| Male | Guatemala | Chronic myeloid leukemia | 1.38 | 1.04 | 1.71 |
| Male | Guatemala | Leukemia | 0.78 | 0.28 | 1.28 |
| Male | Guatemala | Other leukemia | -1.27 | -2.05 | -0.48 |
| Female | Guatemala | Acute lymphoid leukemia | 5.42 | 5.06 | 5.77 |
| Female | Guatemala | Chronic lymphoid leukemia | 2.21 | 1.64 | 2.78 |
| Female | Guatemala | Acute myeloid leukemia | 2.20 | 1.88 | 2.53 |
| Female | Guatemala | Chronic myeloid leukemia | 1.52 | 1.15 | 1.90 |
| Female | Guatemala | Leukemia | 0.60 | 0.19 | 1.00 |
| Female | Guatemala | Other leukemia | -1.38 | -2.05 | -0.71 |
| Both | Guatemala | Acute lymphoid leukemia | 4.81 | 4.50 | 5.12 |
| Both | Guatemala | Chronic lymphoid leukemia | 1.67 | 1.10 | 2.24 |
| Both | Guatemala | Acute myeloid leukemia | 2.73 | 2.44 | 3.03 |
| Both | Guatemala | Chronic myeloid leukemia | 1.33 | 1.01 | 1.65 |
| Both | Guatemala | Leukemia | 0.66 | 0.21 | 1.10 |
| Both | Guatemala | Other leukemia | -1.36 | -2.08 | -0.64 |
| Male | Guinea | Acute lymphoid leukemia | -1.42 | -1.68 | -1.16 |
| Male | Guinea | Chronic lymphoid leukemia | 1.43 | 1.32 | 1.53 |
| Male | Guinea | Acute myeloid leukemia | 0.27 | 0.15 | 0.39 |
| Male | Guinea | Chronic myeloid leukemia | 1.44 | 1.33 | 1.54 |
| Male | Guinea | Leukemia | 0.35 | 0.26 | 0.45 |
| Male | Guinea | Other leukemia | 0.58 | 0.51 | 0.65 |
| Female | Guinea | Acute lymphoid leukemia | -0.22 | -0.35 | -0.10 |
| Female | Guinea | Chronic lymphoid leukemia | 0.03 | 0.00 | 0.06 |
| Female | Guinea | Acute myeloid leukemia | -0.09 | -0.16 | -0.01 |

| | | | | | |
|---|---|---|---|---|---|
| Female | Guinea | Chronic myeloid leukemia | -0.45 | -0.51 | -0.40 |
| Female | Guinea | Leukemia | -0.55 | -0.62 | -0.48 |
| Female | Guinea | Other leukemia | -1.27 | -1.35 | -1.19 |
| Both | Guinea | Acute lymphoid leukemia | -1.07 | -1.30 | -0.83 |
| Both | Guinea | Chronic lymphoid leukemia | 0.56 | 0.52 | 0.59 |
| Both | Guinea | Acute myeloid leukemia | 0.15 | 0.05 | 0.24 |
| Both | Guinea | Chronic myeloid leukemia | 0.49 | 0.42 | 0.56 |
| Both | Guinea | Leukemia | -0.01 | -0.10 | 0.09 |
| Both | Guinea | Other leukemia | -0.15 | -0.22 | -0.08 |
| Male | Guinea-Bissau | Acute lymphoid leukemia | -1.53 | -1.64 | -1.42 |
| Male | Guinea-Bissau | Chronic lymphoid leukemia | 1.01 | 0.97 | 1.04 |
| Male | Guinea-Bissau | Acute myeloid leukemia | 0.64 | 0.55 | 0.72 |
| Male | Guinea-Bissau | Chronic myeloid leukemia | -0.11 | -0.16 | -0.06 |
| Male | Guinea-Bissau | Leukemia | 0.15 | 0.09 | 0.21 |
| Male | Guinea-Bissau | Other leukemia | 0.34 | 0.22 | 0.46 |
| Female | Guinea-Bissau | Acute lymphoid leukemia | 1.07 | 0.90 | 1.24 |
| Female | Guinea-Bissau | Chronic lymphoid leukemia | 0.35 | 0.31 | 0.38 |
| Female | Guinea-Bissau | Acute myeloid leukemia | 0.92 | 0.79 | 1.05 |
| Female | Guinea-Bissau | Chronic myeloid leukemia | -0.55 | -0.59 | -0.50 |
| Female | Guinea-Bissau | Leukemia | 0.15 | 0.06 | 0.24 |
| Female | Guinea-Bissau | Other leukemia | -0.32 | -0.42 | -0.21 |
| Both | Guinea-Bissau | Acute lymphoid leukemia | -0.67 | -0.79 | -0.55 |
| Both | Guinea-Bissau | Chronic lymphoid leukemia | 0.64 | 0.61 | 0.67 |
| Both | Guinea-Bissau | Acute myeloid leukemia | 0.79 | 0.69 | 0.89 |
| Both | Guinea-Bissau | Chronic myeloid leukemia | -0.49 | -0.52 | -0.45 |
| Both | Guinea-Bissau | Leukemia | 0.13 | 0.06 | 0.20 |
| Both | Guinea-Bissau | Other leukemia | 0.05 | -0.06 | 0.16 |
| Male | Guyana | Acute lymphoid leukemia | 1.24 | 0.94 | 1.54 |
| Male | Guyana | Chronic lymphoid leukemia | 2.93 | 2.27 | 3.59 |
| Male | Guyana | Acute myeloid leukemia | -2.38 | -2.86 | -1.91 |
| Male | Guyana | Chronic myeloid leukemia | -0.21 | -0.58 | 0.17 |
| Male | Guyana | Leukemia | 0.19 | 0.00 | 0.38 |
| Male | Guyana | Other leukemia | -0.11 | -0.36 | 0.13 |
| Female | Guyana | Acute lymphoid leukemia | 1.26 | 0.94 | 1.59 |
| Female | Guyana | Chronic lymphoid leukemia | 4.38 | 3.43 | 5.34 |
| Female | Guyana | Acute myeloid leukemia | 0.02 | -0.28 | 0.32 |
| Female | Guyana | Chronic myeloid leukemia | 0.78 | 0.24 | 1.33 |
| Female | Guyana | Leukemia | 0.06 | -0.19 | 0.30 |
| Female | Guyana | Other leukemia | -1.06 | -1.33 | -0.79 |
| Both | Guyana | Acute lymphoid leukemia | 1.23 | 0.99 | 1.47 |
| Both | Guyana | Chronic lymphoid leukemia | 3.69 | 2.89 | 4.49 |
| Both | Guyana | Acute myeloid leukemia | -1.29 | -1.68 | -0.89 |
| Both | Guyana | Chronic myeloid leukemia | 0.15 | -0.21 | 0.51 |
| Both | Guyana | Leukemia | 0.12 | -0.07 | 0.31 |
| Both | Guyana | Other leukemia | -0.68 | -0.89 | -0.47 |
| Male | Haiti | Acute lymphoid leukemia | -0.63 | -0.69 | -0.58 |
| Male | Haiti | Chronic lymphoid leukemia | 1.13 | 1.03 | 1.23 |
| Male | Haiti | Acute myeloid leukemia | 0.49 | 0.32 | 0.66 |

| | | | | | |
|---|---|---|---|---|---|
| Male | Haiti | Chronic myeloid leukemia | -0.33 | -0.37 | -0.29 |
| Male | Haiti | Leukemia | -0.28 | -0.32 | -0.25 |
| Male | Haiti | Other leukemia | -0.58 | -0.64 | -0.52 |
| Female | Haiti | Acute lymphoid leukemia | -0.58 | -0.60 | -0.55 |
| Female | Haiti | Chronic lymphoid leukemia | 0.52 | 0.46 | 0.57 |
| Female | Haiti | Acute myeloid leukemia | -0.39 | -0.42 | -0.35 |
| Female | Haiti | Chronic myeloid leukemia | -1.53 | -1.59 | -1.47 |
| Female | Haiti | Leukemia | -1.05 | -1.08 | -1.01 |
| Female | Haiti | Other leukemia | -1.37 | -1.42 | -1.31 |
| Both | Haiti | Acute lymphoid leukemia | -0.62 | -0.66 | -0.58 |
| Both | Haiti | Chronic lymphoid leukemia | 0.89 | 0.79 | 0.98 |
| Both | Haiti | Acute myeloid leukemia | -0.01 | -0.08 | 0.07 |
| Both | Haiti | Chronic myeloid leukemia | -0.97 | -1.01 | -0.93 |
| Both | Haiti | Leukemia | -0.66 | -0.69 | -0.63 |
| Both | Haiti | Other leukemia | -0.97 | -1.02 | -0.92 |
| Male | High SDI | Acute lymphoid leukemia | -1.94 | -2.01 | -1.88 |
| Male | High SDI | Chronic lymphoid leukemia | -0.12 | -0.33 | 0.08 |
| Male | High SDI | Acute myeloid leukemia | 0.61 | 0.48 | 0.74 |
| Male | High SDI | Chronic myeloid leukemia | -3.72 | -3.93 | -3.51 |
| Male | High SDI | Leukemia | -0.68 | -0.76 | -0.59 |
| Male | High SDI | Other leukemia | -0.82 | -0.87 | -0.77 |
| Female | High SDI | Acute lymphoid leukemia | -1.83 | -1.87 | -1.78 |
| Female | High SDI | Chronic lymphoid leukemia | 0.05 | -0.13 | 0.24 |
| Female | High SDI | Acute myeloid leukemia | 0.53 | 0.37 | 0.70 |
| Female | High SDI | Chronic myeloid leukemia | -4.50 | -4.76 | -4.24 |
| Female | High SDI | Leukemia | -0.77 | -0.82 | -0.71 |
| Female | High SDI | Other leukemia | -1.18 | -1.23 | -1.12 |
| Both | High SDI | Acute lymphoid leukemia | -1.88 | -1.94 | -1.83 |
| Both | High SDI | Chronic lymphoid leukemia | 0.02 | -0.18 | 0.22 |
| Both | High SDI | Acute myeloid leukemia | 0.60 | 0.45 | 0.74 |
| Both | High SDI | Chronic myeloid leukemia | -4.00 | -4.23 | -3.78 |
| Both | High SDI | Leukemia | -0.67 | -0.74 | -0.60 |
| Both | High SDI | Other leukemia | -0.92 | -0.97 | -0.87 |
| Male | High-income Asia Pacific | Acute lymphoid leukemia | -2.08 | -2.21 | -1.95 |
| Male | High-income Asia Pacific | Chronic lymphoid leukemia | 2.18 | 1.96 | 2.39 |
| Male | High-income Asia Pacific | Acute myeloid leukemia | 0.81 | 0.52 | 1.10 |
| Male | High-income Asia Pacific | Chronic myeloid leukemia | -2.93 | -3.10 | -2.76 |
| Male | High-income Asia Pacific | Leukemia | -0.38 | -0.47 | -0.29 |
| Male | High-income Asia Pacific | Other leukemia | -0.58 | -0.76 | -0.40 |
| Female | High-income Asia Pacific | Acute lymphoid leukemia | -2.52 | -2.62 | -2.43 |
| Female | High-income Asia Pacific | Chronic lymphoid leukemia | 2.57 | 2.29 | 2.85 |
| Female | High-income Asia Pacific | Acute myeloid leukemia | 0.68 | 0.39 | 0.97 |
| Female | High-income Asia Pacific | Chronic myeloid leukemia | -3.71 | -3.92 | -3.49 |
| Female | High-income Asia Pacific | Leukemia | -0.59 | -0.72 | -0.45 |
| Female | High-income Asia Pacific | Other leukemia | -0.93 | -1.16 | -0.70 |
| Both | High-income Asia Pacific | Acute lymphoid leukemia | -2.27 | -2.39 | -2.16 |
| Both | High-income Asia Pacific | Chronic lymphoid leukemia | 2.40 | 2.22 | 2.58 |
| Both | High-income Asia Pacific | Acute myeloid leukemia | 0.74 | 0.48 | 1.01 |

| | | | | | |
|---|---|---|---|---|---|
| Both | High-income Asia Pacific | Chronic myeloid leukemia | -3.20 | -3.37 | -3.02 |
| Both | High-income Asia Pacific | Other leukemia | -0.74 | -0.95 | -0.54 |
| Both | High-income Asia Pacific | Leukemia | -0.47 | -0.57 | -0.38 |
| Male | High-income North America | Acute lymphoid leukemia | -1.52 | -1.62 | -1.41 |
| Male | High-income North America | Chronic lymphoid leukemia | -0.34 | -0.62 | -0.05 |
| Male | High-income North America | Acute myeloid leukemia | 1.01 | 0.76 | 1.27 |
| Male | High-income North America | Chronic myeloid leukemia | -4.32 | -4.68 | -3.96 |
| Male | High-income North America | Leukemia | -0.67 | -0.86 | -0.48 |
| Male | High-income North America | Other leukemia | -1.14 | -1.39 | -0.89 |
| Female | High-income North America | Acute lymphoid leukemia | -1.44 | -1.50 | -1.37 |
| Female | High-income North America | Chronic lymphoid leukemia | -0.07 | -0.42 | 0.28 |
| Female | High-income North America | Acute myeloid leukemia | 0.99 | 0.74 | 1.25 |
| Female | High-income North America | Chronic myeloid leukemia | -4.33 | -4.73 | -3.93 |
| Female | High-income North America | Leukemia | -0.63 | -0.80 | -0.47 |
| Female | High-income North America | Other leukemia | -1.45 | -1.60 | -1.31 |
| Both | High-income North America | Acute lymphoid leukemia | -1.47 | -1.56 | -1.39 |
| Both | High-income North America | Chronic lymphoid leukemia | -0.16 | -0.47 | 0.15 |
| Both | High-income North America | Acute myeloid leukemia | 1.04 | 0.78 | 1.29 |
| Both | High-income North America | Chronic myeloid leukemia | -4.28 | -4.66 | -3.90 |
| Both | High-income North America | Leukemia | -0.61 | -0.78 | -0.43 |
| Both | High-income North America | Other leukemia | -1.22 | -1.42 | -1.02 |
| Male | High-middle SDI | Acute lymphoid leukemia | -0.11 | -0.26 | 0.03 |
| Male | High-middle SDI | Chronic lymphoid leukemia | 2.61 | 2.32 | 2.91 |
| Male | High-middle SDI | Acute myeloid leukemia | 0.64 | 0.57 | 0.70 |
| Male | High-middle SDI | Chronic myeloid leukemia | -1.43 | -1.60 | -1.27 |
| Male | High-middle SDI | Leukemia | 0.76 | 0.68 | 0.83 |
| Male | High-middle SDI | Other leukemia | 0.83 | 0.74 | 0.91 |
| Female | High-middle SDI | Acute lymphoid leukemia | -0.04 | -0.16 | 0.08 |
| Female | High-middle SDI | Chronic lymphoid leukemia | 2.64 | 2.33 | 2.96 |
| Female | High-middle SDI | Acute myeloid leukemia | 0.03 | -0.05 | 0.11 |
| Female | High-middle SDI | Chronic myeloid leukemia | -2.34 | -2.53 | -2.15 |
| Female | High-middle SDI | Leukemia | 0.26 | 0.19 | 0.34 |
| Female | High-middle SDI | Other leukemia | 0.18 | 0.03 | 0.33 |
| Both | High-middle SDI | Acute lymphoid leukemia | -0.08 | -0.20 | 0.05 |
| Both | High-middle SDI | Chronic lymphoid leukemia | 2.70 | 2.40 | 3.00 |
| Both | High-middle SDI | Acute myeloid leukemia | 0.36 | 0.29 | 0.43 |
| Both | High-middle SDI | Chronic myeloid leukemia | -1.77 | -1.95 | -1.60 |
| Both | High-middle SDI | Leukemia | 0.56 | 0.50 | 0.63 |
| Both | High-middle SDI | Other leukemia | 0.57 | 0.46 | 0.67 |
| Male | Honduras | Acute lymphoid leukemia | 0.64 | 0.43 | 0.85 |
| Male | Honduras | Chronic lymphoid leukemia | 1.89 | 1.74 | 2.04 |
| Male | Honduras | Acute myeloid leukemia | 1.29 | 1.06 | 1.52 |
| Male | Honduras | Chronic myeloid leukemia | -0.30 | -0.52 | -0.09 |
| Male | Honduras | Leukemia | 0.18 | 0.05 | 0.30 |
| Male | Honduras | Other leukemia | -1.34 | -1.37 | -1.31 |
| Female | Honduras | Acute lymphoid leukemia | 1.41 | 1.13 | 1.70 |
| Female | Honduras | Chronic lymphoid leukemia | 1.73 | 1.54 | 1.92 |
| Female | Honduras | Acute myeloid leukemia | 1.44 | 1.17 | 1.71 |

| | | | | | |
|---|---|---|---|---|---|
| Female | Honduras | Chronic myeloid leukemia | -0.43 | -0.74 | -0.12 |
| Female | Honduras | Leukemia | 0.30 | 0.12 | 0.47 |
| Female | Honduras | Other leukemia | -1.37 | -1.43 | -1.30 |
| Both | Honduras | Acute lymphoid leukemia | 0.98 | 0.74 | 1.21 |
| Both | Honduras | Chronic lymphoid leukemia | 1.81 | 1.66 | 1.96 |
| Both | Honduras | Acute myeloid leukemia | 1.37 | 1.12 | 1.61 |
| Both | Honduras | Chronic myeloid leukemia | -0.37 | -0.63 | -0.11 |
| Both | Honduras | Leukemia | 0.23 | 0.09 | 0.38 |
| Both | Honduras | Other leukemia | -1.35 | -1.38 | -1.31 |
| Male | Hungary | Acute lymphoid leukemia | -2.45 | -2.64 | -2.26 |
| Male | Hungary | Chronic lymphoid leukemia | 0.16 | 0.03 | 0.28 |
| Male | Hungary | Acute myeloid leukemia | -0.06 | -0.22 | 0.10 |
| Male | Hungary | Chronic myeloid leukemia | -4.53 | -4.81 | -4.26 |
| Male | Hungary | Leukemia | -0.32 | -0.80 | 0.15 |
| Male | Hungary | Other leukemia | 1.34 | -0.27 | 2.98 |
| Female | Hungary | Acute lymphoid leukemia | -3.16 | -3.31 | -3.01 |
| Female | Hungary | Chronic lymphoid leukemia | 0.22 | 0.05 | 0.40 |
| Female | Hungary | Acute myeloid leukemia | 0.07 | -0.13 | 0.27 |
| Female | Hungary | Chronic myeloid leukemia | -5.58 | -5.91 | -5.25 |
| Female | Hungary | Leukemia | -0.38 | -0.93 | 0.17 |
| Female | Hungary | Other leukemia | 1.14 | -0.43 | 2.74 |
| Both | Hungary | Acute lymphoid leukemia | -2.74 | -2.91 | -2.58 |
| Both | Hungary | Chronic lymphoid leukemia | 0.19 | 0.06 | 0.32 |
| Both | Hungary | Acute myeloid leukemia | -0.03 | -0.18 | 0.12 |
| Both | Hungary | Chronic myeloid leukemia | -5.01 | -5.29 | -4.73 |
| Both | Hungary | Leukemia | -0.37 | -0.87 | 0.12 |
| Both | Hungary | Other leukemia | 1.23 | -0.35 | 2.83 |
| Male | Iceland | Acute lymphoid leukemia | -0.75 | -1.00 | -0.50 |
| Male | Iceland | Chronic lymphoid leukemia | -0.14 | -0.62 | 0.35 |
| Male | Iceland | Acute myeloid leukemia | -1.09 | -1.31 | -0.87 |
| Male | Iceland | Chronic myeloid leukemia | -0.64 | -1.02 | -0.25 |
| Male | Iceland | Leukemia | -0.43 | -0.54 | -0.33 |
| Male | Iceland | Other leukemia | -0.26 | -0.95 | 0.44 |
| Female | Iceland | Acute lymphoid leukemia | -2.54 | -2.81 | -2.27 |
| Female | Iceland | Chronic lymphoid leukemia | -1.72 | -2.27 | -1.17 |
| Female | Iceland | Acute myeloid leukemia | -1.23 | -1.45 | -1.01 |
| Female | Iceland | Chronic myeloid leukemia | -3.60 | -3.98 | -3.21 |
| Female | Iceland | Leukemia | -1.87 | -2.13 | -1.60 |
| Female | Iceland | Other leukemia | -2.28 | -2.74 | -1.82 |
| Both | Iceland | Acute lymphoid leukemia | -1.37 | -1.51 | -1.23 |
| Both | Iceland | Chronic lymphoid leukemia | -0.71 | -1.19 | -0.22 |
| Both | Iceland | Acute myeloid leukemia | -1.15 | -1.32 | -0.99 |
| Both | Iceland | Chronic myeloid leukemia | -1.35 | -1.70 | -0.99 |
| Both | Iceland | Leukemia | -0.96 | -1.07 | -0.84 |
| Both | Iceland | Other leukemia | -0.89 | -1.50 | -0.27 |
| Male | India | Acute lymphoid leukemia | -0.66 | -0.85 | -0.46 |
| Male | India | Chronic lymphoid leukemia | 1.71 | 1.61 | 1.81 |
| Male | India | Acute myeloid leukemia | 1.02 | 0.95 | 1.09 |

| | | | | | |
|---|---|---|---|---|---|
| Male | India | Chronic myeloid leukemia | 0.08 | -0.06 | 0.23 |
| Male | India | Leukemia | 0.04 | -0.06 | 0.13 |
| Male | India | Other leukemia | -0.75 | -0.87 | -0.64 |
| Female | India | Acute lymphoid leukemia | -0.44 | -0.61 | -0.26 |
| Female | India | Chronic lymphoid leukemia | 1.30 | 1.05 | 1.55 |
| Female | India | Acute myeloid leukemia | 0.19 | 0.08 | 0.30 |
| Female | India | Chronic myeloid leukemia | -0.99 | -1.14 | -0.84 |
| Female | India | Leukemia | -0.89 | -1.01 | -0.78 |
| Female | India | Other leukemia | -2.13 | -2.29 | -1.96 |
| Both | India | Acute lymphoid leukemia | -0.61 | -0.79 | -0.43 |
| Both | India | Chronic lymphoid leukemia | 1.48 | 1.35 | 1.62 |
| Both | India | Acute myeloid leukemia | 0.62 | 0.54 | 0.71 |
| Both | India | Chronic myeloid leukemia | -0.47 | -0.58 | -0.36 |
| Both | India | Leukemia | -0.40 | -0.50 | -0.31 |
| Both | India | Other leukemia | -1.43 | -1.54 | -1.31 |
| Male | Indonesia | Acute lymphoid leukemia | 1.42 | 1.15 | 1.69 |
| Male | Indonesia | Chronic lymphoid leukemia | 3.07 | 2.87 | 3.27 |
| Male | Indonesia | Acute myeloid leukemia | 1.48 | 1.29 | 1.67 |
| Male | Indonesia | Chronic myeloid leukemia | 1.01 | 0.81 | 1.22 |
| Male | Indonesia | Leukemia | 0.46 | 0.33 | 0.58 |
| Male | Indonesia | Other leukemia | -0.30 | -0.37 | -0.22 |
| Female | Indonesia | Acute lymphoid leukemia | 1.29 | 1.08 | 1.51 |
| Female | Indonesia | Chronic lymphoid leukemia | 1.98 | 1.86 | 2.10 |
| Female | Indonesia | Acute myeloid leukemia | 1.01 | 0.87 | 1.14 |
| Female | Indonesia | Chronic myeloid leukemia | -0.32 | -0.55 | -0.09 |
| Female | Indonesia | Leukemia | -0.30 | -0.41 | -0.20 |
| Female | Indonesia | Other leukemia | -0.98 | -1.07 | -0.90 |
| Both | Indonesia | Acute lymphoid leukemia | 1.36 | 1.11 | 1.61 |
| Both | Indonesia | Chronic lymphoid leukemia | 2.58 | 2.41 | 2.75 |
| Both | Indonesia | Acute myeloid leukemia | 1.22 | 1.07 | 1.38 |
| Both | Indonesia | Chronic myeloid leukemia | 0.35 | 0.14 | 0.57 |
| Both | Indonesia | Leukemia | 0.07 | -0.04 | 0.19 |
| Both | Indonesia | Other leukemia | -0.67 | -0.75 | -0.58 |
| Male | Iran | Acute lymphoid leukemia | -0.13 | -0.59 | 0.34 |
| Male | Iran | Chronic lymphoid leukemia | 3.61 | 3.22 | 4.01 |
| Male | Iran | Acute myeloid leukemia | 0.66 | 0.31 | 1.02 |
| Male | Iran | Chronic myeloid leukemia | -1.40 | -1.63 | -1.18 |
| Male | Iran | Leukemia | 1.23 | 0.85 | 1.60 |
| Male | Iran | Other leukemia | 2.07 | 1.50 | 2.63 |
| Female | Iran | Acute lymphoid leukemia | 0.06 | -0.54 | 0.67 |
| Female | Iran | Chronic lymphoid leukemia | 2.03 | 1.88 | 2.17 |
| Female | Iran | Acute myeloid leukemia | -0.42 | -0.86 | 0.01 |
| Female | Iran | Chronic myeloid leukemia | -2.47 | -2.67 | -2.27 |
| Female | Iran | Leukemia | 0.66 | 0.29 | 1.03 |
| Female | Iran | Other leukemia | 1.63 | 1.13 | 2.14 |
| Both | Iran | Acute lymphoid leukemia | -0.08 | -0.59 | 0.44 |
| Both | Iran | Chronic lymphoid leukemia | 2.91 | 2.68 | 3.15 |
| Both | Iran | Acute myeloid leukemia | 0.15 | -0.24 | 0.53 |

| | | | | | |
|---|---|---|---|---|---|
| Both | Iran | Chronic myeloid leukemia | -1.82 | -2.03 | -1.61 |
| Both | Iran | Leukemia | 0.98 | 0.62 | 1.34 |
| Both | Iran | Other leukemia | 1.89 | 1.36 | 2.41 |
| Male | Iraq | Acute lymphoid leukemia | -1.43 | -1.75 | -1.12 |
| Male | Iraq | Chronic lymphoid leukemia | -1.62 | -1.73 | -1.51 |
| Male | Iraq | Acute myeloid leukemia | -1.15 | -1.32 | -0.98 |
| Male | Iraq | Chronic myeloid leukemia | -3.05 | -3.35 | -2.74 |
| Male | Iraq | Leukemia | -1.84 | -2.14 | -1.54 |
| Male | Iraq | Other leukemia | -2.00 | -2.35 | -1.65 |
| Female | Iraq | Acute lymphoid leukemia | -0.61 | -0.84 | -0.38 |
| Female | Iraq | Chronic lymphoid leukemia | -2.31 | -2.69 | -1.92 |
| Female | Iraq | Acute myeloid leukemia | -1.45 | -1.70 | -1.19 |
| Female | Iraq | Chronic myeloid leukemia | -4.03 | -4.58 | -3.47 |
| Female | Iraq | Leukemia | -2.05 | -2.37 | -1.74 |
| Female | Iraq | Other leukemia | -2.24 | -2.57 | -1.91 |
| Both | Iraq | Acute lymphoid leukemia | -1.11 | -1.40 | -0.83 |
| Both | Iraq | Chronic lymphoid leukemia | -1.85 | -2.06 | -1.63 |
| Both | Iraq | Acute myeloid leukemia | -1.28 | -1.49 | -1.07 |
| Both | Iraq | Chronic myeloid leukemia | -3.46 | -3.88 | -3.05 |
| Both | Iraq | Leukemia | -1.92 | -2.23 | -1.62 |
| Both | Iraq | Other leukemia | -2.10 | -2.44 | -1.76 |
| Male | Ireland | Acute lymphoid leukemia | -2.66 | -3.22 | -2.11 |
| Male | Ireland | Chronic lymphoid leukemia | 2.24 | 1.94 | 2.54 |
| Male | Ireland | Acute myeloid leukemia | 1.54 | 1.21 | 1.88 |
| Male | Ireland | Chronic myeloid leukemia | -3.15 | -3.58 | -2.70 |
| Male | Ireland | Leukemia | 0.04 | -0.52 | 0.59 |
| Male | Ireland | Other leukemia | -1.53 | -3.18 | 0.14 |
| Female | Ireland | Acute lymphoid leukemia | -2.12 | -2.46 | -1.78 |
| Female | Ireland | Chronic lymphoid leukemia | 1.57 | 1.24 | 1.89 |
| Female | Ireland | Acute myeloid leukemia | 0.59 | 0.32 | 0.87 |
| Female | Ireland | Chronic myeloid leukemia | -3.47 | -3.82 | -3.12 |
| Female | Ireland | Leukemia | -0.58 | -1.09 | -0.06 |
| Female | Ireland | Other leukemia | -2.20 | -3.73 | -0.65 |
| Both | Ireland | Acute lymphoid leukemia | -2.44 | -2.71 | -2.18 |
| Both | Ireland | Chronic lymphoid leukemia | 2.02 | 1.75 | 2.30 |
| Both | Ireland | Acute myeloid leukemia | 1.13 | 0.86 | 1.41 |
| Both | Ireland | Chronic myeloid leukemia | -3.23 | -3.61 | -2.86 |
| Both | Ireland | Leukemia | -0.18 | -0.71 | 0.35 |
| Both | Ireland | Other leukemia | -1.75 | -3.36 | -0.12 |
| Male | Israel | Acute lymphoid leukemia | -2.15 | -2.54 | -1.77 |
| Male | Israel | Chronic lymphoid leukemia | 0.10 | -0.29 | 0.49 |
| Male | Israel | Acute myeloid leukemia | -0.25 | -0.50 | 0.00 |
| Male | Israel | Chronic myeloid leukemia | -3.61 | -4.07 | -3.14 |
| Male | Israel | Leukemia | -0.65 | -1.02 | -0.29 |
| Male | Israel | Other leukemia | -0.81 | -1.47 | -0.14 |
| Female | Israel | Acute lymphoid leukemia | -2.01 | -2.43 | -1.59 |
| Female | Israel | Chronic lymphoid leukemia | -0.49 | -0.81 | -0.17 |
| Female | Israel | Acute myeloid leukemia | -0.29 | -0.52 | -0.07 |

| | | | | | |
|---|---|---|---|---|---|
| Female | Israel | Chronic myeloid leukemia | -6.25 | -6.72 | -5.79 |
| Female | Israel | Leukemia | -1.25 | -1.48 | -1.01 |
| Female | Israel | Other leukemia | -1.96 | -2.50 | -1.42 |
| Both | Israel | Acute lymphoid leukemia | -2.09 | -2.46 | -1.71 |
| Both | Israel | Chronic lymphoid leukemia | -0.14 | -0.49 | 0.20 |
| Both | Israel | Acute myeloid leukemia | -0.29 | -0.50 | -0.08 |
| Both | Israel | Chronic myeloid leukemia | -4.77 | -5.20 | -4.35 |
| Both | Israel | Leukemia | -0.92 | -1.23 | -0.62 |
| Both | Israel | Other leukemia | -1.30 | -1.91 | -0.69 |
| Male | Italy | Acute lymphoid leukemia | -1.19 | -1.30 | -1.08 |
| Male | Italy | Chronic lymphoid leukemia | 0.87 | 0.50 | 1.24 |
| Male | Italy | Acute myeloid leukemia | 1.80 | 1.54 | 2.06 |
| Male | Italy | Chronic myeloid leukemia | -2.64 | -2.88 | -2.41 |
| Male | Italy | Leukemia | -0.18 | -0.36 | -0.01 |
| Male | Italy | Other leukemia | -1.46 | -1.65 | -1.27 |
| Female | Italy | Acute lymphoid leukemia | -1.56 | -1.66 | -1.46 |
| Female | Italy | Chronic lymphoid leukemia | 0.43 | 0.23 | 0.64 |
| Female | Italy | Acute myeloid leukemia | 1.49 | 1.19 | 1.78 |
| Female | Italy | Chronic myeloid leukemia | -4.36 | -4.54 | -4.17 |
| Female | Italy | Leukemia | -0.82 | -0.93 | -0.70 |
| Female | Italy | Other leukemia | -2.54 | -2.79 | -2.29 |
| Both | Italy | Acute lymphoid leukemia | -1.32 | -1.41 | -1.24 |
| Both | Italy | Chronic lymphoid leukemia | 0.70 | 0.41 | 0.99 |
| Both | Italy | Acute myeloid leukemia | 1.64 | 1.38 | 1.89 |
| Both | Italy | Chronic myeloid leukemia | -3.42 | -3.56 | -3.27 |
| Both | Italy | Leukemia | -0.46 | -0.58 | -0.34 |
| Both | Italy | Other leukemia | -1.92 | -2.12 | -1.72 |
| Male | Ivory Coast | Acute lymphoid leukemia | -0.83 | -1.05 | -0.62 |
| Male | Ivory Coast | Chronic lymphoid leukemia | 1.53 | 1.42 | 1.63 |
| Male | Ivory Coast | Acute myeloid leukemia | 0.85 | 0.69 | 1.00 |
| Male | Ivory Coast | Chronic myeloid leukemia | 0.35 | 0.18 | 0.51 |
| Male | Ivory Coast | Leukemia | 0.37 | 0.25 | 0.50 |
| Male | Ivory Coast | Other leukemia | 0.11 | -0.04 | 0.26 |
| Female | Ivory Coast | Acute lymphoid leukemia | 0.39 | 0.19 | 0.60 |
| Female | Ivory Coast | Chronic lymphoid leukemia | 0.03 | -0.02 | 0.08 |
| Female | Ivory Coast | Acute myeloid leukemia | 0.38 | 0.26 | 0.51 |
| Female | Ivory Coast | Chronic myeloid leukemia | -0.51 | -0.59 | -0.43 |
| Female | Ivory Coast | Leukemia | -0.16 | -0.25 | -0.07 |
| Female | Ivory Coast | Other leukemia | -0.66 | -0.78 | -0.54 |
| Both | Ivory Coast | Acute lymphoid leukemia | -0.55 | -0.76 | -0.34 |
| Both | Ivory Coast | Chronic lymphoid leukemia | 0.69 | 0.62 | 0.76 |
| Both | Ivory Coast | Acute myeloid leukemia | 0.73 | 0.60 | 0.86 |
| Both | Ivory Coast | Chronic myeloid leukemia | -0.01 | -0.14 | 0.12 |
| Both | Ivory Coast | Leukemia | 0.18 | 0.08 | 0.27 |
| Both | Ivory Coast | Other leukemia | -0.22 | -0.32 | -0.12 |
| Male | Jamaica | Acute lymphoid leukemia | -1.31 | -1.92 | -0.71 |
| Male | Jamaica | Chronic lymphoid leukemia | 7.52 | 6.38 | 8.68 |
| Male | Jamaica | Acute myeloid leukemia | 1.94 | 1.57 | 2.32 |

| | | | | | |
|---|---|---|---|---|---|
| Male | Jamaica | Chronic myeloid leukemia | 2.62 | 2.29 | 2.96 |
| Male | Jamaica | Leukemia | 1.40 | 0.85 | 1.95 |
| Male | Jamaica | Other leukemia | 1.09 | 0.27 | 1.92 |
| Female | Jamaica | Acute lymphoid leukemia | 0.60 | 0.37 | 0.85 |
| Female | Jamaica | Chronic lymphoid leukemia | 6.30 | 5.71 | 6.91 |
| Female | Jamaica | Acute myeloid leukemia | 2.90 | 2.49 | 3.31 |
| Female | Jamaica | Chronic myeloid leukemia | 3.17 | 2.72 | 3.61 |
| Female | Jamaica | Leukemia | 1.63 | 1.17 | 2.09 |
| Female | Jamaica | Other leukemia | 0.84 | 0.12 | 1.56 |
| Both | Jamaica | Acute lymphoid leukemia | -0.53 | -0.97 | -0.10 |
| Both | Jamaica | Chronic lymphoid leukemia | 7.02 | 6.18 | 7.86 |
| Both | Jamaica | Acute myeloid leukemia | 2.41 | 2.05 | 2.78 |
| Both | Jamaica | Chronic myeloid leukemia | 2.87 | 2.55 | 3.20 |
| Both | Jamaica | Leukemia | 1.52 | 1.02 | 2.03 |
| Both | Jamaica | Other leukemia | 1.02 | 0.25 | 1.80 |
| Male | Japan | Acute lymphoid leukemia | -2.20 | -2.31 | -2.09 |
| Male | Japan | Chronic lymphoid leukemia | 2.05 | 1.81 | 2.30 |
| Male | Japan | Acute myeloid leukemia | 1.12 | 0.87 | 1.37 |
| Male | Japan | Chronic myeloid leukemia | -3.54 | -3.76 | -3.33 |
| Male | Japan | Leukemia | -0.37 | -0.48 | -0.27 |
| Male | Japan | Other leukemia | -0.81 | -0.94 | -0.69 |
| Female | Japan | Acute lymphoid leukemia | -2.10 | -2.27 | -1.93 |
| Female | Japan | Chronic lymphoid leukemia | 2.06 | 1.79 | 2.33 |
| Female | Japan | Acute myeloid leukemia | 1.04 | 0.83 | 1.26 |
| Female | Japan | Chronic myeloid leukemia | -3.79 | -4.06 | -3.51 |
| Female | Japan | Leukemia | -0.43 | -0.56 | -0.30 |
| Female | Japan | Other leukemia | -1.01 | -1.22 | -0.80 |
| Both | Japan | Acute lymphoid leukemia | -2.16 | -2.29 | -2.03 |
| Both | Japan | Chronic lymphoid leukemia | 2.09 | 1.88 | 2.30 |
| Both | Japan | Acute myeloid leukemia | 1.08 | 0.87 | 1.29 |
| Both | Japan | Chronic myeloid leukemia | -3.62 | -3.86 | -3.39 |
| Both | Japan | Leukemia | -0.40 | -0.50 | -0.31 |
| Both | Japan | Other leukemia | -0.90 | -1.06 | -0.74 |
| Male | Jordan | Acute lymphoid leukemia | -0.96 | -1.08 | -0.84 |
| Male | Jordan | Chronic lymphoid leukemia | 2.23 | 1.99 | 2.46 |
| Male | Jordan | Acute myeloid leukemia | 0.50 | 0.38 | 0.61 |
| Male | Jordan | Chronic myeloid leukemia | -1.20 | -1.34 | -1.07 |
| Male | Jordan | Leukemia | -0.40 | -0.47 | -0.34 |
| Male | Jordan | Other leukemia | -0.46 | -0.52 | -0.41 |
| Female | Jordan | Acute lymphoid leukemia | -0.98 | -1.14 | -0.81 |
| Female | Jordan | Chronic lymphoid leukemia | -0.66 | -1.24 | -0.09 |
| Female | Jordan | Acute myeloid leukemia | -0.86 | -1.08 | -0.65 |
| Female | Jordan | Chronic myeloid leukemia | -4.01 | -4.66 | -3.35 |
| Female | Jordan | Leukemia | -1.17 | -1.55 | -0.80 |
| Female | Jordan | Other leukemia | -1.17 | -1.56 | -0.77 |
| Both | Jordan | Acute lymphoid leukemia | -0.98 | -1.13 | -0.83 |
| Both | Jordan | Chronic lymphoid leukemia | 0.95 | 0.72 | 1.18 |
| Both | Jordan | Acute myeloid leukemia | -0.11 | -0.25 | 0.04 |

| | | | | | |
|---|---|---|---|---|---|
| Both | Jordan | Chronic myeloid leukemia | -2.19 | -2.46 | -1.92 |
| Both | Jordan | Leukemia | -0.74 | -0.90 | -0.57 |
| Both | Jordan | Other leukemia | -0.76 | -0.93 | -0.59 |
| Male | Kazakhstan | Acute lymphoid leukemia | -0.92 | -1.17 | -0.67 |
| Male | Kazakhstan | Chronic lymphoid leukemia | 1.13 | 0.89 | 1.37 |
| Male | Kazakhstan | Acute myeloid leukemia | -0.37 | -0.54 | -0.19 |
| Male | Kazakhstan | Chronic myeloid leukemia | -2.32 | -2.69 | -1.94 |
| Male | Kazakhstan | Leukemia | -0.69 | -0.85 | -0.53 |
| Male | Kazakhstan | Other leukemia | -0.95 | -1.08 | -0.83 |
| Female | Kazakhstan | Acute lymphoid leukemia | -0.90 | -1.06 | -0.74 |
| Female | Kazakhstan | Chronic lymphoid leukemia | 0.15 | 0.00 | 0.29 |
| Female | Kazakhstan | Acute myeloid leukemia | 0.13 | -0.13 | 0.39 |
| Female | Kazakhstan | Chronic myeloid leukemia | -1.65 | -2.25 | -1.05 |
| Female | Kazakhstan | Leukemia | -0.39 | -0.57 | -0.21 |
| Female | Kazakhstan | Other leukemia | -0.36 | -0.45 | -0.26 |
| Both | Kazakhstan | Acute lymphoid leukemia | -0.95 | -1.16 | -0.75 |
| Both | Kazakhstan | Chronic lymphoid leukemia | 0.65 | 0.48 | 0.81 |
| Both | Kazakhstan | Acute myeloid leukemia | -0.16 | -0.35 | 0.04 |
| Both | Kazakhstan | Chronic myeloid leukemia | -2.02 | -2.50 | -1.54 |
| Both | Kazakhstan | Leukemia | -0.57 | -0.73 | -0.41 |
| Both | Kazakhstan | Other leukemia | -0.69 | -0.78 | -0.59 |
| Male | Kenya | Acute lymphoid leukemia | 0.93 | 0.78 | 1.07 |
| Male | Kenya | Chronic lymphoid leukemia | 1.56 | 1.39 | 1.73 |
| Male | Kenya | Acute myeloid leukemia | 0.91 | 0.71 | 1.12 |
| Male | Kenya | Chronic myeloid leukemia | 1.17 | 0.90 | 1.44 |
| Male | Kenya | Leukemia | 0.96 | 0.79 | 1.14 |
| Male | Kenya | Other leukemia | 0.81 | 0.60 | 1.02 |
| Female | Kenya | Acute lymphoid leukemia | 0.86 | 0.67 | 1.05 |
| Female | Kenya | Chronic lymphoid leukemia | 0.07 | 0.03 | 0.11 |
| Female | Kenya | Acute myeloid leukemia | 0.20 | 0.04 | 0.36 |
| Female | Kenya | Chronic myeloid leukemia | -0.98 | -1.08 | -0.89 |
| Female | Kenya | Leukemia | -0.26 | -0.36 | -0.17 |
| Female | Kenya | Other leukemia | -1.13 | -1.21 | -1.06 |
| Both | Kenya | Acute lymphoid leukemia | 0.90 | 0.78 | 1.02 |
| Both | Kenya | Chronic lymphoid leukemia | 0.71 | 0.65 | 0.77 |
| Both | Kenya | Acute myeloid leukemia | 0.57 | 0.45 | 0.69 |
| Both | Kenya | Chronic myeloid leukemia | -0.05 | -0.20 | 0.10 |
| Both | Kenya | Leukemia | 0.39 | 0.30 | 0.48 |
| Both | Kenya | Other leukemia | -0.25 | -0.35 | -0.15 |
| Male | Kiribati | Acute lymphoid leukemia | 0.36 | 0.05 | 0.67 |
| Male | Kiribati | Chronic lymphoid leukemia | -0.87 | -1.02 | -0.73 |
| Male | Kiribati | Acute myeloid leukemia | 0.75 | 0.57 | 0.93 |
| Male | Kiribati | Chronic myeloid leukemia | -0.12 | -0.27 | 0.02 |
| Male | Kiribati | Leukemia | 0.27 | 0.14 | 0.41 |
| Male | Kiribati | Other leukemia | 0.02 | -0.06 | 0.11 |
| Female | Kiribati | Acute lymphoid leukemia | 0.70 | 0.46 | 0.94 |
| Female | Kiribati | Chronic lymphoid leukemia | 0.47 | 0.19 | 0.74 |
| Female | Kiribati | Acute myeloid leukemia | -0.11 | -0.39 | 0.17 |

| | | | | | |
|---|---|---|---|---|---|
| Female | Kiribati | Chronic myeloid leukemia | -0.56 | -0.74 | -0.39 |
| Female | Kiribati | Leukemia | -0.21 | -0.44 | 0.02 |
| Female | Kiribati | Other leukemia | -0.50 | -0.71 | -0.28 |
| Both | Kiribati | Acute lymphoid leukemia | 0.49 | 0.31 | 0.66 |
| Both | Kiribati | Chronic lymphoid leukemia | 0.56 | 0.30 | 0.81 |
| Both | Kiribati | Acute myeloid leukemia | 0.31 | 0.24 | 0.39 |
| Both | Kiribati | Chronic myeloid leukemia | -0.34 | -0.43 | -0.25 |
| Both | Kiribati | Leukemia | 0.01 | -0.08 | 0.10 |
| Both | Kiribati | Other leukemia | -0.28 | -0.38 | -0.18 |
| Male | Kuwait | Acute lymphoid leukemia | -1.01 | -1.71 | -0.30 |
| Male | Kuwait | Chronic lymphoid leukemia | 2.36 | 1.78 | 2.95 |
| Male | Kuwait | Acute myeloid leukemia | 1.60 | 1.01 | 2.19 |
| Male | Kuwait | Chronic myeloid leukemia | -0.98 | -1.29 | -0.66 |
| Male | Kuwait | Leukemia | -0.79 | -1.17 | -0.42 |
| Male | Kuwait | Other leukemia | -1.79 | -2.18 | -1.39 |
| Female | Kuwait | Acute lymphoid leukemia | -0.60 | -1.63 | 0.44 |
| Female | Kuwait | Chronic lymphoid leukemia | 1.41 | 1.02 | 1.80 |
| Female | Kuwait | Acute myeloid leukemia | 0.55 | -0.45 | 1.56 |
| Female | Kuwait | Chronic myeloid leukemia | -0.09 | -1.33 | 1.17 |
| Female | Kuwait | Leukemia | -2.42 | -2.84 | -2.01 |
| Female | Kuwait | Other leukemia | -3.40 | -3.74 | -3.06 |
| Both | Kuwait | Acute lymphoid leukemia | -0.81 | -1.50 | -0.12 |
| Both | Kuwait | Chronic lymphoid leukemia | 2.20 | 1.70 | 2.70 |
| Both | Kuwait | Acute myeloid leukemia | 1.29 | 0.63 | 1.96 |
| Both | Kuwait | Chronic myeloid leukemia | -0.75 | -1.17 | -0.33 |
| Both | Kuwait | Leukemia | -1.32 | -1.64 | -1.00 |
| Both | Kuwait | Other leukemia | -2.38 | -2.68 | -2.08 |
| Male | Kyrgyzstan | Acute lymphoid leukemia | 0.57 | 0.02 | 1.12 |
| Male | Kyrgyzstan | Chronic lymphoid leukemia | -1.71 | -2.23 | -1.19 |
| Male | Kyrgyzstan | Acute myeloid leukemia | 0.26 | -0.14 | 0.66 |
| Male | Kyrgyzstan | Chronic myeloid leukemia | -3.39 | -4.03 | -2.75 |
| Male | Kyrgyzstan | Leukemia | -1.28 | -1.46 | -1.10 |
| Male | Kyrgyzstan | Other leukemia | -2.42 | -2.61 | -2.24 |
| Female | Kyrgyzstan | Acute lymphoid leukemia | -0.59 | -0.91 | -0.28 |
| Female | Kyrgyzstan | Chronic lymphoid leukemia | -3.13 | -3.64 | -2.63 |
| Female | Kyrgyzstan | Acute myeloid leukemia | 2.06 | 1.38 | 2.74 |
| Female | Kyrgyzstan | Chronic myeloid leukemia | -0.62 | -1.38 | 0.15 |
| Female | Kyrgyzstan | Leukemia | -1.59 | -1.71 | -1.46 |
| Female | Kyrgyzstan | Other leukemia | -3.54 | -3.75 | -3.32 |
| Both | Kyrgyzstan | Acute lymphoid leukemia | 0.10 | -0.33 | 0.53 |
| Both | Kyrgyzstan | Chronic lymphoid leukemia | -2.49 | -2.81 | -2.16 |
| Both | Kyrgyzstan | Acute myeloid leukemia | 1.04 | 0.55 | 1.53 |
| Both | Kyrgyzstan | Chronic myeloid leukemia | -2.20 | -2.81 | -1.59 |
| Both | Kyrgyzstan | Leukemia | -1.43 | -1.57 | -1.28 |
| Both | Kyrgyzstan | Other leukemia | -2.97 | -3.15 | -2.79 |
| Male | Laos | Acute lymphoid leukemia | 1.12 | 0.88 | 1.36 |
| Male | Laos | Chronic lymphoid leukemia | 2.51 | 2.41 | 2.60 |
| Male | Laos | Acute myeloid leukemia | 1.70 | 1.67 | 1.73 |

| | | | | | |
|---|---|---|---|---|---|
| Male | Laos | Chronic myeloid leukemia | -0.61 | -0.64 | -0.58 |
| Male | Laos | Leukemia | -0.11 | -0.17 | -0.06 |
| Male | Laos | Other leukemia | -0.86 | -0.92 | -0.81 |
| Female | Laos | Acute lymphoid leukemia | 1.45 | 1.29 | 1.61 |
| Female | Laos | Chronic lymphoid leukemia | 1.12 | 1.06 | 1.18 |
| Female | Laos | Acute myeloid leukemia | 0.63 | 0.52 | 0.75 |
| Female | Laos | Chronic myeloid leukemia | -1.33 | -1.53 | -1.14 |
| Female | Laos | Leukemia | -0.81 | -0.93 | -0.68 |
| Female | Laos | Other leukemia | -1.49 | -1.63 | -1.34 |
| Both | Laos | Acute lymphoid leukemia | 1.25 | 1.04 | 1.46 |
| Both | Laos | Chronic lymphoid leukemia | 1.91 | 1.86 | 1.97 |
| Both | Laos | Acute myeloid leukemia | 1.06 | 0.99 | 1.13 |
| Both | Laos | Chronic myeloid leukemia | -1.02 | -1.14 | -0.90 |
| Both | Laos | Leukemia | -0.48 | -0.57 | -0.39 |
| Both | Laos | Other leukemia | -1.21 | -1.31 | -1.10 |
| Male | Latvia | Acute lymphoid leukemia | -1.83 | -2.12 | -1.53 |
| Male | Latvia | Chronic lymphoid leukemia | 1.84 | 1.56 | 2.12 |
| Male | Latvia | Acute myeloid leukemia | 0.33 | 0.10 | 0.56 |
| Male | Latvia | Chronic myeloid leukemia | -0.57 | -0.88 | -0.26 |
| Male | Latvia | Leukemia | 0.57 | 0.34 | 0.80 |
| Male | Latvia | Other leukemia | 0.75 | 0.32 | 1.18 |
| Female | Latvia | Acute lymphoid leukemia | -0.96 | -1.20 | -0.72 |
| Female | Latvia | Chronic lymphoid leukemia | 2.64 | 2.17 | 3.10 |
| Female | Latvia | Acute myeloid leukemia | 0.75 | 0.37 | 1.14 |
| Female | Latvia | Chronic myeloid leukemia | -1.40 | -1.70 | -1.11 |
| Female | Latvia | Leukemia | 1.05 | 0.78 | 1.33 |
| Female | Latvia | Other leukemia | 1.03 | 0.52 | 1.54 |
| Both | Latvia | Acute lymphoid leukemia | -1.48 | -1.64 | -1.32 |
| Both | Latvia | Chronic lymphoid leukemia | 2.30 | 1.95 | 2.65 |
| Both | Latvia | Acute myeloid leukemia | 0.62 | 0.32 | 0.93 |
| Both | Latvia | Chronic myeloid leukemia | -0.98 | -1.27 | -0.70 |
| Both | Latvia | Leukemia | 0.81 | 0.59 | 1.04 |
| Both | Latvia | Other leukemia | 0.90 | 0.43 | 1.37 |
| Male | Lebanon | Acute lymphoid leukemia | 0.41 | 0.15 | 0.67 |
| Male | Lebanon | Chronic lymphoid leukemia | 2.98 | 2.55 | 3.40 |
| Male | Lebanon | Acute myeloid leukemia | 0.80 | 0.37 | 1.22 |
| Male | Lebanon | Chronic myeloid leukemia | 1.04 | 0.77 | 1.31 |
| Male | Lebanon | Leukemia | 0.55 | 0.27 | 0.83 |
| Male | Lebanon | Other leukemia | 0.37 | 0.11 | 0.63 |
| Female | Lebanon | Acute lymphoid leukemia | -0.15 | -0.43 | 0.12 |
| Female | Lebanon | Chronic lymphoid leukemia | 4.76 | 4.50 | 5.02 |
| Female | Lebanon | Acute myeloid leukemia | 0.25 | -0.06 | 0.55 |
| Female | Lebanon | Chronic myeloid leukemia | -0.68 | -0.86 | -0.51 |
| Female | Lebanon | Leukemia | 0.09 | -0.16 | 0.34 |
| Female | Lebanon | Other leukemia | -0.13 | -0.37 | 0.10 |
| Both | Lebanon | Acute lymphoid leukemia | 0.20 | -0.07 | 0.47 |
| Both | Lebanon | Chronic lymphoid leukemia | 3.88 | 3.53 | 4.23 |
| Both | Lebanon | Acute myeloid leukemia | 0.60 | 0.23 | 0.97 |

| | | | | | |
|---|---|---|---|---|---|
| Both | Lebanon | Chronic myeloid leukemia | 0.31 | 0.09 | 0.54 |
| Both | Lebanon | Leukemia | 0.36 | 0.10 | 0.63 |
| Both | Lebanon | Other leukemia | 0.16 | -0.09 | 0.40 |
| Male | Lesotho | Acute lymphoid leukemia | 1.85 | 1.56 | 2.14 |
| Male | Lesotho | Chronic lymphoid leukemia | 1.82 | 1.68 | 1.96 |
| Male | Lesotho | Acute myeloid leukemia | 0.72 | 0.53 | 0.90 |
| Male | Lesotho | Chronic myeloid leukemia | 2.02 | 1.70 | 2.35 |
| Male | Lesotho | Leukemia | 0.90 | 0.69 | 1.11 |
| Male | Lesotho | Other leukemia | 0.82 | 0.56 | 1.08 |
| Female | Lesotho | Acute lymphoid leukemia | 1.78 | 1.00 | 2.58 |
| Female | Lesotho | Chronic lymphoid leukemia | 1.80 | 1.35 | 2.25 |
| Female | Lesotho | Acute myeloid leukemia | 2.22 | 1.38 | 3.06 |
| Female | Lesotho | Chronic myeloid leukemia | 1.74 | 1.12 | 2.37 |
| Female | Lesotho | Leukemia | 1.18 | 0.61 | 1.75 |
| Female | Lesotho | Other leukemia | 0.99 | 0.45 | 1.52 |
| Both | Lesotho | Acute lymphoid leukemia | 1.79 | 1.24 | 2.35 |
| Both | Lesotho | Chronic lymphoid leukemia | 1.58 | 1.36 | 1.80 |
| Both | Lesotho | Acute myeloid leukemia | 0.83 | 0.52 | 1.14 |
| Both | Lesotho | Chronic myeloid leukemia | 1.89 | 1.39 | 2.39 |
| Both | Lesotho | Leukemia | 1.06 | 0.66 | 1.46 |
| Both | Lesotho | Other leukemia | 1.05 | 0.60 | 1.49 |
| Male | Liberia | Acute lymphoid leukemia | -2.34 | -2.82 | -1.86 |
| Male | Liberia | Chronic lymphoid leukemia | 1.17 | 1.01 | 1.33 |
| Male | Liberia | Acute myeloid leukemia | -0.10 | -0.43 | 0.24 |
| Male | Liberia | Chronic myeloid leukemia | 0.10 | -0.01 | 0.20 |
| Male | Liberia | Leukemia | -0.25 | -0.48 | -0.02 |
| Male | Liberia | Other leukemia | 0.07 | -0.03 | 0.18 |
| Female | Liberia | Acute lymphoid leukemia | 0.01 | -0.35 | 0.37 |
| Female | Liberia | Chronic lymphoid leukemia | 0.50 | 0.43 | 0.58 |
| Female | Liberia | Acute myeloid leukemia | 0.10 | -0.14 | 0.34 |
| Female | Liberia | Chronic myeloid leukemia | -1.01 | -1.17 | -0.84 |
| Female | Liberia | Leukemia | -0.41 | -0.58 | -0.24 |
| Female | Liberia | Other leukemia | -0.92 | -1.10 | -0.75 |
| Both | Liberia | Acute lymphoid leukemia | -1.62 | -2.07 | -1.16 |
| Both | Liberia | Chronic lymphoid leukemia | 0.80 | 0.73 | 0.87 |
| Both | Liberia | Acute myeloid leukemia | -0.03 | -0.33 | 0.28 |
| Both | Liberia | Chronic myeloid leukemia | -0.49 | -0.62 | -0.36 |
| Both | Liberia | Leukemia | -0.34 | -0.54 | -0.13 |
| Both | Liberia | Other leukemia | -0.39 | -0.53 | -0.24 |
| Male | Libya | Acute lymphoid leukemia | 1.64 | 1.38 | 1.90 |
| Male | Libya | Chronic lymphoid leukemia | 2.23 | 1.86 | 2.61 |
| Male | Libya | Acute myeloid leukemia | 0.75 | 0.49 | 1.01 |
| Male | Libya | Chronic myeloid leukemia | 1.25 | 1.10 | 1.40 |
| Male | Libya | Leukemia | 1.16 | 0.94 | 1.39 |
| Male | Libya | Other leukemia | 1.10 | 0.89 | 1.31 |
| Female | Libya | Acute lymphoid leukemia | 1.55 | 1.23 | 1.87 |
| Female | Libya | Chronic lymphoid leukemia | 3.45 | 3.12 | 3.77 |
| Female | Libya | Acute myeloid leukemia | 0.87 | 0.59 | 1.16 |

| | | | | | |
|---|---|---|---|---|---|
| Female | Libya | Chronic myeloid leukemia | 0.10 | -0.08 | 0.27 |
| Female | Libya | Leukemia | 0.92 | 0.66 | 1.18 |
| Female | Libya | Other leukemia | 0.76 | 0.51 | 1.02 |
| Both | Libya | Acute lymphoid leukemia | 1.58 | 1.30 | 1.86 |
| Both | Libya | Chronic lymphoid leukemia | 2.80 | 2.46 | 3.14 |
| Both | Libya | Acute myeloid leukemia | 0.79 | 0.53 | 1.06 |
| Both | Libya | Chronic myeloid leukemia | 0.73 | 0.59 | 0.87 |
| Both | Libya | Leukemia | 1.04 | 0.81 | 1.28 |
| Both | Libya | Other leukemia | 0.93 | 0.70 | 1.16 |
| Male | Lithuania | Acute lymphoid leukemia | -1.37 | -1.92 | -0.82 |
| Male | Lithuania | Chronic lymphoid leukemia | 5.12 | 4.11 | 6.14 |
| Male | Lithuania | Acute myeloid leukemia | 2.99 | 2.57 | 3.43 |
| Male | Lithuania | Chronic myeloid leukemia | -2.59 | -2.89 | -2.29 |
| Male | Lithuania | Leukemia | 1.15 | 0.90 | 1.40 |
| Male | Lithuania | Other leukemia | -1.59 | -2.35 | -0.82 |
| Female | Lithuania | Acute lymphoid leukemia | -2.14 | -2.60 | -1.68 |
| Female | Lithuania | Chronic lymphoid leukemia | 3.30 | 2.58 | 4.03 |
| Female | Lithuania | Acute myeloid leukemia | 1.55 | 1.14 | 1.96 |
| Female | Lithuania | Chronic myeloid leukemia | -2.18 | -2.93 | -1.42 |
| Female | Lithuania | Leukemia | 0.04 | -0.21 | 0.29 |
| Female | Lithuania | Other leukemia | -2.72 | -3.37 | -2.07 |
| Both | Lithuania | Acute lymphoid leukemia | -1.72 | -2.20 | -1.24 |
| Both | Lithuania | Chronic lymphoid leukemia | 4.27 | 3.40 | 5.13 |
| Both | Lithuania | Acute myeloid leukemia | 2.17 | 1.77 | 2.56 |
| Both | Lithuania | Chronic myeloid leukemia | -2.44 | -2.90 | -1.97 |
| Both | Lithuania | Leukemia | 0.56 | 0.32 | 0.80 |
| Both | Lithuania | Other leukemia | -2.17 | -2.87 | -1.48 |
| Male | Low SDI | Acute lymphoid leukemia | -0.86 | -0.97 | -0.76 |
| Male | Low SDI | Chronic lymphoid leukemia | 1.23 | 1.17 | 1.28 |
| Male | Low SDI | Acute myeloid leukemia | 0.59 | 0.54 | 0.64 |
| Male | Low SDI | Chronic myeloid leukemia | -0.43 | -0.50 | -0.36 |
| Male | Low SDI | Leukemia | -0.23 | -0.28 | -0.18 |
| Male | Low SDI | Other leukemia | -0.80 | -0.86 | -0.73 |
| Female | Low SDI | Acute lymphoid leukemia | 0.12 | 0.04 | 0.19 |
| Female | Low SDI | Chronic lymphoid leukemia | -0.08 | -0.19 | 0.02 |
| Female | Low SDI | Acute myeloid leukemia | 0.04 | 0.00 | 0.09 |
| Female | Low SDI | Chronic myeloid leukemia | -1.37 | -1.45 | -1.28 |
| Female | Low SDI | Leukemia | -0.89 | -0.95 | -0.83 |
| Female | Low SDI | Other leukemia | -1.72 | -1.81 | -1.63 |
| Both | Low SDI | Acute lymphoid leukemia | -0.52 | -0.60 | -0.43 |
| Both | Low SDI | Chronic lymphoid leukemia | 0.62 | 0.57 | 0.67 |
| Both | Low SDI | Acute myeloid leukemia | 0.34 | 0.29 | 0.38 |
| Both | Low SDI | Chronic myeloid leukemia | -0.93 | -0.97 | -0.88 |
| Both | Low SDI | Leukemia | -0.55 | -0.59 | -0.50 |
| Both | Low SDI | Other leukemia | -1.27 | -1.33 | -1.20 |
| Male | Low-middle SDI | Acute lymphoid leukemia | -0.05 | -0.16 | 0.06 |
| Male | Low-middle SDI | Chronic lymphoid leukemia | 1.73 | 1.64 | 1.82 |
| Male | Low-middle SDI | Acute myeloid leukemia | 0.96 | 0.91 | 1.01 |

| | | | | | |
|---|---|---|---|---|---|
| Male | Low-middle SDI | Chronic myeloid leukemia | -0.02 | -0.13 | 0.09 |
| Male | Low-middle SDI | Leukemia | -0.08 | -0.14 | -0.03 |
| Male | Low-middle SDI | Other leukemia | -0.87 | -0.94 | -0.79 |
| Female | Low-middle SDI | Acute lymphoid leukemia | 0.70 | 0.57 | 0.84 |
| Female | Low-middle SDI | Chronic lymphoid leukemia | 0.86 | 0.71 | 1.01 |
| Female | Low-middle SDI | Acute myeloid leukemia | 0.70 | 0.62 | 0.78 |
| Female | Low-middle SDI | Chronic myeloid leukemia | -0.89 | -1.03 | -0.74 |
| Female | Low-middle SDI | Leukemia | -0.60 | -0.66 | -0.53 |
| Female | Low-middle SDI | Other leukemia | -1.66 | -1.77 | -1.55 |
| Both | Low-middle SDI | Acute lymphoid leukemia | 0.23 | 0.12 | 0.34 |
| Both | Low-middle SDI | Chronic lymphoid leukemia | 1.31 | 1.20 | 1.41 |
| Both | Low-middle SDI | Acute myeloid leukemia | 0.82 | 0.77 | 0.88 |
| Both | Low-middle SDI | Chronic myeloid leukemia | -0.48 | -0.60 | -0.36 |
| Both | Low-middle SDI | Leukemia | -0.34 | -0.40 | -0.28 |
| Both | Low-middle SDI | Other leukemia | -1.26 | -1.35 | -1.17 |
| Male | Luxembourg | Acute lymphoid leukemia | -3.17 | -3.38 | -2.96 |
| Male | Luxembourg | Chronic lymphoid leukemia | 0.27 | -0.13 | 0.67 |
| Male | Luxembourg | Acute myeloid leukemia | -0.25 | -0.37 | -0.14 |
| Male | Luxembourg | Chronic myeloid leukemia | -3.68 | -4.05 | -3.31 |
| Male | Luxembourg | Leukemia | -1.11 | -1.27 | -0.95 |
| Male | Luxembourg | Other leukemia | -1.79 | -1.91 | -1.66 |
| Female | Luxembourg | Acute lymphoid leukemia | -1.67 | -1.89 | -1.45 |
| Female | Luxembourg | Chronic lymphoid leukemia | 1.03 | 0.70 | 1.36 |
| Female | Luxembourg | Acute myeloid leukemia | 0.27 | 0.16 | 0.38 |
| Female | Luxembourg | Chronic myeloid leukemia | -3.93 | -4.23 | -3.64 |
| Female | Luxembourg | Leukemia | -0.41 | -0.50 | -0.31 |
| Female | Luxembourg | Other leukemia | -1.62 | -1.83 | -1.40 |
| Both | Luxembourg | Acute lymphoid leukemia | -2.59 | -2.80 | -2.39 |
| Both | Luxembourg | Chronic lymphoid leukemia | 0.70 | 0.34 | 1.05 |
| Both | Luxembourg | Acute myeloid leukemia | 0.02 | -0.06 | 0.09 |
| Both | Luxembourg | Chronic myeloid leukemia | -3.66 | -3.99 | -3.34 |
| Both | Luxembourg | Leukemia | -0.75 | -0.86 | -0.64 |
| Both | Luxembourg | Other leukemia | -1.67 | -1.84 | -1.49 |
| Male | Macedonia | Acute lymphoid leukemia | -1.18 | -1.36 | -1.01 |
| Male | Macedonia | Chronic lymphoid leukemia | 1.61 | 1.46 | 1.76 |
| Male | Macedonia | Acute myeloid leukemia | 0.42 | 0.32 | 0.51 |
| Male | Macedonia | Chronic myeloid leukemia | -1.24 | -1.48 | -1.00 |
| Male | Macedonia | Leukemia | 0.18 | 0.08 | 0.28 |
| Male | Macedonia | Other leukemia | -0.08 | -0.28 | 0.13 |
| Female | Macedonia | Acute lymphoid leukemia | -0.44 | -0.70 | -0.18 |
| Female | Macedonia | Chronic lymphoid leukemia | 1.09 | 0.88 | 1.30 |
| Female | Macedonia | Acute myeloid leukemia | 0.25 | 0.08 | 0.42 |
| Female | Macedonia | Chronic myeloid leukemia | -2.28 | -2.50 | -2.07 |
| Female | Macedonia | Leukemia | 0.17 | 0.03 | 0.32 |
| Female | Macedonia | Other leukemia | 0.68 | 0.44 | 0.92 |
| Both | Macedonia | Acute lymphoid leukemia | -0.91 | -1.10 | -0.71 |
| Both | Macedonia | Chronic lymphoid leukemia | 1.36 | 1.22 | 1.51 |
| Both | Macedonia | Acute myeloid leukemia | 0.30 | 0.19 | 0.41 |

| | | | | | |
|---|---|---|---|---|---|
| Both | Macedonia | Chronic myeloid leukemia | -1.78 | -1.98 | -1.59 |
| Both | Macedonia | Leukemia | 0.12 | 0.02 | 0.22 |
| Both | Macedonia | Other leukemia | 0.22 | 0.01 | 0.43 |
| Male | Madagascar | Acute lymphoid leukemia | -2.21 | -2.73 | -1.68 |
| Male | Madagascar | Chronic lymphoid leukemia | 2.95 | 2.68 | 3.21 |
| Male | Madagascar | Acute myeloid leukemia | 0.12 | 0.03 | 0.21 |
| Male | Madagascar | Chronic myeloid leukemia | -0.22 | -0.33 | -0.11 |
| Male | Madagascar | Leukemia | -0.63 | -0.82 | -0.45 |
| Male | Madagascar | Other leukemia | -1.54 | -1.76 | -1.32 |
| Female | Madagascar | Acute lymphoid leukemia | 0.35 | 0.19 | 0.51 |
| Female | Madagascar | Chronic lymphoid leukemia | 0.69 | 0.62 | 0.77 |
| Female | Madagascar | Acute myeloid leukemia | 0.66 | 0.54 | 0.77 |
| Female | Madagascar | Chronic myeloid leukemia | -0.41 | -0.50 | -0.31 |
| Female | Madagascar | Leukemia | -0.28 | -0.36 | -0.19 |
| Female | Madagascar | Other leukemia | -1.02 | -1.10 | -0.94 |
| Both | Madagascar | Acute lymphoid leukemia | -1.25 | -1.64 | -0.86 |
| Both | Madagascar | Chronic lymphoid leukemia | 1.68 | 1.54 | 1.81 |
| Both | Madagascar | Acute myeloid leukemia | 0.12 | 0.03 | 0.21 |
| Both | Madagascar | Chronic myeloid leukemia | -0.31 | -0.40 | -0.21 |
| Both | Madagascar | Leukemia | -0.50 | -0.64 | -0.36 |
| Both | Madagascar | Other leukemia | -1.25 | -1.39 | -1.11 |
| Male | Malawi | Acute lymphoid leukemia | 0.04 | -0.23 | 0.31 |
| Male | Malawi | Chronic lymphoid leukemia | 2.50 | 2.38 | 2.63 |
| Male | Malawi | Acute myeloid leukemia | 0.52 | 0.33 | 0.72 |
| Male | Malawi | Chronic myeloid leukemia | 0.04 | -0.18 | 0.27 |
| Male | Malawi | Leukemia | 0.19 | 0.00 | 0.38 |
| Male | Malawi | Other leukemia | -0.81 | -1.04 | -0.57 |
| Female | Malawi | Acute lymphoid leukemia | 1.23 | 0.98 | 1.49 |
| Female | Malawi | Chronic lymphoid leukemia | -0.40 | -0.61 | -0.18 |
| Female | Malawi | Acute myeloid leukemia | 0.39 | 0.21 | 0.58 |
| Female | Malawi | Chronic myeloid leukemia | -1.89 | -2.21 | -1.57 |
| Female | Malawi | Leukemia | -0.94 | -1.19 | -0.68 |
| Female | Malawi | Other leukemia | -1.96 | -2.26 | -1.66 |
| Both | Malawi | Acute lymphoid leukemia | 0.46 | 0.20 | 0.73 |
| Both | Malawi | Chronic lymphoid leukemia | 0.83 | 0.72 | 0.94 |
| Both | Malawi | Acute myeloid leukemia | 0.31 | 0.10 | 0.51 |
| Both | Malawi | Chronic myeloid leukemia | -1.03 | -1.28 | -0.78 |
| Both | Malawi | Leukemia | -0.36 | -0.57 | -0.15 |
| Both | Malawi | Other leukemia | -1.42 | -1.68 | -1.17 |
| Male | Malaysia | Acute lymphoid leukemia | -0.54 | -0.66 | -0.43 |
| Male | Malaysia | Chronic lymphoid leukemia | 2.61 | 2.52 | 2.70 |
| Male | Malaysia | Acute myeloid leukemia | 0.87 | 0.80 | 0.94 |
| Male | Malaysia | Chronic myeloid leukemia | 0.11 | 0.02 | 0.20 |
| Male | Malaysia | Leukemia | 0.02 | -0.07 | 0.10 |
| Male | Malaysia | Other leukemia | -0.75 | -0.90 | -0.59 |
| Female | Malaysia | Acute lymphoid leukemia | -0.95 | -1.03 | -0.88 |
| Female | Malaysia | Chronic lymphoid leukemia | 3.00 | 2.88 | 3.12 |
| Female | Malaysia | Acute myeloid leukemia | 0.40 | 0.32 | 0.48 |

| | | | | | |
|---|---|---|---|---|---|
| Female | Malaysia | Chronic myeloid leukemia | -0.56 | -0.67 | -0.45 |
| Female | Malaysia | Leukemia | -0.31 | -0.37 | -0.25 |
| Female | Malaysia | Other leukemia | -1.61 | -1.70 | -1.51 |
| Both | Malaysia | Acute lymphoid leukemia | -0.66 | -0.74 | -0.57 |
| Both | Malaysia | Chronic lymphoid leukemia | 2.83 | 2.74 | 2.91 |
| Both | Malaysia | Acute myeloid leukemia | 0.56 | 0.50 | 0.63 |
| Both | Malaysia | Chronic myeloid leukemia | 0.06 | -0.02 | 0.15 |
| Both | Malaysia | Leukemia | -0.11 | -0.17 | -0.04 |
| Both | Malaysia | Other leukemia | -1.10 | -1.22 | -0.97 |
| Male | Maldives | Acute lymphoid leukemia | -1.11 | -1.33 | -0.89 |
| Male | Maldives | Chronic lymphoid leukemia | 3.94 | 3.76 | 4.12 |
| Male | Maldives | Acute myeloid leukemia | 0.89 | 0.75 | 1.02 |
| Male | Maldives | Chronic myeloid leukemia | -1.25 | -1.36 | -1.13 |
| Male | Maldives | Leukemia | -0.79 | -0.93 | -0.66 |
| Male | Maldives | Other leukemia | -1.71 | -1.85 | -1.57 |
| Female | Maldives | Acute lymphoid leukemia | 0.53 | 0.40 | 0.66 |
| Female | Maldives | Chronic lymphoid leukemia | 3.09 | 2.97 | 3.21 |
| Female | Maldives | Acute myeloid leukemia | 0.39 | 0.07 | 0.72 |
| Female | Maldives | Chronic myeloid leukemia | -2.19 | -2.37 | -2.01 |
| Female | Maldives | Leukemia | -1.28 | -1.52 | -1.05 |
| Female | Maldives | Other leukemia | -1.43 | -1.69 | -1.18 |
| Both | Maldives | Acute lymphoid leukemia | -1.16 | -1.35 | -0.98 |
| Both | Maldives | Chronic lymphoid leukemia | 3.43 | 3.31 | 3.55 |
| Both | Maldives | Acute myeloid leukemia | 0.59 | 0.48 | 0.69 |
| Both | Maldives | Chronic myeloid leukemia | -1.84 | -1.95 | -1.73 |
| Both | Maldives | Leukemia | -1.05 | -1.16 | -0.94 |
| Both | Maldives | Other leukemia | -1.58 | -1.72 | -1.44 |
| Male | Mali | Acute lymphoid leukemia | -1.79 | -2.05 | -1.54 |
| Male | Mali | Chronic lymphoid leukemia | 1.39 | 1.33 | 1.45 |
| Male | Mali | Acute myeloid leukemia | 0.36 | 0.28 | 0.44 |
| Male | Mali | Chronic myeloid leukemia | 0.06 | -0.04 | 0.17 |
| Male | Mali | Leukemia | -0.02 | -0.12 | 0.08 |
| Male | Mali | Other leukemia | 0.08 | -0.02 | 0.18 |
| Female | Mali | Acute lymphoid leukemia | -0.19 | -0.33 | -0.05 |
| Female | Mali | Chronic lymphoid leukemia | -0.36 | -0.43 | -0.28 |
| Female | Mali | Acute myeloid leukemia | -0.07 | -0.17 | 0.02 |
| Female | Mali | Chronic myeloid leukemia | -1.60 | -1.71 | -1.48 |
| Female | Mali | Leukemia | -0.92 | -1.05 | -0.80 |
| Female | Mali | Other leukemia | -1.49 | -1.64 | -1.33 |
| Both | Mali | Acute lymphoid leukemia | -1.24 | -1.45 | -1.03 |
| Both | Mali | Chronic lymphoid leukemia | 0.32 | 0.29 | 0.36 |
| Both | Mali | Acute myeloid leukemia | 0.24 | 0.15 | 0.32 |
| Both | Mali | Chronic myeloid leukemia | -0.76 | -0.85 | -0.67 |
| Both | Mali | Leukemia | -0.39 | -0.50 | -0.29 |
| Both | Mali | Other leukemia | -0.62 | -0.74 | -0.51 |
| Male | Malta | Acute lymphoid leukemia | -1.48 | -1.67 | -1.30 |
| Male | Malta | Chronic lymphoid leukemia | 0.66 | 0.48 | 0.84 |
| Male | Malta | Acute myeloid leukemia | 0.75 | 0.57 | 0.94 |

| | | | | | |
|---|---|---|---|---|---|
| Male | Malta | Chronic myeloid leukemia | -2.69 | -2.94 | -2.45 |
| Male | Malta | Leukemia | -0.16 | -0.30 | -0.01 |
| Male | Malta | Other leukemia | -0.27 | -0.63 | 0.09 |
| Female | Malta | Acute lymphoid leukemia | -1.59 | -1.68 | -1.51 |
| Female | Malta | Chronic lymphoid leukemia | 0.59 | 0.49 | 0.69 |
| Female | Malta | Acute myeloid leukemia | 1.03 | 0.87 | 1.20 |
| Female | Malta | Chronic myeloid leukemia | -3.58 | -3.77 | -3.39 |
| Female | Malta | Leukemia | -0.23 | -0.32 | -0.14 |
| Female | Malta | Other leukemia | -1.04 | -1.36 | -0.72 |
| Both | Malta | Acute lymphoid leukemia | -1.50 | -1.62 | -1.39 |
| Both | Malta | Chronic lymphoid leukemia | 0.64 | 0.52 | 0.76 |
| Both | Malta | Acute myeloid leukemia | 0.87 | 0.72 | 1.03 |
| Both | Malta | Chronic myeloid leukemia | -3.00 | -3.21 | -2.79 |
| Both | Malta | Leukemia | -0.19 | -0.28 | -0.09 |
| Both | Malta | Other leukemia | -0.65 | -0.98 | -0.32 |
| Male | Marshall Islands | Acute lymphoid leukemia | 0.22 | 0.07 | 0.37 |
| Male | Marshall Islands | Chronic lymphoid leukemia | -0.18 | -0.33 | -0.03 |
| Male | Marshall Islands | Acute myeloid leukemia | 0.94 | 0.80 | 1.08 |
| Male | Marshall Islands | Chronic myeloid leukemia | -0.86 | -0.91 | -0.81 |
| Male | Marshall Islands | Leukemia | 0.31 | 0.20 | 0.42 |
| Male | Marshall Islands | Other leukemia | 0.13 | 0.02 | 0.24 |
| Female | Marshall Islands | Acute lymphoid leukemia | 0.18 | -0.08 | 0.43 |
| Female | Marshall Islands | Chronic lymphoid leukemia | 1.06 | 0.98 | 1.13 |
| Female | Marshall Islands | Acute myeloid leukemia | -0.19 | -0.39 | 0.02 |
| Female | Marshall Islands | Chronic myeloid leukemia | -0.99 | -1.21 | -0.78 |
| Female | Marshall Islands | Leukemia | -0.14 | -0.31 | 0.02 |
| Female | Marshall Islands | Other leukemia | -0.41 | -0.58 | -0.23 |
| Both | Marshall Islands | Acute lymphoid leukemia | 0.20 | 0.01 | 0.39 |
| Both | Marshall Islands | Chronic lymphoid leukemia | 0.83 | 0.78 | 0.88 |
| Both | Marshall Islands | Acute myeloid leukemia | 0.60 | 0.44 | 0.76 |
| Both | Marshall Islands | Chronic myeloid leukemia | -0.93 | -1.06 | -0.81 |
| Both | Marshall Islands | Leukemia | 0.14 | 0.00 | 0.27 |
| Both | Marshall Islands | Other leukemia | -0.04 | -0.18 | 0.10 |
| Male | Mauritania | Acute lymphoid leukemia | -1.01 | -1.17 | -0.85 |
| Male | Mauritania | Chronic lymphoid leukemia | 1.90 | 1.79 | 2.02 |
| Male | Mauritania | Acute myeloid leukemia | 1.08 | 1.01 | 1.14 |
| Male | Mauritania | Chronic myeloid leukemia | 0.13 | 0.04 | 0.21 |
| Male | Mauritania | Leukemia | 0.50 | 0.45 | 0.55 |
| Male | Mauritania | Other leukemia | 0.36 | 0.27 | 0.44 |
| Female | Mauritania | Acute lymphoid leukemia | 0.88 | 0.79 | 0.98 |
| Female | Mauritania | Chronic lymphoid leukemia | 0.03 | -0.02 | 0.08 |
| Female | Mauritania | Acute myeloid leukemia | 0.67 | 0.62 | 0.72 |
| Female | Mauritania | Chronic myeloid leukemia | -0.99 | -1.02 | -0.97 |
| Female | Mauritania | Leukemia | -0.21 | -0.24 | -0.19 |
| Female | Mauritania | Other leukemia | -1.01 | -1.07 | -0.96 |
| Both | Mauritania | Acute lymphoid leukemia | -0.25 | -0.35 | -0.14 |
| Both | Mauritania | Chronic lymphoid leukemia | 0.64 | 0.55 | 0.73 |
| Both | Mauritania | Acute myeloid leukemia | 0.94 | 0.88 | 0.99 |

| | | | | | |
|---|---|---|---|---|---|
| Both | Mauritania | Chronic myeloid leukemia | -0.30 | -0.36 | -0.24 |
| Both | Mauritania | Leukemia | 0.21 | 0.18 | 0.25 |
| Both | Mauritania | Other leukemia | -0.23 | -0.27 | -0.19 |
| Male | Mauritius | Acute lymphoid leukemia | 2.13 | 1.28 | 2.99 |
| Male | Mauritius | Chronic lymphoid leukemia | 0.96 | 0.27 | 1.64 |
| Male | Mauritius | Acute myeloid leukemia | 1.35 | 0.48 | 2.22 |
| Male | Mauritius | Chronic myeloid leukemia | 0.20 | -0.24 | 0.63 |
| Male | Mauritius | Leukemia | -0.52 | -0.72 | -0.33 |
| Male | Mauritius | Other leukemia | -1.37 | -1.62 | -1.11 |
| Female | Mauritius | Acute lymphoid leukemia | 0.44 | -0.54 | 1.42 |
| Female | Mauritius | Chronic lymphoid leukemia | -0.25 | -0.63 | 0.13 |
| Female | Mauritius | Acute myeloid leukemia | 1.96 | 1.01 | 2.91 |
| Female | Mauritius | Chronic myeloid leukemia | 0.96 | 0.35 | 1.58 |
| Female | Mauritius | Leukemia | -0.43 | -0.71 | -0.16 |
| Female | Mauritius | Other leukemia | -1.20 | -1.54 | -0.85 |
| Both | Mauritius | Acute lymphoid leukemia | 1.60 | 0.80 | 2.42 |
| Both | Mauritius | Chronic lymphoid leukemia | 0.27 | -0.08 | 0.63 |
| Both | Mauritius | Acute myeloid leukemia | 1.67 | 0.81 | 2.54 |
| Both | Mauritius | Chronic myeloid leukemia | 0.65 | 0.17 | 1.12 |
| Both | Mauritius | Leukemia | -0.47 | -0.68 | -0.26 |
| Both | Mauritius | Other leukemia | -1.29 | -1.56 | -1.01 |
| Male | Mexico | Acute lymphoid leukemia | 1.33 | 1.05 | 1.61 |
| Male | Mexico | Chronic lymphoid leukemia | 1.81 | 1.66 | 1.97 |
| Male | Mexico | Acute myeloid leukemia | 0.19 | 0.05 | 0.33 |
| Male | Mexico | Chronic myeloid leukemia | -1.09 | -1.44 | -0.74 |
| Male | Mexico | Leukemia | -0.03 | -0.15 | 0.09 |
| Male | Mexico | Other leukemia | -2.12 | -2.24 | -2.00 |
| Female | Mexico | Acute lymphoid leukemia | 1.21 | 0.91 | 1.51 |
| Female | Mexico | Chronic lymphoid leukemia | 1.21 | 1.00 | 1.42 |
| Female | Mexico | Acute myeloid leukemia | 0.71 | 0.60 | 0.81 |
| Female | Mexico | Chronic myeloid leukemia | -1.92 | -2.38 | -1.46 |
| Female | Mexico | Leukemia | -0.06 | -0.19 | 0.07 |
| Female | Mexico | Other leukemia | -2.22 | -2.34 | -2.09 |
| Both | Mexico | Acute lymphoid leukemia | 1.27 | 0.99 | 1.56 |
| Both | Mexico | Chronic lymphoid leukemia | 1.55 | 1.39 | 1.71 |
| Both | Mexico | Acute myeloid leukemia | 0.44 | 0.32 | 0.55 |
| Both | Mexico | Chronic myeloid leukemia | -1.44 | -1.83 | -1.05 |
| Both | Mexico | Leukemia | -0.05 | -0.17 | 0.07 |
| Both | Mexico | Other leukemia | -2.17 | -2.29 | -2.06 |
| Male | Middle SDI | Acute lymphoid leukemia | 0.81 | 0.71 | 0.91 |
| Male | Middle SDI | Chronic lymphoid leukemia | 3.58 | 3.20 | 3.95 |
| Male | Middle SDI | Acute myeloid leukemia | 1.08 | 1.03 | 1.12 |
| Male | Middle SDI | Chronic myeloid leukemia | -0.12 | -0.22 | -0.03 |
| Male | Middle SDI | Leukemia | 0.01 | -0.16 | 0.18 |
| Male | Middle SDI | Other leukemia | -0.87 | -1.10 | -0.64 |
| Female | Middle SDI | Acute lymphoid leukemia | 0.85 | 0.77 | 0.93 |
| Female | Middle SDI | Chronic lymphoid leukemia | 3.29 | 2.79 | 3.79 |
| Female | Middle SDI | Acute myeloid leukemia | 0.99 | 0.93 | 1.05 |

| | | | | | |
|---|---|---|---|---|---|
| Female | Middle SDI | Chronic myeloid leukemia | -1.15 | -1.27 | -1.03 |
| Female | Middle SDI | Leukemia | -0.42 | -0.55 | -0.29 |
| Female | Middle SDI | Other leukemia | -1.36 | -1.51 | -1.20 |
| Both | Middle SDI | Acute lymphoid leukemia | 0.81 | 0.73 | 0.90 |
| Both | Middle SDI | Chronic lymphoid leukemia | 3.48 | 3.06 | 3.91 |
| Both | Middle SDI | Acute myeloid leukemia | 1.03 | 0.99 | 1.07 |
| Both | Middle SDI | Chronic myeloid leukemia | -0.56 | -0.66 | -0.45 |
| Both | Middle SDI | Leukemia | -0.19 | -0.34 | -0.04 |
| Both | Middle SDI | Other leukemia | -1.11 | -1.30 | -0.91 |
| Male | Moldova | Acute lymphoid leukemia | -3.71 | -3.90 | -3.51 |
| Male | Moldova | Chronic lymphoid leukemia | 0.57 | 0.27 | 0.88 |
| Male | Moldova | Acute myeloid leukemia | -0.66 | -1.04 | -0.27 |
| Male | Moldova | Chronic myeloid leukemia | -2.08 | -2.37 | -1.80 |
| Male | Moldova | Leukemia | -1.68 | -1.95 | -1.41 |
| Male | Moldova | Other leukemia | -1.32 | -1.75 | -0.88 |
| Female | Moldova | Acute lymphoid leukemia | -3.32 | -3.55 | -3.09 |
| Female | Moldova | Chronic lymphoid leukemia | 0.05 | -0.28 | 0.38 |
| Female | Moldova | Acute myeloid leukemia | -1.64 | -1.94 | -1.34 |
| Female | Moldova | Chronic myeloid leukemia | -2.13 | -2.50 | -1.77 |
| Female | Moldova | Leukemia | -1.94 | -2.18 | -1.69 |
| Female | Moldova | Other leukemia | -1.79 | -2.25 | -1.32 |
| Both | Moldova | Acute lymphoid leukemia | -3.60 | -3.80 | -3.41 |
| Both | Moldova | Chronic lymphoid leukemia | 0.38 | 0.10 | 0.66 |
| Both | Moldova | Acute myeloid leukemia | -1.11 | -1.43 | -0.78 |
| Both | Moldova | Chronic myeloid leukemia | -2.04 | -2.32 | -1.76 |
| Both | Moldova | Leukemia | -1.81 | -2.06 | -1.56 |
| Both | Moldova | Other leukemia | -1.55 | -1.98 | -1.12 |
| Male | Mongolia | Acute lymphoid leukemia | -1.77 | -2.02 | -1.53 |
| Male | Mongolia | Chronic lymphoid leukemia | 1.33 | 1.21 | 1.44 |
| Male | Mongolia | Acute myeloid leukemia | -0.61 | -0.81 | -0.40 |
| Male | Mongolia | Chronic myeloid leukemia | -1.31 | -1.56 | -1.06 |
| Male | Mongolia | Leukemia | -0.97 | -1.17 | -0.76 |
| Male | Mongolia | Other leukemia | -0.65 | -0.83 | -0.47 |
| Female | Mongolia | Acute lymphoid leukemia | 1.10 | 0.79 | 1.40 |
| Female | Mongolia | Chronic lymphoid leukemia | 1.66 | 1.39 | 1.94 |
| Female | Mongolia | Acute myeloid leukemia | 0.74 | 0.52 | 0.96 |
| Female | Mongolia | Chronic myeloid leukemia | -1.37 | -1.79 | -0.96 |
| Female | Mongolia | Leukemia | 0.42 | 0.19 | 0.65 |
| Female | Mongolia | Other leukemia | -0.97 | -1.21 | -0.72 |
| Both | Mongolia | Acute lymphoid leukemia | -0.87 | -1.07 | -0.67 |
| Both | Mongolia | Chronic lymphoid leukemia | 1.43 | 1.24 | 1.62 |
| Both | Mongolia | Acute myeloid leukemia | 0.22 | 0.04 | 0.39 |
| Both | Mongolia | Chronic myeloid leukemia | -1.44 | -1.72 | -1.16 |
| Both | Mongolia | Leukemia | -0.34 | -0.53 | -0.15 |
| Both | Mongolia | Other leukemia | -0.86 | -1.07 | -0.65 |
| Male | Montenegro | Acute lymphoid leukemia | -1.56 | -1.82 | -1.30 |
| Male | Montenegro | Chronic lymphoid leukemia | 0.55 | 0.48 | 0.62 |
| Male | Montenegro | Acute myeloid leukemia | -0.66 | -0.83 | -0.49 |

| | | | | | |
|---|---|---|---|---|---|
| Male | Montenegro | Chronic myeloid leukemia | -1.31 | -1.54 | -1.09 |
| Male | Montenegro | Leukemia | -0.54 | -0.65 | -0.44 |
| Male | Montenegro | Other leukemia | -1.36 | -1.49 | -1.24 |
| Female | Montenegro | Acute lymphoid leukemia | -1.37 | -1.53 | -1.20 |
| Female | Montenegro | Chronic lymphoid leukemia | 1.10 | 1.01 | 1.20 |
| Female | Montenegro | Acute myeloid leukemia | -0.68 | -0.83 | -0.53 |
| Female | Montenegro | Chronic myeloid leukemia | -1.59 | -1.99 | -1.19 |
| Female | Montenegro | Leukemia | -0.47 | -0.61 | -0.34 |
| Female | Montenegro | Other leukemia | -1.18 | -1.31 | -1.05 |
| Both | Montenegro | Acute lymphoid leukemia | -1.50 | -1.70 | -1.30 |
| Both | Montenegro | Chronic lymphoid leukemia | 0.82 | 0.78 | 0.87 |
| Both | Montenegro | Acute myeloid leukemia | -0.65 | -0.80 | -0.50 |
| Both | Montenegro | Chronic myeloid leukemia | -1.44 | -1.73 | -1.14 |
| Both | Montenegro | Leukemia | -0.50 | -0.61 | -0.39 |
| Both | Montenegro | Other leukemia | -1.28 | -1.38 | -1.18 |
| Male | Morocco | Acute lymphoid leukemia | -0.07 | -0.16 | 0.02 |
| Male | Morocco | Chronic lymphoid leukemia | 0.95 | 0.83 | 1.08 |
| Male | Morocco | Acute myeloid leukemia | 0.08 | -0.06 | 0.21 |
| Male | Morocco | Chronic myeloid leukemia | -0.27 | -0.42 | -0.12 |
| Male | Morocco | Leukemia | 0.16 | 0.11 | 0.22 |
| Male | Morocco | Other leukemia | 0.24 | 0.19 | 0.30 |
| Female | Morocco | Acute lymphoid leukemia | 0.47 | 0.40 | 0.54 |
| Female | Morocco | Chronic lymphoid leukemia | 1.12 | 0.99 | 1.24 |
| Female | Morocco | Acute myeloid leukemia | 0.03 | -0.01 | 0.07 |
| Female | Morocco | Chronic myeloid leukemia | -1.78 | -1.84 | -1.72 |
| Female | Morocco | Leukemia | -0.27 | -0.31 | -0.22 |
| Female | Morocco | Other leukemia | -0.47 | -0.53 | -0.40 |
| Both | Morocco | Acute lymphoid leukemia | 0.12 | 0.06 | 0.19 |
| Both | Morocco | Chronic lymphoid leukemia | 0.98 | 0.85 | 1.10 |
| Both | Morocco | Acute myeloid leukemia | 0.06 | -0.02 | 0.14 |
| Both | Morocco | Chronic myeloid leukemia | -1.03 | -1.12 | -0.94 |
| Both | Morocco | Leukemia | -0.03 | -0.05 | 0.00 |
| Both | Morocco | Other leukemia | -0.07 | -0.10 | -0.03 |
| Male | Mozambique | Acute lymphoid leukemia | -0.05 | -0.54 | 0.44 |
| Male | Mozambique | Chronic lymphoid leukemia | 2.64 | 2.40 | 2.88 |
| Male | Mozambique | Acute myeloid leukemia | 0.59 | 0.35 | 0.83 |
| Male | Mozambique | Chronic myeloid leukemia | 0.88 | 0.68 | 1.08 |
| Male | Mozambique | Leukemia | 0.12 | -0.15 | 0.39 |
| Male | Mozambique | Other leukemia | -1.05 | -1.27 | -0.84 |
| Female | Mozambique | Acute lymphoid leukemia | 0.84 | 0.38 | 1.30 |
| Female | Mozambique | Chronic lymphoid leukemia | 0.65 | 0.47 | 0.82 |
| Female | Mozambique | Acute myeloid leukemia | 0.08 | -0.36 | 0.51 |
| Female | Mozambique | Chronic myeloid leukemia | -1.53 | -1.81 | -1.25 |
| Female | Mozambique | Leukemia | -1.05 | -1.38 | -0.72 |
| Female | Mozambique | Other leukemia | -2.26 | -2.54 | -1.98 |
| Both | Mozambique | Acute lymphoid leukemia | 0.24 | -0.24 | 0.72 |
| Both | Mozambique | Chronic lymphoid leukemia | 1.47 | 1.30 | 1.65 |
| Both | Mozambique | Acute myeloid leukemia | 0.36 | 0.07 | 0.65 |

| | | | | | |
|---|---|---|---|---|---|
| Both | Mozambique | Chronic myeloid leukemia | -0.48 | -0.71 | -0.25 |
| Both | Mozambique | Leukemia | -0.43 | -0.73 | -0.14 |
| Both | Mozambique | Other leukemia | -1.70 | -1.94 | -1.46 |
| Male | Myanmar | Acute lymphoid leukemia | 1.19 | 0.89 | 1.49 |
| Male | Myanmar | Chronic lymphoid leukemia | 2.76 | 2.58 | 2.95 |
| Male | Myanmar | Acute myeloid leukemia | 1.98 | 1.85 | 2.10 |
| Male | Myanmar | Chronic myeloid leukemia | -0.78 | -0.89 | -0.67 |
| Male | Myanmar | Leukemia | 0.08 | -0.03 | 0.18 |
| Male | Myanmar | Other leukemia | -0.61 | -0.70 | -0.52 |
| Female | Myanmar | Acute lymphoid leukemia | 1.01 | 0.74 | 1.27 |
| Female | Myanmar | Chronic lymphoid leukemia | 1.17 | 1.03 | 1.31 |
| Female | Myanmar | Acute myeloid leukemia | 0.34 | 0.11 | 0.58 |
| Female | Myanmar | Chronic myeloid leukemia | -1.92 | -2.22 | -1.61 |
| Female | Myanmar | Leukemia | -1.19 | -1.43 | -0.96 |
| Female | Myanmar | Other leukemia | -1.88 | -2.13 | -1.62 |
| Both | Myanmar | Acute lymphoid leukemia | 1.08 | 0.79 | 1.36 |
| Both | Myanmar | Chronic lymphoid leukemia | 1.93 | 1.79 | 2.07 |
| Both | Myanmar | Acute myeloid leukemia | 0.98 | 0.79 | 1.17 |
| Both | Myanmar | Chronic myeloid leukemia | -1.45 | -1.68 | -1.23 |
| Both | Myanmar | Leukemia | -0.64 | -0.82 | -0.46 |
| Both | Myanmar | Other leukemia | -1.33 | -1.52 | -1.15 |
| Male | Namibia | Acute lymphoid leukemia | -0.01 | -0.32 | 0.30 |
| Male | Namibia | Chronic lymphoid leukemia | 0.98 | 0.80 | 1.15 |
| Male | Namibia | Acute myeloid leukemia | -0.01 | -0.29 | 0.27 |
| Male | Namibia | Chronic myeloid leukemia | -0.67 | -1.02 | -0.31 |
| Male | Namibia | Leukemia | -0.21 | -0.49 | 0.07 |
| Male | Namibia | Other leukemia | -0.72 | -1.03 | -0.41 |
| Female | Namibia | Acute lymphoid leukemia | 0.48 | 0.14 | 0.83 |
| Female | Namibia | Chronic lymphoid leukemia | 0.11 | -0.40 | 0.61 |
| Female | Namibia | Acute myeloid leukemia | 0.45 | 0.01 | 0.90 |
| Female | Namibia | Chronic myeloid leukemia | -2.03 | -2.66 | -1.40 |
| Female | Namibia | Leukemia | -1.40 | -1.88 | -0.92 |
| Female | Namibia | Other leukemia | -1.77 | -2.26 | -1.27 |
| Both | Namibia | Acute lymphoid leukemia | 0.26 | -0.07 | 0.59 |
| Both | Namibia | Chronic lymphoid leukemia | 0.54 | 0.26 | 0.82 |
| Both | Namibia | Acute myeloid leukemia | -0.07 | -0.38 | 0.25 |
| Both | Namibia | Chronic myeloid leukemia | -1.50 | -2.02 | -0.97 |
| Both | Namibia | Leukemia | -0.82 | -1.21 | -0.43 |
| Both | Namibia | Other leukemia | -1.38 | -1.82 | -0.94 |
| Male | Nepal | Acute lymphoid leukemia | -0.43 | -0.64 | -0.22 |
| Male | Nepal | Chronic lymphoid leukemia | 1.59 | 1.49 | 1.69 |
| Male | Nepal | Acute myeloid leukemia | 0.97 | 0.94 | 1.01 |
| Male | Nepal | Chronic myeloid leukemia | -0.53 | -0.72 | -0.34 |
| Male | Nepal | Leukemia | -0.09 | -0.25 | 0.06 |
| Male | Nepal | Other leukemia | -1.18 | -1.40 | -0.95 |
| Female | Nepal | Acute lymphoid leukemia | 0.89 | 0.74 | 1.05 |
| Female | Nepal | Chronic lymphoid leukemia | 0.38 | 0.02 | 0.74 |
| Female | Nepal | Acute myeloid leukemia | 0.19 | 0.11 | 0.28 |

| | | | | | |
|---|---|---|---|---|---|
| Female | Nepal | Chronic myeloid leukemia | -1.70 | -1.91 | -1.49 |
| Female | Nepal | Leukemia | -0.83 | -1.00 | -0.66 |
| Female | Nepal | Other leukemia | -2.22 | -2.44 | -2.00 |
| Both | Nepal | Acute lymphoid leukemia | 0.10 | -0.07 | 0.27 |
| Both | Nepal | Chronic lymphoid leukemia | 1.14 | 0.99 | 1.29 |
| Both | Nepal | Acute myeloid leukemia | 0.59 | 0.54 | 0.65 |
| Both | Nepal | Chronic myeloid leukemia | -1.16 | -1.36 | -0.95 |
| Both | Nepal | Leukemia | -0.46 | -0.62 | -0.30 |
| Both | Nepal | Other leukemia | -1.72 | -1.94 | -1.49 |
| Male | Netherlands | Acute lymphoid leukemia | -2.55 | -2.74 | -2.36 |
| Male | Netherlands | Chronic lymphoid leukemia | -3.22 | -4.42 | -2.00 |
| Male | Netherlands | Acute myeloid leukemia | 0.54 | 0.23 | 0.84 |
| Male | Netherlands | Chronic myeloid leukemia | -2.94 | -3.23 | -2.65 |
| Male | Netherlands | Leukemia | -0.18 | -0.30 | -0.06 |
| Male | Netherlands | Other leukemia | 2.77 | 2.41 | 3.13 |
| Female | Netherlands | Acute lymphoid leukemia | -2.58 | -2.72 | -2.45 |
| Female | Netherlands | Chronic lymphoid leukemia | -2.85 | -3.91 | -1.77 |
| Female | Netherlands | Acute myeloid leukemia | 1.26 | 1.03 | 1.48 |
| Female | Netherlands | Chronic myeloid leukemia | -3.39 | -3.73 | -3.06 |
| Female | Netherlands | Leukemia | -0.18 | -0.33 | -0.03 |
| Female | Netherlands | Other leukemia | 2.43 | 2.14 | 2.72 |
| Both | Netherlands | Acute lymphoid leukemia | -2.54 | -2.68 | -2.41 |
| Both | Netherlands | Chronic lymphoid leukemia | -2.98 | -4.12 | -1.82 |
| Both | Netherlands | Acute myeloid leukemia | 0.89 | 0.63 | 1.14 |
| Both | Netherlands | Chronic myeloid leukemia | -3.10 | -3.39 | -2.81 |
| Both | Netherlands | Leukemia | -0.14 | -0.26 | -0.01 |
| Both | Netherlands | Other leukemia | 2.68 | 2.37 | 3.00 |
| Male | New Zealand | Acute lymphoid leukemia | -2.44 | -2.67 | -2.21 |
| Male | New Zealand | Chronic lymphoid leukemia | -0.11 | -0.34 | 0.13 |
| Male | New Zealand | Acute myeloid leukemia | -0.64 | -0.72 | -0.55 |
| Male | New Zealand | Chronic myeloid leukemia | -4.02 | -4.29 | -3.75 |
| Male | New Zealand | Leukemia | -1.00 | -1.13 | -0.87 |
| Male | New Zealand | Other leukemia | -0.21 | -0.41 | 0.00 |
| Female | New Zealand | Acute lymphoid leukemia | -2.10 | -2.30 | -1.90 |
| Female | New Zealand | Chronic lymphoid leukemia | -0.30 | -0.52 | -0.09 |
| Female | New Zealand | Acute myeloid leukemia | 0.03 | -0.31 | 0.38 |
| Female | New Zealand | Chronic myeloid leukemia | -3.58 | -3.87 | -3.30 |
| Female | New Zealand | Leukemia | -0.69 | -0.87 | -0.51 |
| Female | New Zealand | Other leukemia | -0.39 | -0.64 | -0.15 |
| Both | New Zealand | Acute lymphoid leukemia | -2.28 | -2.48 | -2.08 |
| Both | New Zealand | Chronic lymphoid leukemia | -0.08 | -0.30 | 0.13 |
| Both | New Zealand | Acute myeloid leukemia | -0.28 | -0.42 | -0.14 |
| Both | New Zealand | Chronic myeloid leukemia | -3.76 | -4.01 | -3.51 |
| Both | New Zealand | Leukemia | -0.80 | -0.93 | -0.68 |
| Both | New Zealand | Other leukemia | -0.18 | -0.37 | 0.02 |
| Male | Nicaragua | Acute lymphoid leukemia | 0.49 | 0.33 | 0.65 |
| Male | Nicaragua | Chronic lymphoid leukemia | -0.24 | -0.57 | 0.09 |
| Male | Nicaragua | Acute myeloid leukemia | -0.30 | -0.45 | -0.15 |

| | | | | | |
|---|---|---|---|---|---|
| Male | Nicaragua | Chronic myeloid leukemia | -0.68 | -0.85 | -0.50 |
| Male | Nicaragua | Leukemia | -0.69 | -0.79 | -0.59 |
| Male | Nicaragua | Other leukemia | -2.30 | -2.50 | -2.11 |
| Female | Nicaragua | Acute lymphoid leukemia | 1.23 | 0.94 | 1.52 |
| Female | Nicaragua | Chronic lymphoid leukemia | -2.19 | -3.00 | -1.37 |
| Female | Nicaragua | Acute myeloid leukemia | -0.84 | -1.07 | -0.61 |
| Female | Nicaragua | Chronic myeloid leukemia | 0.38 | -0.14 | 0.91 |
| Female | Nicaragua | Leukemia | -0.53 | -0.69 | -0.37 |
| Female | Nicaragua | Other leukemia | -1.95 | -2.07 | -1.83 |
| Both | Nicaragua | Acute lymphoid leukemia | 0.83 | 0.62 | 1.04 |
| Both | Nicaragua | Chronic lymphoid leukemia | -1.14 | -1.58 | -0.70 |
| Both | Nicaragua | Acute myeloid leukemia | -0.59 | -0.76 | -0.42 |
| Both | Nicaragua | Chronic myeloid leukemia | -0.20 | -0.45 | 0.05 |
| Both | Nicaragua | Leukemia | -0.62 | -0.74 | -0.50 |
| Both | Nicaragua | Other leukemia | -2.14 | -2.29 | -1.99 |
| Male | Niger | Acute lymphoid leukemia | -2.89 | -3.20 | -2.58 |
| Male | Niger | Chronic lymphoid leukemia | 0.48 | 0.33 | 0.62 |
| Male | Niger | Acute myeloid leukemia | -0.65 | -0.84 | -0.45 |
| Male | Niger | Chronic myeloid leukemia | 0.15 | 0.07 | 0.22 |
| Male | Niger | Leukemia | -0.63 | -0.78 | -0.49 |
| Male | Niger | Other leukemia | -0.23 | -0.32 | -0.15 |
| Female | Niger | Acute lymphoid leukemia | -0.49 | -0.61 | -0.38 |
| Female | Niger | Chronic lymphoid leukemia | 0.28 | 0.23 | 0.34 |
| Female | Niger | Acute myeloid leukemia | -0.20 | -0.28 | -0.11 |
| Female | Niger | Chronic myeloid leukemia | -0.82 | -0.91 | -0.72 |
| Female | Niger | Leukemia | -0.60 | -0.68 | -0.52 |
| Female | Niger | Other leukemia | -1.03 | -1.11 | -0.95 |
| Both | Niger | Acute lymphoid leukemia | -2.13 | -2.35 | -1.90 |
| Both | Niger | Chronic lymphoid leukemia | 0.48 | 0.40 | 0.55 |
| Both | Niger | Acute myeloid leukemia | -0.49 | -0.62 | -0.35 |
| Both | Niger | Chronic myeloid leukemia | -0.31 | -0.39 | -0.23 |
| Both | Niger | Leukemia | -0.62 | -0.72 | -0.52 |
| Both | Niger | Other leukemia | -0.62 | -0.70 | -0.54 |
| Male | Nigeria | Acute lymphoid leukemia | -1.89 | -2.24 | -1.55 |
| Male | Nigeria | Chronic lymphoid leukemia | 1.41 | 1.23 | 1.59 |
| Male | Nigeria | Acute myeloid leukemia | 0.62 | 0.36 | 0.88 |
| Male | Nigeria | Chronic myeloid leukemia | 0.18 | 0.07 | 0.29 |
| Male | Nigeria | Leukemia | -0.07 | -0.27 | 0.13 |
| Male | Nigeria | Other leukemia | -0.28 | -0.42 | -0.14 |
| Female | Nigeria | Acute lymphoid leukemia | 1.18 | 1.03 | 1.33 |
| Female | Nigeria | Chronic lymphoid leukemia | 0.26 | 0.19 | 0.33 |
| Female | Nigeria | Acute myeloid leukemia | 1.15 | 1.02 | 1.28 |
| Female | Nigeria | Chronic myeloid leukemia | -0.96 | -1.09 | -0.82 |
| Female | Nigeria | Leukemia | -0.04 | -0.06 | -0.02 |
| Female | Nigeria | Other leukemia | -0.97 | -1.05 | -0.89 |
| Both | Nigeria | Acute lymphoid leukemia | -0.75 | -0.91 | -0.59 |
| Both | Nigeria | Chronic lymphoid leukemia | 0.72 | 0.56 | 0.89 |
| Both | Nigeria | Acute myeloid leukemia | 0.78 | 0.65 | 0.90 |

| | | | | | |
|---|---|---|---|---|---|
| Both | Nigeria | Chronic myeloid leukemia | -0.01 | -0.03 | 0.01 |
| Both | Nigeria | Leukemia | -0.04 | -0.14 | 0.07 |
| Both | Nigeria | Other leukemia | -0.62 | -0.71 | -0.52 |
| Male | North Africa and Middle East | Acute lymphoid leukemia | -0.61 | -0.71 | -0.52 |
| Male | North Africa and Middle East | Chronic lymphoid leukemia | 2.51 | 2.25 | 2.76 |
| Male | North Africa and Middle East | Acute myeloid leukemia | 0.08 | -0.02 | 0.17 |
| Male | North Africa and Middle East | Chronic myeloid leukemia | -1.04 | -1.10 | -0.97 |
| Male | North Africa and Middle East | Leukemia | 0.05 | -0.02 | 0.11 |
| Male | North Africa and Middle East | Other leukemia | 0.01 | -0.05 | 0.07 |
| Female | North Africa and Middle East | Acute lymphoid leukemia | -0.26 | -0.43 | -0.09 |
| Female | North Africa and Middle East | Chronic lymphoid leukemia | 1.71 | 1.56 | 1.86 |
| Female | North Africa and Middle East | Acute myeloid leukemia | -0.75 | -0.87 | -0.62 |
| Female | North Africa and Middle East | Chronic myeloid leukemia | -2.27 | -2.38 | -2.17 |
| Female | North Africa and Middle East | Leukemia | -0.60 | -0.67 | -0.52 |
| Female | North Africa and Middle East | Other leukemia | -0.63 | -0.68 | -0.58 |
| Both | North Africa and Middle East | Acute lymphoid leukemia | -0.48 | -0.60 | -0.36 |
| Both | North Africa and Middle East | Chronic lymphoid leukemia | 2.18 | 1.99 | 2.37 |
| Both | North Africa and Middle East | Acute myeloid leukemia | -0.32 | -0.43 | -0.22 |
| Both | North Africa and Middle East | Chronic myeloid leukemia | -1.59 | -1.67 | -1.52 |
| Both | North Africa and Middle East | Other leukemia | -0.27 | -0.32 | -0.21 |
| Both | North Africa and Middle East | Leukemia | -0.24 | -0.31 | -0.17 |
| Male | North Korea | Acute lymphoid leukemia | 0.99 | 0.83 | 1.15 |
| Male | North Korea | Chronic lymphoid leukemia | 1.14 | 1.05 | 1.22 |
| Male | North Korea | Acute myeloid leukemia | -0.09 | -0.15 | -0.03 |
| Male | North Korea | Chronic myeloid leukemia | -0.37 | -0.50 | -0.24 |
| Male | North Korea | Leukemia | 0.31 | 0.18 | 0.44 |
| Male | North Korea | Other leukemia | 0.24 | 0.06 | 0.41 |
| Female | North Korea | Acute lymphoid leukemia | 0.45 | 0.37 | 0.53 |
| Female | North Korea | Chronic lymphoid leukemia | 1.53 | 1.34 | 1.72 |
| Female | North Korea | Acute myeloid leukemia | 0.18 | 0.13 | 0.22 |
| Female | North Korea | Chronic myeloid leukemia | -0.69 | -0.89 | -0.49 |
| Female | North Korea | Leukemia | 0.23 | 0.12 | 0.34 |
| Female | North Korea | Other leukemia | 0.16 | 0.01 | 0.32 |
| Both | North Korea | Acute lymphoid leukemia | 0.79 | 0.67 | 0.90 |
| Both | North Korea | Chronic lymphoid leukemia | 1.37 | 1.22 | 1.53 |
| Both | North Korea | Acute myeloid leukemia | 0.03 | -0.01 | 0.06 |
| Both | North Korea | Chronic myeloid leukemia | -0.49 | -0.64 | -0.34 |
| Both | North Korea | Leukemia | 0.29 | 0.18 | 0.40 |
| Both | North Korea | Other leukemia | 0.22 | 0.07 | 0.38 |
| Male | Northern Mariana Islands | Acute lymphoid leukemia | -1.03 | -1.18 | -0.88 |
| Male | Northern Mariana Islands | Chronic lymphoid leukemia | 0.88 | 0.43 | 1.32 |
| Male | Northern Mariana Islands | Acute myeloid leukemia | 1.68 | 1.44 | 1.92 |
| Male | Northern Mariana Islands | Chronic myeloid leukemia | -1.30 | -1.80 | -0.79 |
| Male | Northern Mariana Islands | Leukemia | 1.09 | 0.89 | 1.30 |
| Male | Northern Mariana Islands | Other leukemia | 0.84 | 0.49 | 1.19 |
| Female | Northern Mariana Islands | Acute lymphoid leukemia | -2.54 | -2.95 | -2.14 |
| Female | Northern Mariana Islands | Chronic lymphoid leukemia | 0.45 | 0.11 | 0.80 |
| Female | Northern Mariana Islands | Acute myeloid leukemia | -2.15 | -2.54 | -1.76 |

| | | | | | |
|---|---|---|---|---|---|
| Female | Northern Mariana Islands | Chronic myeloid leukemia | -3.51 | -3.93 | -3.09 |
| Female | Northern Mariana Islands | Leukemia | -1.12 | -1.37 | -0.87 |
| Female | Northern Mariana Islands | Other leukemia | -0.25 | -0.53 | 0.02 |
| Both | Northern Mariana Islands | Acute lymphoid leukemia | -1.97 | -2.22 | -1.72 |
| Both | Northern Mariana Islands | Chronic lymphoid leukemia | 0.76 | 0.40 | 1.12 |
| Both | Northern Mariana Islands | Acute myeloid leukemia | 0.89 | 0.66 | 1.13 |
| Both | Northern Mariana Islands | Chronic myeloid leukemia | -2.70 | -3.16 | -2.25 |
| Both | Northern Mariana Islands | Leukemia | 0.22 | 0.03 | 0.41 |
| Both | Northern Mariana Islands | Other leukemia | 0.42 | 0.12 | 0.73 |
| Male | Norway | Acute lymphoid leukemia | -1.54 | -1.80 | -1.28 |
| Male | Norway | Chronic lymphoid leukemia | 2.02 | 1.63 | 2.41 |
| Male | Norway | Acute myeloid leukemia | -1.34 | -1.51 | -1.16 |
| Male | Norway | Chronic myeloid leukemia | -1.82 | -2.14 | -1.50 |
| Male | Norway | Leukemia | 0.22 | 0.01 | 0.44 |
| Male | Norway | Other leukemia | 0.57 | 0.38 | 0.76 |
| Female | Norway | Acute lymphoid leukemia | -1.19 | -1.46 | -0.93 |
| Female | Norway | Chronic lymphoid leukemia | 1.73 | 1.33 | 2.14 |
| Female | Norway | Acute myeloid leukemia | -0.15 | -0.51 | 0.22 |
| Female | Norway | Chronic myeloid leukemia | -3.21 | -3.61 | -2.82 |
| Female | Norway | Leukemia | 0.02 | -0.26 | 0.31 |
| Female | Norway | Other leukemia | -0.49 | -0.66 | -0.32 |
| Both | Norway | Acute lymphoid leukemia | -1.39 | -1.62 | -1.15 |
| Both | Norway | Chronic lymphoid leukemia | 1.97 | 1.58 | 2.36 |
| Both | Norway | Acute myeloid leukemia | -0.75 | -0.99 | -0.51 |
| Both | Norway | Chronic myeloid leukemia | -2.36 | -2.68 | -2.03 |
| Both | Norway | Leukemia | 0.17 | -0.06 | 0.41 |
| Both | Norway | Other leukemia | 0.23 | 0.07 | 0.40 |
| Male | Oceania | Acute lymphoid leukemia | -0.32 | -0.37 | -0.28 |
| Male | Oceania | Chronic lymphoid leukemia | -0.57 | -0.65 | -0.48 |
| Male | Oceania | Acute myeloid leukemia | 0.49 | 0.43 | 0.56 |
| Male | Oceania | Chronic myeloid leukemia | -1.01 | -1.06 | -0.96 |
| Male | Oceania | Leukemia | 0.10 | 0.06 | 0.15 |
| Male | Oceania | Other leukemia | 0.13 | 0.06 | 0.19 |
| Female | Oceania | Acute lymphoid leukemia | -0.02 | -0.08 | 0.04 |
| Female | Oceania | Chronic lymphoid leukemia | 0.49 | 0.44 | 0.54 |
| Female | Oceania | Acute myeloid leukemia | -0.19 | -0.28 | -0.11 |
| Female | Oceania | Chronic myeloid leukemia | -1.10 | -1.13 | -1.08 |
| Female | Oceania | Leukemia | -0.27 | -0.31 | -0.23 |
| Female | Oceania | Other leukemia | -0.31 | -0.38 | -0.25 |
| Both | Oceania | Acute lymphoid leukemia | -0.21 | -0.25 | -0.16 |
| Both | Oceania | Chronic lymphoid leukemia | 0.37 | 0.32 | 0.42 |
| Both | Oceania | Acute myeloid leukemia | 0.23 | 0.16 | 0.30 |
| Both | Oceania | Chronic myeloid leukemia | -1.06 | -1.09 | -1.03 |
| Both | Oceania | Other leukemia | -0.06 | -0.12 | 0.00 |
| Both | Oceania | Leukemia | -0.07 | -0.11 | -0.03 |
| Male | Oman | Acute lymphoid leukemia | 0.09 | -0.22 | 0.41 |
| Male | Oman | Chronic lymphoid leukemia | 1.85 | 1.69 | 2.01 |
| Male | Oman | Acute myeloid leukemia | 0.28 | 0.06 | 0.51 |

| | | | | | |
|---|---|---|---|---|---|
| Male | Oman | Chronic myeloid leukemia | -0.30 | -0.47 | -0.14 |
| Male | Oman | Leukemia | 0.15 | -0.07 | 0.37 |
| Male | Oman | Other leukemia | 0.05 | -0.18 | 0.28 |
| Female | Oman | Acute lymphoid leukemia | -0.32 | -0.54 | -0.10 |
| Female | Oman | Chronic lymphoid leukemia | 2.20 | 2.06 | 2.34 |
| Female | Oman | Acute myeloid leukemia | -0.10 | -0.24 | 0.04 |
| Female | Oman | Chronic myeloid leukemia | -1.62 | -1.71 | -1.52 |
| Female | Oman | Leukemia | -0.39 | -0.59 | -0.20 |
| Female | Oman | Other leukemia | -0.58 | -0.81 | -0.35 |
| Both | Oman | Acute lymphoid leukemia | -0.03 | -0.31 | 0.25 |
| Both | Oman | Chronic lymphoid leukemia | 2.00 | 1.87 | 2.13 |
| Both | Oman | Acute myeloid leukemia | 0.14 | -0.03 | 0.30 |
| Both | Oman | Chronic myeloid leukemia | -0.78 | -0.91 | -0.64 |
| Both | Oman | Leukemia | -0.03 | -0.22 | 0.17 |
| Both | Oman | Other leukemia | -0.14 | -0.36 | 0.07 |
| Male | Pakistan | Acute lymphoid leukemia | 0.68 | 0.48 | 0.89 |
| Male | Pakistan | Chronic lymphoid leukemia | 1.33 | 1.21 | 1.45 |
| Male | Pakistan | Acute myeloid leukemia | 1.15 | 1.03 | 1.27 |
| Male | Pakistan | Chronic myeloid leukemia | -0.11 | -0.28 | 0.05 |
| Male | Pakistan | Leukemia | 0.55 | 0.42 | 0.69 |
| Male | Pakistan | Other leukemia | -0.10 | -0.28 | 0.09 |
| Female | Pakistan | Acute lymphoid leukemia | 2.19 | 1.90 | 2.48 |
| Female | Pakistan | Chronic lymphoid leukemia | 1.14 | 1.05 | 1.23 |
| Female | Pakistan | Acute myeloid leukemia | 1.70 | 1.48 | 1.92 |
| Female | Pakistan | Chronic myeloid leukemia | -0.25 | -0.54 | 0.03 |
| Female | Pakistan | Leukemia | 0.80 | 0.63 | 0.97 |
| Female | Pakistan | Other leukemia | -0.43 | -0.59 | -0.26 |
| Both | Pakistan | Acute lymphoid leukemia | 1.42 | 1.26 | 1.59 |
| Both | Pakistan | Chronic lymphoid leukemia | 1.20 | 1.11 | 1.28 |
| Both | Pakistan | Acute myeloid leukemia | 1.46 | 1.31 | 1.62 |
| Both | Pakistan | Chronic myeloid leukemia | -0.14 | -0.38 | 0.10 |
| Both | Pakistan | Leukemia | 0.71 | 0.57 | 0.85 |
| Both | Pakistan | Other leukemia | -0.26 | -0.43 | -0.10 |
| Male | Palestine | Acute lymphoid leukemia | 0.78 | 0.54 | 1.02 |
| Male | Palestine | Chronic lymphoid leukemia | 2.74 | 2.61 | 2.87 |
| Male | Palestine | Acute myeloid leukemia | 1.37 | 1.25 | 1.48 |
| Male | Palestine | Chronic myeloid leukemia | -0.09 | -0.35 | 0.17 |
| Male | Palestine | Leukemia | -0.50 | -0.56 | -0.45 |
| Male | Palestine | Other leukemia | -0.78 | -0.84 | -0.73 |
| Female | Palestine | Acute lymphoid leukemia | 0.27 | 0.11 | 0.42 |
| Female | Palestine | Chronic lymphoid leukemia | 1.41 | 1.25 | 1.58 |
| Female | Palestine | Acute myeloid leukemia | -0.54 | -0.63 | -0.45 |
| Female | Palestine | Chronic myeloid leukemia | -1.08 | -1.12 | -1.03 |
| Female | Palestine | Leukemia | -0.65 | -0.70 | -0.59 |
| Female | Palestine | Other leukemia | -0.88 | -0.96 | -0.80 |
| Both | Palestine | Acute lymphoid leukemia | 0.66 | 0.50 | 0.83 |
| Both | Palestine | Chronic lymphoid leukemia | 1.57 | 1.39 | 1.76 |
| Both | Palestine | Acute myeloid leukemia | 0.30 | 0.26 | 0.35 |

| | | | | | |
|---|---|---|---|---|---|
| Both | Palestine | Chronic myeloid leukemia | -0.53 | -0.68 | -0.38 |
| Both | Palestine | Leukemia | -0.55 | -0.59 | -0.52 |
| Both | Palestine | Other leukemia | -0.82 | -0.85 | -0.78 |
| Male | Panama | Acute lymphoid leukemia | -0.34 | -0.83 | 0.14 |
| Male | Panama | Chronic lymphoid leukemia | 1.05 | 0.74 | 1.36 |
| Male | Panama | Acute myeloid leukemia | -0.38 | -0.59 | -0.16 |
| Male | Panama | Chronic myeloid leukemia | -1.31 | -1.64 | -0.97 |
| Male | Panama | Leukemia | -0.58 | -0.91 | -0.26 |
| Male | Panama | Other leukemia | -1.24 | -1.66 | -0.82 |
| Female | Panama | Acute lymphoid leukemia | 0.11 | -0.17 | 0.40 |
| Female | Panama | Chronic lymphoid leukemia | -0.34 | -0.55 | -0.12 |
| Female | Panama | Acute myeloid leukemia | -0.68 | -1.15 | -0.21 |
| Female | Panama | Chronic myeloid leukemia | -1.81 | -2.15 | -1.47 |
| Female | Panama | Leukemia | -0.46 | -0.76 | -0.16 |
| Female | Panama | Other leukemia | -0.48 | -0.80 | -0.15 |
| Both | Panama | Acute lymphoid leukemia | -0.13 | -0.51 | 0.24 |
| Both | Panama | Chronic lymphoid leukemia | 0.43 | 0.29 | 0.56 |
| Both | Panama | Acute myeloid leukemia | -0.53 | -0.80 | -0.26 |
| Both | Panama | Chronic myeloid leukemia | -1.52 | -1.83 | -1.20 |
| Both | Panama | Leukemia | -0.54 | -0.84 | -0.23 |
| Both | Panama | Other leukemia | -0.91 | -1.27 | -0.54 |
| Male | Papua New Guinea | Acute lymphoid leukemia | -0.11 | -0.20 | -0.02 |
| Male | Papua New Guinea | Chronic lymphoid leukemia | -0.48 | -0.56 | -0.39 |
| Male | Papua New Guinea | Acute myeloid leukemia | 0.49 | 0.46 | 0.52 |
| Male | Papua New Guinea | Chronic myeloid leukemia | -0.96 | -1.01 | -0.91 |
| Male | Papua New Guinea | Leukemia | 0.07 | 0.02 | 0.13 |
| Male | Papua New Guinea | Other leukemia | 0.08 | 0.00 | 0.16 |
| Female | Papua New Guinea | Acute lymphoid leukemia | 0.28 | 0.18 | 0.37 |
| Female | Papua New Guinea | Chronic lymphoid leukemia | 0.65 | 0.58 | 0.72 |
| Female | Papua New Guinea | Acute myeloid leukemia | -0.20 | -0.26 | -0.14 |
| Female | Papua New Guinea | Chronic myeloid leukemia | -1.18 | -1.22 | -1.14 |
| Female | Papua New Guinea | Leukemia | -0.27 | -0.34 | -0.20 |
| Female | Papua New Guinea | Other leukemia | -0.32 | -0.42 | -0.22 |
| Both | Papua New Guinea | Acute lymphoid leukemia | 0.04 | -0.05 | 0.13 |
| Both | Papua New Guinea | Chronic lymphoid leukemia | 0.44 | 0.39 | 0.49 |
| Both | Papua New Guinea | Acute myeloid leukemia | 0.21 | 0.18 | 0.24 |
| Both | Papua New Guinea | Chronic myeloid leukemia | -1.10 | -1.14 | -1.07 |
| Both | Papua New Guinea | Leukemia | -0.09 | -0.15 | -0.03 |
| Both | Papua New Guinea | Other leukemia | -0.08 | -0.17 | 0.01 |
| Male | Paraguay | Acute lymphoid leukemia | 1.79 | 1.28 | 2.31 |
| Male | Paraguay | Chronic lymphoid leukemia | 2.25 | 2.02 | 2.48 |
| Male | Paraguay | Acute myeloid leukemia | 2.07 | 1.87 | 2.27 |
| Male | Paraguay | Chronic myeloid leukemia | 1.53 | 1.12 | 1.95 |
| Male | Paraguay | Leukemia | -0.04 | -0.23 | 0.14 |
| Male | Paraguay | Other leukemia | -2.43 | -2.77 | -2.10 |
| Female | Paraguay | Acute lymphoid leukemia | 1.03 | 0.63 | 1.43 |
| Female | Paraguay | Chronic lymphoid leukemia | 3.73 | 3.12 | 4.35 |
| Female | Paraguay | Acute myeloid leukemia | 2.85 | 2.34 | 3.36 |

| | | | | | |
|---|---|---|---|---|---|
| Female | Paraguay | Chronic myeloid leukemia | 0.92 | 0.37 | 1.48 |
| Female | Paraguay | Leukemia | -0.28 | -0.55 | -0.02 |
| Female | Paraguay | Other leukemia | -3.00 | -3.38 | -2.63 |
| Both | Paraguay | Acute lymphoid leukemia | 1.44 | 0.99 | 1.90 |
| Both | Paraguay | Chronic lymphoid leukemia | 2.76 | 2.41 | 3.11 |
| Both | Paraguay | Acute myeloid leukemia | 2.42 | 2.13 | 2.72 |
| Both | Paraguay | Chronic myeloid leukemia | 1.31 | 0.86 | 1.76 |
| Both | Paraguay | Leukemia | -0.15 | -0.37 | 0.07 |
| Both | Paraguay | Other leukemia | -2.70 | -3.06 | -2.35 |
| Male | Peru | Acute lymphoid leukemia | 0.28 | 0.05 | 0.51 |
| Male | Peru | Chronic lymphoid leukemia | 1.56 | 1.31 | 1.82 |
| Male | Peru | Acute myeloid leukemia | 0.94 | 0.70 | 1.19 |
| Male | Peru | Chronic myeloid leukemia | 0.17 | -0.04 | 0.39 |
| Male | Peru | Leukemia | -0.25 | -0.39 | -0.11 |
| Male | Peru | Other leukemia | -1.17 | -1.52 | -0.83 |
| Female | Peru | Acute lymphoid leukemia | 1.13 | 0.86 | 1.41 |
| Female | Peru | Chronic lymphoid leukemia | 3.04 | 2.49 | 3.60 |
| Female | Peru | Acute myeloid leukemia | 1.00 | 0.72 | 1.27 |
| Female | Peru | Chronic myeloid leukemia | -0.63 | -0.87 | -0.40 |
| Female | Peru | Leukemia | -0.19 | -0.33 | -0.04 |
| Female | Peru | Other leukemia | -1.33 | -1.69 | -0.97 |
| Both | Peru | Acute lymphoid leukemia | 0.65 | 0.40 | 0.90 |
| Both | Peru | Chronic lymphoid leukemia | 2.17 | 1.80 | 2.53 |
| Both | Peru | Acute myeloid leukemia | 0.97 | 0.72 | 1.21 |
| Both | Peru | Chronic myeloid leukemia | -0.14 | -0.35 | 0.07 |
| Both | Peru | Leukemia | -0.22 | -0.35 | -0.08 |
| Both | Peru | Other leukemia | -1.24 | -1.59 | -0.89 |
| Male | Philippines | Acute lymphoid leukemia | 1.62 | 1.29 | 1.96 |
| Male | Philippines | Chronic lymphoid leukemia | 2.35 | 2.08 | 2.62 |
| Male | Philippines | Acute myeloid leukemia | 2.34 | 2.14 | 2.55 |
| Male | Philippines | Chronic myeloid leukemia | 2.04 | 1.84 | 2.25 |
| Male | Philippines | Leukemia | 0.95 | 0.73 | 1.17 |
| Male | Philippines | Other leukemia | -0.09 | -0.36 | 0.17 |
| Female | Philippines | Acute lymphoid leukemia | 1.81 | 1.42 | 2.20 |
| Female | Philippines | Chronic lymphoid leukemia | 0.94 | 0.59 | 1.30 |
| Female | Philippines | Acute myeloid leukemia | 2.81 | 2.59 | 3.03 |
| Female | Philippines | Chronic myeloid leukemia | 2.23 | 1.88 | 2.57 |
| Female | Philippines | Leukemia | 1.08 | 0.81 | 1.35 |
| Female | Philippines | Other leukemia | -0.02 | -0.34 | 0.31 |
| Both | Philippines | Acute lymphoid leukemia | 1.69 | 1.34 | 2.05 |
| Both | Philippines | Chronic lymphoid leukemia | 1.64 | 1.36 | 1.92 |
| Both | Philippines | Acute myeloid leukemia | 2.57 | 2.37 | 2.77 |
| Both | Philippines | Chronic myeloid leukemia | 2.10 | 1.85 | 2.36 |
| Both | Philippines | Leukemia | 1.00 | 0.77 | 1.23 |
| Both | Philippines | Other leukemia | -0.07 | -0.34 | 0.21 |
| Male | Poland | Acute lymphoid leukemia | -1.30 | -1.38 | -1.22 |
| Male | Poland | Chronic lymphoid leukemia | 2.86 | 2.67 | 3.05 |
| Male | Poland | Acute myeloid leukemia | 0.74 | 0.52 | 0.96 |

| | | | | | |
|---|---|---|---|---|---|
| Male | Poland | Chronic myeloid leukemia | -3.48 | -3.84 | -3.12 |
| Male | Poland | Leukemia | -0.01 | -0.13 | 0.10 |
| Male | Poland | Other leukemia | -1.97 | -2.44 | -1.50 |
| Female | Poland | Acute lymphoid leukemia | -1.57 | -1.68 | -1.46 |
| Female | Poland | Chronic lymphoid leukemia | 2.49 | 2.27 | 2.71 |
| Female | Poland | Acute myeloid leukemia | 0.64 | 0.36 | 0.93 |
| Female | Poland | Chronic myeloid leukemia | -4.36 | -4.88 | -3.85 |
| Female | Poland | Leukemia | -0.41 | -0.59 | -0.23 |
| Female | Poland | Other leukemia | -2.66 | -3.16 | -2.16 |
| Both | Poland | Acute lymphoid leukemia | -1.38 | -1.46 | -1.29 |
| Both | Poland | Chronic lymphoid leukemia | 2.75 | 2.56 | 2.94 |
| Both | Poland | Acute myeloid leukemia | 0.68 | 0.44 | 0.92 |
| Both | Poland | Chronic myeloid leukemia | -3.85 | -4.25 | -3.45 |
| Both | Poland | Leukemia | -0.17 | -0.31 | -0.04 |
| Both | Poland | Other leukemia | -2.25 | -2.73 | -1.77 |
| Male | Portugal | Acute lymphoid leukemia | -2.55 | -2.87 | -2.22 |
| Male | Portugal | Chronic lymphoid leukemia | 1.95 | 1.65 | 2.26 |
| Male | Portugal | Acute myeloid leukemia | 0.16 | -0.12 | 0.45 |
| Male | Portugal | Chronic myeloid leukemia | -3.56 | -4.07 | -3.04 |
| Male | Portugal | Leukemia | -0.85 | -1.02 | -0.67 |
| Male | Portugal | Other leukemia | -2.67 | -3.05 | -2.29 |
| Female | Portugal | Acute lymphoid leukemia | -4.08 | -4.62 | -3.54 |
| Female | Portugal | Chronic lymphoid leukemia | 1.00 | 0.66 | 1.35 |
| Female | Portugal | Acute myeloid leukemia | -0.56 | -1.06 | -0.06 |
| Female | Portugal | Chronic myeloid leukemia | -4.48 | -4.94 | -4.01 |
| Female | Portugal | Leukemia | -1.75 | -1.98 | -1.52 |
| Female | Portugal | Other leukemia | -3.36 | -3.74 | -2.98 |
| Both | Portugal | Acute lymphoid leukemia | -3.16 | -3.53 | -2.79 |
| Both | Portugal | Chronic lymphoid leukemia | 1.66 | 1.35 | 1.99 |
| Both | Portugal | Acute myeloid leukemia | -0.17 | -0.55 | 0.21 |
| Both | Portugal | Chronic myeloid leukemia | -3.85 | -4.34 | -3.37 |
| Both | Portugal | Leukemia | -1.21 | -1.41 | -1.02 |
| Both | Portugal | Other leukemia | -2.96 | -3.34 | -2.59 |
| Male | Puerto Rico | Acute lymphoid leukemia | -1.42 | -1.67 | -1.17 |
| Male | Puerto Rico | Chronic lymphoid leukemia | -0.05 | -0.22 | 0.11 |
| Male | Puerto Rico | Acute myeloid leukemia | -0.15 | -0.41 | 0.11 |
| Male | Puerto Rico | Chronic myeloid leukemia | -4.56 | -4.98 | -4.13 |
| Male | Puerto Rico | Leukemia | -0.42 | -0.54 | -0.30 |
| Male | Puerto Rico | Other leukemia | 0.44 | 0.11 | 0.77 |
| Female | Puerto Rico | Acute lymphoid leukemia | -1.91 | -2.27 | -1.56 |
| Female | Puerto Rico | Chronic lymphoid leukemia | -0.91 | -1.25 | -0.57 |
| Female | Puerto Rico | Acute myeloid leukemia | -1.04 | -1.28 | -0.80 |
| Female | Puerto Rico | Chronic myeloid leukemia | -4.12 | -4.54 | -3.71 |
| Female | Puerto Rico | Leukemia | -0.96 | -1.09 | -0.84 |
| Female | Puerto Rico | Other leukemia | 0.05 | -0.27 | 0.38 |
| Both | Puerto Rico | Acute lymphoid leukemia | -1.63 | -1.90 | -1.36 |
| Both | Puerto Rico | Chronic lymphoid leukemia | -0.45 | -0.66 | -0.25 |
| Both | Puerto Rico | Acute myeloid leukemia | -0.60 | -0.83 | -0.36 |

| | | | | | |
|---|---|---|---|---|---|
| Both | Puerto Rico | Chronic myeloid leukemia | -4.37 | -4.75 | -3.99 |
| Both | Puerto Rico | Leukemia | -0.69 | -0.80 | -0.59 |
| Both | Puerto Rico | Other leukemia | 0.23 | -0.08 | 0.54 |
| Male | Qatar | Acute lymphoid leukemia | -3.53 | -4.05 | -3.01 |
| Male | Qatar | Chronic lymphoid leukemia | -1.77 | -2.51 | -1.02 |
| Male | Qatar | Acute myeloid leukemia | -2.93 | -3.61 | -2.24 |
| Male | Qatar | Chronic myeloid leukemia | -2.87 | -3.49 | -2.25 |
| Male | Qatar | Leukemia | -1.87 | -2.28 | -1.47 |
| Male | Qatar | Other leukemia | -0.70 | -1.06 | -0.33 |
| Female | Qatar | Acute lymphoid leukemia | -1.68 | -1.95 | -1.41 |
| Female | Qatar | Chronic lymphoid leukemia | 1.06 | 0.84 | 1.27 |
| Female | Qatar | Acute myeloid leukemia | -1.54 | -1.77 | -1.31 |
| Female | Qatar | Chronic myeloid leukemia | -3.18 | -3.44 | -2.91 |
| Female | Qatar | Leukemia | -1.46 | -1.60 | -1.33 |
| Female | Qatar | Other leukemia | -1.80 | -2.02 | -1.58 |
| Both | Qatar | Acute lymphoid leukemia | -2.86 | -3.29 | -2.42 |
| Both | Qatar | Chronic lymphoid leukemia | -0.53 | -1.06 | 0.01 |
| Both | Qatar | Acute myeloid leukemia | -2.25 | -2.77 | -1.73 |
| Both | Qatar | Chronic myeloid leukemia | -2.53 | -3.00 | -2.06 |
| Both | Qatar | Leukemia | -1.38 | -1.66 | -1.10 |
| Both | Qatar | Other leukemia | -0.56 | -0.79 | -0.34 |
| Male | Republic of Congo | Acute lymphoid leukemia | -0.29 | -0.55 | -0.04 |
| Male | Republic of Congo | Chronic lymphoid leukemia | 3.19 | 3.02 | 3.36 |
| Male | Republic of Congo | Acute myeloid leukemia | 0.04 | -0.12 | 0.20 |
| Male | Republic of Congo | Chronic myeloid leukemia | 0.43 | 0.31 | 0.56 |
| Male | Republic of Congo | Leukemia | -0.26 | -0.42 | -0.11 |
| Male | Republic of Congo | Other leukemia | -1.38 | -1.60 | -1.16 |
| Female | Republic of Congo | Acute lymphoid leukemia | 0.64 | 0.46 | 0.81 |
| Female | Republic of Congo | Chronic lymphoid leukemia | 1.38 | 1.29 | 1.48 |
| Female | Republic of Congo | Acute myeloid leukemia | 1.27 | 1.14 | 1.39 |
| Female | Republic of Congo | Chronic myeloid leukemia | -0.12 | -0.27 | 0.03 |
| Female | Republic of Congo | Leukemia | -0.39 | -0.57 | -0.21 |
| Female | Republic of Congo | Other leukemia | -1.41 | -1.68 | -1.14 |
| Both | Republic of Congo | Acute lymphoid leukemia | 0.11 | -0.10 | 0.31 |
| Both | Republic of Congo | Chronic lymphoid leukemia | 2.05 | 1.95 | 2.16 |
| Both | Republic of Congo | Acute myeloid leukemia | 0.43 | 0.28 | 0.57 |
| Both | Republic of Congo | Chronic myeloid leukemia | 0.00 | -0.11 | 0.10 |
| Both | Republic of Congo | Leukemia | -0.32 | -0.49 | -0.16 |
| Both | Republic of Congo | Other leukemia | -1.44 | -1.69 | -1.19 |
| Male | Romania | Acute lymphoid leukemia | -1.35 | -1.61 | -1.10 |
| Male | Romania | Chronic lymphoid leukemia | 2.98 | 2.74 | 3.22 |
| Male | Romania | Acute myeloid leukemia | 0.53 | 0.42 | 0.64 |
| Male | Romania | Chronic myeloid leukemia | -1.10 | -1.53 | -0.66 |
| Male | Romania | Leukemia | 0.39 | 0.30 | 0.49 |
| Male | Romania | Other leukemia | 0.08 | 0.01 | 0.15 |
| Female | Romania | Acute lymphoid leukemia | -1.40 | -1.70 | -1.09 |
| Female | Romania | Chronic lymphoid leukemia | 2.88 | 2.64 | 3.12 |
| Female | Romania | Acute myeloid leukemia | 0.34 | 0.19 | 0.48 |

| | | | | | |
|---|---|---|---|---|---|
| Female | Romania | Chronic myeloid leukemia | -2.66 | -3.03 | -2.30 |
| Female | Romania | Leukemia | 0.13 | -0.03 | 0.30 |
| Female | Romania | Other leukemia | -0.13 | -0.38 | 0.12 |
| Both | Romania | Acute lymphoid leukemia | -1.41 | -1.68 | -1.15 |
| Both | Romania | Chronic lymphoid leukemia | 2.90 | 2.66 | 3.13 |
| Both | Romania | Acute myeloid leukemia | 0.39 | 0.29 | 0.50 |
| Both | Romania | Chronic myeloid leukemia | -1.70 | -2.09 | -1.32 |
| Both | Romania | Leukemia | 0.23 | 0.12 | 0.34 |
| Both | Romania | Other leukemia | -0.05 | -0.18 | 0.08 |
| Male | Russia | Acute lymphoid leukemia | -1.34 | -1.59 | -1.09 |
| Male | Russia | Chronic lymphoid leukemia | 1.97 | 1.74 | 2.21 |
| Male | Russia | Acute myeloid leukemia | 0.16 | -0.06 | 0.38 |
| Male | Russia | Chronic myeloid leukemia | -0.19 | -0.64 | 0.26 |
| Male | Russia | Leukemia | 0.33 | 0.12 | 0.53 |
| Male | Russia | Other leukemia | 0.15 | -0.09 | 0.39 |
| Female | Russia | Acute lymphoid leukemia | -1.43 | -1.62 | -1.24 |
| Female | Russia | Chronic lymphoid leukemia | 3.09 | 2.69 | 3.48 |
| Female | Russia | Acute myeloid leukemia | -1.53 | -1.77 | -1.29 |
| Female | Russia | Chronic myeloid leukemia | 0.02 | -0.43 | 0.47 |
| Female | Russia | Leukemia | 0.34 | 0.14 | 0.54 |
| Female | Russia | Other leukemia | 0.93 | 0.62 | 1.24 |
| Both | Russia | Acute lymphoid leukemia | -1.39 | -1.60 | -1.17 |
| Both | Russia | Chronic lymphoid leukemia | 2.64 | 2.34 | 2.95 |
| Both | Russia | Acute myeloid leukemia | -0.61 | -0.76 | -0.46 |
| Both | Russia | Chronic myeloid leukemia | -0.01 | -0.47 | 0.45 |
| Both | Russia | Leukemia | 0.38 | 0.18 | 0.59 |
| Both | Russia | Other leukemia | 0.53 | 0.26 | 0.81 |
| Male | Rwanda | Acute lymphoid leukemia | -1.33 | -1.69 | -0.98 |
| Male | Rwanda | Chronic lymphoid leukemia | 2.63 | 2.46 | 2.80 |
| Male | Rwanda | Acute myeloid leukemia | 0.94 | 0.83 | 1.04 |
| Male | Rwanda | Chronic myeloid leukemia | -1.27 | -1.45 | -1.08 |
| Male | Rwanda | Leukemia | -0.50 | -0.70 | -0.30 |
| Male | Rwanda | Other leukemia | -2.38 | -2.76 | -2.00 |
| Female | Rwanda | Acute lymphoid leukemia | 1.21 | 0.94 | 1.49 |
| Female | Rwanda | Chronic lymphoid leukemia | 0.27 | 0.19 | 0.35 |
| Female | Rwanda | Acute myeloid leukemia | 0.80 | 0.66 | 0.95 |
| Female | Rwanda | Chronic myeloid leukemia | -2.03 | -2.27 | -1.79 |
| Female | Rwanda | Leukemia | -0.92 | -1.14 | -0.70 |
| Female | Rwanda | Other leukemia | -2.25 | -2.63 | -1.88 |
| Both | Rwanda | Acute lymphoid leukemia | -0.30 | -0.60 | 0.01 |
| Both | Rwanda | Chronic lymphoid leukemia | 1.18 | 1.11 | 1.26 |
| Both | Rwanda | Acute myeloid leukemia | 0.71 | 0.60 | 0.82 |
| Both | Rwanda | Chronic myeloid leukemia | -1.74 | -1.96 | -1.53 |
| Both | Rwanda | Leukemia | -0.77 | -0.98 | -0.57 |
| Both | Rwanda | Other leukemia | -2.33 | -2.70 | -1.95 |
| Male | Saint Lucia | Acute lymphoid leukemia | -0.34 | -0.72 | 0.05 |
| Male | Saint Lucia | Chronic lymphoid leukemia | -0.44 | -0.62 | -0.26 |
| Male | Saint Lucia | Acute myeloid leukemia | -0.07 | -0.21 | 0.07 |

| | | | | | |
|---|---|---|---|---|---|
| Male | Saint Lucia | Chronic myeloid leukemia | -1.87 | -2.10 | -1.64 |
| Male | Saint Lucia | Leukemia | -1.08 | -1.24 | -0.93 |
| Male | Saint Lucia | Other leukemia | -1.89 | -2.08 | -1.70 |
| Female | Saint Lucia | Acute lymphoid leukemia | -0.51 | -0.60 | -0.41 |
| Female | Saint Lucia | Chronic lymphoid leukemia | 1.24 | 0.88 | 1.61 |
| Female | Saint Lucia | Acute myeloid leukemia | 0.32 | 0.12 | 0.51 |
| Female | Saint Lucia | Chronic myeloid leukemia | -2.76 | -3.04 | -2.47 |
| Female | Saint Lucia | Leukemia | -1.06 | -1.28 | -0.85 |
| Female | Saint Lucia | Other leukemia | -1.88 | -2.12 | -1.65 |
| Both | Saint Lucia | Acute lymphoid leukemia | -0.36 | -0.62 | -0.09 |
| Both | Saint Lucia | Chronic lymphoid leukemia | 0.28 | 0.08 | 0.48 |
| Both | Saint Lucia | Acute myeloid leukemia | 0.13 | 0.03 | 0.23 |
| Both | Saint Lucia | Chronic myeloid leukemia | -2.28 | -2.52 | -2.03 |
| Both | Saint Lucia | Leukemia | -1.02 | -1.14 | -0.89 |
| Both | Saint Lucia | Other leukemia | -1.84 | -1.99 | -1.69 |
| Male | Saint Vincent and the Grenadines | Acute lymphoid leukemia | -1.58 | -1.81 | -1.34 |
| Male | Saint Vincent and the Grenadines | Chronic lymphoid leukemia | 1.02 | 0.93 | 1.11 |
| Male | Saint Vincent and the Grenadines | Acute myeloid leukemia | 1.62 | 1.35 | 1.88 |
| Male | Saint Vincent and the Grenadines | Chronic myeloid leukemia | 2.97 | 2.28 | 3.67 |
| Male | Saint Vincent and the Grenadines | Leukemia | -0.07 | -0.21 | 0.07 |
| Male | Saint Vincent and the Grenadines | Other leukemia | -1.12 | -1.29 | -0.94 |
| Female | Saint Vincent and the Grenadines | Acute lymphoid leukemia | 3.63 | 2.52 | 4.76 |
| Female | Saint Vincent and the Grenadines | Chronic lymphoid leukemia | 2.34 | 1.72 | 2.96 |
| Female | Saint Vincent and the Grenadines | Acute myeloid leukemia | 2.73 | 2.15 | 3.32 |
| Female | Saint Vincent and the Grenadines | Chronic myeloid leukemia | -6.26 | -7.36 | -5.15 |
| Female | Saint Vincent and the Grenadines | Leukemia | -0.42 | -0.64 | -0.21 |
| Female | Saint Vincent and the Grenadines | Other leukemia | -0.14 | -0.57 | 0.29 |
| Both | Saint Vincent and the Grenadines | Acute lymphoid leukemia | 1.40 | 0.82 | 1.99 |
| Both | Saint Vincent and the Grenadines | Chronic lymphoid leukemia | 1.67 | 1.43 | 1.91 |
| Both | Saint Vincent and the Grenadines | Acute myeloid leukemia | 2.21 | 1.81 | 2.61 |
| Both | Saint Vincent and the Grenadines | Chronic myeloid leukemia | -3.50 | -4.21 | -2.77 |
| Both | Saint Vincent and the Grenadines | Leukemia | -0.21 | -0.36 | -0.05 |
| Both | Saint Vincent and the Grenadines | Other leukemia | -0.62 | -0.88 | -0.36 |
| Male | Samoa | Acute lymphoid leukemia | -0.44 | -0.53 | -0.35 |
| Male | Samoa | Chronic lymphoid leukemia | -0.68 | -0.83 | -0.53 |
| Male | Samoa | Acute myeloid leukemia | 0.22 | 0.10 | 0.34 |
| Male | Samoa | Chronic myeloid leukemia | -1.48 | -1.60 | -1.36 |
| Male | Samoa | Leukemia | -0.49 | -0.53 | -0.45 |
| Male | Samoa | Other leukemia | -0.76 | -0.83 | -0.69 |
| Female | Samoa | Acute lymphoid leukemia | -0.26 | -0.35 | -0.17 |
| Female | Samoa | Chronic lymphoid leukemia | 1.21 | 1.14 | 1.27 |
| Female | Samoa | Acute myeloid leukemia | -0.49 | -0.55 | -0.43 |
| Female | Samoa | Chronic myeloid leukemia | -0.91 | -1.01 | -0.81 |
| Female | Samoa | Leukemia | -0.53 | -0.59 | -0.47 |
| Female | Samoa | Other leukemia | -0.89 | -0.96 | -0.81 |
| Both | Samoa | Acute lymphoid leukemia | -0.38 | -0.47 | -0.30 |
| Both | Samoa | Chronic lymphoid leukemia | 1.03 | 0.96 | 1.11 |
| Both | Samoa | Acute myeloid leukemia | -0.07 | -0.14 | 0.00 |

| | | | | | |
|---|---|---|---|---|---|
| Both | Samoa | Chronic myeloid leukemia | -1.19 | -1.30 | -1.08 |
| Both | Samoa | Leukemia | -0.54 | -0.59 | -0.49 |
| Both | Samoa | Other leukemia | -0.86 | -0.94 | -0.78 |
| Male | Sao Tome and Principe | Acute lymphoid leukemia | -1.37 | -1.61 | -1.12 |
| Male | Sao Tome and Principe | Chronic lymphoid leukemia | 2.11 | 2.01 | 2.21 |
| Male | Sao Tome and Principe | Acute myeloid leukemia | 0.55 | 0.45 | 0.66 |
| Male | Sao Tome and Principe | Chronic myeloid leukemia | 1.22 | 1.16 | 1.27 |
| Male | Sao Tome and Principe | Leukemia | 0.62 | 0.54 | 0.70 |
| Male | Sao Tome and Principe | Other leukemia | 1.13 | 1.06 | 1.19 |
| Female | Sao Tome and Principe | Acute lymphoid leukemia | 0.55 | 0.33 | 0.78 |
| Female | Sao Tome and Principe | Chronic lymphoid leukemia | 0.89 | 0.83 | 0.95 |
| Female | Sao Tome and Principe | Acute myeloid leukemia | 0.58 | 0.45 | 0.70 |
| Female | Sao Tome and Principe | Chronic myeloid leukemia | -0.28 | -0.50 | -0.05 |
| Female | Sao Tome and Principe | Leukemia | 0.00 | -0.10 | 0.11 |
| Female | Sao Tome and Principe | Other leukemia | -1.13 | -1.25 | -1.01 |
| Both | Sao Tome and Principe | Acute lymphoid leukemia | -0.50 | -0.64 | -0.36 |
| Both | Sao Tome and Principe | Chronic lymphoid leukemia | 1.30 | 1.25 | 1.35 |
| Both | Sao Tome and Principe | Acute myeloid leukemia | 0.61 | 0.52 | 0.70 |
| Both | Sao Tome and Principe | Chronic myeloid leukemia | 0.36 | 0.21 | 0.51 |
| Both | Sao Tome and Principe | Leukemia | 0.34 | 0.26 | 0.42 |
| Both | Sao Tome and Principe | Other leukemia | 0.18 | 0.11 | 0.25 |
| Male | Saudi Arabia | Acute lymphoid leukemia | 0.87 | 0.76 | 0.99 |
| Male | Saudi Arabia | Chronic lymphoid leukemia | 4.63 | 4.30 | 4.95 |
| Male | Saudi Arabia | Acute myeloid leukemia | 1.47 | 1.29 | 1.64 |
| Male | Saudi Arabia | Chronic myeloid leukemia | 0.60 | 0.36 | 0.84 |
| Male | Saudi Arabia | Leukemia | 1.44 | 1.29 | 1.60 |
| Male | Saudi Arabia | Other leukemia | 1.38 | 1.15 | 1.60 |
| Female | Saudi Arabia | Acute lymphoid leukemia | -0.04 | -0.20 | 0.12 |
| Female | Saudi Arabia | Chronic lymphoid leukemia | 3.39 | 2.82 | 3.96 |
| Female | Saudi Arabia | Acute myeloid leukemia | -0.01 | -0.11 | 0.09 |
| Female | Saudi Arabia | Chronic myeloid leukemia | 0.13 | -0.06 | 0.33 |
| Female | Saudi Arabia | Leukemia | -0.02 | -0.09 | 0.06 |
| Female | Saudi Arabia | Other leukemia | -0.25 | -0.36 | -0.14 |
| Both | Saudi Arabia | Acute lymphoid leukemia | 0.55 | 0.45 | 0.66 |
| Both | Saudi Arabia | Chronic lymphoid leukemia | 4.31 | 3.98 | 4.65 |
| Both | Saudi Arabia | Acute myeloid leukemia | 0.65 | 0.55 | 0.75 |
| Both | Saudi Arabia | Chronic myeloid leukemia | 0.46 | 0.32 | 0.61 |
| Both | Saudi Arabia | Leukemia | 0.93 | 0.82 | 1.04 |
| Both | Saudi Arabia | Other leukemia | 0.84 | 0.67 | 1.02 |
| Male | Senegal | Acute lymphoid leukemia | -1.55 | -1.68 | -1.41 |
| Male | Senegal | Chronic lymphoid leukemia | 2.00 | 1.90 | 2.10 |
| Male | Senegal | Acute myeloid leukemia | 0.75 | 0.61 | 0.88 |
| Male | Senegal | Chronic myeloid leukemia | 0.34 | 0.25 | 0.44 |
| Male | Senegal | Leukemia | 0.59 | 0.51 | 0.66 |
| Male | Senegal | Other leukemia | 1.10 | 0.99 | 1.21 |
| Female | Senegal | Acute lymphoid leukemia | 1.24 | 1.11 | 1.36 |
| Female | Senegal | Chronic lymphoid leukemia | 0.48 | 0.41 | 0.56 |
| Female | Senegal | Acute myeloid leukemia | 1.06 | 0.95 | 1.18 |

| | | | | | |
|---|---|---|---|---|---|
| Female | Senegal | Chronic myeloid leukemia | -0.13 | -0.18 | -0.09 |
| Female | Senegal | Leukemia | 0.33 | 0.25 | 0.41 |
| Female | Senegal | Other leukemia | -0.31 | -0.41 | -0.21 |
| Both | Senegal | Acute lymphoid leukemia | -0.63 | -0.72 | -0.53 |
| Both | Senegal | Chronic lymphoid leukemia | 1.10 | 1.02 | 1.18 |
| Both | Senegal | Acute myeloid leukemia | 0.87 | 0.75 | 1.00 |
| Both | Senegal | Chronic myeloid leukemia | 0.10 | 0.06 | 0.15 |
| Both | Senegal | Leukemia | 0.47 | 0.39 | 0.55 |
| Both | Senegal | Other leukemia | 0.50 | 0.40 | 0.60 |
| Male | Serbia | Acute lymphoid leukemia | -0.87 | -1.07 | -0.67 |
| Male | Serbia | Chronic lymphoid leukemia | 3.20 | 2.76 | 3.64 |
| Male | Serbia | Acute myeloid leukemia | 0.36 | 0.12 | 0.61 |
| Male | Serbia | Chronic myeloid leukemia | 1.11 | 0.63 | 1.59 |
| Male | Serbia | Leukemia | 0.19 | -0.03 | 0.41 |
| Male | Serbia | Other leukemia | -0.78 | -1.02 | -0.54 |
| Female | Serbia | Acute lymphoid leukemia | 0.30 | 0.06 | 0.53 |
| Female | Serbia | Chronic lymphoid leukemia | 2.28 | 1.96 | 2.61 |
| Female | Serbia | Acute myeloid leukemia | 0.36 | 0.10 | 0.62 |
| Female | Serbia | Chronic myeloid leukemia | 0.58 | 0.15 | 1.01 |
| Female | Serbia | Leukemia | -0.32 | -0.45 | -0.19 |
| Female | Serbia | Other leukemia | -1.41 | -1.63 | -1.19 |
| Both | Serbia | Acute lymphoid leukemia | -0.49 | -0.68 | -0.31 |
| Both | Serbia | Chronic lymphoid leukemia | 2.91 | 2.52 | 3.30 |
| Both | Serbia | Acute myeloid leukemia | 0.38 | 0.13 | 0.62 |
| Both | Serbia | Chronic myeloid leukemia | 0.96 | 0.50 | 1.42 |
| Both | Serbia | Leukemia | 0.01 | -0.16 | 0.19 |
| Both | Serbia | Other leukemia | -1.02 | -1.23 | -0.81 |
| Male | Seychelles | Acute lymphoid leukemia | 0.19 | -0.08 | 0.47 |
| Male | Seychelles | Chronic lymphoid leukemia | 1.75 | 1.45 | 2.06 |
| Male | Seychelles | Acute myeloid leukemia | 1.01 | 0.74 | 1.28 |
| Male | Seychelles | Chronic myeloid leukemia | 0.18 | -0.14 | 0.49 |
| Male | Seychelles | Leukemia | 0.46 | 0.21 | 0.72 |
| Male | Seychelles | Other leukemia | -0.11 | -0.37 | 0.14 |
| Female | Seychelles | Acute lymphoid leukemia | 0.10 | -0.35 | 0.55 |
| Female | Seychelles | Chronic lymphoid leukemia | 2.81 | 2.41 | 3.21 |
| Female | Seychelles | Acute myeloid leukemia | 1.03 | 0.82 | 1.24 |
| Female | Seychelles | Chronic myeloid leukemia | 0.56 | 0.40 | 0.71 |
| Female | Seychelles | Leukemia | 0.72 | 0.53 | 0.91 |
| Female | Seychelles | Other leukemia | 0.42 | 0.34 | 0.51 |
| Both | Seychelles | Acute lymphoid leukemia | 0.26 | -0.02 | 0.55 |
| Both | Seychelles | Chronic lymphoid leukemia | 2.30 | 1.98 | 2.61 |
| Both | Seychelles | Acute myeloid leukemia | 1.08 | 0.87 | 1.30 |
| Both | Seychelles | Chronic myeloid leukemia | 0.34 | 0.12 | 0.56 |
| Both | Seychelles | Leukemia | 0.62 | 0.42 | 0.83 |
| Both | Seychelles | Other leukemia | 0.16 | 0.00 | 0.32 |
| Male | Sierra Leone | Acute lymphoid leukemia | -1.97 | -2.27 | -1.68 |
| Male | Sierra Leone | Chronic lymphoid leukemia | 1.35 | 1.32 | 1.38 |
| Male | Sierra Leone | Acute myeloid leukemia | 0.19 | -0.03 | 0.40 |

| | | | | | |
|---|---|---|---|---|---|
| Male | Sierra Leone | Chronic myeloid leukemia | 0.36 | 0.30 | 0.43 |
| Male | Sierra Leone | Leukemia | 0.15 | 0.07 | 0.23 |
| Male | Sierra Leone | Other leukemia | 0.69 | 0.58 | 0.81 |
| Female | Sierra Leone | Acute lymphoid leukemia | 0.10 | -0.09 | 0.29 |
| Female | Sierra Leone | Chronic lymphoid leukemia | 1.13 | 1.00 | 1.25 |
| Female | Sierra Leone | Acute myeloid leukemia | 0.45 | 0.35 | 0.55 |
| Female | Sierra Leone | Chronic myeloid leukemia | 0.28 | 0.12 | 0.44 |
| Female | Sierra Leone | Leukemia | 0.11 | 0.05 | 0.18 |
| Female | Sierra Leone | Other leukemia | -0.54 | -0.63 | -0.46 |
| Both | Sierra Leone | Acute lymphoid leukemia | -1.31 | -1.58 | -1.04 |
| Both | Sierra Leone | Chronic lymphoid leukemia | 1.26 | 1.16 | 1.37 |
| Both | Sierra Leone | Acute myeloid leukemia | 0.32 | 0.16 | 0.48 |
| Both | Sierra Leone | Chronic myeloid leukemia | 0.24 | 0.14 | 0.35 |
| Both | Sierra Leone | Leukemia | 0.13 | 0.07 | 0.20 |
| Both | Sierra Leone | Other leukemia | 0.15 | 0.08 | 0.22 |
| Male | Singapore | Acute lymphoid leukemia | -2.51 | -2.79 | -2.22 |
| Male | Singapore | Chronic lymphoid leukemia | 3.57 | 2.99 | 4.15 |
| Male | Singapore | Acute myeloid leukemia | 0.68 | 0.47 | 0.89 |
| Male | Singapore | Chronic myeloid leukemia | -3.60 | -3.97 | -3.23 |
| Male | Singapore | Leukemia | -0.31 | -0.45 | -0.17 |
| Male | Singapore | Other leukemia | -0.31 | -0.73 | 0.11 |
| Female | Singapore | Acute lymphoid leukemia | -2.56 | -2.92 | -2.19 |
| Female | Singapore | Chronic lymphoid leukemia | 2.06 | 1.73 | 2.39 |
| Female | Singapore | Acute myeloid leukemia | -0.01 | -0.22 | 0.20 |
| Female | Singapore | Chronic myeloid leukemia | -1.63 | -1.92 | -1.33 |
| Female | Singapore | Leukemia | -0.81 | -0.98 | -0.65 |
| Female | Singapore | Other leukemia | -1.95 | -2.44 | -1.47 |
| Both | Singapore | Acute lymphoid leukemia | -2.61 | -2.90 | -2.32 |
| Both | Singapore | Chronic lymphoid leukemia | 2.97 | 2.55 | 3.39 |
| Both | Singapore | Acute myeloid leukemia | 0.31 | 0.12 | 0.50 |
| Both | Singapore | Chronic myeloid leukemia | -2.82 | -3.09 | -2.55 |
| Both | Singapore | Leukemia | -0.59 | -0.72 | -0.46 |
| Both | Singapore | Other leukemia | -1.15 | -1.55 | -0.75 |
| Male | Slovakia | Acute lymphoid leukemia | -1.73 | -2.06 | -1.39 |
| Male | Slovakia | Chronic lymphoid leukemia | 3.49 | 3.22 | 3.76 |
| Male | Slovakia | Acute myeloid leukemia | 1.72 | 1.51 | 1.93 |
| Male | Slovakia | Chronic myeloid leukemia | -2.73 | -3.24 | -2.22 |
| Male | Slovakia | Leukemia | 2.10 | 1.82 | 2.37 |
| Male | Slovakia | Other leukemia | 5.68 | 4.82 | 6.55 |
| Female | Slovakia | Acute lymphoid leukemia | -0.76 | -0.93 | -0.60 |
| Female | Slovakia | Chronic lymphoid leukemia | 2.27 | 2.10 | 2.44 |
| Female | Slovakia | Acute myeloid leukemia | 0.65 | 0.57 | 0.73 |
| Female | Slovakia | Chronic myeloid leukemia | -3.74 | -4.00 | -3.48 |
| Female | Slovakia | Leukemia | 1.45 | 1.32 | 1.58 |
| Female | Slovakia | Other leukemia | 4.48 | 3.91 | 5.05 |
| Both | Slovakia | Acute lymphoid leukemia | -1.35 | -1.58 | -1.12 |
| Both | Slovakia | Chronic lymphoid leukemia | 3.06 | 2.83 | 3.29 |
| Both | Slovakia | Acute myeloid leukemia | 1.18 | 1.05 | 1.31 |

| | | | | | |
|---|---|---|---|---|---|
| Both | Slovakia | Chronic myeloid leukemia | -3.02 | -3.42 | -2.63 |
| Both | Slovakia | Leukemia | 1.80 | 1.59 | 2.00 |
| Both | Slovakia | Other leukemia | 5.04 | 4.34 | 5.75 |
| Male | Slovenia | Acute lymphoid leukemia | 1.07 | 0.53 | 1.62 |
| Male | Slovenia | Chronic lymphoid leukemia | -0.33 | -0.49 | -0.16 |
| Male | Slovenia | Acute myeloid leukemia | -0.93 | -1.28 | -0.58 |
| Male | Slovenia | Chronic myeloid leukemia | 1.59 | 0.94 | 2.24 |
| Male | Slovenia | Leukemia | -0.05 | -0.24 | 0.15 |
| Male | Slovenia | Other leukemia | -0.94 | -1.39 | -0.49 |
| Female | Slovenia | Acute lymphoid leukemia | -3.89 | -4.27 | -3.50 |
| Female | Slovenia | Chronic lymphoid leukemia | -1.08 | -1.31 | -0.86 |
| Female | Slovenia | Acute myeloid leukemia | 1.86 | 1.33 | 2.39 |
| Female | Slovenia | Chronic myeloid leukemia | -2.96 | -3.41 | -2.51 |
| Female | Slovenia | Leukemia | -0.68 | -0.92 | -0.43 |
| Female | Slovenia | Other leukemia | 0.15 | -0.79 | 1.10 |
| Both | Slovenia | Acute lymphoid leukemia | -0.80 | -1.11 | -0.49 |
| Both | Slovenia | Chronic lymphoid leukemia | -0.55 | -0.72 | -0.38 |
| Both | Slovenia | Acute myeloid leukemia | 0.33 | 0.01 | 0.64 |
| Both | Slovenia | Chronic myeloid leukemia | 0.03 | -0.33 | 0.39 |
| Both | Slovenia | Leukemia | -0.30 | -0.49 | -0.10 |
| Both | Slovenia | Other leukemia | -0.47 | -1.12 | 0.19 |
| Male | Solomon Islands | Acute lymphoid leukemia | -0.12 | -0.20 | -0.05 |
| Male | Solomon Islands | Chronic lymphoid leukemia | -0.85 | -0.96 | -0.74 |
| Male | Solomon Islands | Acute myeloid leukemia | 0.49 | 0.44 | 0.54 |
| Male | Solomon Islands | Chronic myeloid leukemia | -1.37 | -1.45 | -1.30 |
| Male | Solomon Islands | Leukemia | -0.09 | -0.11 | -0.07 |
| Male | Solomon Islands | Other leukemia | -0.20 | -0.24 | -0.16 |
| Female | Solomon Islands | Acute lymphoid leukemia | 0.20 | 0.10 | 0.30 |
| Female | Solomon Islands | Chronic lymphoid leukemia | 0.10 | 0.03 | 0.17 |
| Female | Solomon Islands | Acute myeloid leukemia | -0.31 | -0.37 | -0.25 |
| Female | Solomon Islands | Chronic myeloid leukemia | -1.28 | -1.32 | -1.24 |
| Female | Solomon Islands | Leukemia | -0.46 | -0.50 | -0.43 |
| Female | Solomon Islands | Other leukemia | -0.53 | -0.58 | -0.49 |
| Both | Solomon Islands | Acute lymphoid leukemia | -0.03 | -0.11 | 0.06 |
| Both | Solomon Islands | Chronic lymphoid leukemia | 0.35 | 0.28 | 0.42 |
| Both | Solomon Islands | Acute myeloid leukemia | 0.14 | 0.09 | 0.19 |
| Both | Solomon Islands | Chronic myeloid leukemia | -1.23 | -1.27 | -1.18 |
| Both | Solomon Islands | Leukemia | -0.28 | -0.31 | -0.26 |
| Both | Solomon Islands | Other leukemia | -0.42 | -0.46 | -0.38 |
| Male | Somalia | Acute lymphoid leukemia | 0.14 | -0.21 | 0.50 |
| Male | Somalia | Chronic lymphoid leukemia | 2.90 | 2.76 | 3.04 |
| Male | Somalia | Acute myeloid leukemia | 1.21 | 1.04 | 1.38 |
| Male | Somalia | Chronic myeloid leukemia | 0.33 | 0.17 | 0.49 |
| Male | Somalia | Leukemia | 0.52 | 0.31 | 0.74 |
| Male | Somalia | Other leukemia | -0.56 | -0.83 | -0.30 |
| Female | Somalia | Acute lymphoid leukemia | 2.19 | 1.90 | 2.48 |
| Female | Somalia | Chronic lymphoid leukemia | 0.84 | 0.76 | 0.92 |
| Female | Somalia | Acute myeloid leukemia | 1.84 | 1.67 | 2.02 |

| | | | | | |
|---|---|---|---|---|---|
| Female | Somalia | Chronic myeloid leukemia | 0.25 | 0.14 | 0.35 |
| Female | Somalia | Leukemia | 0.53 | 0.36 | 0.69 |
| Female | Somalia | Other leukemia | -0.36 | -0.57 | -0.14 |
| Both | Somalia | Acute lymphoid leukemia | 0.94 | 0.61 | 1.27 |
| Both | Somalia | Chronic lymphoid leukemia | 1.71 | 1.64 | 1.79 |
| Both | Somalia | Acute myeloid leukemia | 1.29 | 1.13 | 1.45 |
| Both | Somalia | Chronic myeloid leukemia | 0.31 | 0.18 | 0.43 |
| Both | Somalia | Leukemia | 0.51 | 0.32 | 0.70 |
| Both | Somalia | Other leukemia | -0.43 | -0.66 | -0.19 |
| Male | South Africa | Acute lymphoid leukemia | -1.43 | -1.91 | -0.95 |
| Male | South Africa | Chronic lymphoid leukemia | 0.44 | 0.24 | 0.65 |
| Male | South Africa | Acute myeloid leukemia | -0.45 | -0.71 | -0.18 |
| Male | South Africa | Chronic myeloid leukemia | -1.62 | -2.11 | -1.12 |
| Male | South Africa | Leukemia | -0.42 | -0.73 | -0.11 |
| Male | South Africa | Other leukemia | -0.44 | -0.83 | -0.04 |
| Female | South Africa | Acute lymphoid leukemia | 0.25 | -0.10 | 0.61 |
| Female | South Africa | Chronic lymphoid leukemia | 0.27 | -0.19 | 0.73 |
| Female | South Africa | Acute myeloid leukemia | -0.01 | -0.55 | 0.53 |
| Female | South Africa | Chronic myeloid leukemia | -1.89 | -2.65 | -1.12 |
| Female | South Africa | Leukemia | -0.78 | -1.27 | -0.29 |
| Female | South Africa | Other leukemia | -0.93 | -1.42 | -0.43 |
| Both | South Africa | Acute lymphoid leukemia | -0.51 | -0.91 | -0.11 |
| Both | South Africa | Chronic lymphoid leukemia | 0.28 | 0.06 | 0.49 |
| Both | South Africa | Acute myeloid leukemia | -0.48 | -0.77 | -0.19 |
| Both | South Africa | Chronic myeloid leukemia | -1.82 | -2.46 | -1.17 |
| Both | South Africa | Leukemia | -0.64 | -1.03 | -0.24 |
| Both | South Africa | Other leukemia | -0.76 | -1.21 | -0.30 |
| Male | South Asia | Acute lymphoid leukemia | -0.44 | -0.61 | -0.27 |
| Male | South Asia | Chronic lymphoid leukemia | 1.50 | 1.42 | 1.59 |
| Male | South Asia | Acute myeloid leukemia | 0.92 | 0.85 | 1.00 |
| Male | South Asia | Chronic myeloid leukemia | -0.13 | -0.26 | -0.01 |
| Male | South Asia | Leukemia | 0.00 | -0.09 | 0.09 |
| Male | South Asia | Other leukemia | -0.79 | -0.89 | -0.68 |
| Female | South Asia | Acute lymphoid leukemia | 0.35 | 0.20 | 0.49 |
| Female | South Asia | Chronic lymphoid leukemia | 1.32 | 1.10 | 1.54 |
| Female | South Asia | Acute myeloid leukemia | 0.41 | 0.32 | 0.49 |
| Female | South Asia | Chronic myeloid leukemia | -0.99 | -1.11 | -0.87 |
| Female | South Asia | Leukemia | -0.61 | -0.70 | -0.52 |
| Female | South Asia | Other leukemia | -1.88 | -2.01 | -1.75 |
| Both | South Asia | Acute lymphoid leukemia | -0.17 | -0.32 | -0.02 |
| Both | South Asia | Chronic lymphoid leukemia | 1.35 | 1.24 | 1.46 |
| Both | South Asia | Acute myeloid leukemia | 0.66 | 0.59 | 0.74 |
| Both | South Asia | Chronic myeloid leukemia | -0.58 | -0.68 | -0.48 |
| Both | South Asia | Other leukemia | -1.34 | -1.44 | -1.23 |
| Both | South Asia | Leukemia | -0.30 | -0.39 | -0.22 |
| Male | South Korea | Acute lymphoid leukemia | -1.36 | -1.91 | -0.81 |
| Male | South Korea | Chronic lymphoid leukemia | 5.41 | 4.96 | 5.87 |
| Male | South Korea | Acute myeloid leukemia | 0.07 | -0.70 | 0.85 |

| | | | | | |
|---|---|---|---|---|---|
| Male | South Korea | Chronic myeloid leukemia | 0.52 | -0.33 | 1.38 |
| Male | South Korea | Leukemia | 0.14 | -0.01 | 0.29 |
| Male | South Korea | Other leukemia | 0.01 | -0.53 | 0.56 |
| Female | South Korea | Acute lymphoid leukemia | -3.81 | -4.65 | -2.97 |
| Female | South Korea | Chronic lymphoid leukemia | 5.76 | 5.34 | 6.19 |
| Female | South Korea | Acute myeloid leukemia | -0.16 | -0.99 | 0.69 |
| Female | South Korea | Chronic myeloid leukemia | -2.70 | -3.21 | -2.19 |
| Female | South Korea | Leukemia | -0.69 | -0.89 | -0.50 |
| Female | South Korea | Other leukemia | -0.78 | -1.21 | -0.35 |
| Both | South Korea | Acute lymphoid leukemia | -2.31 | -2.95 | -1.67 |
| Both | South Korea | Chronic lymphoid leukemia | 5.54 | 5.24 | 5.84 |
| Both | South Korea | Acute myeloid leukemia | -0.04 | -0.83 | 0.76 |
| Both | South Korea | Chronic myeloid leukemia | -0.61 | -1.31 | 0.09 |
| Both | South Korea | Leukemia | -0.28 | -0.45 | -0.11 |
| Both | South Korea | Other leukemia | -0.40 | -0.89 | 0.09 |
| Male | South Sudan | Acute lymphoid leukemia | 0.97 | 0.56 | 1.39 |
| Male | South Sudan | Chronic lymphoid leukemia | 2.51 | 2.35 | 2.68 |
| Male | South Sudan | Acute myeloid leukemia | 0.91 | 0.74 | 1.08 |
| Male | South Sudan | Chronic myeloid leukemia | 0.67 | 0.51 | 0.84 |
| Male | South Sudan | Leukemia | 0.70 | 0.49 | 0.91 |
| Male | South Sudan | Other leukemia | -0.10 | -0.32 | 0.12 |
| Female | South Sudan | Acute lymphoid leukemia | 1.63 | 1.32 | 1.93 |
| Female | South Sudan | Chronic lymphoid leukemia | 0.22 | 0.18 | 0.27 |
| Female | South Sudan | Acute myeloid leukemia | 1.08 | 0.86 | 1.30 |
| Female | South Sudan | Chronic myeloid leukemia | -0.14 | -0.29 | 0.00 |
| Female | South Sudan | Leukemia | 0.13 | -0.06 | 0.31 |
| Female | South Sudan | Other leukemia | -0.55 | -0.74 | -0.36 |
| Both | South Sudan | Acute lymphoid leukemia | 1.19 | 0.83 | 1.56 |
| Both | South Sudan | Chronic lymphoid leukemia | 1.24 | 1.14 | 1.34 |
| Both | South Sudan | Acute myeloid leukemia | 0.85 | 0.68 | 1.01 |
| Both | South Sudan | Chronic myeloid leukemia | 0.28 | 0.14 | 0.43 |
| Both | South Sudan | Leukemia | 0.44 | 0.25 | 0.64 |
| Both | South Sudan | Other leukemia | -0.32 | -0.52 | -0.12 |
| Male | Southeast Asia | Acute lymphoid leukemia | 0.89 | 0.70 | 1.08 |
| Male | Southeast Asia | Chronic lymphoid leukemia | 2.97 | 2.91 | 3.04 |
| Male | Southeast Asia | Acute myeloid leukemia | 1.66 | 1.57 | 1.75 |
| Male | Southeast Asia | Chronic myeloid leukemia | 0.49 | 0.40 | 0.59 |
| Male | Southeast Asia | Leukemia | 0.42 | 0.33 | 0.52 |
| Male | Southeast Asia | Other leukemia | -0.34 | -0.43 | -0.26 |
| Female | Southeast Asia | Acute lymphoid leukemia | 0.77 | 0.56 | 0.97 |
| Female | Southeast Asia | Chronic lymphoid leukemia | 2.15 | 2.10 | 2.21 |
| Female | Southeast Asia | Acute myeloid leukemia | 1.14 | 1.03 | 1.25 |
| Female | Southeast Asia | Chronic myeloid leukemia | -0.49 | -0.67 | -0.32 |
| Female | Southeast Asia | Leukemia | -0.27 | -0.41 | -0.14 |
| Female | Southeast Asia | Other leukemia | -1.12 | -1.28 | -0.97 |
| Both | Southeast Asia | Acute lymphoid leukemia | 0.83 | 0.63 | 1.03 |
| Both | Southeast Asia | Chronic lymphoid leukemia | 2.60 | 2.55 | 2.66 |
| Both | Southeast Asia | Acute myeloid leukemia | 1.38 | 1.29 | 1.48 |

| | | | | | |
|---|---|---|---|---|---|
| Both | Southeast Asia | Chronic myeloid leukemia | 0.05 | -0.08 | 0.17 |
| Both | Southeast Asia | Leukemia | 0.07 | -0.04 | 0.19 |
| Both | Southeast Asia | Other leukemia | -0.76 | -0.87 | -0.64 |
| Male | Southern Latin America | Acute lymphoid leukemia | -0.38 | -0.43 | -0.33 |
| Male | Southern Latin America | Chronic lymphoid leukemia | 0.02 | -0.22 | 0.26 |
| Male | Southern Latin America | Acute myeloid leukemia | 0.12 | 0.02 | 0.22 |
| Male | Southern Latin America | Chronic myeloid leukemia | -3.24 | -3.63 | -2.86 |
| Male | Southern Latin America | Leukemia | -0.75 | -0.80 | -0.69 |
| Male | Southern Latin America | Other leukemia | -0.97 | -1.10 | -0.84 |
| Female | Southern Latin America | Acute lymphoid leukemia | -0.04 | -0.20 | 0.12 |
| Female | Southern Latin America | Chronic lymphoid leukemia | 0.44 | 0.20 | 0.69 |
| Female | Southern Latin America | Acute myeloid leukemia | 0.28 | 0.17 | 0.39 |
| Female | Southern Latin America | Chronic myeloid leukemia | -3.33 | -3.80 | -2.86 |
| Female | Southern Latin America | Leukemia | -0.54 | -0.64 | -0.44 |
| Female | Southern Latin America | Other leukemia | -0.96 | -1.04 | -0.89 |
| Both | Southern Latin America | Acute lymphoid leukemia | -0.23 | -0.32 | -0.14 |
| Both | Southern Latin America | Chronic lymphoid leukemia | 0.18 | -0.05 | 0.41 |
| Both | Southern Latin America | Acute myeloid leukemia | 0.18 | 0.08 | 0.27 |
| Both | Southern Latin America | Chronic myeloid leukemia | -3.29 | -3.71 | -2.88 |
| Both | Southern Latin America | Other leukemia | -1.00 | -1.11 | -0.89 |
| Both | Southern Latin America | Leukemia | -0.68 | -0.75 | -0.61 |
| Male | Southern Sub-Saharan Africa | Acute lymphoid leukemia | -0.39 | -0.84 | 0.07 |
| Male | Southern Sub-Saharan Africa | Chronic lymphoid leukemia | 0.60 | 0.39 | 0.81 |
| Male | Southern Sub-Saharan Africa | Acute myeloid leukemia | -0.22 | -0.51 | 0.07 |
| Male | Southern Sub-Saharan Africa | Chronic myeloid leukemia | -0.67 | -1.20 | -0.13 |
| Male | Southern Sub-Saharan Africa | Leukemia | -0.13 | -0.45 | 0.19 |
| Male | Southern Sub-Saharan Africa | Other leukemia | -0.11 | -0.50 | 0.27 |
| Female | Southern Sub-Saharan Africa | Acute lymphoid leukemia | 0.19 | -0.12 | 0.49 |
| Female | Southern Sub-Saharan Africa | Chronic lymphoid leukemia | 0.41 | 0.02 | 0.80 |
| Female | Southern Sub-Saharan Africa | Acute myeloid leukemia | 0.21 | -0.28 | 0.71 |
| Female | Southern Sub-Saharan Africa | Chronic myeloid leukemia | -0.81 | -1.48 | -0.14 |
| Female | Southern Sub-Saharan Africa | Leukemia | -0.43 | -0.89 | 0.04 |
| Female | Southern Sub-Saharan Africa | Other leukemia | -0.55 | -1.02 | -0.09 |
| Both | Southern Sub-Saharan Africa | Acute lymphoid leukemia | -0.10 | -0.46 | 0.27 |
| Both | Southern Sub-Saharan Africa | Chronic lymphoid leukemia | 0.39 | 0.17 | 0.61 |
| Both | Southern Sub-Saharan Africa | Acute myeloid leukemia | -0.26 | -0.56 | 0.05 |
| Both | Southern Sub-Saharan Africa | Chronic myeloid leukemia | -0.77 | -1.37 | -0.16 |
| Both | Southern Sub-Saharan Africa | Other leukemia | -0.38 | -0.82 | 0.06 |
| Both | Southern Sub-Saharan Africa | Leukemia | -0.30 | -0.69 | 0.08 |
| Male | Spain | Acute lymphoid leukemia | -2.58 | -2.83 | -2.34 |
| Male | Spain | Chronic lymphoid leukemia | 1.91 | 1.63 | 2.19 |
| Male | Spain | Acute myeloid leukemia | 1.82 | 1.61 | 2.03 |
| Male | Spain | Chronic myeloid leukemia | -3.59 | -3.93 | -3.26 |
| Male | Spain | Leukemia | -0.17 | -0.21 | -0.13 |
| Male | Spain | Other leukemia | -1.26 | -1.60 | -0.93 |
| Female | Spain | Acute lymphoid leukemia | -1.95 | -2.07 | -1.82 |
| Female | Spain | Chronic lymphoid leukemia | 1.67 | 1.41 | 1.93 |
| Female | Spain | Acute myeloid leukemia | 1.92 | 1.71 | 2.14 |

| | | | | | |
|---|---|---|---|---|---|
| Female | Spain | Chronic myeloid leukemia | -5.08 | -5.55 | -4.61 |
| Female | Spain | Leukemia | -0.47 | -0.57 | -0.36 |
| Female | Spain | Other leukemia | -1.97 | -2.32 | -1.61 |
| Both | Spain | Acute lymphoid leukemia | -2.35 | -2.53 | -2.16 |
| Both | Spain | Chronic lymphoid leukemia | 1.89 | 1.61 | 2.17 |
| Both | Spain | Acute myeloid leukemia | 1.87 | 1.67 | 2.07 |
| Both | Spain | Chronic myeloid leukemia | -4.13 | -4.52 | -3.75 |
| Both | Spain | Leukemia | -0.26 | -0.31 | -0.21 |
| Both | Spain | Other leukemia | -1.52 | -1.85 | -1.18 |
| Male | Sri Lanka | Acute lymphoid leukemia | -0.83 | -1.13 | -0.52 |
| Male | Sri Lanka | Chronic lymphoid leukemia | 1.92 | 1.72 | 2.12 |
| Male | Sri Lanka | Acute myeloid leukemia | 1.42 | 1.33 | 1.52 |
| Male | Sri Lanka | Chronic myeloid leukemia | -0.51 | -0.84 | -0.18 |
| Male | Sri Lanka | Leukemia | 0.16 | 0.02 | 0.30 |
| Male | Sri Lanka | Other leukemia | 0.24 | 0.09 | 0.38 |
| Female | Sri Lanka | Acute lymphoid leukemia | -0.67 | -0.90 | -0.45 |
| Female | Sri Lanka | Chronic lymphoid leukemia | 2.03 | 1.70 | 2.36 |
| Female | Sri Lanka | Acute myeloid leukemia | 1.25 | 1.00 | 1.51 |
| Female | Sri Lanka | Chronic myeloid leukemia | -0.09 | -0.35 | 0.16 |
| Female | Sri Lanka | Leukemia | 0.18 | 0.00 | 0.37 |
| Female | Sri Lanka | Other leukemia | 0.00 | -0.30 | 0.30 |
| Both | Sri Lanka | Acute lymphoid leukemia | -0.85 | -1.11 | -0.59 |
| Both | Sri Lanka | Chronic lymphoid leukemia | 1.91 | 1.67 | 2.15 |
| Both | Sri Lanka | Acute myeloid leukemia | 1.28 | 1.12 | 1.44 |
| Both | Sri Lanka | Chronic myeloid leukemia | -0.52 | -0.73 | -0.32 |
| Both | Sri Lanka | Leukemia | 0.09 | -0.02 | 0.19 |
| Both | Sri Lanka | Other leukemia | 0.05 | -0.14 | 0.24 |
| Male | Sudan | Acute lymphoid leukemia | 0.55 | 0.41 | 0.70 |
| Male | Sudan | Chronic lymphoid leukemia | 0.82 | 0.62 | 1.03 |
| Male | Sudan | Acute myeloid leukemia | 0.68 | 0.58 | 0.79 |
| Male | Sudan | Chronic myeloid leukemia | -0.21 | -0.22 | -0.20 |
| Male | Sudan | Leukemia | 0.36 | 0.26 | 0.46 |
| Male | Sudan | Other leukemia | 0.22 | 0.11 | 0.34 |
| Female | Sudan | Acute lymphoid leukemia | 0.63 | 0.46 | 0.81 |
| Female | Sudan | Chronic lymphoid leukemia | 0.37 | 0.22 | 0.51 |
| Female | Sudan | Acute myeloid leukemia | -0.33 | -0.40 | -0.26 |
| Female | Sudan | Chronic myeloid leukemia | -1.98 | -2.04 | -1.91 |
| Female | Sudan | Leukemia | -0.66 | -0.74 | -0.57 |
| Female | Sudan | Other leukemia | -0.93 | -1.03 | -0.83 |
| Both | Sudan | Acute lymphoid leukemia | 0.60 | 0.44 | 0.76 |
| Both | Sudan | Chronic lymphoid leukemia | 0.72 | 0.55 | 0.89 |
| Both | Sudan | Acute myeloid leukemia | 0.21 | 0.13 | 0.30 |
| Both | Sudan | Chronic myeloid leukemia | -1.15 | -1.17 | -1.12 |
| Both | Sudan | Leukemia | -0.05 | -0.15 | 0.05 |
| Both | Sudan | Other leukemia | -0.22 | -0.33 | -0.11 |
| Male | Suriname | Acute lymphoid leukemia | -0.46 | -0.60 | -0.32 |
| Male | Suriname | Chronic lymphoid leukemia | 2.75 | 2.49 | 3.00 |
| Male | Suriname | Acute myeloid leukemia | 0.56 | 0.48 | 0.65 |

| | | | | | |
|---|---|---|---|---|---|
| Male | Suriname | Chronic myeloid leukemia | -0.71 | -0.88 | -0.54 |
| Male | Suriname | Leukemia | -0.30 | -0.41 | -0.18 |
| Male | Suriname | Other leukemia | -0.78 | -0.93 | -0.63 |
| Female | Suriname | Acute lymphoid leukemia | -0.48 | -0.74 | -0.21 |
| Female | Suriname | Chronic lymphoid leukemia | 2.87 | 2.56 | 3.18 |
| Female | Suriname | Acute myeloid leukemia | 0.46 | 0.31 | 0.61 |
| Female | Suriname | Chronic myeloid leukemia | -1.45 | -1.66 | -1.25 |
| Female | Suriname | Leukemia | -0.64 | -0.73 | -0.55 |
| Female | Suriname | Other leukemia | -1.26 | -1.34 | -1.19 |
| Both | Suriname | Acute lymphoid leukemia | -0.48 | -0.67 | -0.29 |
| Both | Suriname | Chronic lymphoid leukemia | 2.82 | 2.54 | 3.10 |
| Both | Suriname | Acute myeloid leukemia | 0.50 | 0.41 | 0.58 |
| Both | Suriname | Chronic myeloid leukemia | -1.08 | -1.26 | -0.89 |
| Both | Suriname | Leukemia | -0.47 | -0.56 | -0.38 |
| Both | Suriname | Other leukemia | -1.01 | -1.12 | -0.91 |
| Male | Swaziland | Acute lymphoid leukemia | 1.05 | 0.61 | 1.50 |
| Male | Swaziland | Chronic lymphoid leukemia | 1.40 | 1.21 | 1.60 |
| Male | Swaziland | Acute myeloid leukemia | 0.54 | 0.24 | 0.85 |
| Male | Swaziland | Chronic myeloid leukemia | 1.03 | 0.58 | 1.49 |
| Male | Swaziland | Leukemia | 0.61 | 0.28 | 0.93 |
| Male | Swaziland | Other leukemia | 0.49 | 0.11 | 0.87 |
| Female | Swaziland | Acute lymphoid leukemia | 0.52 | 0.11 | 0.94 |
| Female | Swaziland | Chronic lymphoid leukemia | 0.84 | 0.53 | 1.16 |
| Female | Swaziland | Acute myeloid leukemia | 0.71 | 0.22 | 1.20 |
| Female | Swaziland | Chronic myeloid leukemia | 0.32 | -0.23 | 0.88 |
| Female | Swaziland | Leukemia | -0.05 | -0.47 | 0.38 |
| Female | Swaziland | Other leukemia | -0.23 | -0.65 | 0.19 |
| Both | Swaziland | Acute lymphoid leukemia | 0.71 | 0.31 | 1.13 |
| Both | Swaziland | Chronic lymphoid leukemia | 1.04 | 0.82 | 1.26 |
| Both | Swaziland | Acute myeloid leukemia | 0.49 | 0.16 | 0.82 |
| Both | Swaziland | Chronic myeloid leukemia | 0.60 | 0.08 | 1.13 |
| Both | Swaziland | Leukemia | 0.29 | -0.09 | 0.67 |
| Both | Swaziland | Other leukemia | 0.09 | -0.32 | 0.51 |
| Male | Sweden | Acute lymphoid leukemia | -1.35 | -1.47 | -1.23 |
| Male | Sweden | Chronic lymphoid leukemia | 1.84 | 1.46 | 2.22 |
| Male | Sweden | Acute myeloid leukemia | -0.47 | -0.68 | -0.25 |
| Male | Sweden | Chronic myeloid leukemia | -2.69 | -2.96 | -2.42 |
| Male | Sweden | Leukemia | 0.17 | 0.00 | 0.34 |
| Male | Sweden | Other leukemia | -0.16 | -0.25 | -0.08 |
| Female | Sweden | Acute lymphoid leukemia | -1.23 | -1.40 | -1.06 |
| Female | Sweden | Chronic lymphoid leukemia | 1.96 | 1.73 | 2.20 |
| Female | Sweden | Acute myeloid leukemia | 0.17 | 0.01 | 0.33 |
| Female | Sweden | Chronic myeloid leukemia | -3.40 | -3.75 | -3.05 |
| Female | Sweden | Leukemia | 0.21 | 0.10 | 0.32 |
| Female | Sweden | Other leukemia | -0.19 | -0.32 | -0.06 |
| Both | Sweden | Acute lymphoid leukemia | -1.29 | -1.41 | -1.17 |
| Both | Sweden | Chronic lymphoid leukemia | 1.97 | 1.66 | 2.29 |
| Both | Sweden | Acute myeloid leukemia | -0.14 | -0.32 | 0.04 |

| | | | | | |
|---|---|---|---|---|---|
| Both | Sweden | Chronic myeloid leukemia | -2.95 | -3.24 | -2.65 |
| Both | Sweden | Leukemia | 0.24 | 0.11 | 0.37 |
| Both | Sweden | Other leukemia | -0.08 | -0.17 | 0.00 |
| Male | Switzerland | Acute lymphoid leukemia | -2.42 | -2.95 | -1.89 |
| Male | Switzerland | Chronic lymphoid leukemia | 0.26 | -0.22 | 0.74 |
| Male | Switzerland | Acute myeloid leukemia | 0.71 | 0.42 | 1.01 |
| Male | Switzerland | Chronic myeloid leukemia | -2.89 | -3.40 | -2.37 |
| Male | Switzerland | Leukemia | -1.02 | -1.34 | -0.70 |
| Male | Switzerland | Other leukemia | -2.48 | -2.84 | -2.11 |
| Female | Switzerland | Acute lymphoid leukemia | -1.00 | -1.57 | -0.43 |
| Female | Switzerland | Chronic lymphoid leukemia | 1.06 | 0.43 | 1.69 |
| Female | Switzerland | Acute myeloid leukemia | 1.03 | 0.70 | 1.36 |
| Female | Switzerland | Chronic myeloid leukemia | -4.48 | -5.16 | -3.80 |
| Female | Switzerland | Leukemia | -0.75 | -1.09 | -0.42 |
| Female | Switzerland | Other leukemia | -2.48 | -2.84 | -2.12 |
| Both | Switzerland | Acute lymphoid leukemia | -1.85 | -2.38 | -1.31 |
| Both | Switzerland | Chronic lymphoid leukemia | 0.70 | 0.16 | 1.23 |
| Both | Switzerland | Acute myeloid leukemia | 0.88 | 0.58 | 1.19 |
| Both | Switzerland | Chronic myeloid leukemia | -3.48 | -4.03 | -2.92 |
| Both | Switzerland | Leukemia | -0.85 | -1.17 | -0.53 |
| Both | Switzerland | Other leukemia | -2.43 | -2.78 | -2.07 |
| Male | Syria | Acute lymphoid leukemia | -1.15 | -1.58 | -0.72 |
| Male | Syria | Chronic lymphoid leukemia | 1.37 | 1.06 | 1.67 |
| Male | Syria | Acute myeloid leukemia | -0.43 | -0.89 | 0.03 |
| Male | Syria | Chronic myeloid leukemia | -1.03 | -1.34 | -0.71 |
| Male | Syria | Leukemia | -0.78 | -1.11 | -0.45 |
| Male | Syria | Other leukemia | -0.94 | -1.25 | -0.64 |
| Female | Syria | Acute lymphoid leukemia | -0.89 | -1.33 | -0.44 |
| Female | Syria | Chronic lymphoid leukemia | 0.69 | 0.41 | 0.98 |
| Female | Syria | Acute myeloid leukemia | -1.34 | -1.71 | -0.96 |
| Female | Syria | Chronic myeloid leukemia | -3.20 | -3.54 | -2.85 |
| Female | Syria | Leukemia | -1.95 | -2.23 | -1.66 |
| Female | Syria | Other leukemia | -2.36 | -2.61 | -2.11 |
| Both | Syria | Acute lymphoid leukemia | -1.06 | -1.46 | -0.65 |
| Both | Syria | Chronic lymphoid leukemia | 1.05 | 0.76 | 1.35 |
| Both | Syria | Acute myeloid leukemia | -0.85 | -1.25 | -0.44 |
| Both | Syria | Chronic myeloid leukemia | -2.03 | -2.35 | -1.72 |
| Both | Syria | Leukemia | -1.26 | -1.56 | -0.95 |
| Both | Syria | Other leukemia | -1.50 | -1.77 | -1.22 |
| Male | Taiwan (Province of China) | Acute lymphoid leukemia | -0.34 | -0.51 | -0.16 |
| Male | Taiwan (Province of China) | Chronic lymphoid leukemia | 5.91 | 5.77 | 6.05 |
| Male | Taiwan (Province of China) | Acute myeloid leukemia | 2.36 | 2.23 | 2.48 |
| Male | Taiwan (Province of China) | Chronic myeloid leukemia | -0.08 | -0.40 | 0.25 |
| Male | Taiwan (Province of China) | Leukemia | 1.60 | 1.48 | 1.72 |
| Male | Taiwan (Province of China) | Other leukemia | 1.06 | 0.87 | 1.26 |
| Female | Taiwan (Province of China) | Acute lymphoid leukemia | 0.34 | 0.14 | 0.54 |
| Female | Taiwan (Province of China) | Chronic lymphoid leukemia | 4.63 | 4.48 | 4.77 |
| Female | Taiwan (Province of China) | Acute myeloid leukemia | 1.30 | 1.12 | 1.48 |

| | | | | | |
|---|---|---|---|---|---|
| Female | Taiwan (Province of China) | Chronic myeloid leukemia | 0.60 | 0.48 | 0.72 |
| Female | Taiwan (Province of China) | Leukemia | 1.23 | 1.08 | 1.38 |
| Female | Taiwan (Province of China) | Other leukemia | 0.93 | 0.72 | 1.14 |
| Both | Taiwan (Province of China) | Acute lymphoid leukemia | -0.10 | -0.27 | 0.06 |
| Both | Taiwan (Province of China) | Chronic lymphoid leukemia | 5.27 | 5.15 | 5.39 |
| Both | Taiwan (Province of China) | Acute myeloid leukemia | 1.84 | 1.70 | 1.98 |
| Both | Taiwan (Province of China) | Chronic myeloid leukemia | 0.08 | -0.15 | 0.30 |
| Both | Taiwan (Province of China) | Leukemia | 1.39 | 1.26 | 1.52 |
| Both | Taiwan (Province of China) | Other leukemia | 0.95 | 0.76 | 1.15 |
| Male | Tajikistan | Acute lymphoid leukemia | -0.28 | -0.61 | 0.04 |
| Male | Tajikistan | Chronic lymphoid leukemia | 0.04 | -0.35 | 0.43 |
| Male | Tajikistan | Acute myeloid leukemia | 0.40 | 0.05 | 0.75 |
| Male | Tajikistan | Chronic myeloid leukemia | -3.46 | -3.88 | -3.04 |
| Male | Tajikistan | Leukemia | -0.77 | -1.10 | -0.45 |
| Male | Tajikistan | Other leukemia | -1.30 | -1.64 | -0.96 |
| Female | Tajikistan | Acute lymphoid leukemia | -0.12 | -0.37 | 0.13 |
| Female | Tajikistan | Chronic lymphoid leukemia | -0.73 | -1.18 | -0.27 |
| Female | Tajikistan | Acute myeloid leukemia | 0.58 | 0.31 | 0.85 |
| Female | Tajikistan | Chronic myeloid leukemia | -1.37 | -1.54 | -1.19 |
| Female | Tajikistan | Leukemia | -0.69 | -0.92 | -0.46 |
| Female | Tajikistan | Other leukemia | -1.48 | -1.70 | -1.26 |
| Both | Tajikistan | Acute lymphoid leukemia | -0.19 | -0.48 | 0.11 |
| Both | Tajikistan | Chronic lymphoid leukemia | -0.25 | -0.66 | 0.16 |
| Both | Tajikistan | Acute myeloid leukemia | 0.48 | 0.16 | 0.80 |
| Both | Tajikistan | Chronic myeloid leukemia | -2.51 | -2.82 | -2.19 |
| Both | Tajikistan | Leukemia | -0.71 | -1.00 | -0.42 |
| Both | Tajikistan | Other leukemia | -1.35 | -1.64 | -1.06 |
| Male | Tanzania | Acute lymphoid leukemia | 0.70 | 0.52 | 0.89 |
| Male | Tanzania | Chronic lymphoid leukemia | 2.62 | 2.46 | 2.77 |
| Male | Tanzania | Acute myeloid leukemia | 0.94 | 0.82 | 1.06 |
| Male | Tanzania | Chronic myeloid leukemia | 0.17 | 0.04 | 0.30 |
| Male | Tanzania | Leukemia | 0.64 | 0.52 | 0.76 |
| Male | Tanzania | Other leukemia | -0.23 | -0.33 | -0.14 |
| Female | Tanzania | Acute lymphoid leukemia | 2.18 | 2.06 | 2.31 |
| Female | Tanzania | Chronic lymphoid leukemia | 0.48 | 0.38 | 0.59 |
| Female | Tanzania | Acute myeloid leukemia | 1.31 | 1.23 | 1.40 |
| Female | Tanzania | Chronic myeloid leukemia | -0.95 | -1.09 | -0.80 |
| Female | Tanzania | Leukemia | 0.15 | 0.07 | 0.23 |
| Female | Tanzania | Other leukemia | -0.82 | -0.95 | -0.70 |
| Both | Tanzania | Acute lymphoid leukemia | 1.34 | 1.19 | 1.49 |
| Both | Tanzania | Chronic lymphoid leukemia | 1.36 | 1.25 | 1.46 |
| Both | Tanzania | Acute myeloid leukemia | 0.98 | 0.88 | 1.08 |
| Both | Tanzania | Chronic myeloid leukemia | -0.51 | -0.64 | -0.38 |
| Both | Tanzania | Leukemia | 0.40 | 0.31 | 0.50 |
| Both | Tanzania | Other leukemia | -0.57 | -0.66 | -0.47 |
| Male | Thailand | Acute lymphoid leukemia | -0.30 | -0.54 | -0.05 |
| Male | Thailand | Chronic lymphoid leukemia | 2.95 | 2.85 | 3.04 |
| Male | Thailand | Acute myeloid leukemia | 1.35 | 1.20 | 1.51 |

| | | | | | |
|---|---|---|---|---|---|
| Male | Thailand | Chronic myeloid leukemia | -0.39 | -0.62 | -0.15 |
| Male | Thailand | Leukemia | -0.10 | -0.26 | 0.05 |
| Male | Thailand | Other leukemia | -0.67 | -0.81 | -0.53 |
| Female | Thailand | Acute lymphoid leukemia | -0.08 | -0.33 | 0.18 |
| Female | Thailand | Chronic lymphoid leukemia | 3.04 | 2.86 | 3.22 |
| Female | Thailand | Acute myeloid leukemia | 0.99 | 0.82 | 1.17 |
| Female | Thailand | Chronic myeloid leukemia | -0.75 | -1.03 | -0.46 |
| Female | Thailand | Leukemia | -0.63 | -0.89 | -0.36 |
| Female | Thailand | Other leukemia | -1.50 | -1.84 | -1.17 |
| Both | Thailand | Acute lymphoid leukemia | -0.21 | -0.44 | 0.02 |
| Both | Thailand | Chronic lymphoid leukemia | 3.00 | 2.92 | 3.09 |
| Both | Thailand | Acute myeloid leukemia | 1.17 | 1.02 | 1.31 |
| Both | Thailand | Chronic myeloid leukemia | -0.51 | -0.73 | -0.29 |
| Both | Thailand | Leukemia | -0.35 | -0.53 | -0.17 |
| Both | Thailand | Other leukemia | -1.07 | -1.29 | -0.85 |
| Male | Timor-Leste | Acute lymphoid leukemia | 2.00 | 1.86 | 2.14 |
| Male | Timor-Leste | Chronic lymphoid leukemia | 4.04 | 3.81 | 4.27 |
| Male | Timor-Leste | Acute myeloid leukemia | 2.65 | 2.54 | 2.75 |
| Male | Timor-Leste | Chronic myeloid leukemia | 0.98 | 0.88 | 1.08 |
| Male | Timor-Leste | Leukemia | 0.94 | 0.82 | 1.05 |
| Male | Timor-Leste | Other leukemia | 0.10 | -0.03 | 0.22 |
| Female | Timor-Leste | Acute lymphoid leukemia | 1.89 | 1.77 | 2.00 |
| Female | Timor-Leste | Chronic lymphoid leukemia | 2.39 | 2.25 | 2.53 |
| Female | Timor-Leste | Acute myeloid leukemia | 1.11 | 0.99 | 1.24 |
| Female | Timor-Leste | Chronic myeloid leukemia | -0.67 | -0.75 | -0.58 |
| Female | Timor-Leste | Leukemia | -0.44 | -0.53 | -0.34 |
| Female | Timor-Leste | Other leukemia | -1.17 | -1.28 | -1.06 |
| Both | Timor-Leste | Acute lymphoid leukemia | 1.94 | 1.81 | 2.06 |
| Both | Timor-Leste | Chronic lymphoid leukemia | 3.38 | 3.20 | 3.56 |
| Both | Timor-Leste | Acute myeloid leukemia | 1.90 | 1.80 | 2.00 |
| Both | Timor-Leste | Chronic myeloid leukemia | 0.13 | 0.07 | 0.19 |
| Both | Timor-Leste | Leukemia | 0.28 | 0.18 | 0.37 |
| Both | Timor-Leste | Other leukemia | -0.55 | -0.64 | -0.45 |
| Male | Togo | Acute lymphoid leukemia | -1.72 | -1.87 | -1.57 |
| Male | Togo | Chronic lymphoid leukemia | 1.25 | 1.16 | 1.34 |
| Male | Togo | Acute myeloid leukemia | -0.12 | -0.25 | 0.01 |
| Male | Togo | Chronic myeloid leukemia | 0.61 | 0.45 | 0.77 |
| Male | Togo | Leukemia | 0.18 | 0.07 | 0.30 |
| Male | Togo | Other leukemia | 0.76 | 0.58 | 0.95 |
| Female | Togo | Acute lymphoid leukemia | -0.14 | -0.24 | -0.05 |
| Female | Togo | Chronic lymphoid leukemia | -0.06 | -0.13 | 0.01 |
| Female | Togo | Acute myeloid leukemia | -0.10 | -0.22 | 0.02 |
| Female | Togo | Chronic myeloid leukemia | -0.92 | -0.99 | -0.85 |
| Female | Togo | Leukemia | -0.60 | -0.69 | -0.51 |
| Female | Togo | Other leukemia | -1.14 | -1.26 | -1.03 |
| Both | Togo | Acute lymphoid leukemia | -1.28 | -1.40 | -1.16 |
| Both | Togo | Chronic lymphoid leukemia | 0.46 | 0.37 | 0.54 |
| Both | Togo | Acute myeloid leukemia | -0.09 | -0.20 | 0.03 |

| | | | | | |
|---|---|---|---|---|---|
| Both | Togo | Chronic myeloid leukemia | -0.39 | -0.50 | -0.27 |
| Both | Togo | Leukemia | -0.22 | -0.33 | -0.12 |
| Both | Togo | Other leukemia | -0.11 | -0.25 | 0.03 |
| Male | Tonga | Acute lymphoid leukemia | 0.46 | 0.34 | 0.58 |
| Male | Tonga | Chronic lymphoid leukemia | -0.31 | -0.53 | -0.10 |
| Male | Tonga | Acute myeloid leukemia | 0.84 | 0.65 | 1.03 |
| Male | Tonga | Chronic myeloid leukemia | -0.61 | -0.73 | -0.49 |
| Male | Tonga | Leukemia | 0.33 | 0.21 | 0.46 |
| Male | Tonga | Other leukemia | 0.00 | -0.10 | 0.11 |
| Female | Tonga | Acute lymphoid leukemia | 0.36 | 0.17 | 0.54 |
| Female | Tonga | Chronic lymphoid leukemia | 0.56 | 0.50 | 0.62 |
| Female | Tonga | Acute myeloid leukemia | -0.15 | -0.30 | 0.01 |
| Female | Tonga | Chronic myeloid leukemia | -1.56 | -1.68 | -1.45 |
| Female | Tonga | Leukemia | -0.28 | -0.37 | -0.19 |
| Female | Tonga | Other leukemia | -0.56 | -0.63 | -0.49 |
| Both | Tonga | Acute lymphoid leukemia | 0.40 | 0.27 | 0.53 |
| Both | Tonga | Chronic lymphoid leukemia | 0.54 | 0.49 | 0.60 |
| Both | Tonga | Acute myeloid leukemia | 0.59 | 0.43 | 0.76 |
| Both | Tonga | Chronic myeloid leukemia | -1.05 | -1.12 | -0.98 |
| Both | Tonga | Leukemia | 0.10 | 0.02 | 0.18 |
| Both | Tonga | Other leukemia | -0.25 | -0.30 | -0.19 |
| Male | Trinidad and Tobago | Acute lymphoid leukemia | -0.70 | -0.91 | -0.50 |
| Male | Trinidad and Tobago | Chronic lymphoid leukemia | 0.79 | 0.33 | 1.26 |
| Male | Trinidad and Tobago | Acute myeloid leukemia | -0.99 | -1.17 | -0.80 |
| Male | Trinidad and Tobago | Chronic myeloid leukemia | -1.18 | -1.49 | -0.87 |
| Male | Trinidad and Tobago | Leukemia | -0.66 | -0.86 | -0.47 |
| Male | Trinidad and Tobago | Other leukemia | -0.42 | -0.76 | -0.08 |
| Female | Trinidad and Tobago | Acute lymphoid leukemia | -1.89 | -2.28 | -1.49 |
| Female | Trinidad and Tobago | Chronic lymphoid leukemia | -0.72 | -1.45 | 0.01 |
| Female | Trinidad and Tobago | Acute myeloid leukemia | -0.25 | -0.49 | -0.01 |
| Female | Trinidad and Tobago | Chronic myeloid leukemia | -4.15 | -4.69 | -3.62 |
| Female | Trinidad and Tobago | Leukemia | -1.45 | -1.63 | -1.27 |
| Female | Trinidad and Tobago | Other leukemia | -1.52 | -1.66 | -1.37 |
| Both | Trinidad and Tobago | Acute lymphoid leukemia | -1.25 | -1.51 | -0.99 |
| Both | Trinidad and Tobago | Chronic lymphoid leukemia | 0.28 | -0.13 | 0.69 |
| Both | Trinidad and Tobago | Acute myeloid leukemia | -0.65 | -0.81 | -0.48 |
| Both | Trinidad and Tobago | Chronic myeloid leukemia | -2.38 | -2.59 | -2.16 |
| Both | Trinidad and Tobago | Leukemia | -1.02 | -1.16 | -0.88 |
| Both | Trinidad and Tobago | Other leukemia | -0.99 | -1.16 | -0.81 |
| Male | Tropical Latin America | Acute lymphoid leukemia | -0.24 | -0.44 | -0.03 |
| Male | Tropical Latin America | Chronic lymphoid leukemia | 1.37 | 1.25 | 1.50 |
| Male | Tropical Latin America | Acute myeloid leukemia | 0.07 | -0.01 | 0.16 |
| Male | Tropical Latin America | Chronic myeloid leukemia | -2.52 | -2.92 | -2.12 |
| Male | Tropical Latin America | Leukemia | -0.46 | -0.54 | -0.37 |
| Male | Tropical Latin America | Other leukemia | -1.03 | -1.10 | -0.96 |
| Female | Tropical Latin America | Acute lymphoid leukemia | -0.56 | -0.78 | -0.34 |
| Female | Tropical Latin America | Chronic lymphoid leukemia | 0.95 | 0.79 | 1.11 |
| Female | Tropical Latin America | Acute myeloid leukemia | 0.19 | 0.07 | 0.32 |

| | | | | | |
|---|---|---|---|---|---|
| Female | Tropical Latin America | Chronic myeloid leukemia | -3.44 | -3.86 | -3.01 |
| Female | Tropical Latin America | Leukemia | -0.71 | -0.81 | -0.60 |
| Female | Tropical Latin America | Other leukemia | -1.40 | -1.45 | -1.35 |
| Both | Tropical Latin America | Acute lymphoid leukemia | -0.37 | -0.57 | -0.17 |
| Both | Tropical Latin America | Chronic lymphoid leukemia | 1.17 | 1.05 | 1.29 |
| Both | Tropical Latin America | Acute myeloid leukemia | 0.11 | 0.02 | 0.21 |
| Both | Tropical Latin America | Chronic myeloid leukemia | -2.93 | -3.34 | -2.53 |
| Both | Tropical Latin America | Other leukemia | -1.22 | -1.27 | -1.16 |
| Both | Tropical Latin America | Leukemia | -0.58 | -0.67 | -0.49 |
| Male | Tunisia | Acute lymphoid leukemia | -0.14 | -0.24 | -0.05 |
| Male | Tunisia | Chronic lymphoid leukemia | 1.30 | 1.13 | 1.48 |
| Male | Tunisia | Acute myeloid leukemia | -0.19 | -0.33 | -0.04 |
| Male | Tunisia | Chronic myeloid leukemia | -0.01 | -0.14 | 0.13 |
| Male | Tunisia | Leukemia | -0.15 | -0.24 | -0.06 |
| Male | Tunisia | Other leukemia | -0.26 | -0.34 | -0.18 |
| Female | Tunisia | Acute lymphoid leukemia | -0.66 | -0.74 | -0.58 |
| Female | Tunisia | Chronic lymphoid leukemia | 1.51 | 1.26 | 1.77 |
| Female | Tunisia | Acute myeloid leukemia | -0.40 | -0.48 | -0.32 |
| Female | Tunisia | Chronic myeloid leukemia | -1.60 | -1.72 | -1.48 |
| Female | Tunisia | Leukemia | -0.99 | -1.04 | -0.95 |
| Female | Tunisia | Other leukemia | -1.31 | -1.36 | -1.27 |
| Both | Tunisia | Acute lymphoid leukemia | -0.38 | -0.46 | -0.29 |
| Both | Tunisia | Chronic lymphoid leukemia | 1.31 | 1.13 | 1.49 |
| Both | Tunisia | Acute myeloid leukemia | -0.30 | -0.41 | -0.19 |
| Both | Tunisia | Chronic myeloid leukemia | -0.69 | -0.82 | -0.57 |
| Both | Tunisia | Leukemia | -0.54 | -0.61 | -0.47 |
| Both | Tunisia | Other leukemia | -0.73 | -0.79 | -0.67 |
| Male | Turkey | Acute lymphoid leukemia | -1.18 | -1.25 | -1.12 |
| Male | Turkey | Chronic lymphoid leukemia | 2.88 | 2.47 | 3.28 |
| Male | Turkey | Acute myeloid leukemia | -0.13 | -0.29 | 0.03 |
| Male | Turkey | Chronic myeloid leukemia | -2.40 | -2.66 | -2.13 |
| Male | Turkey | Leukemia | -0.50 | -0.64 | -0.36 |
| Male | Turkey | Other leukemia | -1.22 | -1.34 | -1.10 |
| Female | Turkey | Acute lymphoid leukemia | -1.29 | -1.47 | -1.11 |
| Female | Turkey | Chronic lymphoid leukemia | 1.69 | 1.41 | 1.97 |
| Female | Turkey | Acute myeloid leukemia | -1.66 | -1.78 | -1.54 |
| Female | Turkey | Chronic myeloid leukemia | -4.51 | -4.69 | -4.34 |
| Female | Turkey | Leukemia | -1.61 | -1.73 | -1.49 |
| Female | Turkey | Other leukemia | -2.18 | -2.36 | -2.00 |
| Both | Turkey | Acute lymphoid leukemia | -1.24 | -1.34 | -1.14 |
| Both | Turkey | Chronic lymphoid leukemia | 2.40 | 2.06 | 2.74 |
| Both | Turkey | Acute myeloid leukemia | -0.88 | -1.02 | -0.74 |
| Both | Turkey | Chronic myeloid leukemia | -3.29 | -3.51 | -3.08 |
| Both | Turkey | Leukemia | -1.00 | -1.12 | -0.88 |
| Both | Turkey | Other leukemia | -1.64 | -1.77 | -1.51 |
| Male | Turkmenistan | Acute lymphoid leukemia | 0.57 | 0.28 | 0.86 |
| Male | Turkmenistan | Chronic lymphoid leukemia | 1.34 | 1.19 | 1.49 |
| Male | Turkmenistan | Acute myeloid leukemia | 1.16 | 0.91 | 1.42 |

| | | | | | |
|---|---|---|---|---|---|
| Male | Turkmenistan | Chronic myeloid leukemia | -1.83 | -2.07 | -1.58 |
| Male | Turkmenistan | Leukemia | -0.08 | -0.33 | 0.17 |
| Male | Turkmenistan | Other leukemia | -1.05 | -1.33 | -0.76 |
| Female | Turkmenistan | Acute lymphoid leukemia | -0.56 | -0.78 | -0.34 |
| Female | Turkmenistan | Chronic lymphoid leukemia | 0.86 | 0.58 | 1.14 |
| Female | Turkmenistan | Acute myeloid leukemia | 0.56 | 0.27 | 0.85 |
| Female | Turkmenistan | Chronic myeloid leukemia | -0.71 | -0.95 | -0.46 |
| Female | Turkmenistan | Leukemia | -0.50 | -0.72 | -0.28 |
| Female | Turkmenistan | Other leukemia | -1.23 | -1.43 | -1.02 |
| Both | Turkmenistan | Acute lymphoid leukemia | 0.20 | -0.03 | 0.43 |
| Both | Turkmenistan | Chronic lymphoid leukemia | 1.08 | 0.87 | 1.29 |
| Both | Turkmenistan | Acute myeloid leukemia | 0.92 | 0.68 | 1.17 |
| Both | Turkmenistan | Chronic myeloid leukemia | -1.21 | -1.43 | -0.98 |
| Both | Turkmenistan | Leukemia | -0.22 | -0.44 | 0.00 |
| Both | Turkmenistan | Other leukemia | -1.07 | -1.31 | -0.84 |
| Male | Uganda | Acute lymphoid leukemia | 1.24 | 0.94 | 1.53 |
| Male | Uganda | Chronic lymphoid leukemia | 2.73 | 2.59 | 2.88 |
| Male | Uganda | Acute myeloid leukemia | 0.90 | 0.68 | 1.12 |
| Male | Uganda | Chronic myeloid leukemia | 1.78 | 1.53 | 2.02 |
| Male | Uganda | Leukemia | 0.95 | 0.81 | 1.10 |
| Male | Uganda | Other leukemia | 0.32 | 0.20 | 0.44 |
| Female | Uganda | Acute lymphoid leukemia | 1.17 | 0.94 | 1.40 |
| Female | Uganda | Chronic lymphoid leukemia | -0.04 | -0.17 | 0.08 |
| Female | Uganda | Acute myeloid leukemia | 0.78 | 0.61 | 0.94 |
| Female | Uganda | Chronic myeloid leukemia | -1.30 | -1.44 | -1.17 |
| Female | Uganda | Leukemia | -0.56 | -0.66 | -0.45 |
| Female | Uganda | Other leukemia | -1.43 | -1.54 | -1.32 |
| Both | Uganda | Acute lymphoid leukemia | 1.20 | 0.93 | 1.46 |
| Both | Uganda | Chronic lymphoid leukemia | 1.07 | 0.96 | 1.17 |
| Both | Uganda | Acute myeloid leukemia | 0.57 | 0.36 | 0.78 |
| Both | Uganda | Chronic myeloid leukemia | -0.14 | -0.28 | 0.00 |
| Both | Uganda | Leukemia | 0.24 | 0.13 | 0.36 |
| Both | Uganda | Other leukemia | -0.61 | -0.71 | -0.50 |
| Male | UK | Acute lymphoid leukemia | -3.15 | -3.35 | -2.96 |
| Male | UK | Chronic lymphoid leukemia | -0.58 | -0.92 | -0.24 |
| Male | UK | Acute myeloid leukemia | -0.02 | -0.14 | 0.11 |
| Male | UK | Chronic myeloid leukemia | -5.03 | -5.56 | -4.49 |
| Male | UK | Leukemia | -0.86 | -1.04 | -0.69 |
| Male | UK | Other leukemia | 0.16 | -0.02 | 0.34 |
| Female | UK | Acute lymphoid leukemia | -2.66 | -2.85 | -2.46 |
| Female | UK | Chronic lymphoid leukemia | -0.11 | -0.42 | 0.20 |
| Female | UK | Acute myeloid leukemia | -0.42 | -0.52 | -0.31 |
| Female | UK | Chronic myeloid leukemia | -5.31 | -5.87 | -4.75 |
| Female | UK | Leukemia | -0.87 | -1.03 | -0.72 |
| Female | UK | Other leukemia | -0.18 | -0.30 | -0.06 |
| Both | UK | Acute lymphoid leukemia | -2.95 | -3.13 | -2.76 |
| Both | UK | Chronic lymphoid leukemia | -0.30 | -0.63 | 0.04 |
| Both | UK | Acute myeloid leukemia | -0.15 | -0.26 | -0.04 |

| | | | | | |
|---|---|---|---|---|---|
| Both | UK | Chronic myeloid leukemia | -5.03 | -5.57 | -4.50 |
| Both | UK | Leukemia | -0.78 | -0.94 | -0.61 |
| Both | UK | Other leukemia | 0.11 | -0.04 | 0.26 |
| Male | Ukraine | Acute lymphoid leukemia | -1.98 | -2.55 | -1.41 |
| Male | Ukraine | Chronic lymphoid leukemia | 2.03 | 1.32 | 2.74 |
| Male | Ukraine | Acute myeloid leukemia | -0.23 | -0.81 | 0.34 |
| Male | Ukraine | Chronic myeloid leukemia | -1.25 | -1.83 | -0.67 |
| Male | Ukraine | Leukemia | -0.25 | -0.63 | 0.14 |
| Male | Ukraine | Other leukemia | -0.38 | -0.79 | 0.04 |
| Female | Ukraine | Acute lymphoid leukemia | -2.11 | -2.81 | -1.40 |
| Female | Ukraine | Chronic lymphoid leukemia | 1.09 | 0.53 | 1.66 |
| Female | Ukraine | Acute myeloid leukemia | -1.86 | -2.62 | -1.09 |
| Female | Ukraine | Chronic myeloid leukemia | -2.01 | -2.52 | -1.49 |
| Female | Ukraine | Leukemia | -0.94 | -1.32 | -0.55 |
| Female | Ukraine | Other leukemia | -0.39 | -0.63 | -0.15 |
| Both | Ukraine | Acute lymphoid leukemia | -2.08 | -2.71 | -1.46 |
| Both | Ukraine | Chronic lymphoid leukemia | 1.68 | 1.03 | 2.33 |
| Both | Ukraine | Acute myeloid leukemia | -1.02 | -1.69 | -0.34 |
| Both | Ukraine | Chronic myeloid leukemia | -1.59 | -2.15 | -1.02 |
| Both | Ukraine | Leukemia | -0.56 | -0.94 | -0.17 |
| Both | Ukraine | Other leukemia | -0.36 | -0.67 | -0.04 |
| Male | United Arab Emirates | Acute lymphoid leukemia | 0.48 | 0.39 | 0.57 |
| Male | United Arab Emirates | Chronic lymphoid leukemia | 1.29 | 1.13 | 1.44 |
| Male | United Arab Emirates | Acute myeloid leukemia | -0.32 | -0.45 | -0.19 |
| Male | United Arab Emirates | Chronic myeloid leukemia | 0.29 | 0.12 | 0.45 |
| Male | United Arab Emirates | Leukemia | 0.45 | 0.35 | 0.56 |
| Male | United Arab Emirates | Other leukemia | 0.64 | 0.52 | 0.77 |
| Female | United Arab Emirates | Acute lymphoid leukemia | 0.19 | 0.13 | 0.24 |
| Female | United Arab Emirates | Chronic lymphoid leukemia | 1.63 | 1.20 | 2.06 |
| Female | United Arab Emirates | Acute myeloid leukemia | -0.71 | -0.77 | -0.66 |
| Female | United Arab Emirates | Chronic myeloid leukemia | -1.74 | -1.91 | -1.57 |
| Female | United Arab Emirates | Leukemia | -0.23 | -0.36 | -0.11 |
| Female | United Arab Emirates | Other leukemia | -0.26 | -0.40 | -0.11 |
| Both | United Arab Emirates | Acute lymphoid leukemia | 0.50 | 0.42 | 0.57 |
| Both | United Arab Emirates | Chronic lymphoid leukemia | 1.36 | 1.11 | 1.61 |
| Both | United Arab Emirates | Acute myeloid leukemia | -0.48 | -0.57 | -0.39 |
| Both | United Arab Emirates | Chronic myeloid leukemia | -0.36 | -0.50 | -0.22 |
| Both | United Arab Emirates | Leukemia | 0.26 | 0.16 | 0.36 |
| Both | United Arab Emirates | Other leukemia | 0.45 | 0.33 | 0.56 |
| Male | Uruguay | Acute lymphoid leukemia | -2.20 | -2.43 | -1.97 |
| Male | Uruguay | Chronic lymphoid leukemia | 0.63 | 0.39 | 0.86 |
| Male | Uruguay | Acute myeloid leukemia | -0.46 | -0.76 | -0.15 |
| Male | Uruguay | Chronic myeloid leukemia | -2.93 | -3.19 | -2.67 |
| Male | Uruguay | Leukemia | -0.70 | -0.81 | -0.58 |
| Male | Uruguay | Other leukemia | -0.50 | -0.79 | -0.21 |
| Female | Uruguay | Acute lymphoid leukemia | -1.51 | -1.80 | -1.23 |
| Female | Uruguay | Chronic lymphoid leukemia | 1.04 | 0.75 | 1.33 |
| Female | Uruguay | Acute myeloid leukemia | -0.51 | -0.64 | -0.37 |

| | | | | | |
|---|---|---|---|---|---|
| Female | Uruguay | Chronic myeloid leukemia | -3.46 | -3.92 | -3.01 |
| Female | Uruguay | Leukemia | -0.55 | -0.70 | -0.39 |
| Female | Uruguay | Other leukemia | -0.01 | -0.23 | 0.21 |
| Both | Uruguay | Acute lymphoid leukemia | -1.93 | -2.14 | -1.72 |
| Both | Uruguay | Chronic lymphoid leukemia | 0.72 | 0.49 | 0.95 |
| Both | Uruguay | Acute myeloid leukemia | -0.50 | -0.68 | -0.32 |
| Both | Uruguay | Chronic myeloid leukemia | -3.13 | -3.38 | -2.89 |
| Both | Uruguay | Leukemia | -0.70 | -0.81 | -0.59 |
| Both | Uruguay | Other leukemia | -0.41 | -0.62 | -0.19 |
| Male | USA | Acute lymphoid leukemia | -1.40 | -1.51 | -1.30 |
| Male | USA | Chronic lymphoid leukemia | -0.44 | -0.74 | -0.13 |
| Male | USA | Acute myeloid leukemia | 1.09 | 0.82 | 1.36 |
| Male | USA | Chronic myeloid leukemia | -4.35 | -4.70 | -3.99 |
| Male | USA | Leukemia | -0.65 | -0.85 | -0.45 |
| Male | USA | Other leukemia | -1.09 | -1.34 | -0.85 |
| Female | USA | Acute lymphoid leukemia | -1.30 | -1.36 | -1.24 |
| Female | USA | Chronic lymphoid leukemia | -0.13 | -0.49 | 0.24 |
| Female | USA | Acute myeloid leukemia | 1.03 | 0.75 | 1.31 |
| Female | USA | Chronic myeloid leukemia | -4.33 | -4.73 | -3.93 |
| Female | USA | Leukemia | -0.62 | -0.79 | -0.44 |
| Female | USA | Other leukemia | -1.44 | -1.58 | -1.30 |
| Both | USA | Acute lymphoid leukemia | -1.35 | -1.43 | -1.27 |
| Both | USA | Chronic lymphoid leukemia | -0.24 | -0.57 | 0.09 |
| Both | USA | Acute myeloid leukemia | 1.10 | 0.83 | 1.37 |
| Both | USA | Chronic myeloid leukemia | -4.29 | -4.66 | -3.92 |
| Both | USA | Leukemia | -0.59 | -0.78 | -0.40 |
| Both | USA | Other leukemia | -1.19 | -1.39 | -0.99 |
| Male | Uzbekistan | Acute lymphoid leukemia | 1.16 | 0.96 | 1.35 |
| Male | Uzbekistan | Chronic lymphoid leukemia | 1.96 | 1.62 | 2.30 |
| Male | Uzbekistan | Acute myeloid leukemia | 1.09 | 0.87 | 1.32 |
| Male | Uzbekistan | Chronic myeloid leukemia | -0.13 | -0.28 | 0.01 |
| Male | Uzbekistan | Leukemia | 0.07 | -0.04 | 0.17 |
| Male | Uzbekistan | Other leukemia | -1.39 | -1.67 | -1.11 |
| Female | Uzbekistan | Acute lymphoid leukemia | -0.32 | -0.53 | -0.10 |
| Female | Uzbekistan | Chronic lymphoid leukemia | 0.34 | 0.25 | 0.44 |
| Female | Uzbekistan | Acute myeloid leukemia | 0.51 | 0.25 | 0.77 |
| Female | Uzbekistan | Chronic myeloid leukemia | -0.95 | -1.37 | -0.52 |
| Female | Uzbekistan | Leukemia | -0.73 | -1.01 | -0.46 |
| Female | Uzbekistan | Other leukemia | -2.05 | -2.48 | -1.61 |
| Both | Uzbekistan | Acute lymphoid leukemia | 0.56 | 0.38 | 0.74 |
| Both | Uzbekistan | Chronic lymphoid leukemia | 1.06 | 0.90 | 1.23 |
| Both | Uzbekistan | Acute myeloid leukemia | 0.79 | 0.63 | 0.96 |
| Both | Uzbekistan | Chronic myeloid leukemia | -0.65 | -0.94 | -0.36 |
| Both | Uzbekistan | Leukemia | -0.34 | -0.52 | -0.17 |
| Both | Uzbekistan | Other leukemia | -1.78 | -2.14 | -1.41 |
| Male | Vanuatu | Acute lymphoid leukemia | 0.23 | 0.14 | 0.31 |
| Male | Vanuatu | Chronic lymphoid leukemia | -0.10 | -0.15 | -0.05 |
| Male | Vanuatu | Acute myeloid leukemia | 1.11 | 1.01 | 1.21 |

| | | | | | |
|---|---|---|---|---|---|
| Male | Vanuatu | Chronic myeloid leukemia | -1.08 | -1.13 | -1.03 |
| Male | Vanuatu | Leukemia | 0.55 | 0.48 | 0.62 |
| Male | Vanuatu | Other leukemia | 0.41 | 0.33 | 0.49 |
| Female | Vanuatu | Acute lymphoid leukemia | 0.59 | 0.42 | 0.76 |
| Female | Vanuatu | Chronic lymphoid leukemia | 0.59 | 0.55 | 0.63 |
| Female | Vanuatu | Acute myeloid leukemia | 0.13 | 0.00 | 0.26 |
| Female | Vanuatu | Chronic myeloid leukemia | -0.96 | -1.08 | -0.84 |
| Female | Vanuatu | Leukemia | 0.03 | -0.07 | 0.13 |
| Female | Vanuatu | Other leukemia | -0.02 | -0.13 | 0.09 |
| Both | Vanuatu | Acute lymphoid leukemia | 0.39 | 0.27 | 0.51 |
| Both | Vanuatu | Chronic lymphoid leukemia | 0.65 | 0.61 | 0.69 |
| Both | Vanuatu | Acute myeloid leukemia | 0.74 | 0.64 | 0.85 |
| Both | Vanuatu | Chronic myeloid leukemia | -0.97 | -1.04 | -0.89 |
| Both | Vanuatu | Leukemia | 0.31 | 0.23 | 0.39 |
| Both | Vanuatu | Other leukemia | 0.19 | 0.11 | 0.28 |
| Male | Venezuela | Acute lymphoid leukemia | 0.30 | 0.15 | 0.44 |
| Male | Venezuela | Chronic lymphoid leukemia | 0.58 | 0.43 | 0.73 |
| Male | Venezuela | Acute myeloid leukemia | 0.50 | 0.35 | 0.65 |
| Male | Venezuela | Chronic myeloid leukemia | -1.77 | -1.95 | -1.60 |
| Male | Venezuela | Leukemia | -0.52 | -0.63 | -0.41 |
| Male | Venezuela | Other leukemia | -2.49 | -2.88 | -2.10 |
| Female | Venezuela | Acute lymphoid leukemia | 0.31 | 0.14 | 0.48 |
| Female | Venezuela | Chronic lymphoid leukemia | -0.44 | -0.63 | -0.26 |
| Female | Venezuela | Acute myeloid leukemia | 0.54 | 0.36 | 0.71 |
| Female | Venezuela | Chronic myeloid leukemia | -3.14 | -3.47 | -2.81 |
| Female | Venezuela | Leukemia | -0.78 | -0.88 | -0.67 |
| Female | Venezuela | Other leukemia | -2.59 | -3.01 | -2.17 |
| Both | Venezuela | Acute lymphoid leukemia | 0.30 | 0.16 | 0.45 |
| Both | Venezuela | Chronic lymphoid leukemia | 0.13 | 0.01 | 0.24 |
| Both | Venezuela | Acute myeloid leukemia | 0.51 | 0.37 | 0.66 |
| Both | Venezuela | Chronic myeloid leukemia | -2.34 | -2.53 | -2.14 |
| Both | Venezuela | Leukemia | -0.64 | -0.74 | -0.54 |
| Both | Venezuela | Other leukemia | -2.53 | -2.92 | -2.14 |
| Male | Vietnam | Acute lymphoid leukemia | 0.89 | 0.70 | 1.07 |
| Male | Vietnam | Chronic lymphoid leukemia | 2.87 | 2.69 | 3.05 |
| Male | Vietnam | Acute myeloid leukemia | 1.57 | 1.43 | 1.71 |
| Male | Vietnam | Chronic myeloid leukemia | 1.43 | 1.27 | 1.60 |
| Male | Vietnam | Leukemia | 1.14 | 0.99 | 1.29 |
| Male | Vietnam | Other leukemia | 0.80 | 0.65 | 0.95 |
| Female | Vietnam | Acute lymphoid leukemia | 0.16 | 0.01 | 0.31 |
| Female | Vietnam | Chronic lymphoid leukemia | 2.64 | 2.49 | 2.79 |
| Female | Vietnam | Acute myeloid leukemia | 0.51 | 0.43 | 0.59 |
| Female | Vietnam | Chronic myeloid leukemia | -0.75 | -0.81 | -0.68 |
| Female | Vietnam | Leukemia | -0.14 | -0.24 | -0.05 |
| Female | Vietnam | Other leukemia | -0.96 | -1.08 | -0.85 |
| Both | Vietnam | Acute lymphoid leukemia | 0.53 | 0.37 | 0.69 |
| Both | Vietnam | Chronic lymphoid leukemia | 2.72 | 2.57 | 2.87 |
| Both | Vietnam | Acute myeloid leukemia | 1.20 | 1.09 | 1.31 |

| | | | | | |
|---|---|---|---|---|---|
| Both | Vietnam | Chronic myeloid leukemia | 0.60 | 0.48 | 0.73 |
| Both | Vietnam | Leukemia | 0.57 | 0.45 | 0.70 |
| Both | Vietnam | Other leukemia | -0.05 | -0.18 | 0.08 |
| Male | Virgin Islands, U.S. | Acute lymphoid leukemia | -0.87 | -1.23 | -0.52 |
| Male | Virgin Islands, U.S. | Chronic lymphoid leukemia | 1.35 | 1.13 | 1.56 |
| Male | Virgin Islands, U.S. | Acute myeloid leukemia | 0.83 | 0.51 | 1.15 |
| Male | Virgin Islands, U.S. | Chronic myeloid leukemia | -1.95 | -2.26 | -1.64 |
| Male | Virgin Islands, U.S. | Leukemia | 0.58 | 0.43 | 0.73 |
| Male | Virgin Islands, U.S. | Other leukemia | 0.17 | 0.06 | 0.29 |
| Female | Virgin Islands, U.S. | Acute lymphoid leukemia | -1.81 | -2.15 | -1.46 |
| Female | Virgin Islands, U.S. | Chronic lymphoid leukemia | 0.37 | 0.15 | 0.60 |
| Female | Virgin Islands, U.S. | Acute myeloid leukemia | 0.17 | 0.01 | 0.32 |
| Female | Virgin Islands, U.S. | Chronic myeloid leukemia | -2.00 | -2.33 | -1.67 |
| Female | Virgin Islands, U.S. | Leukemia | -0.11 | -0.21 | -0.02 |
| Female | Virgin Islands, U.S. | Other leukemia | -0.03 | -0.33 | 0.27 |
| Both | Virgin Islands, U.S. | Acute lymphoid leukemia | -1.05 | -1.41 | -0.70 |
| Both | Virgin Islands, U.S. | Chronic lymphoid leukemia | 1.01 | 0.79 | 1.23 |
| Both | Virgin Islands, U.S. | Acute myeloid leukemia | 0.45 | 0.24 | 0.67 |
| Both | Virgin Islands, U.S. | Chronic myeloid leukemia | -1.98 | -2.29 | -1.66 |
| Both | Virgin Islands, U.S. | Leukemia | 0.28 | 0.15 | 0.42 |
| Both | Virgin Islands, U.S. | Other leukemia | 0.10 | -0.06 | 0.26 |
| Male | Western Europe | Acute lymphoid leukemia | -2.22 | -2.37 | -2.07 |
| Male | Western Europe | Chronic lymphoid leukemia | -0.01 | -0.24 | 0.22 |
| Male | Western Europe | Acute myeloid leukemia | 0.40 | 0.33 | 0.47 |
| Male | Western Europe | Chronic myeloid leukemia | -3.75 | -3.96 | -3.53 |
| Male | Western Europe | Leukemia | -0.76 | -0.85 | -0.67 |
| Male | Western Europe | Other leukemia | -1.02 | -1.18 | -0.86 |
| Female | Western Europe | Acute lymphoid leukemia | -2.05 | -2.18 | -1.93 |
| Female | Western Europe | Chronic lymphoid leukemia | 0.16 | -0.02 | 0.35 |
| Female | Western Europe | Acute myeloid leukemia | 0.35 | 0.24 | 0.46 |
| Female | Western Europe | Chronic myeloid leukemia | -4.74 | -4.99 | -4.48 |
| Female | Western Europe | Leukemia | -0.88 | -0.93 | -0.83 |
| Female | Western Europe | Other leukemia | -1.47 | -1.62 | -1.31 |
| Both | Western Europe | Acute lymphoid leukemia | -2.14 | -2.28 | -2.00 |
| Both | Western Europe | Chronic lymphoid leukemia | 0.14 | -0.07 | 0.35 |
| Both | Western Europe | Acute myeloid leukemia | 0.40 | 0.32 | 0.48 |
| Both | Western Europe | Chronic myeloid leukemia | -4.12 | -4.34 | -3.89 |
| Both | Western Europe | Leukemia | -0.76 | -0.83 | -0.69 |
| Both | Western Europe | Other leukemia | -1.13 | -1.29 | -0.97 |
| Male | Western Sub-Saharan Africa | Acute lymphoid leukemia | -2.25 | -2.63 | -1.87 |
| Male | Western Sub-Saharan Africa | Chronic lymphoid leukemia | 1.48 | 1.38 | 1.57 |
| Male | Western Sub-Saharan Africa | Acute myeloid leukemia | 0.43 | 0.24 | 0.61 |
| Male | Western Sub-Saharan Africa | Chronic myeloid leukemia | 0.39 | 0.35 | 0.43 |
| Male | Western Sub-Saharan Africa | Leukemia | -0.02 | -0.16 | 0.12 |
| Male | Western Sub-Saharan Africa | Other leukemia | 0.07 | -0.01 | 0.14 |
| Female | Western Sub-Saharan Africa | Acute lymphoid leukemia | 0.39 | 0.34 | 0.43 |
| Female | Western Sub-Saharan Africa | Chronic lymphoid leukemia | 0.16 | 0.13 | 0.18 |
| Female | Western Sub-Saharan Africa | Acute myeloid leukemia | 0.55 | 0.52 | 0.59 |

| | | | | | |
|---|---|---|---|---|---|
| Female | Western Sub-Saharan Africa | Chronic myeloid leukemia | -0.97 | -1.06 | -0.87 |
| Female | Western Sub-Saharan Africa | Leukemia | -0.31 | -0.35 | -0.27 |
| Female | Western Sub-Saharan Africa | Other leukemia | -1.03 | -1.11 | -0.95 |
| Both | Western Sub-Saharan Africa | Acute lymphoid leukemia | -1.36 | -1.61 | -1.11 |
| Both | Western Sub-Saharan Africa | Chronic lymphoid leukemia | 0.67 | 0.61 | 0.74 |
| Both | Western Sub-Saharan Africa | Acute myeloid leukemia | 0.44 | 0.34 | 0.54 |
| Both | Western Sub-Saharan Africa | Chronic myeloid leukemia | -0.13 | -0.16 | -0.11 |
| Both | Western Sub-Saharan Africa | Other leukemia | -0.42 | -0.48 | -0.36 |
| Both | Western Sub-Saharan Africa | Leukemia | -0.14 | -0.23 | -0.05 |
| Male | Yemen | Acute lymphoid leukemia | 0.68 | 0.57 | 0.79 |
| Male | Yemen | Chronic lymphoid leukemia | 1.20 | 1.13 | 1.27 |
| Male | Yemen | Acute myeloid leukemia | 0.93 | 0.80 | 1.07 |
| Male | Yemen | Chronic myeloid leukemia | 0.14 | 0.05 | 0.22 |
| Male | Yemen | Leukemia | 0.77 | 0.68 | 0.87 |
| Male | Yemen | Other leukemia | 0.79 | 0.68 | 0.90 |
| Female | Yemen | Acute lymphoid leukemia | 0.97 | 0.83 | 1.10 |
| Female | Yemen | Chronic lymphoid leukemia | 0.84 | 0.79 | 0.90 |
| Female | Yemen | Acute myeloid leukemia | -0.03 | -0.12 | 0.05 |
| Female | Yemen | Chronic myeloid leukemia | -1.46 | -1.57 | -1.34 |
| Female | Yemen | Leukemia | -0.31 | -0.37 | -0.24 |
| Female | Yemen | Other leukemia | -0.54 | -0.61 | -0.47 |
| Both | Yemen | Acute lymphoid leukemia | 0.78 | 0.67 | 0.90 |
| Both | Yemen | Chronic lymphoid leukemia | 1.18 | 1.12 | 1.24 |
| Both | Yemen | Acute myeloid leukemia | 0.43 | 0.33 | 0.54 |
| Both | Yemen | Chronic myeloid leukemia | -0.70 | -0.79 | -0.61 |
| Both | Yemen | Leukemia | 0.28 | 0.20 | 0.37 |
| Both | Yemen | Other leukemia | 0.20 | 0.11 | 0.29 |
| Male | Zambia | Acute lymphoid leukemia | -0.56 | -0.73 | -0.40 |
| Male | Zambia | Chronic lymphoid leukemia | 2.05 | 1.90 | 2.21 |
| Male | Zambia | Acute myeloid leukemia | 0.02 | -0.12 | 0.16 |
| Male | Zambia | Chronic myeloid leukemia | -0.42 | -0.59 | -0.25 |
| Male | Zambia | Leukemia | -0.33 | -0.47 | -0.19 |
| Male | Zambia | Other leukemia | -1.25 | -1.47 | -1.04 |
| Female | Zambia | Acute lymphoid leukemia | 0.96 | 0.77 | 1.14 |
| Female | Zambia | Chronic lymphoid leukemia | -0.73 | -1.17 | -0.28 |
| Female | Zambia | Acute myeloid leukemia | 0.51 | 0.29 | 0.73 |
| Female | Zambia | Chronic myeloid leukemia | -1.84 | -2.12 | -1.56 |
| Female | Zambia | Leukemia | -1.02 | -1.22 | -0.82 |
| Female | Zambia | Other leukemia | -2.10 | -2.27 | -1.93 |
| Both | Zambia | Acute lymphoid leukemia | 0.01 | -0.16 | 0.17 |
| Both | Zambia | Chronic lymphoid leukemia | 0.59 | 0.40 | 0.78 |
| Both | Zambia | Acute myeloid leukemia | -0.08 | -0.18 | 0.03 |
| Both | Zambia | Chronic myeloid leukemia | -1.22 | -1.41 | -1.03 |
| Both | Zambia | Leukemia | -0.67 | -0.79 | -0.55 |
| Both | Zambia | Other leukemia | -1.69 | -1.85 | -1.53 |
| Male | Zimbabwe | Acute lymphoid leukemia | 1.85 | 1.21 | 2.50 |
| Male | Zimbabwe | Chronic lymphoid leukemia | 0.99 | 0.60 | 1.39 |
| Male | Zimbabwe | Acute myeloid leukemia | 0.48 | -0.02 | 0.99 |

| | | | | | |
|---|---|---|---|---|---|
| Male | Zimbabwe | Chronic myeloid leukemia | 2.01 | 1.19 | 2.83 |
| Male | Zimbabwe | Leukemia | 0.84 | 0.30 | 1.37 |
| Male | Zimbabwe | Other leukemia | 1.31 | 0.67 | 1.95 |
| Female | Zimbabwe | Acute lymphoid leukemia | -0.27 | -0.42 | -0.13 |
| Female | Zimbabwe | Chronic lymphoid leukemia | 0.92 | 0.52 | 1.32 |
| Female | Zimbabwe | Acute myeloid leukemia | 0.99 | 0.67 | 1.30 |
| Female | Zimbabwe | Chronic myeloid leukemia | 2.24 | 1.42 | 3.06 |
| Female | Zimbabwe | Leukemia | 1.38 | 0.83 | 1.92 |
| Female | Zimbabwe | Other leukemia | 1.53 | 0.92 | 2.15 |
| Both | Zimbabwe | Acute lymphoid leukemia | 0.83 | 0.49 | 1.17 |
| Both | Zimbabwe | Chronic lymphoid leukemia | 0.70 | 0.32 | 1.09 |
| Both | Zimbabwe | Acute myeloid leukemia | 0.35 | -0.12 | 0.81 |
| Both | Zimbabwe | Chronic myeloid leukemia | 2.16 | 1.35 | 2.97 |
| Both | Zimbabwe | Leukemia | 0.96 | 0.46 | 1.47 |
| Both | Zimbabwe | Other leukemia | 1.49 | 0.88 | 2.11 |
