# Supplementary material for: Leukemia incidence trends at the global, regional, and national level between 1990 and 2017
Source: Exp Hematol Oncol. 2020 Jun 19;9:14. doi: 10.1186/s40164-020-00170-6 (PMC7304189; doi:10.1186/s40164-020-00170-6)

Additional figures

Figure S1. The age-standardized incidence rate (ASIR) of ALL in 2017 and the changing trends of ALL ASIR between 1990 and 2017.


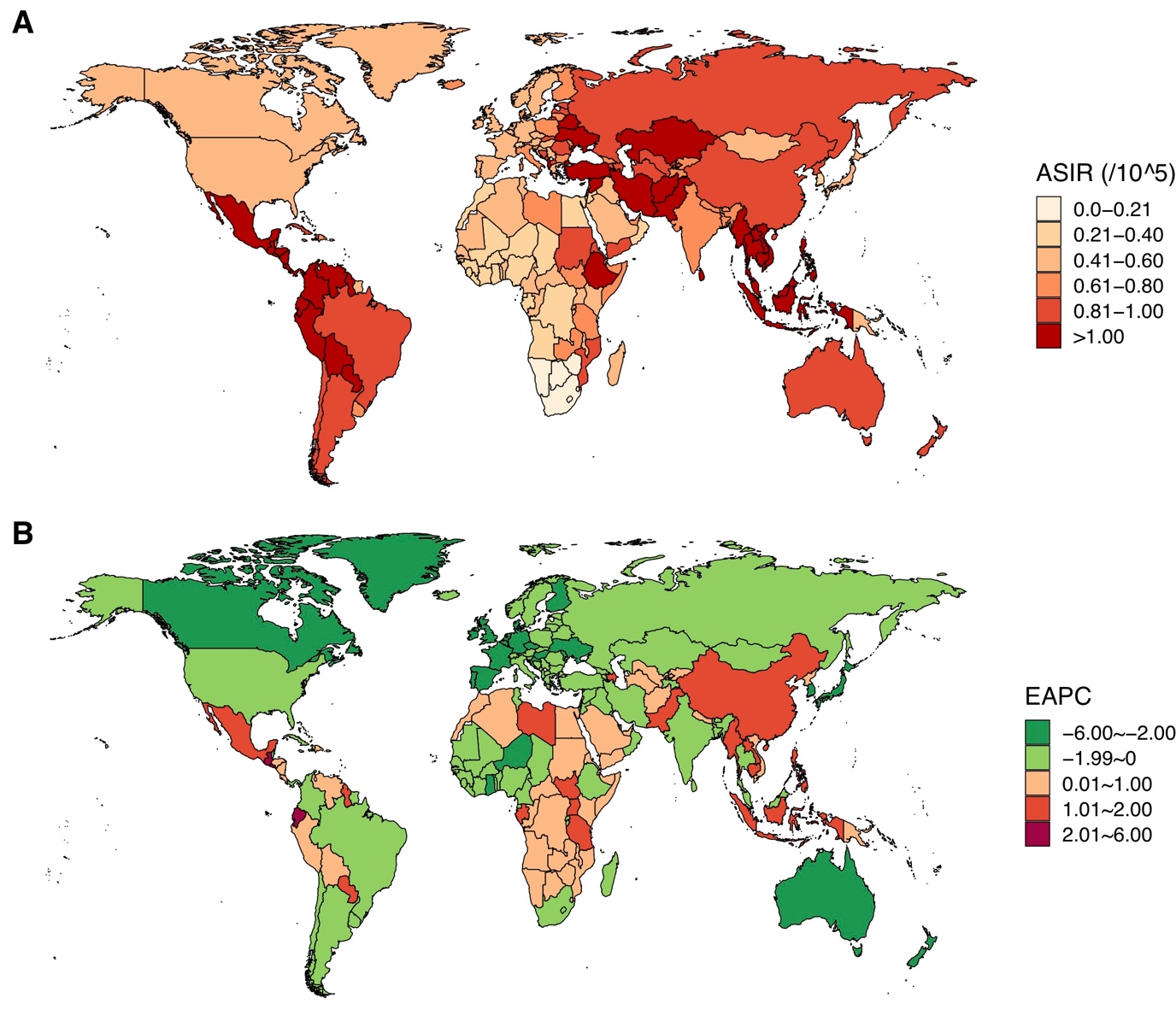


Figure S2. The age-standardized incidence rate (ASIR) of CLL in 2017 and the changing trends of CLL ASIR between 1990 and 2017.


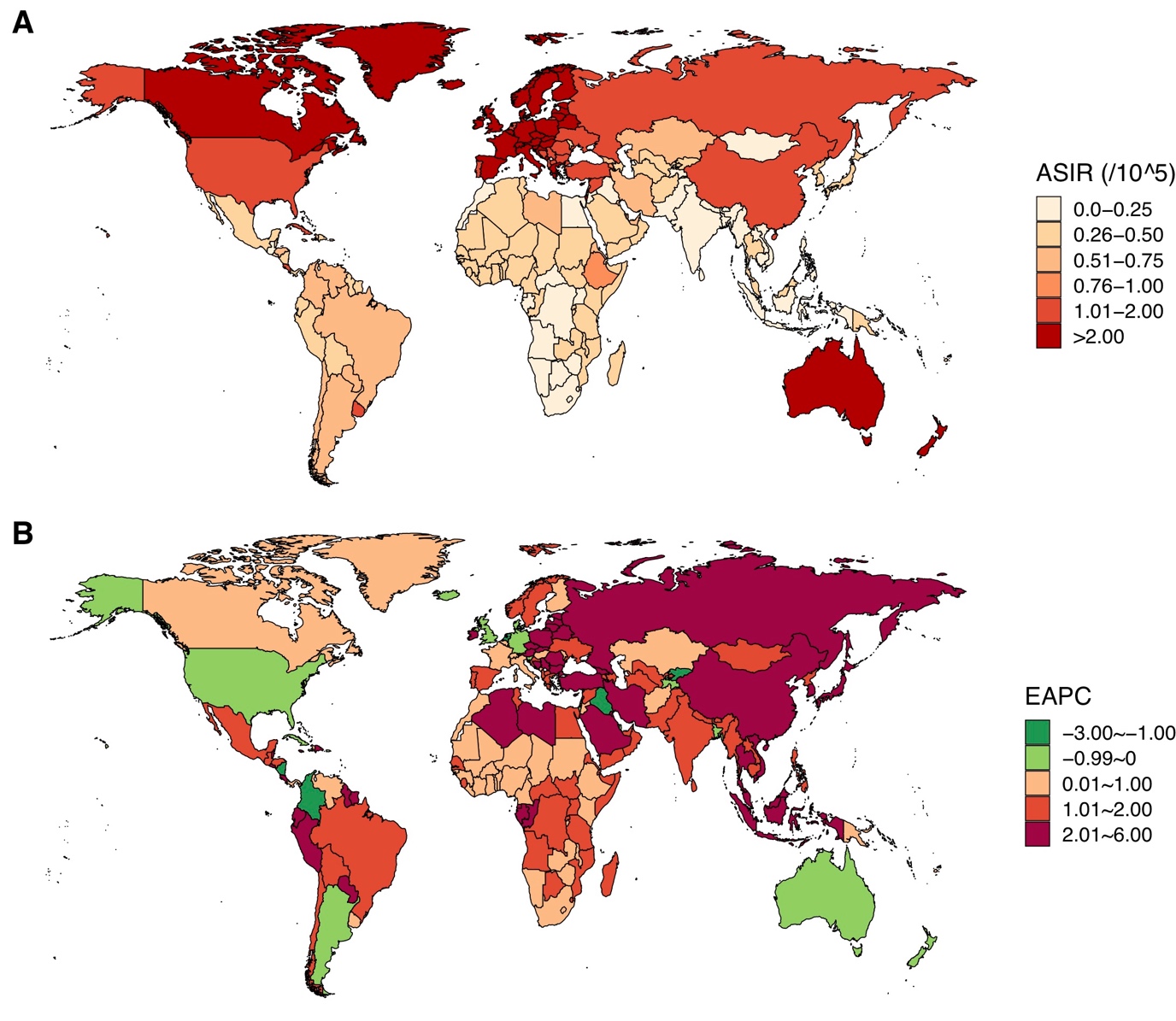


Figure S3. The age-standardized incidence rate (ASIR) of AML in 2017 and the changing trends of AML ASIR between 1990 and 2017.


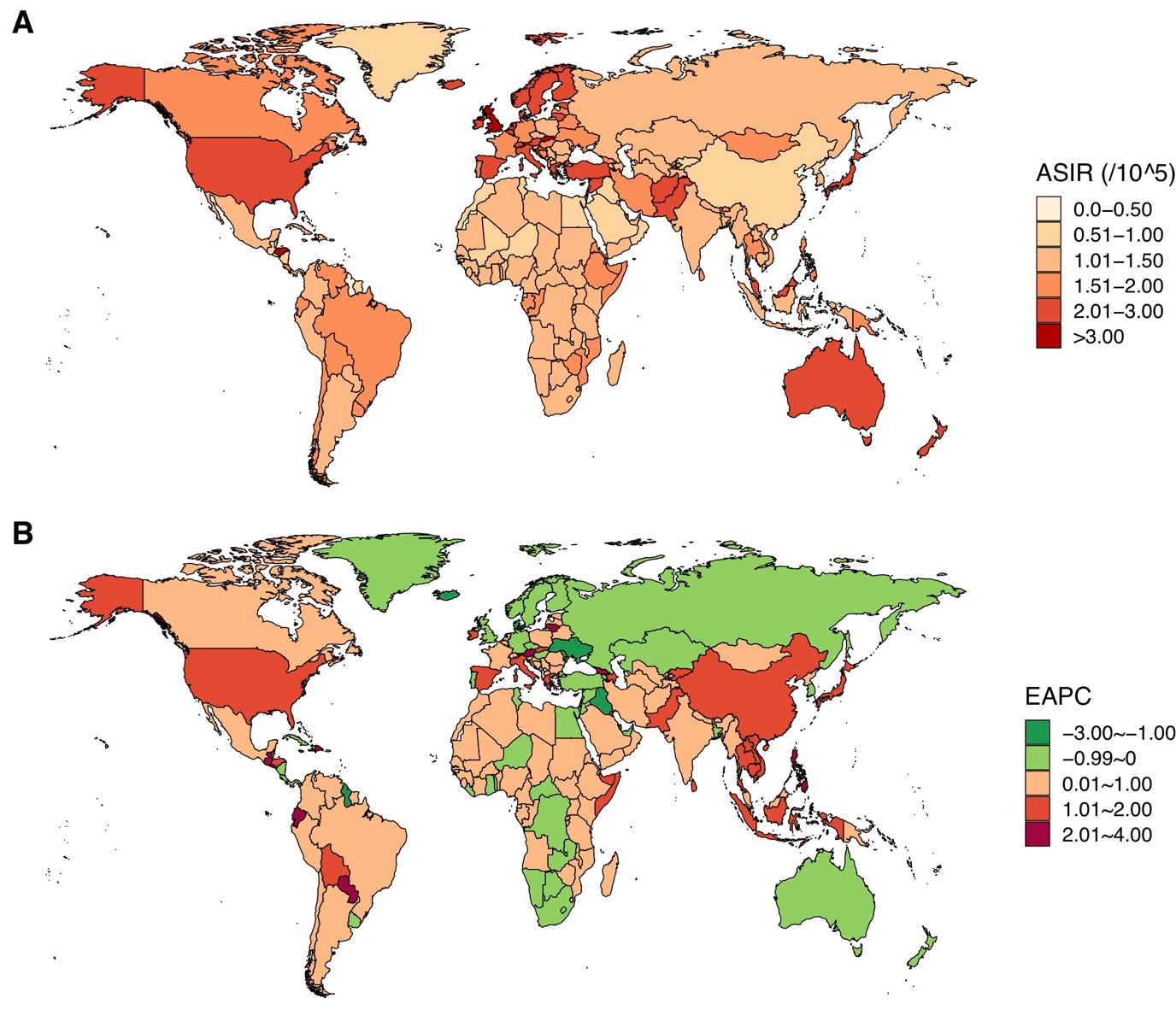


Figure S4. The age-standardized incidence rate (ASIR) of CML in 2017 and the changing trends of CML ASIR between 1990 and 2017.


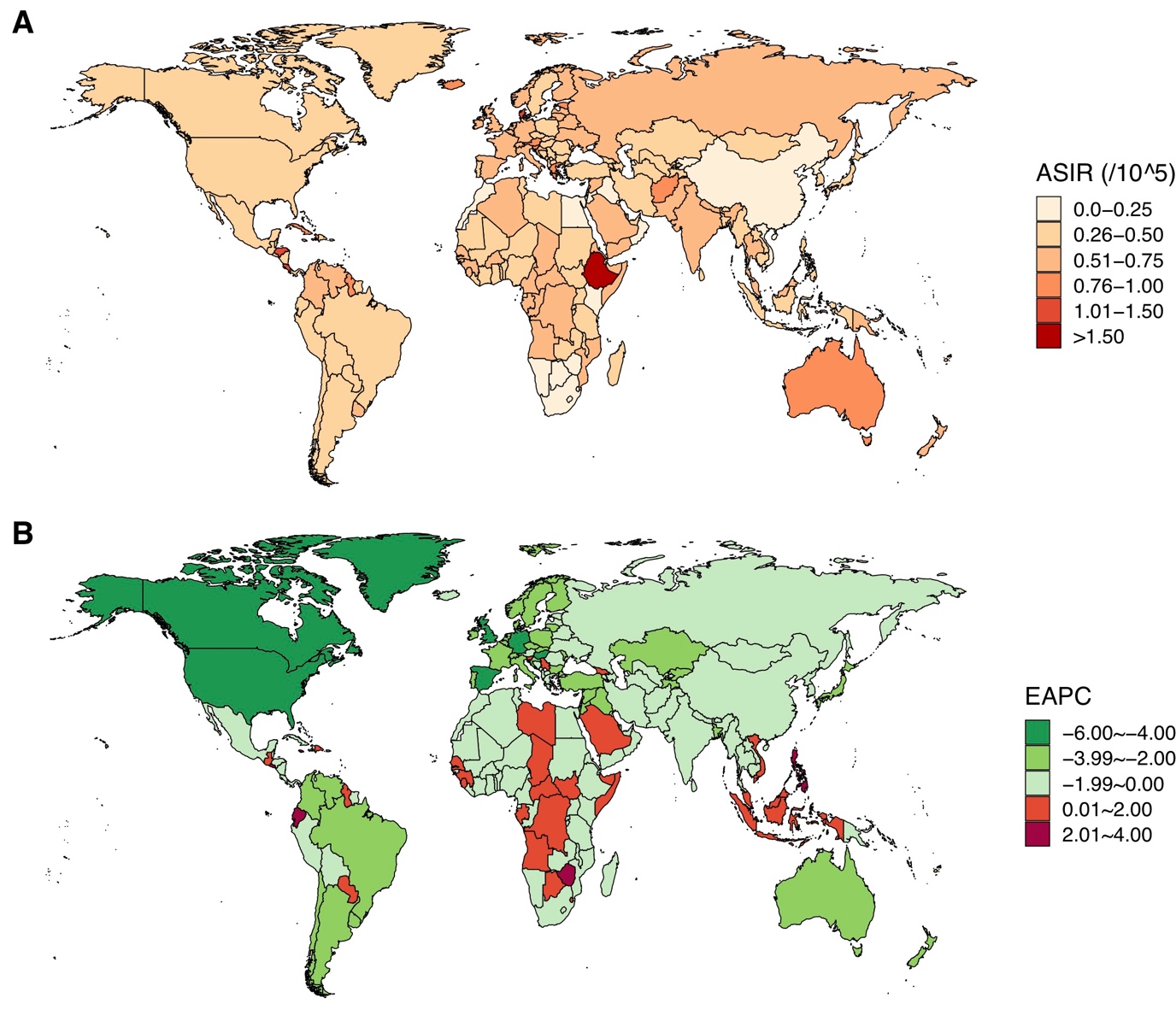


Figure S5. The age-standardized incidence rate (ASIR) of other leukemias in 2017 and the changing trends of other leukemias ASIR between 1990 and 2017.


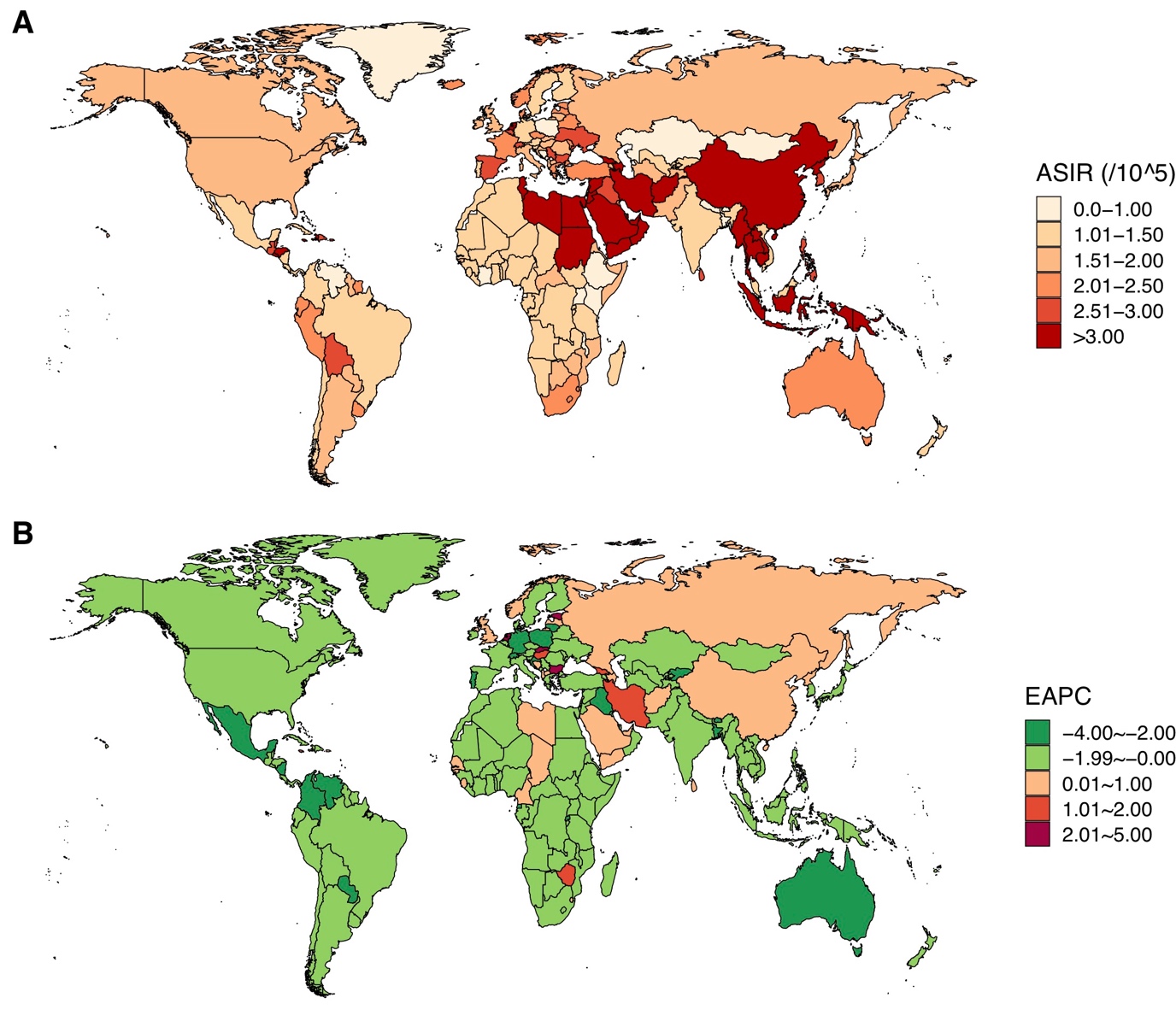

Supplement: Supplementary file 2 — Additional file 2. Supplementary Figures S1–S5. [file 40164_2020_170_MOESM2_ESM.docx]
